# Supplementary material for: A Convenient, Rapid, Conventional Heating Route to MIDA Boronates
Source: Molecules. 2022 Aug 9;27(16):5052. doi: 10.3390/molecules27165052 (PMC9414357; doi:10.3390/molecules27165052)
Supplement: Supplementary file 1 [file molecules-27-05052-s001.zip › molecules-1811037-supplementary.pdf]

A Convenient, Rapid, Conventional Heating Route to MIDA Boronates

Andrew McGown [1], Anthony K. Edmonds [1], Daniel Guest [1], Verity L. Holmes [1], Chris Dadswell [1], Ramón González-Méndez [1], Charles A. I. Goodhall [2], Mark C. Bagley [1], Barnaby W. Greenland [1], and John Spencer [1,*]

Supporting Information

***4-(6-Methyl-4,8-dioxo-1,3,6,2-dioxazaborocan-2-yl)benzenesulfonamide* 8a**

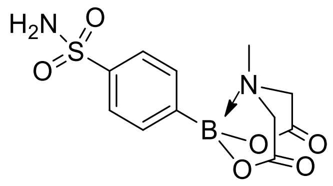

8a

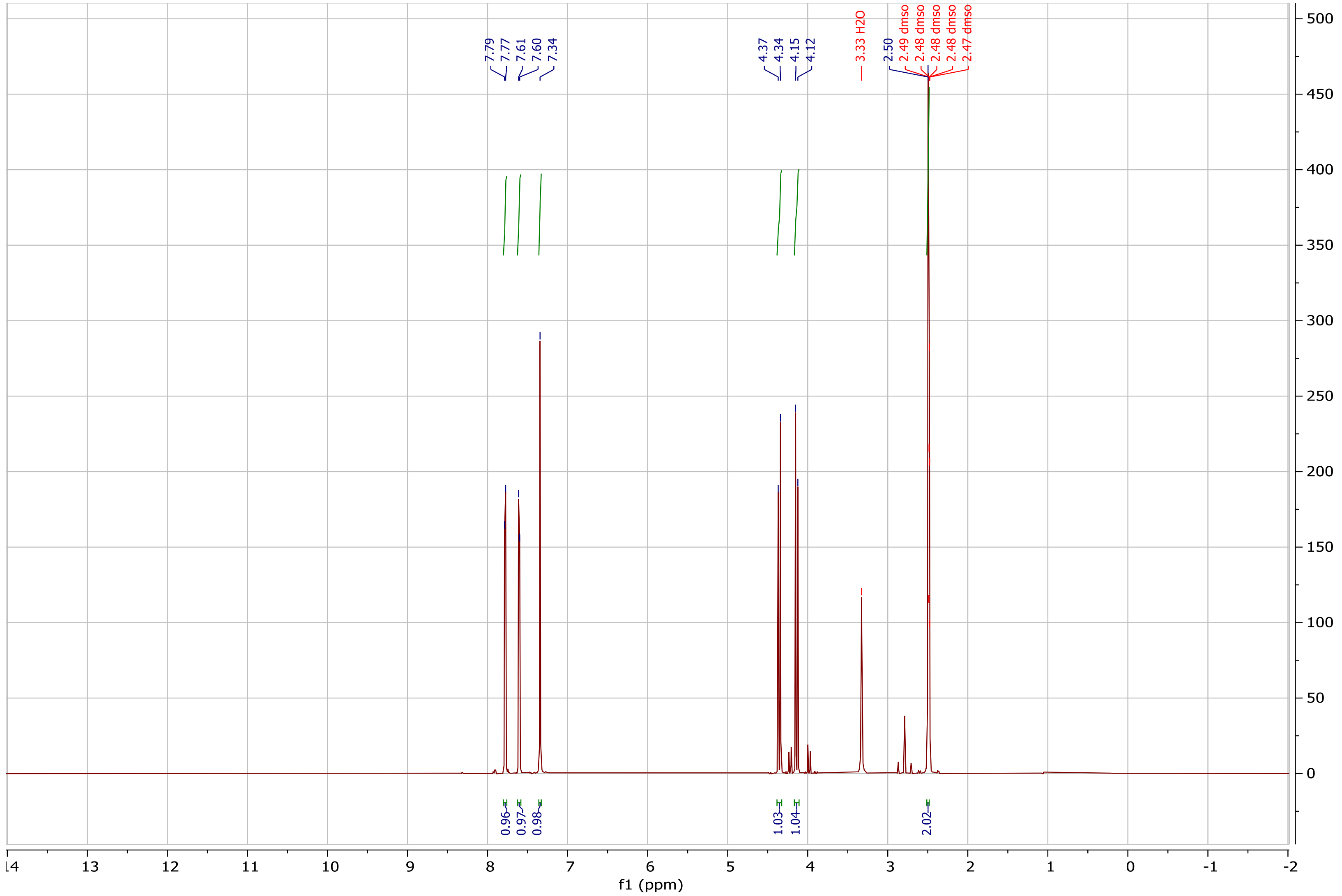

7.79
7.77
7.61
7.60
7.34
4.37
4.34
4.15
4.12
3.33 H2O
2.50
2.49 dmso
2.48 dmso
2.48 dmso
2.48 dmso
2.47 dmso
0.96
0.97
0.98
1.03
1.04
2.02
f1 (ppm)

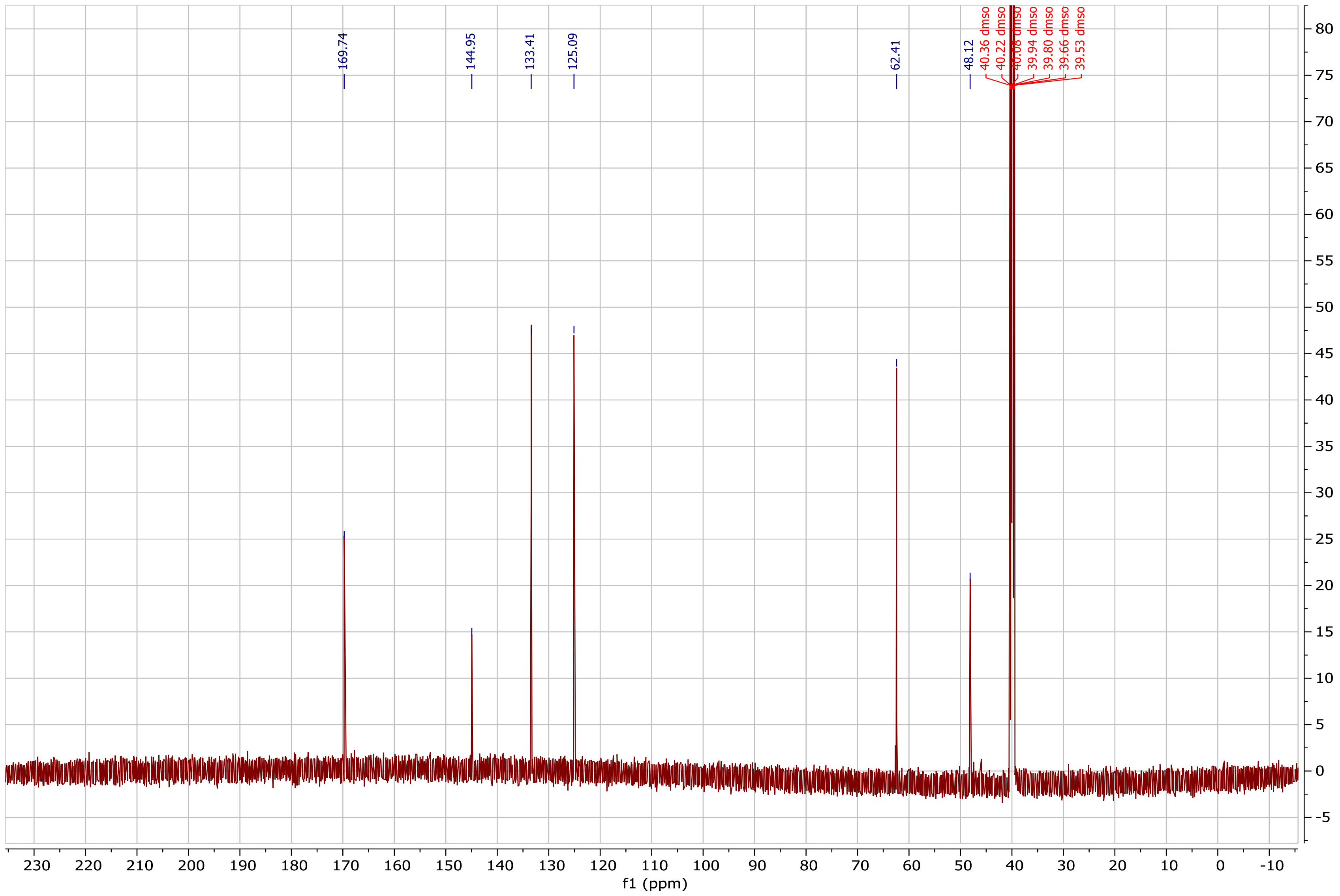
169.74
144.95
133.41
125.09
62.41
48.12
40.36 dmso
40.22 dmso
40.08 dmso
39.94 dmso
39.80 dmso
39.66 dmso
39.53 dmso
f1 (ppm)

Single Mass Analysis
Tolerance = 5.0 mDa / DBE: min = -1.5, max = 120.0
Element prediction: Off
Number of isotope peaks used for i-FIT = 3
Monoisotopic Mass, Even Electron Ions
109 formula(e) evaluated with 1 results within limits (up to 20 best isotopic matches for each mass)
Elements Used:

Mass	Calc. Mass	mDa	PPM	DBE	Formula	i-FIT	i-FIT Norm	Fit Conf %	C	H	11B	N	O	S
330.0939	330.0931	0.8	2.4	5.5	C11 H17 11B N3 O6 S	110.7	n/a	n/a	11	17	1	3	6	1

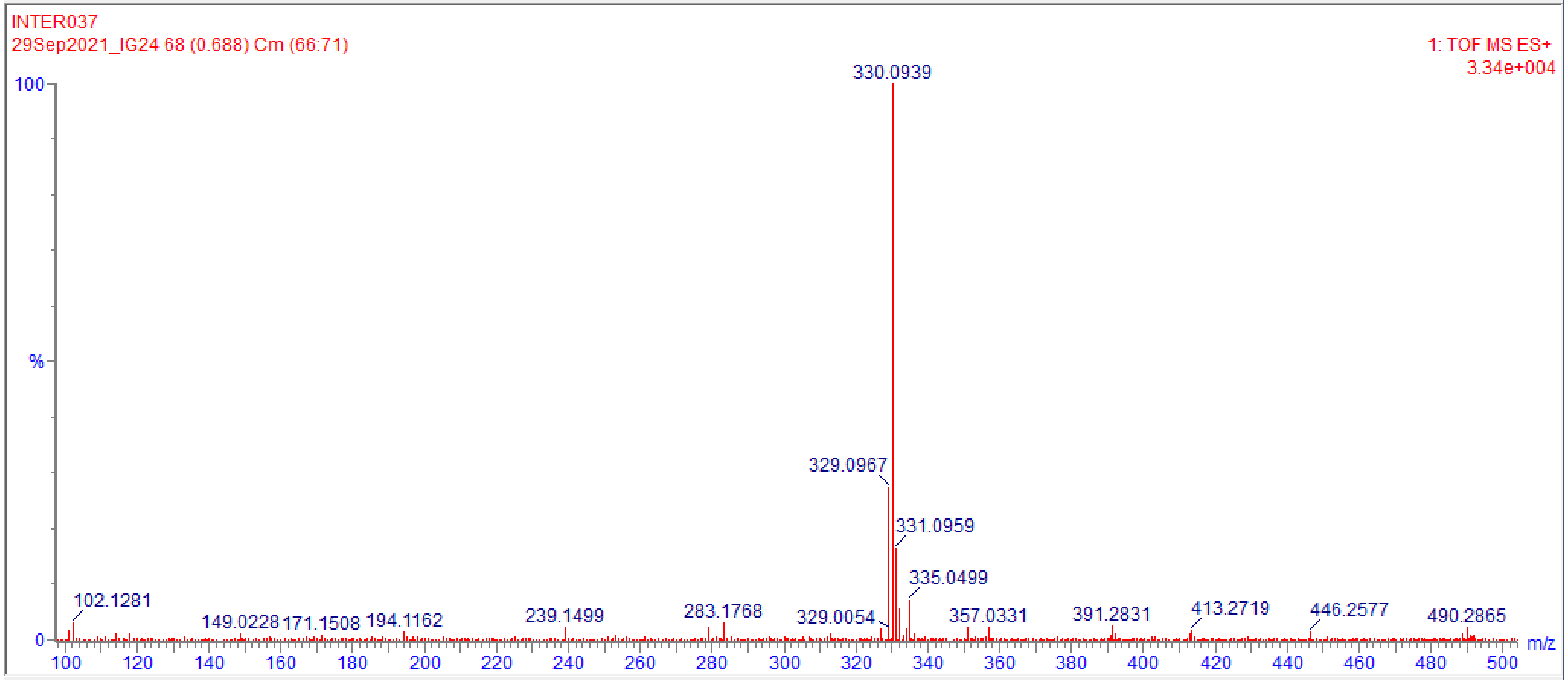

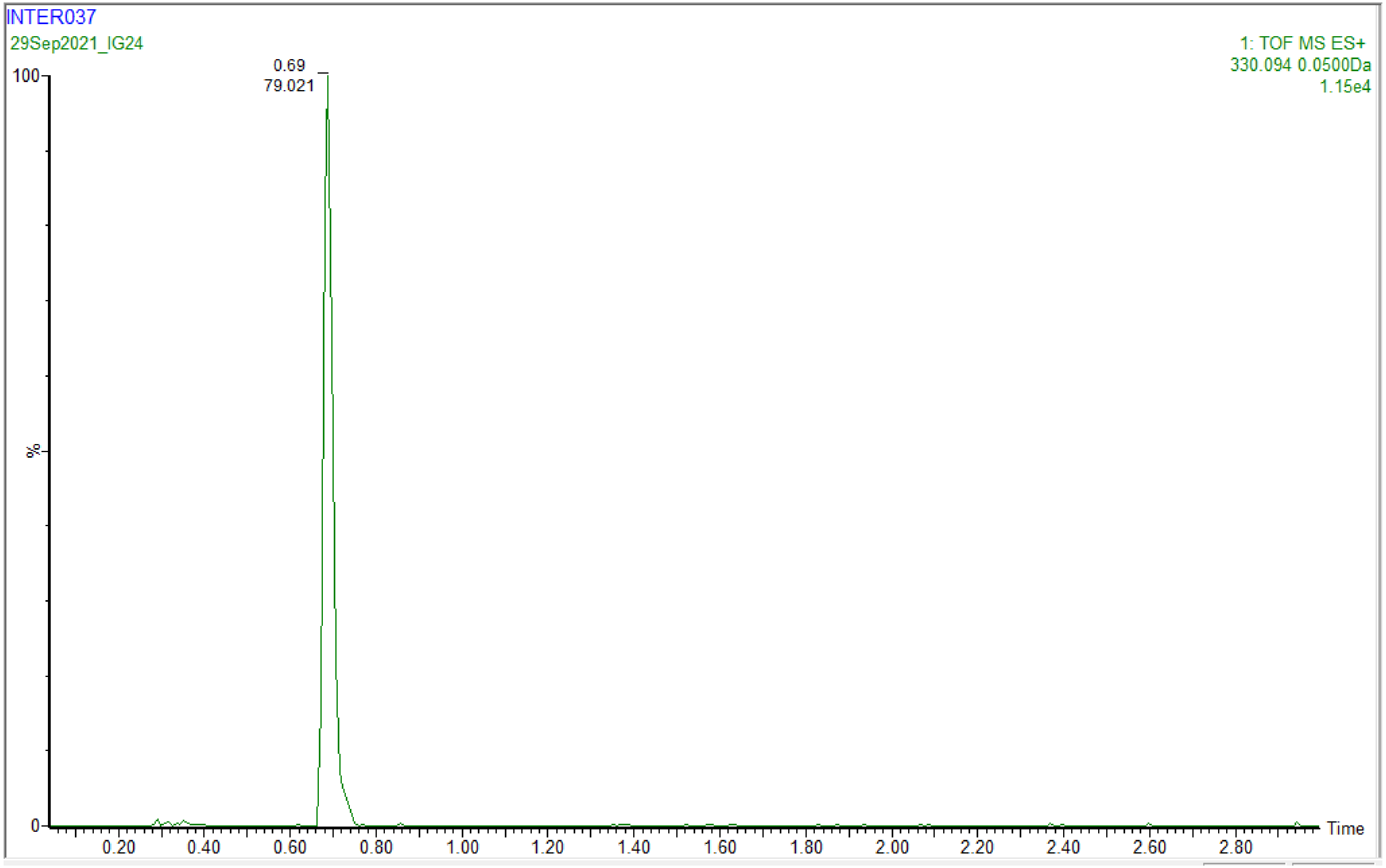

INTER037
29Sep2021_IG24
1: TOF MS ES+
330.094 0.0500Da
1.15e4
0.69
79.021
100
%
0
0.20
0.40
0.60
0.80
1.00
1.20
1.40
1.60
1.80
2.00
2.20
2.40
2.60
2.80
Time

2-(3-Fluoro-4-methoxyphenyl)-6-methyl-1,3,6,2-dioxazaborocane-4,8-dione **8b**

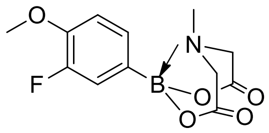

8b

2-(3-fluoro-4-methoxyphenyl)-6-methyl-1,3,6,2-dioxazaborocane-4,8-dione
Chemical Formula: $C_{12}H_{13}BFNO_5$
Molecular Weight: 281.0447

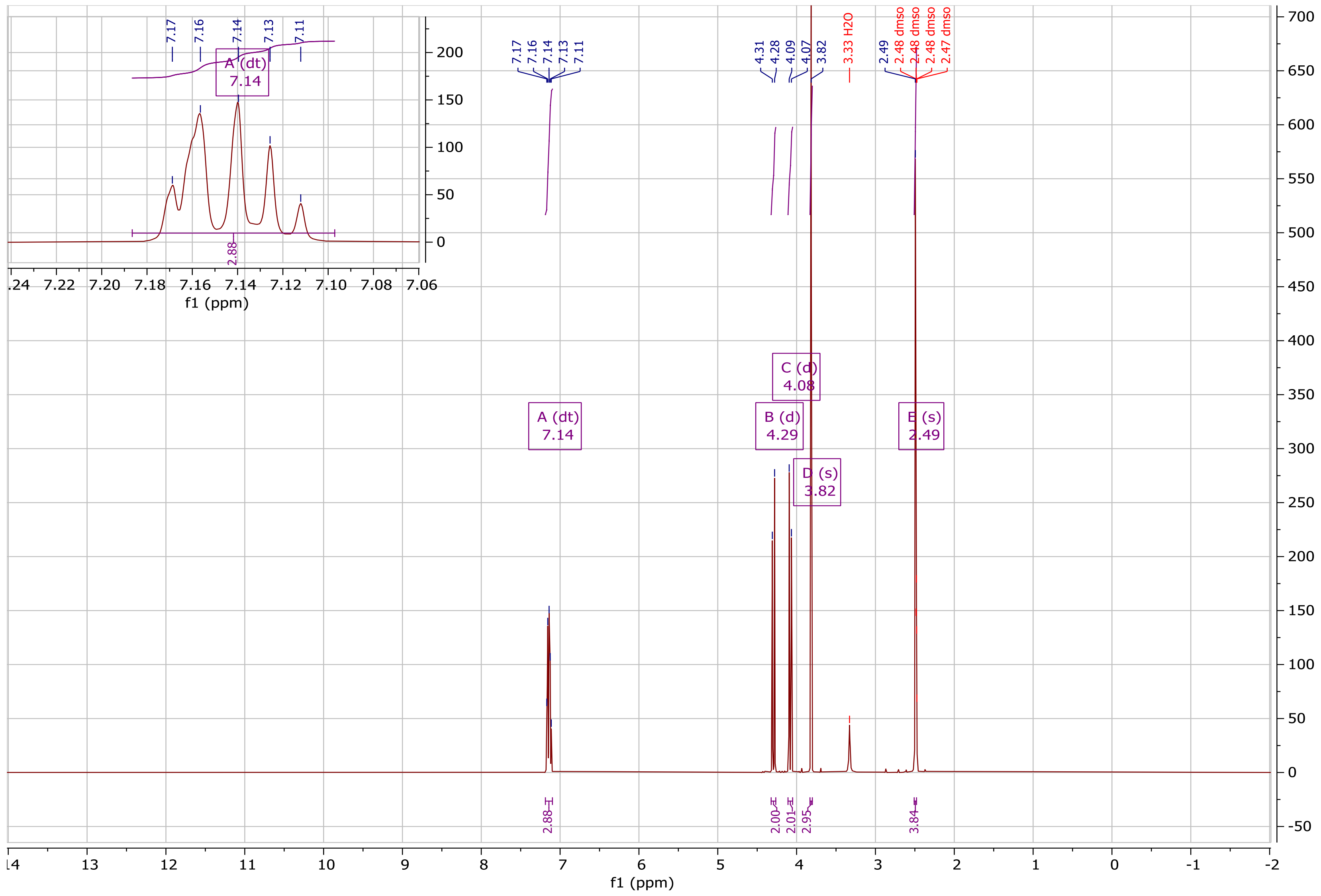

A (dt)
7.14
B (d)
4.29
C (d)
4.08
D (s)
3.82
E (s)
2.49
7.17
7.16
7.14
7.13
7.11
4.31
4.28
4.09
4.07
3.82
3.33 H2O
2.49
2.48 dmso
2.48 dmso
2.48 dmso
2.47 dmso
2.88
2.00
2.01
2.95
3.84
f1 (ppm)

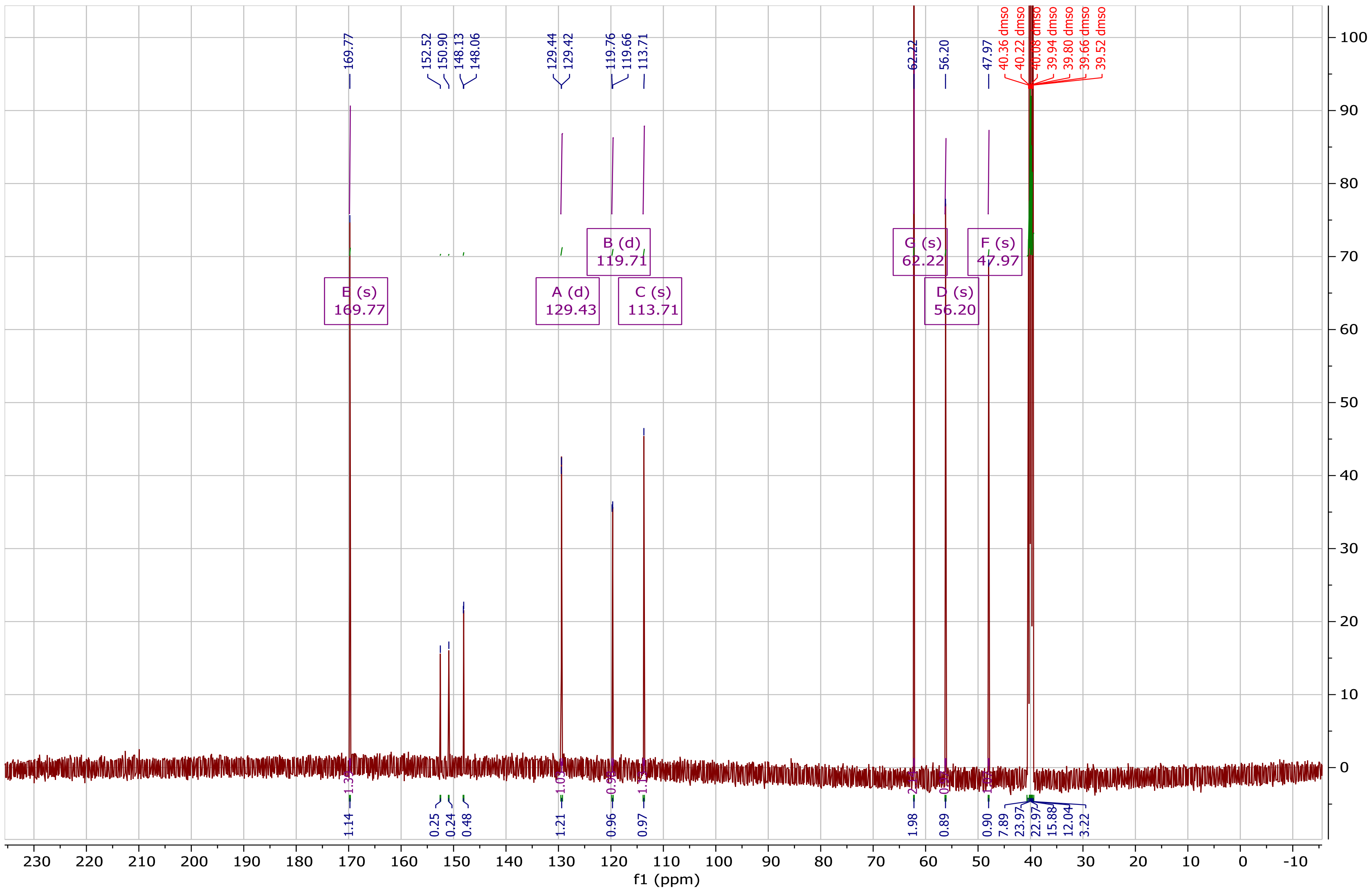

169.77
152.52
150.90
148.13
148.06
129.44
129.42
119.76
119.66
113.71
62.22
56.20
47.97
40.36 dmso
40.22 dmso
40.08 dmso
39.94 dmso
39.80 dmso
39.66 dmso
39.52 dmso
E (s) 169.77
A (d) 129.43
B (d) 119.71
C (s) 113.71
G (s) 62.22
D (s) 56.20
F (s) 47.97
1.14
0.25
0.24
0.48
1.21
0.96
0.97
1.98
0.89
0.90
7.89
23.97
22.97
15.88
12.04
3.22
f1 (ppm)

Single Mass Analysis
Tolerance = 5.0 mDa / DBE: min = -1.5, max = 120.0
Element prediction: Off
Number of isotope peaks used for i-FIT = 3
Monoisotopic Mass, Even Electron Ions
68 formula(e) evaluated with 1 results within limits (up to 20 best isotopic matches for each mass)
Elements Used:

Mass	Calc. Mass	mDa	PPM	DBE	Formula	i-FIT	i-FIT Norm	Fit Conf %	C	H	11B	N	O	F
299.1212	299.1215	-0.3	-1.0	5.5	C12 H17 11B N2 O5 F	179.6	n/a	n/a	12	17	1	2	5	1

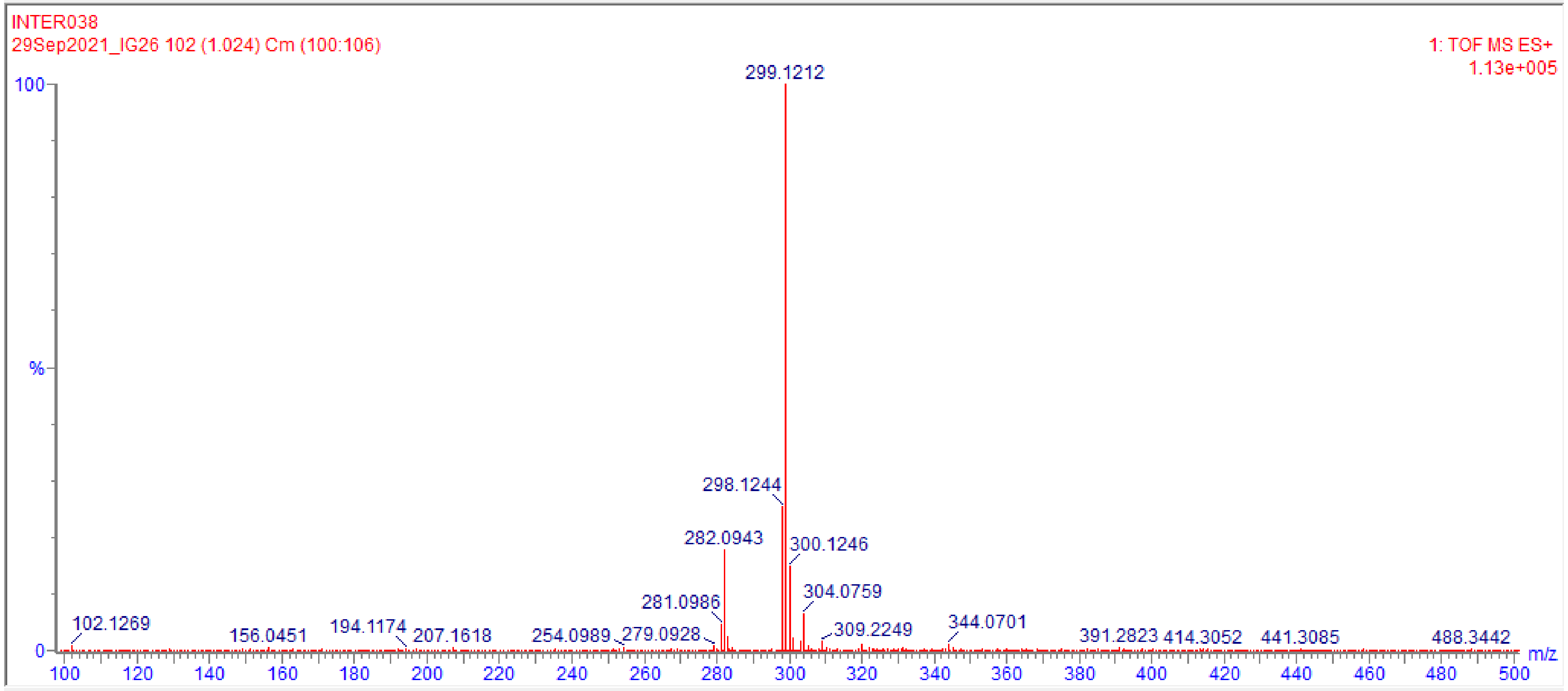

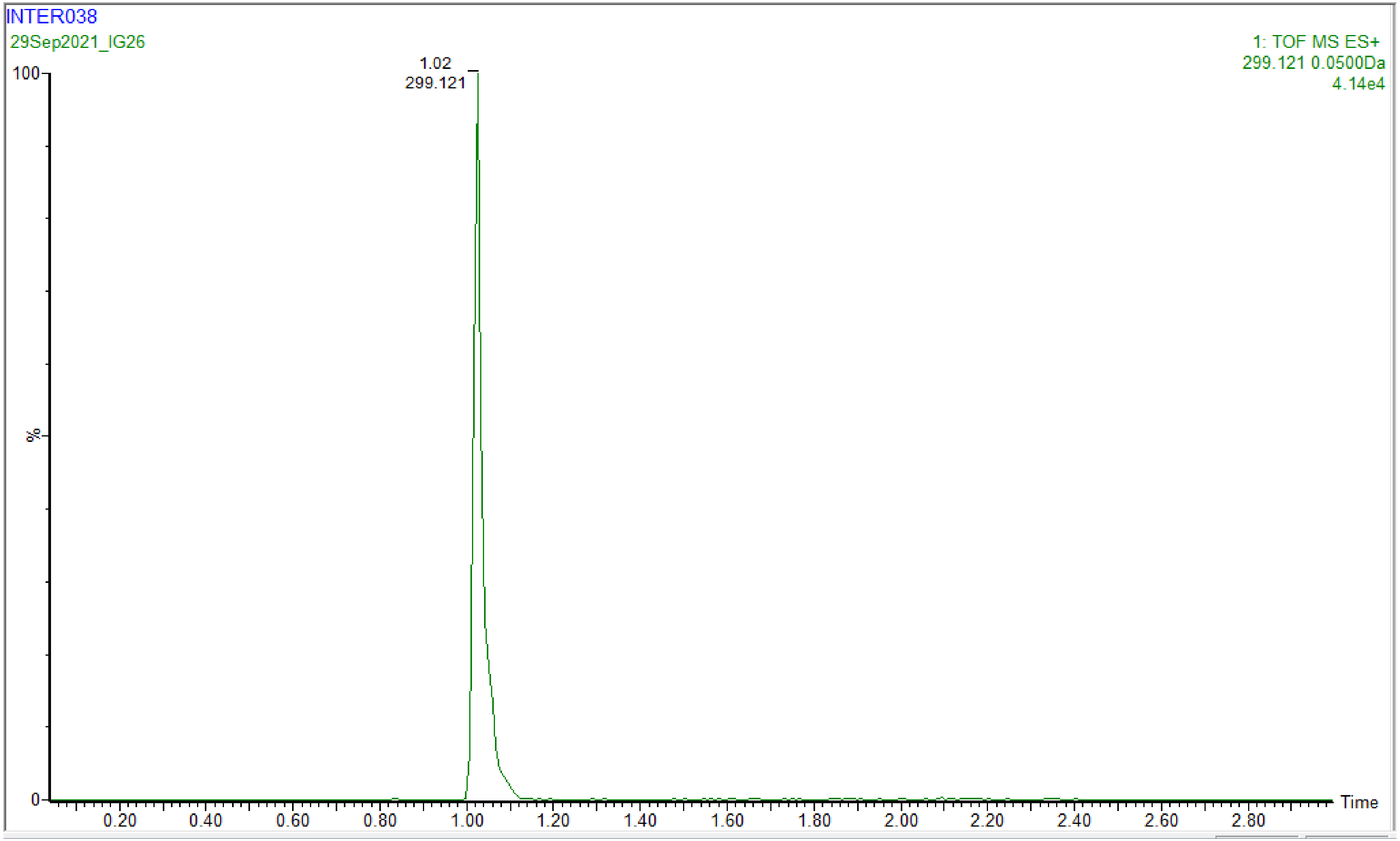
INTER038
29Sep2021_IG26
1: TOF MS ES+
299.121 0.0500Da
4.14e4
1.02
299.121
100
%
0
0.20
0.40
0.60
0.80
1.00
1.20
1.40
1.60
1.80
2.00
2.20
2.40
2.60
2.80
Time

2-(3-Ethoxyphenyl)-6-methyl-1,3,6,2-dioxazaborocane-4,8-dione 8c

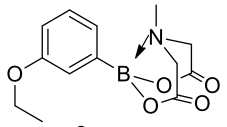

8c

2-(3-ethoxyphenyl)-6-methyl-1,3,6,2-dioxazaborocane-4,8-dione
Chemical Formula: $C_{13}H_{16}BNO_5$
Molecular Weight: 277.0808

Yield = 196.9 mg (71%)

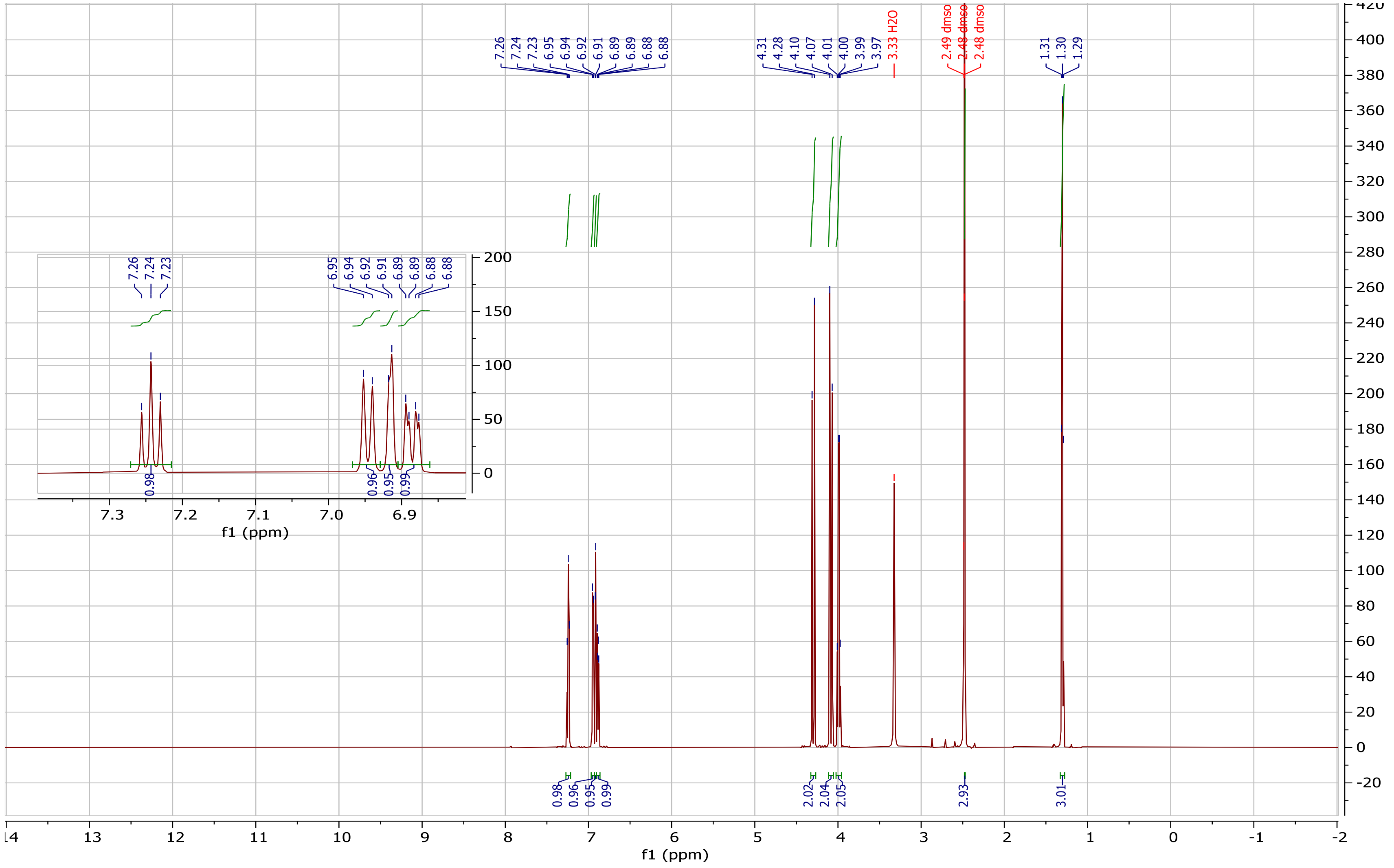
7.26
7.24
7.23
6.95
6.94
6.92
6.91
6.89
6.89
6.88
6.88
4.31
4.28
4.10
4.07
4.01
4.00
3.99
3.97
3.33 H2O
2.49 dmso
2.48 dmso
2.48 dmso
1.31
1.30
1.29
0.98
0.96
0.95
0.99
2.02
2.04
2.05
2.93
3.01
f1 (ppm)
f1 (ppm)

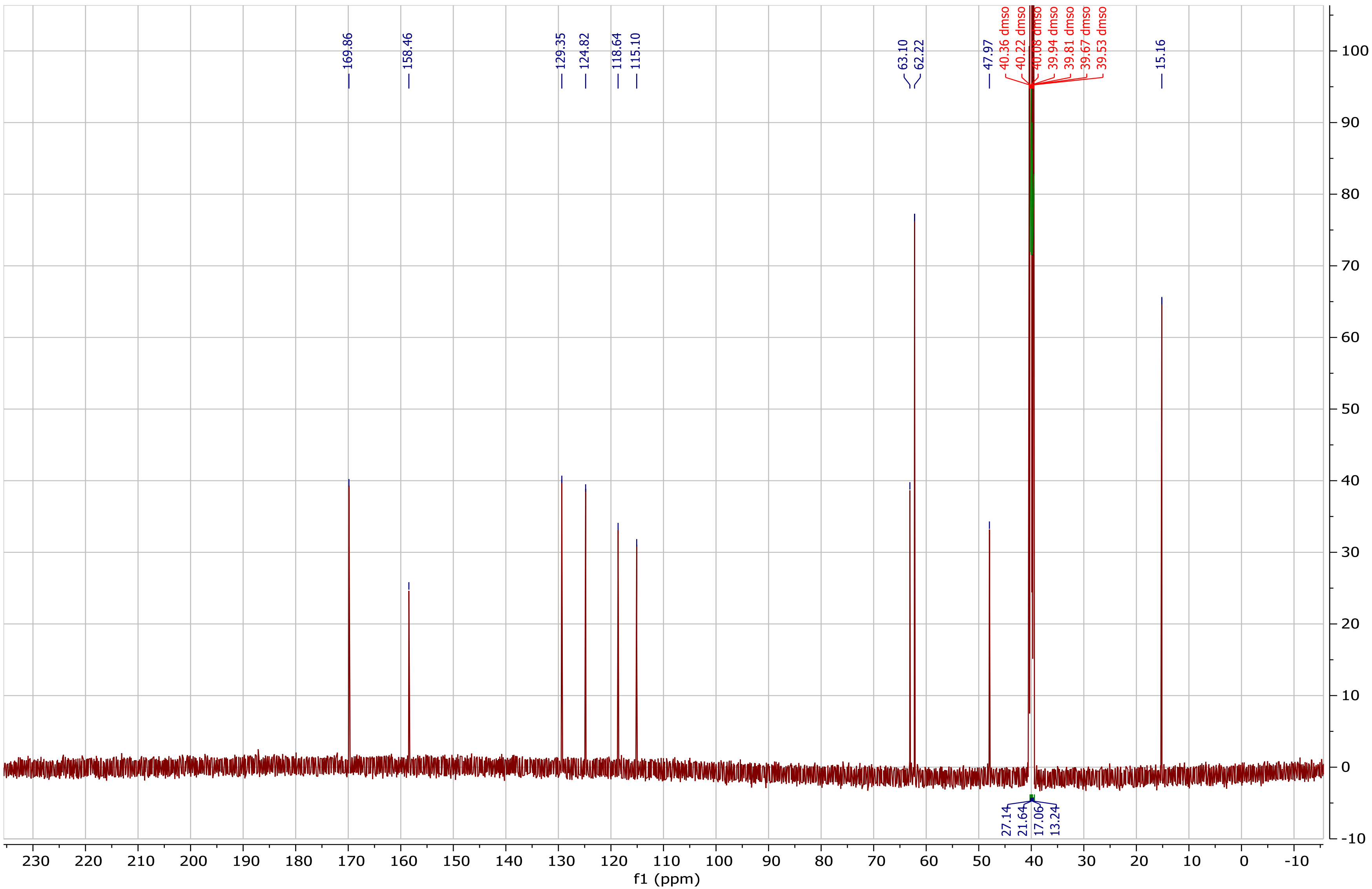
169.86
158.46
129.35
124.82
118.64
115.10
63.10
62.22
47.97
40.36 dmso
40.22 dmso
40.08 dmso
39.94 dmso
39.81 dmso
39.67 dmso
39.53 dmso
15.16
27.14
21.64
17.06
13.24
f1 (ppm)

Single Mass Analysis
Tolerance = 5.0 mDa / DBE: min = -1.5, max = 120.0
Element prediction: Off
Number of isotope peaks used for i-FIT = 3
Monoisotopic Mass, Even Electron Ions
32 formula(e) evaluated with 1 results within limits (up to 20 best isotopic matches for each mass)
Elements Used:

Mass	Calc. Mass	mDa	PPM	DBE	Formula	i-FIT	i-FIT Norm	Fit Conf %	C	H	11B	N	O
278.1200	278.1200	0.0	0.0	6.5	C13 H17 11B N O5	167.5	n/a	n/a	13	17	1	1	5

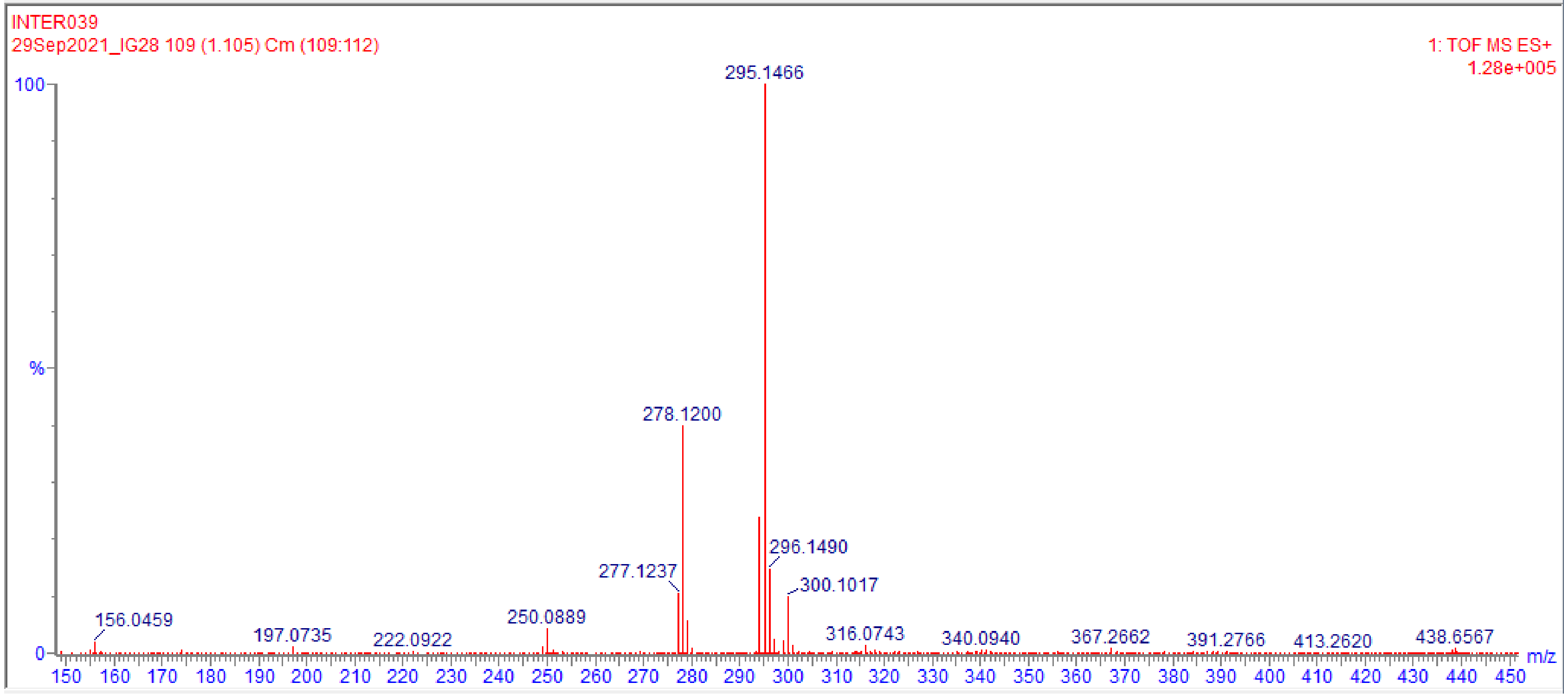

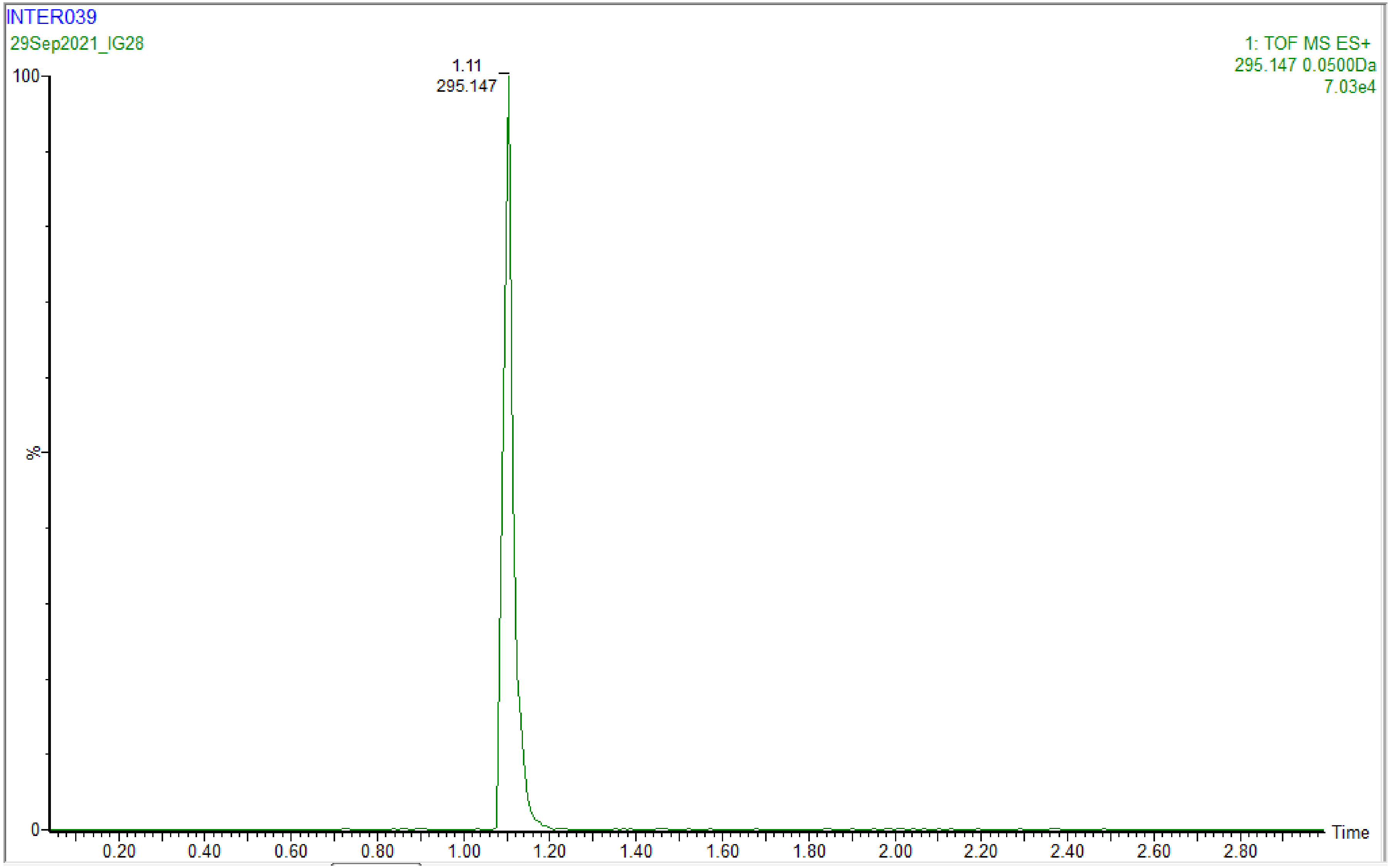

INTER039
29Sep2021_IG28
1: TOF MS ES+
295.147 0.0500Da
7.03e4
1.11
295.147
100
%
0
0.20
0.40
0.60
0.80
1.00
1.20
1.40
1.60
1.80
2.00
2.20
2.40
2.60
2.80
Time

Methyl 4-(6-methyl-4,8-dioxo-1,3,6,2-dioxazaborocan-2-yl)benzoate

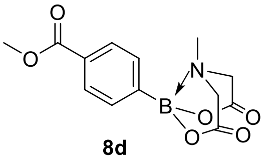

8d

methyl 4-(6-methyl-4,8-dioxo-1,3,6,2-dioxazaborocan-2-yl)benzoate
Chemical Formula: $C_{13}H_{14}BNO_6$
Molecular Weight: 291.0644

Yield = 221.1 mg (76%)

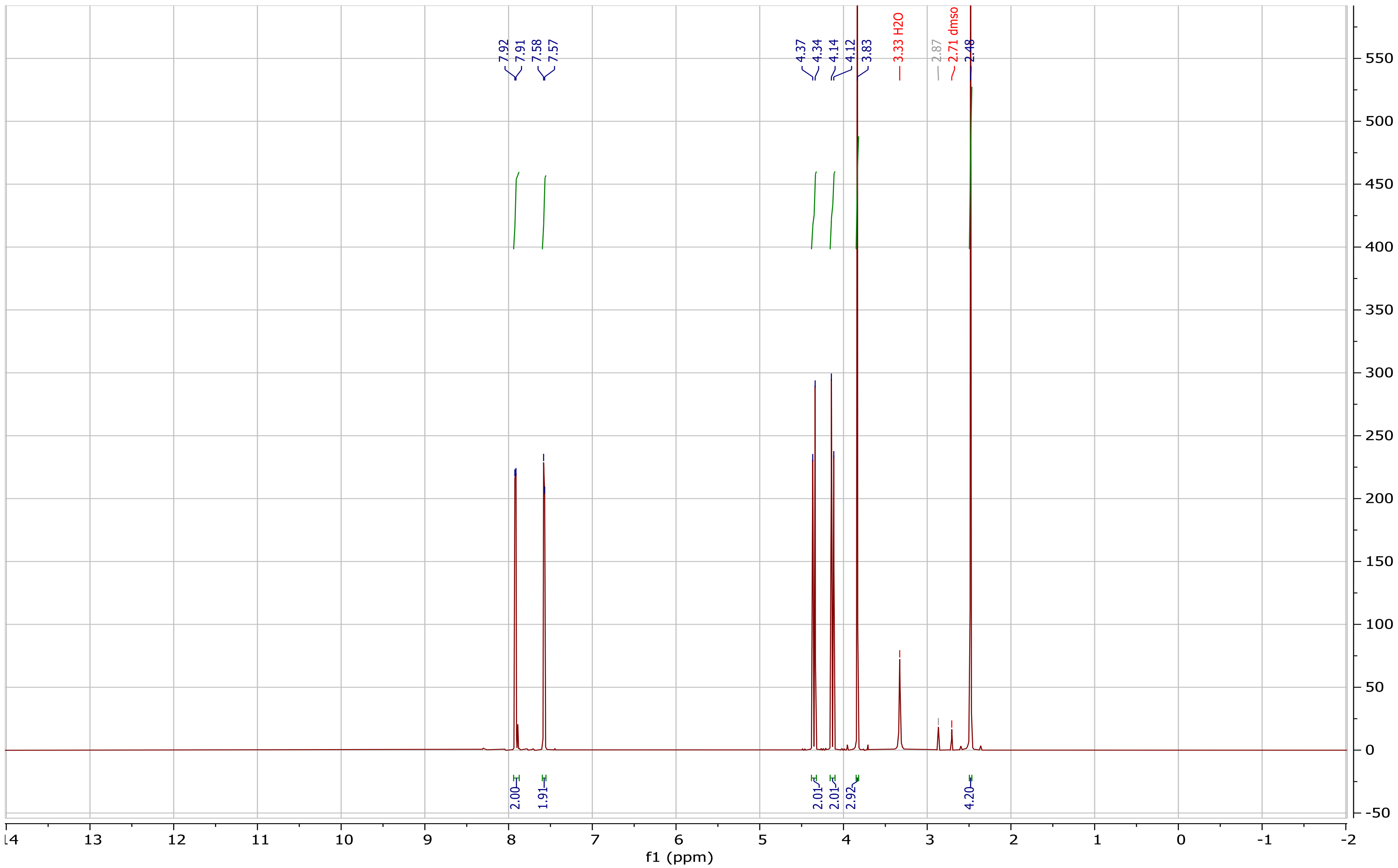

7.92
7.91
7.58
7.57
4.37
4.34
4.14
4.12
3.83
3.33 H2O
2.87
2.71 dmso
2.48
2.00
1.91
2.01
2.01
2.92
4.20
f1 (ppm)

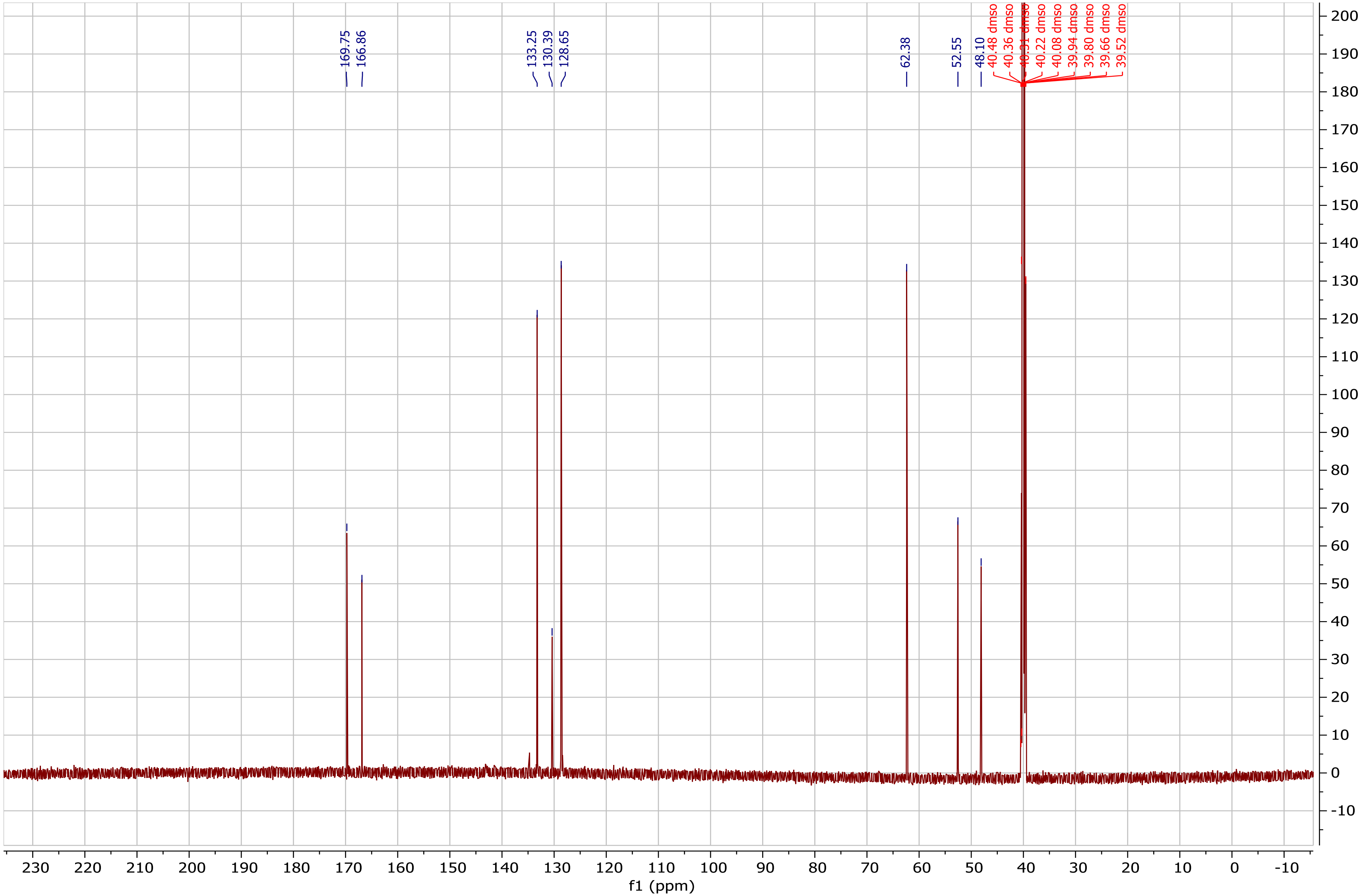

169.75
166.86
133.25
130.39
128.65
62.38
52.55
48.10
40.48 dmso
40.36 dmso
40.31 dmso
40.22 dmso
40.08 dmso
39.94 dmso
39.80 dmso
39.66 dmso
39.52 dmso
f1 (ppm)

Single Mass Analysis
Tolerance = 5.0 mDa / DBE: min = -1.5, max = 120.0
Element prediction: Off
Number of isotope peaks used for i-FIT = 3
Monoisotopic Mass, Even Electron Ions
39 formula(e) evaluated with 1 results within limits (up to 20 best isotopic matches for each mass)
Elements Used:

Mass	Calc. Mass	mDa	PPM	DBE	Formula	i-FIT	i-FIT Norm	Fit Conf %	C	H	11B	N	O
309.1262	309.1258	0.4	1.3	6.5	C13 H18 11B N2 O6	171.5	n/a	n/a	13	18	1	2	6

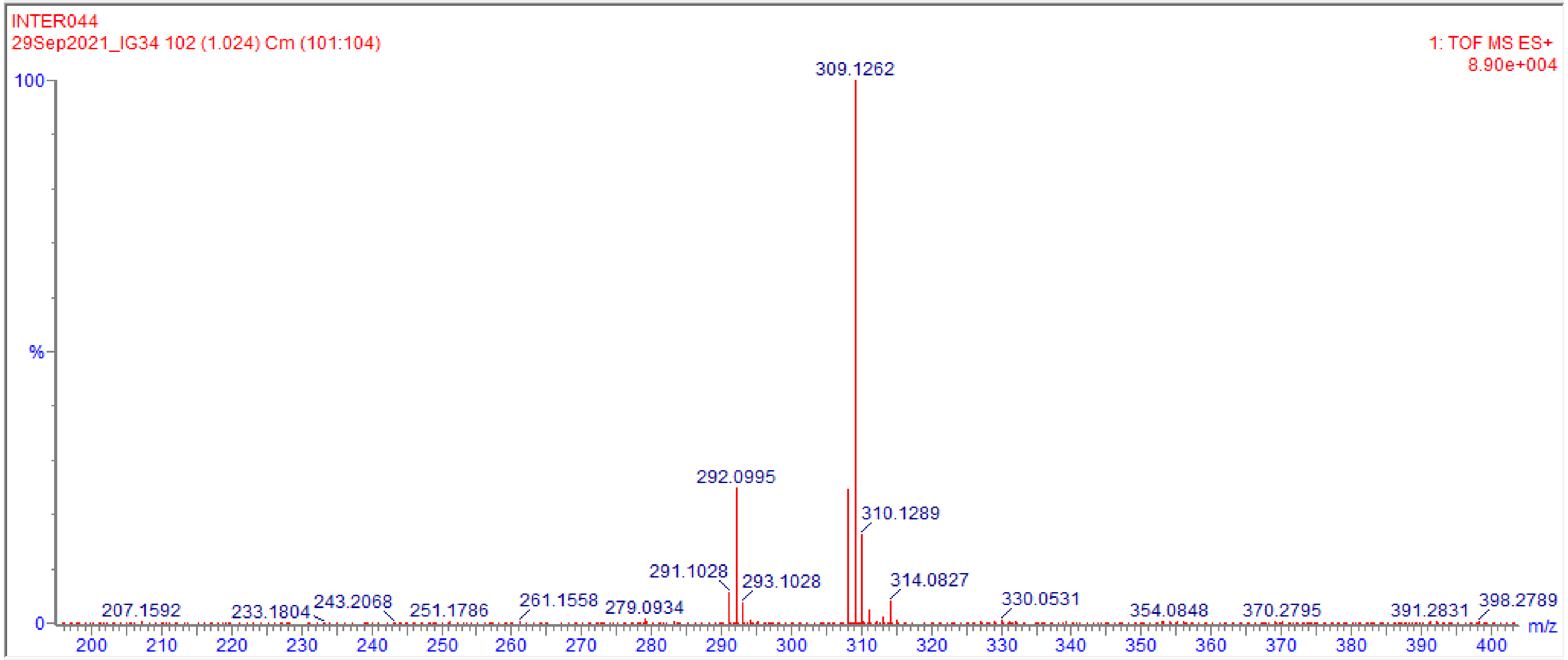

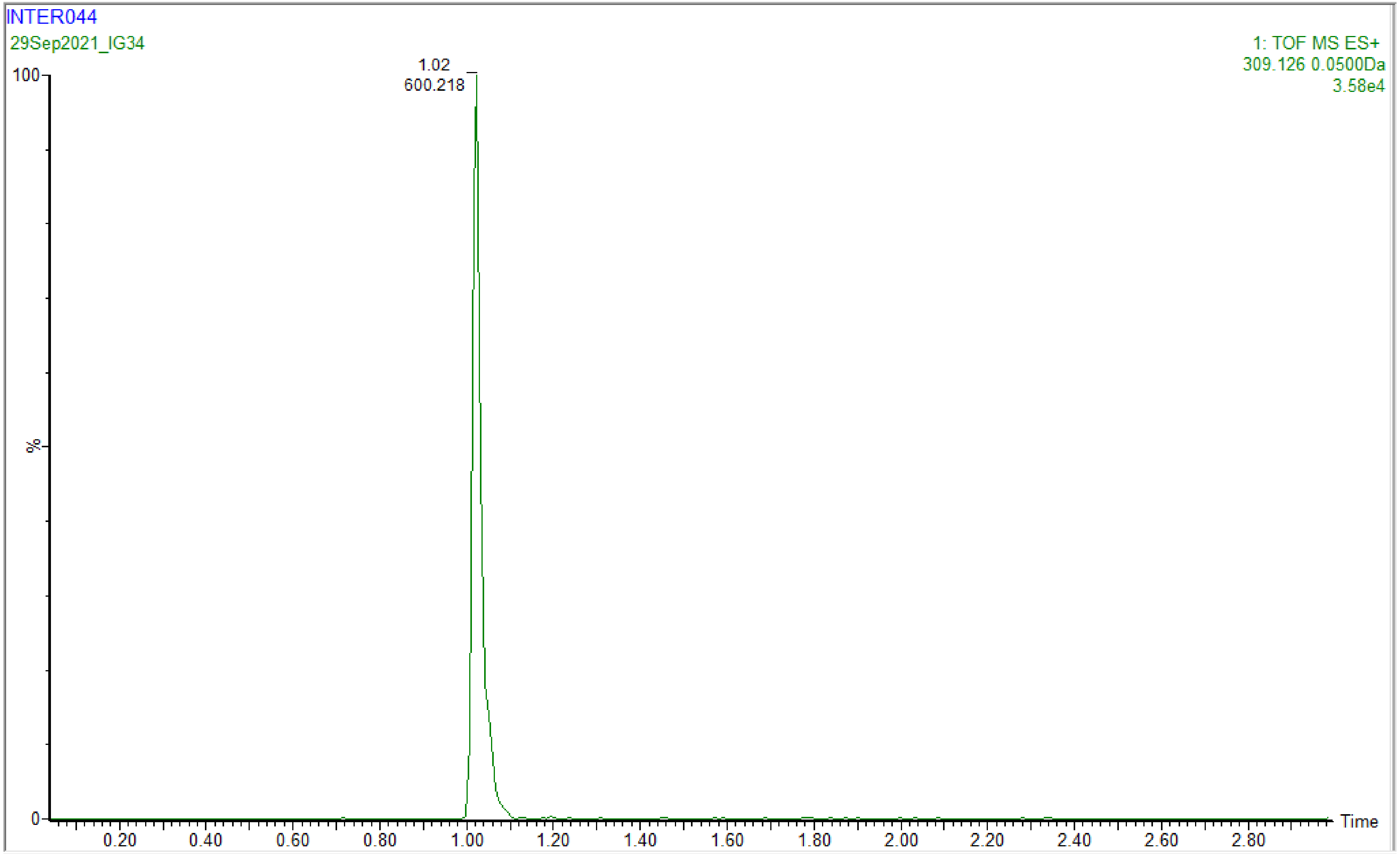

INTER044
29Sep2021_IG34
1: TOF MS ES+
309.126 0.0500Da
3.58e4
1.02
600.218
100
%
0
0.20
0.40
0.60
0.80
1.00
1.20
1.40
1.60
1.80
2.00
2.20
2.40
2.60
2.80
Time

6-Methyl-2-(2-(trifluoromethyl)phenyl)-1,3,6,2-dioxazaborocane-4,8-dione 8e

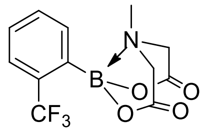

8e

6-methyl-2-(2-(trifluoromethyl)phenyl)-1,3,6,2-dioxazaborocane-4,8-dione
Chemical Formula: $C_{12}H_{11}BF_3NO_4$
Molecular Weight: 301.0262

Yield = 114.3 mg (38%).

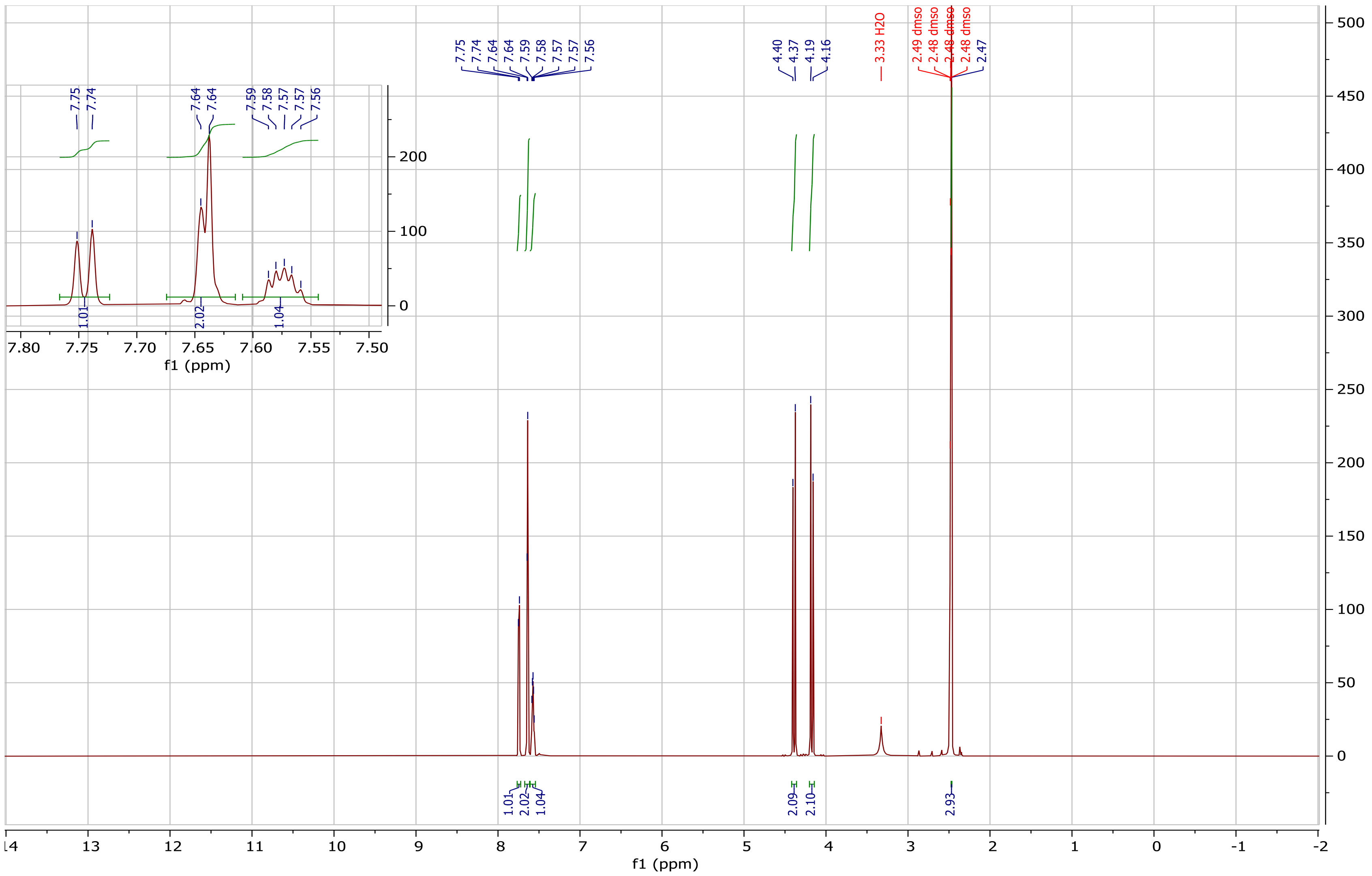

7.75
7.74
7.64
7.64
7.59
7.58
7.57
7.57
7.56
4.40
4.37
4.19
4.16
3.33 H2O
2.49 dmso
2.48 dmso
2.48 dmso
2.48 dmso
2.47
1.01
2.02
1.04
2.09
2.10
2.93
f1 (ppm)
f1 (ppm)

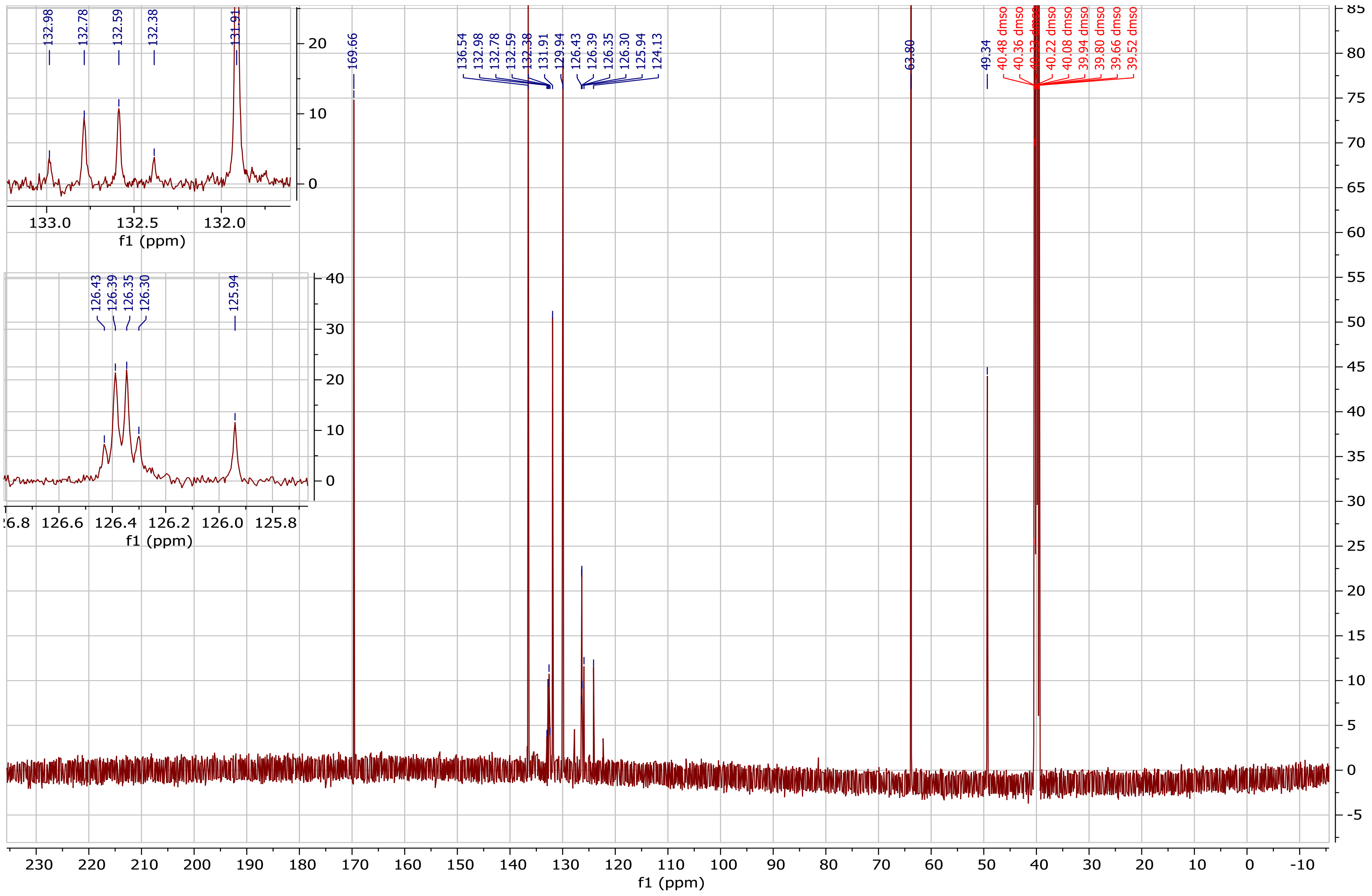
169.66
136.54
132.98
132.78
132.59
132.38
131.91
129.94
126.43
126.39
126.35
126.30
125.94
124.13
63.80
49.34
40.48 dmso
40.36 dmso
40.22 dmso
40.08 dmso
39.94 dmso
39.80 dmso
39.66 dmso
39.52 dmso
f1 (ppm)

Single Mass Analysis

Tolerance = 5.0 mDa / DBE: min = -1.5, max = 120.0

Element prediction: Off

Number of isotope peaks used for i-FIT = 3

Monoisotopic Mass, Even Electron Ions

111 formula(e) evaluated with 1 results within limits (up to 20 best isotopic matches for each mass)

Elements Used:

Mass	Calc. Mass	mDa	PPM	DBE	Formula	i-FIT	i-FIT Norm	Fit Conf %	C	H	11B	N	O	F
302.0811	302.0811	0.0	0.0	6.5	C12 H12 11B N O4 F3	248.6	n/a	n/a	12	12	1	1	4	3

INTER046

29Sep2021_IG36 110 (1.114) Cm (110:113)

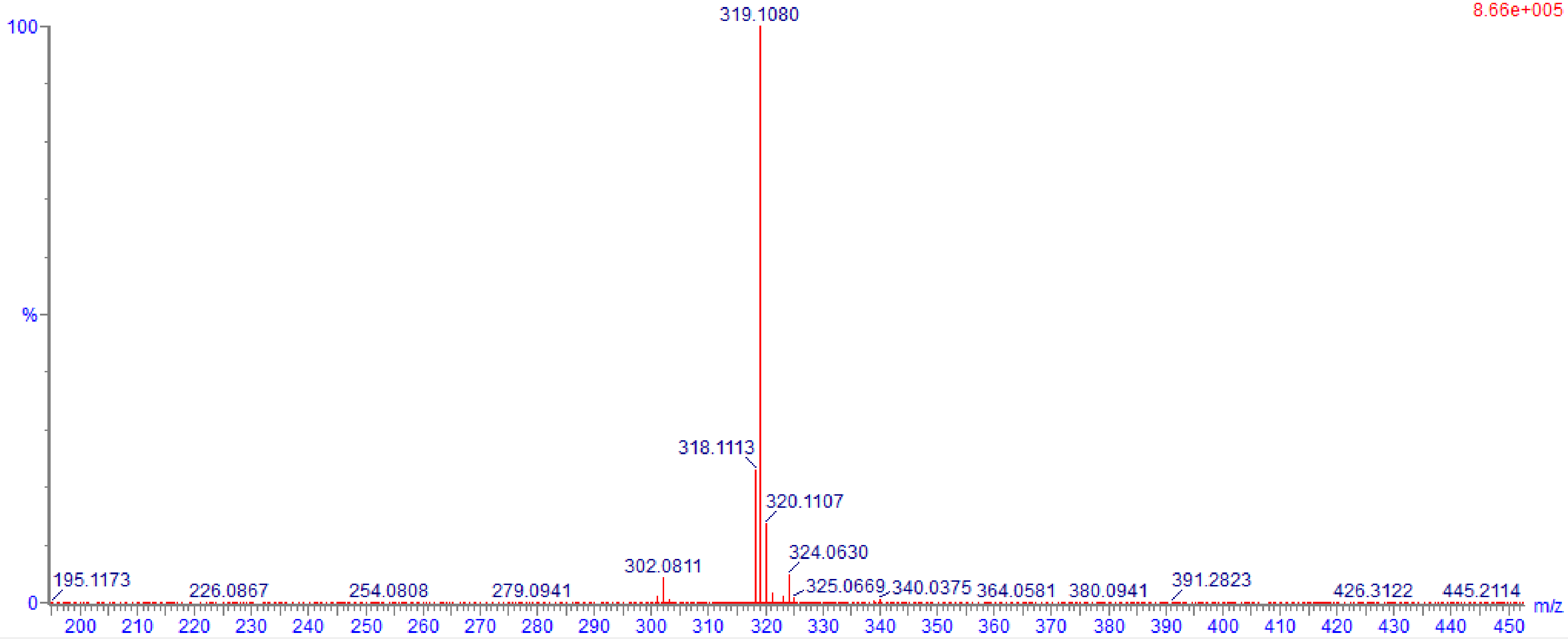

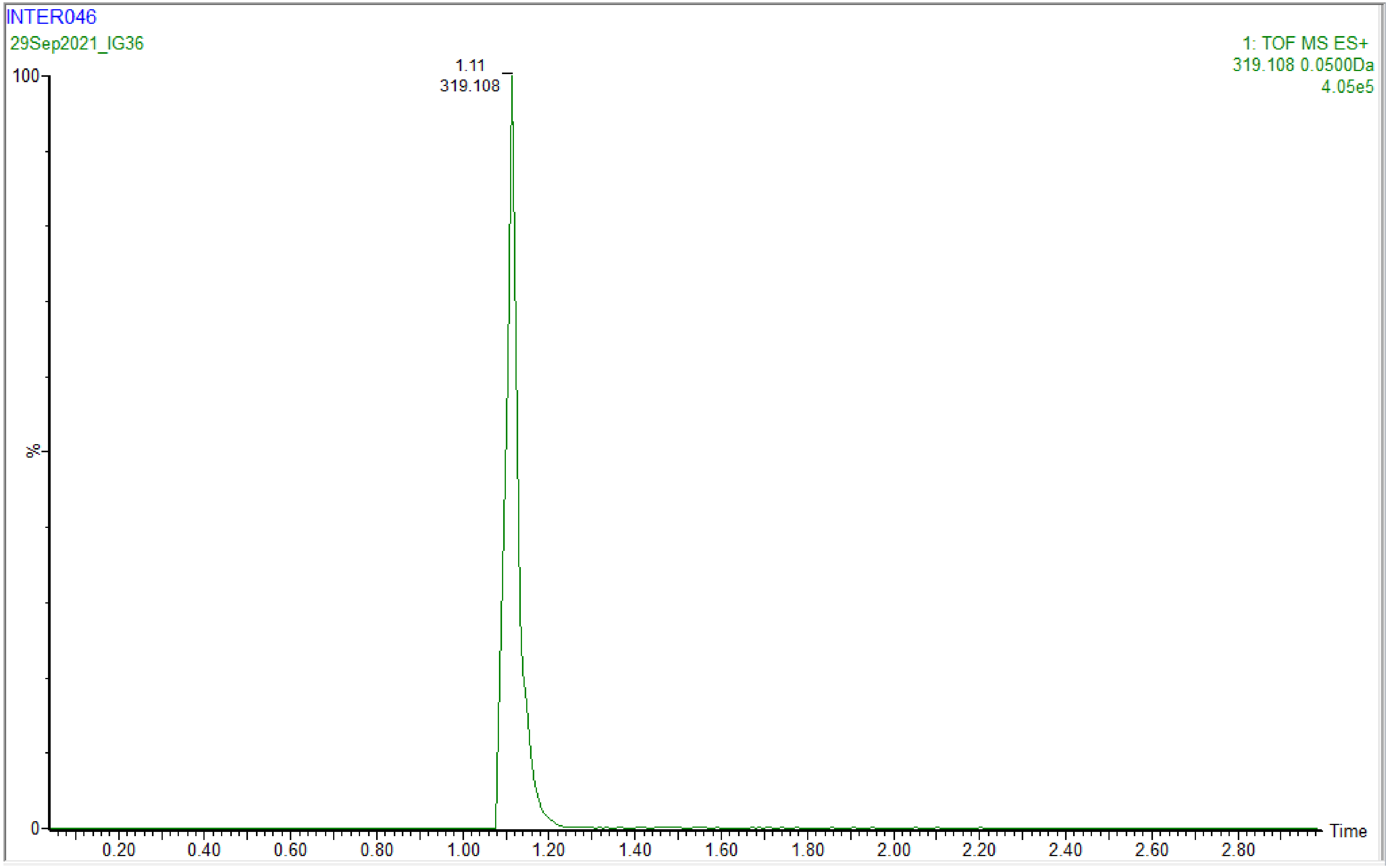
INTER046
29Sep2021_IG36
1: TOF MS ES+
319.108 0.0500Da
4.05e5
1.11
319.108
100
%
0
0.20
0.40
0.60
0.80
1.00
1.20
1.40
1.60
1.80
2.00
2.20
2.40
2.60
2.80
Time

2-Fluoro-4-(6-methyl-4,8-dioxo-1,3,6,2-dioxazaborocan-2-yl)benzonitrile 8f

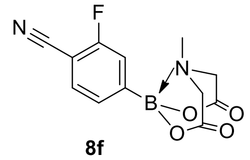

8f

2-fluoro-4-(6-methyl-4,8-dioxo-1,3,6,2-dioxazaborocan-2-yl)benzonitrile
Chemical Formula: $C_{12}H_{10}BFN_2O_4$
Molecular Weight: 276.0282

Yield = 198.0 mg (72%).

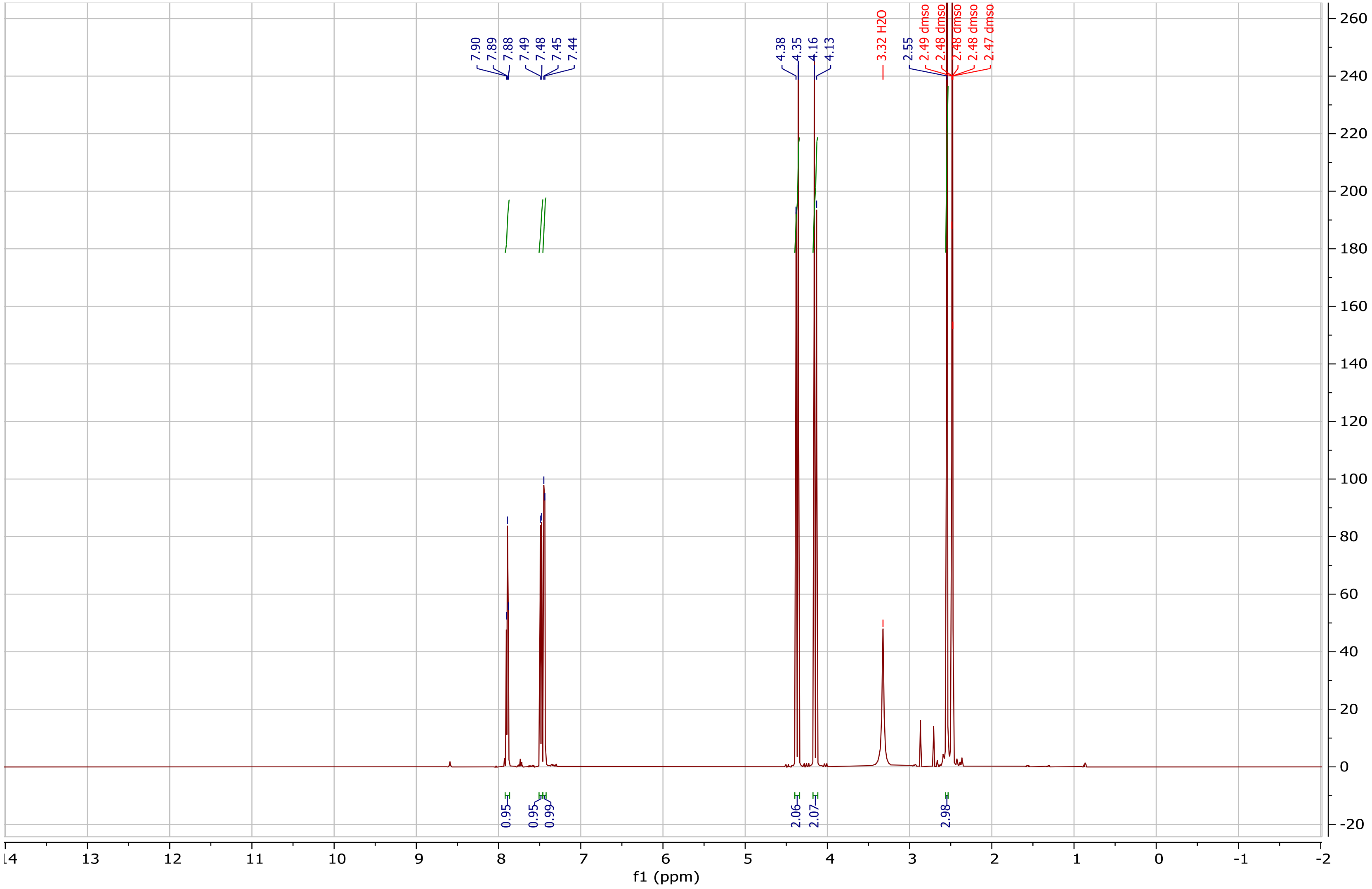

7.90
7.89
7.88
7.49
7.48
7.45
7.44
4.38
4.35
4.16
4.13
3.32 H2O
2.55
2.49 dmso
2.48 dmso
2.48 dmso
2.48 dmso
2.47 dmso
0.95
0.95
0.99
2.06
2.07
2.98
f1 (ppm)

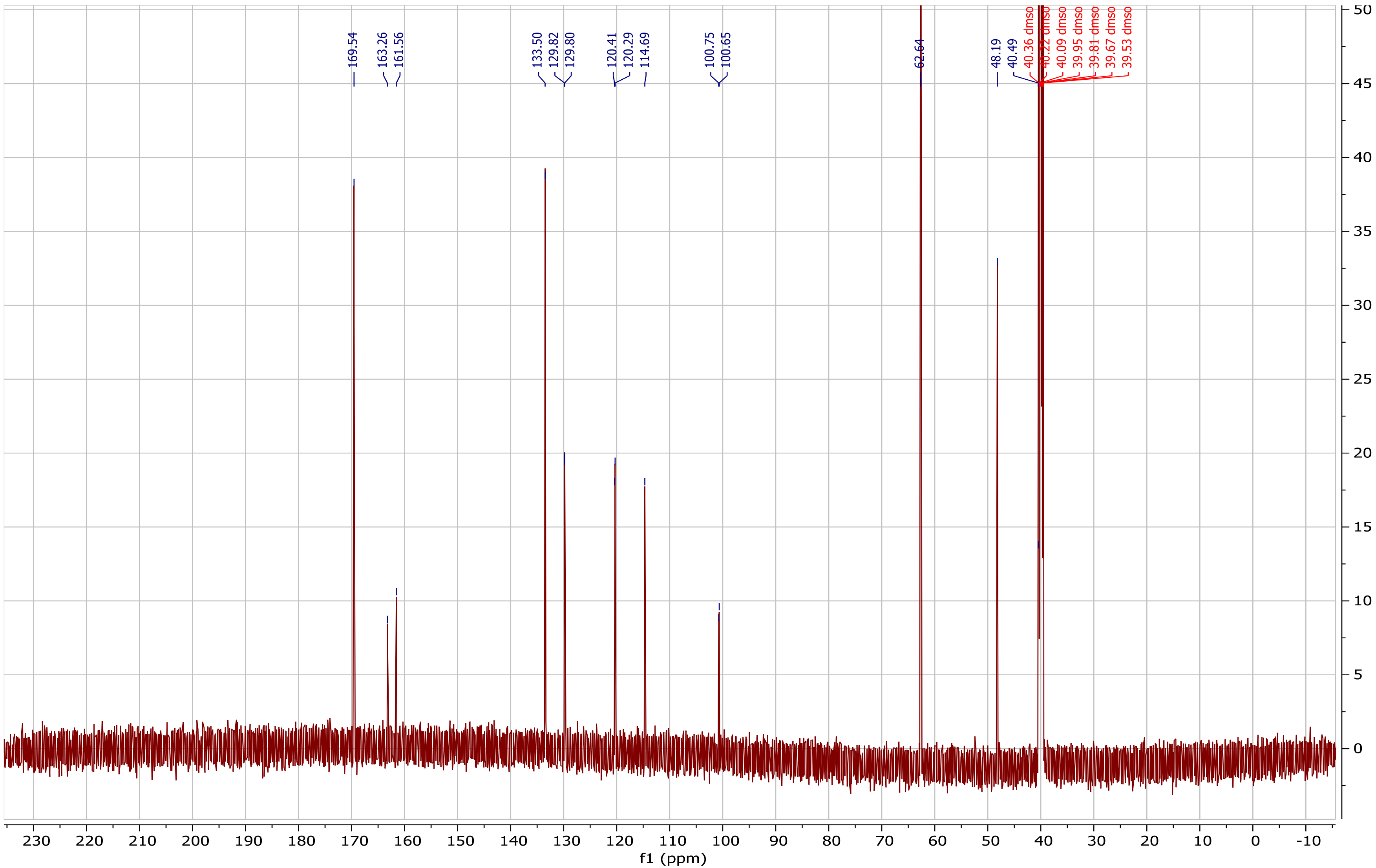
169.54
163.26
161.56
133.50
129.82
129.80
120.41
120.29
114.69
100.75
100.65
62.64
48.19
40.49
40.36 dmso
40.22 dmso
40.09 dmso
39.95 dmso
39.81 dmso
39.67 dmso
39.53 dmso
230 220 210 200 190 180 170 160 150 140 130 120 110 100 90 80 70 60 50 40 30 20 10 0 -10
f1 (ppm)
50 45 40 35 30 25 20 15 10 5 0

Single Mass Analysis
Tolerance = 5.0 mDa / DBE: min = -1.5, max = 120.0
Element prediction: Off
Number of isotope peaks used for i-FIT = 3
Monoisotopic Mass, Even Electron Ions
76 formula(e) evaluated with 1 results within limits (up to 20 best isotopic matches for each mass)
Elements Used:

Mass	Calc. Mass	mDa	PPM	DBE	Formula	i-FIT	i-FIT Norm	Fit Conf %	C	H	11B	N	O	F
294.1063	294.1061	0.2	0.7	7.5	C12 H14 11B N3 O4 F	91.4	n/a	n/a	12	14	1	3	4	1

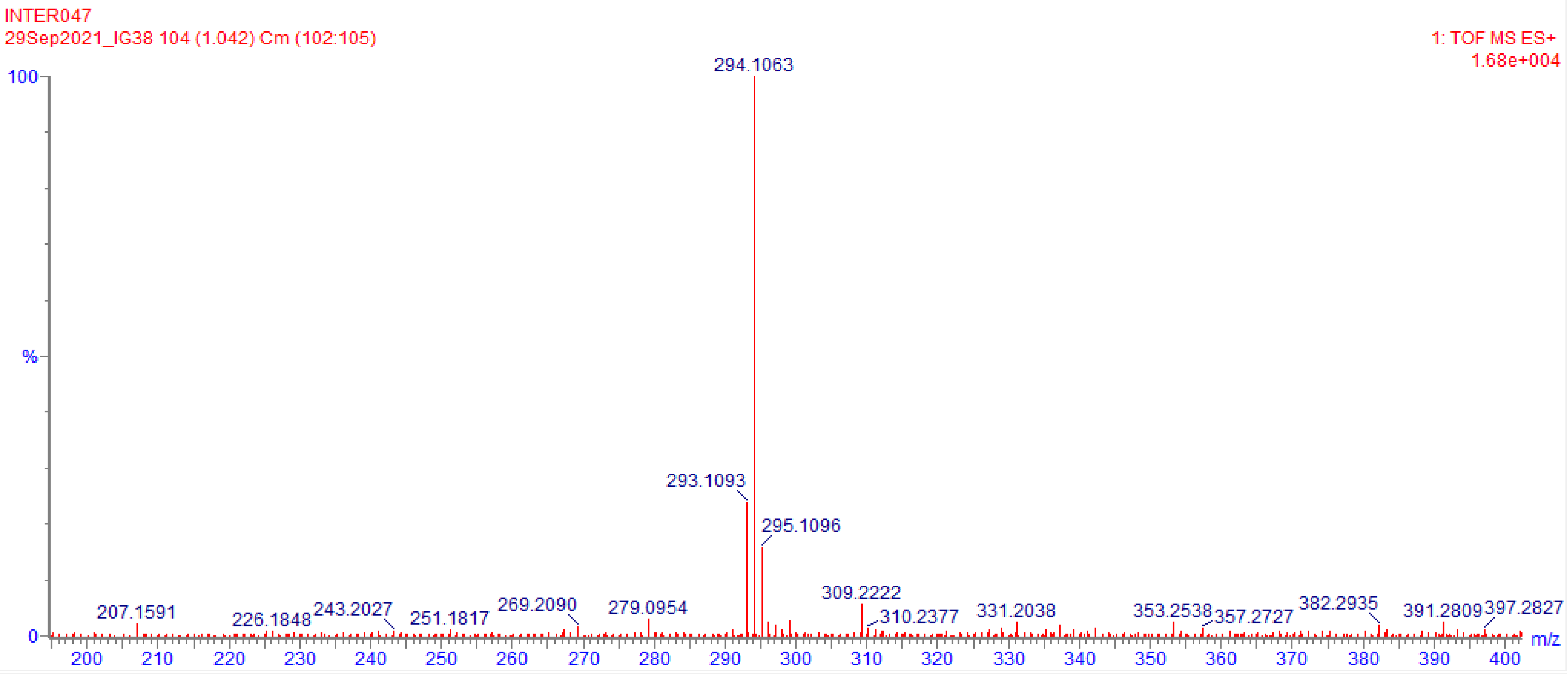

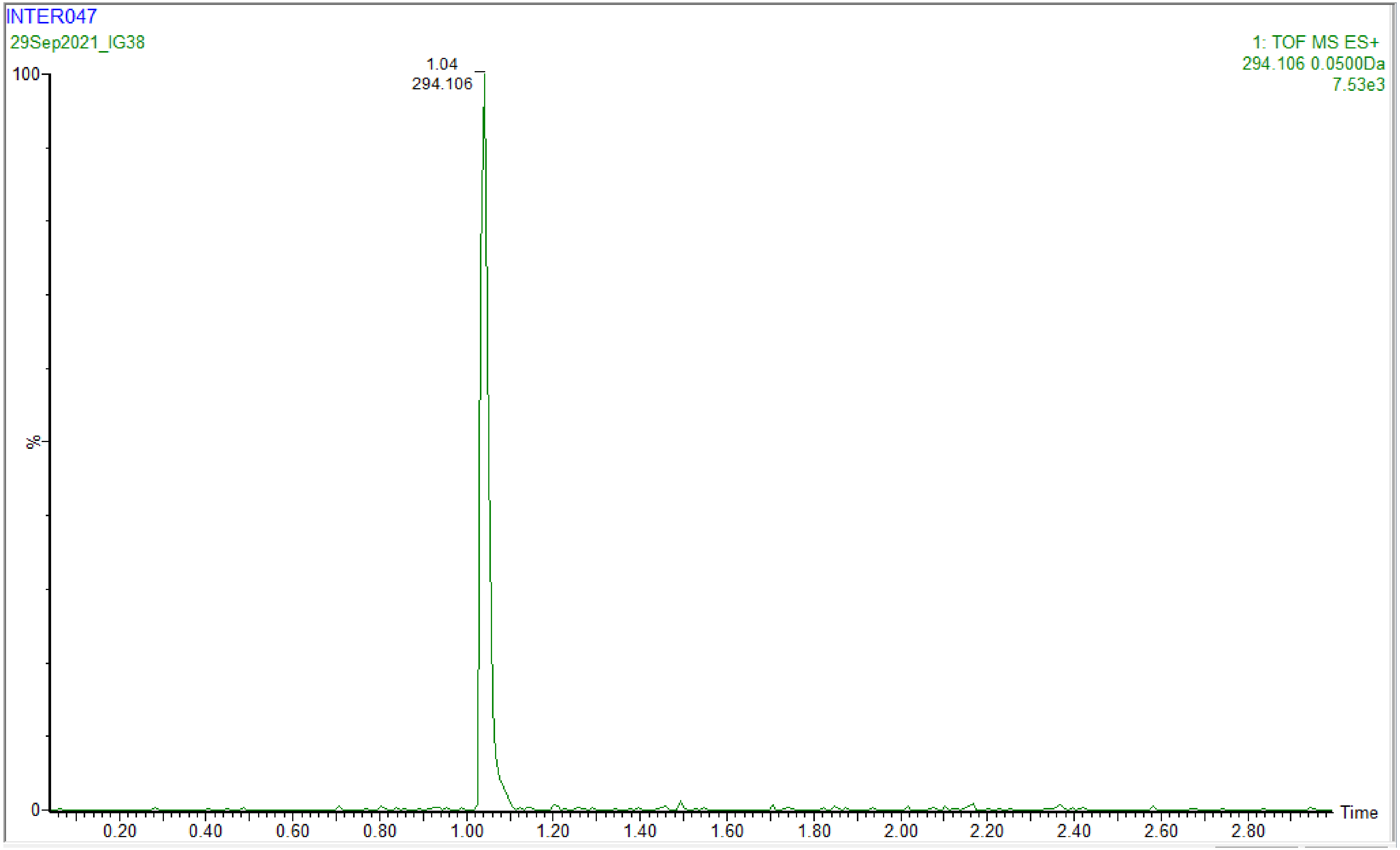
INTER047
29Sep2021_IG38
1: TOF MS ES+
294.106 0.0500Da
7.53e3
1.04
294.106
100
%
0
0.20
0.40
0.60
0.80
1.00
1.20
1.40
1.60
1.80
2.00
2.20
2.40
2.60
2.80
Time

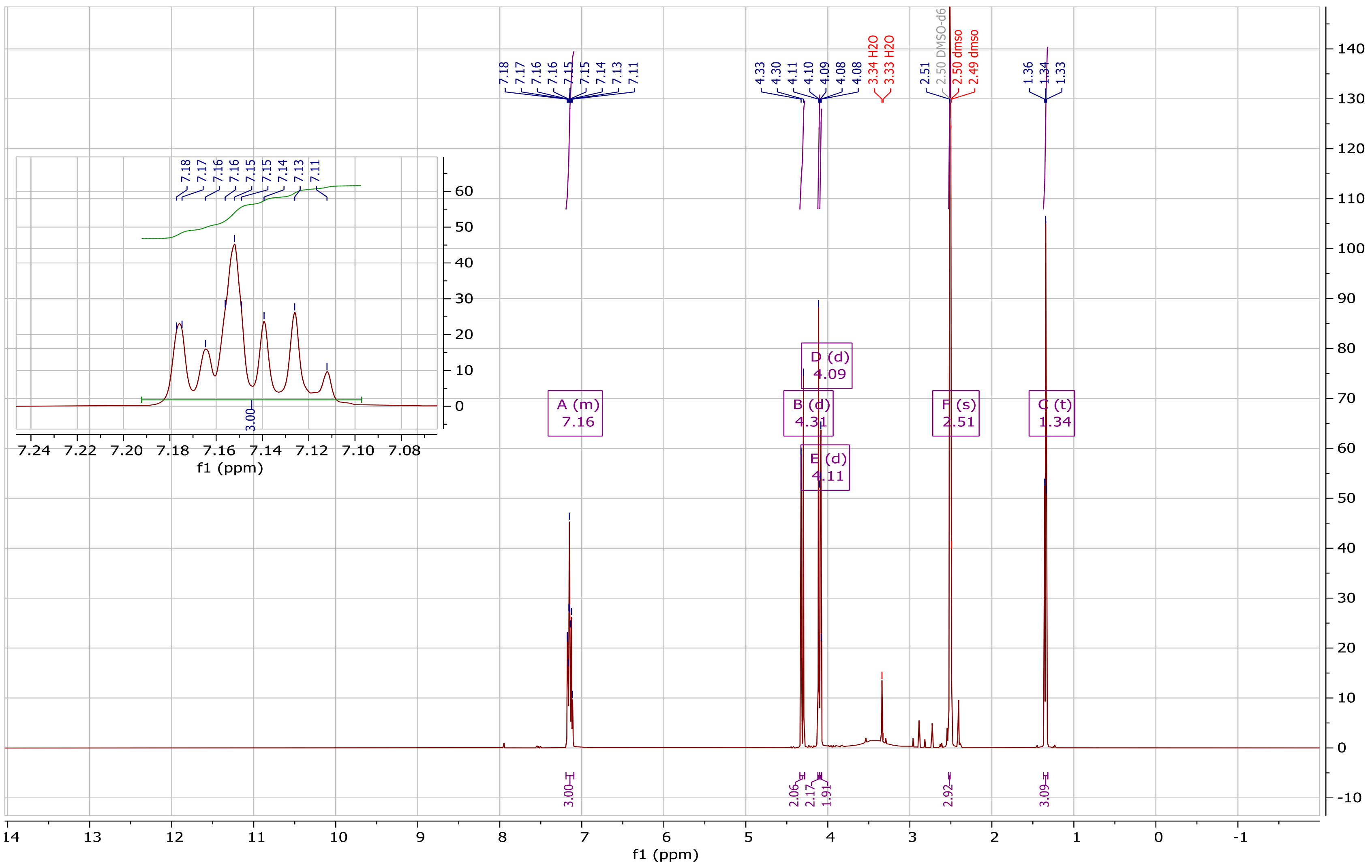
7.18
7.17
7.16
7.16
7.15
7.15
7.14
7.13
7.11
4.33
4.30
4.11
4.10
4.09
4.08
4.08
3.34 H2O
3.33 H2O
2.51
2.50 DMSO-d6
2.50 dmso
2.49 dmso
1.36
1.34
1.33
A (m)
7.16
B (d)
4.31
D (d)
4.09
E (d)
4.11
F (s)
2.51
C (t)
1.34
3.00
2.06
2.17
1.91
2.92
3.09
f1 (ppm)
f1 (ppm)

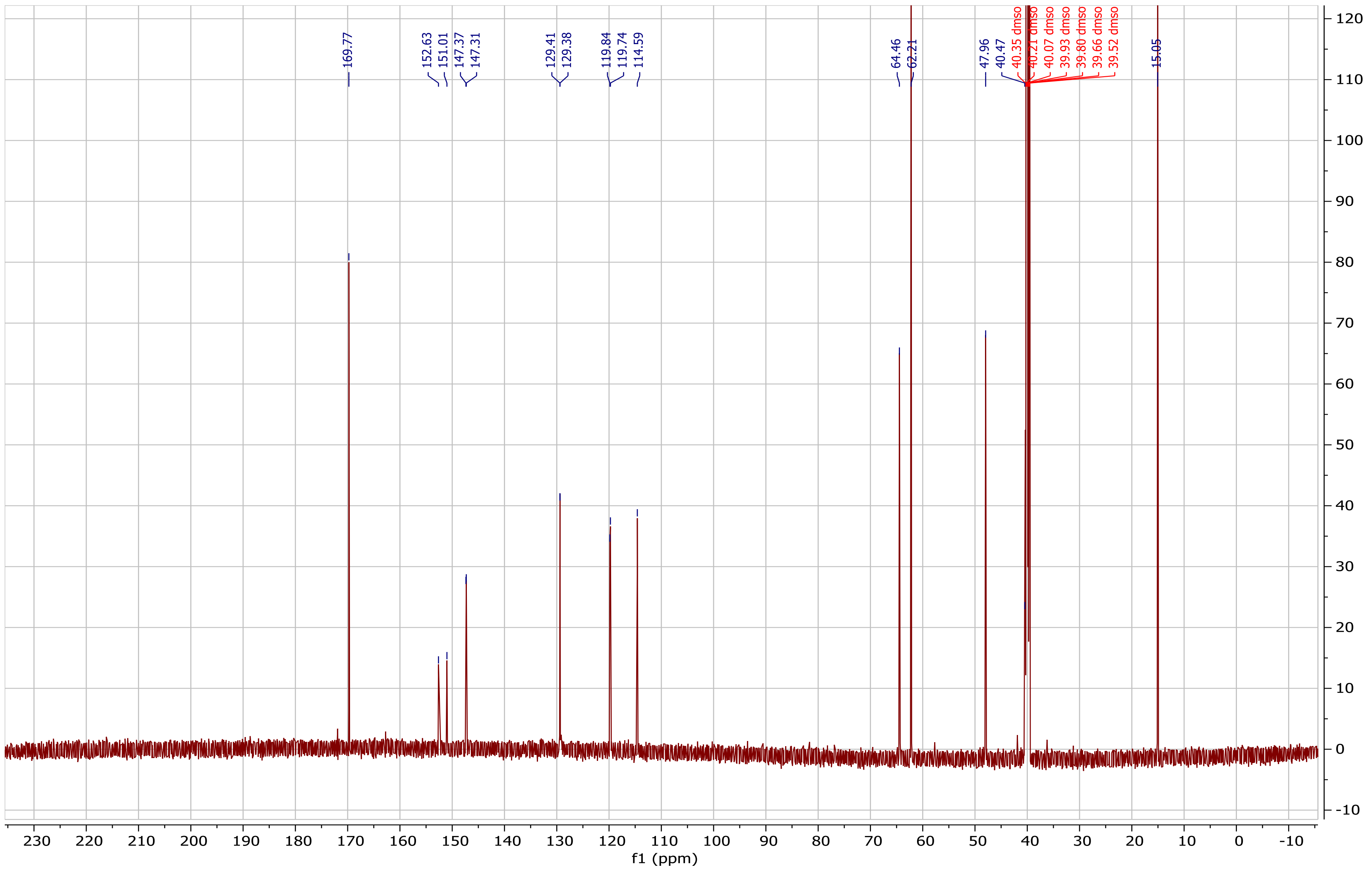

169.77
152.63
151.01
147.37
147.31
129.41
129.38
119.84
119.74
114.59
64.46
62.21
47.96
40.47
40.35 dmso
40.21 dmso
40.07 dmso
39.93 dmso
39.80 dmso
39.66 dmso
39.52 dmso
15.05
f1 (ppm)

Single Mass Analysis
Tolerance = 5.0 mDa / DBE: min = -1.5, max = 120.0
Element prediction: Off
Number of isotope peaks used for i-FIT = 3
Monoisotopic Mass, Even Electron Ions
64 formula(e) evaluated with 1 results within limits (up to 20 best isotopic matches for each mass)
Elements Used:

Mass	Calc. Mass	mDa	PPM	DBE	Formula	i-FIT	i-FIT Norm	Fit Conf %	C	H	11B	N	O	F
296.1112	296.1106	0.6	2.0	6.5	C13 H16 11B N O5 F	168.0	n/a	n/a	13	16	1	1	5	1

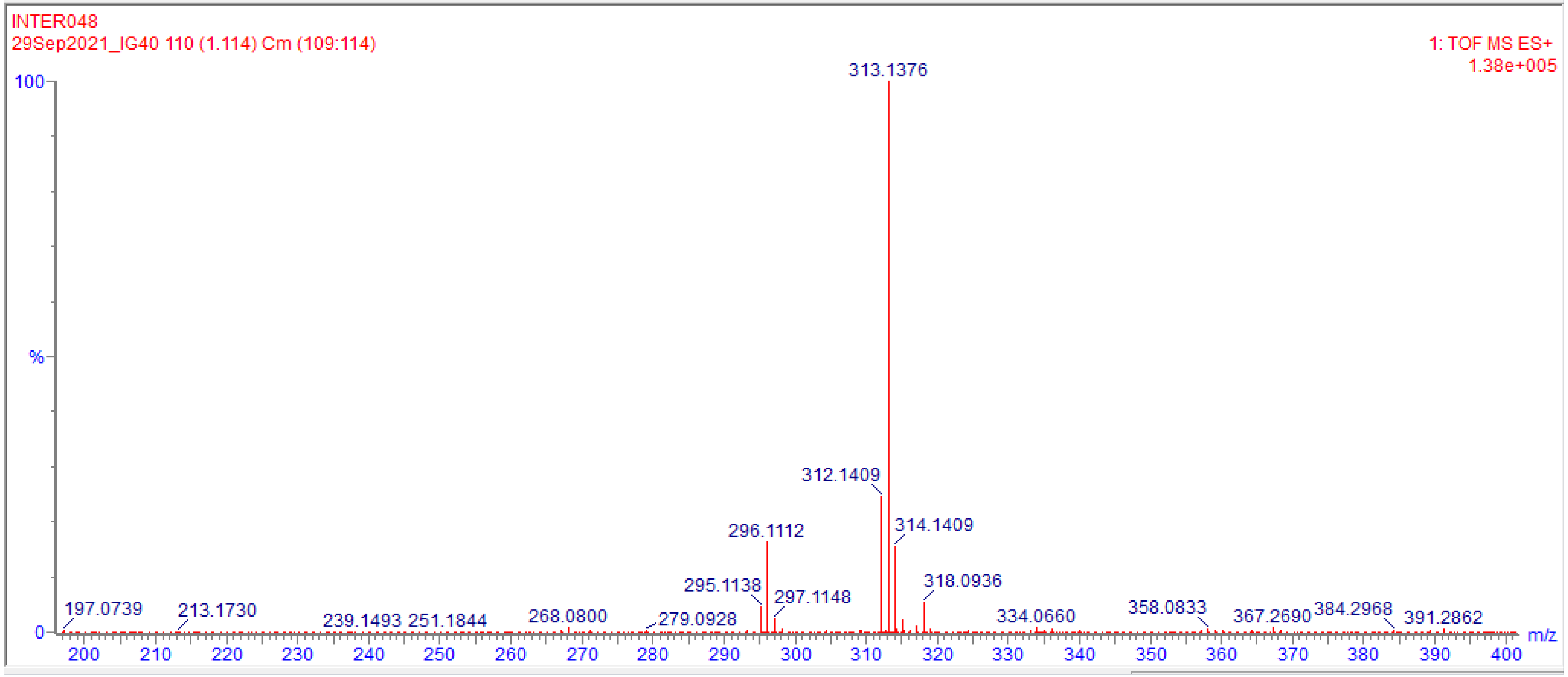

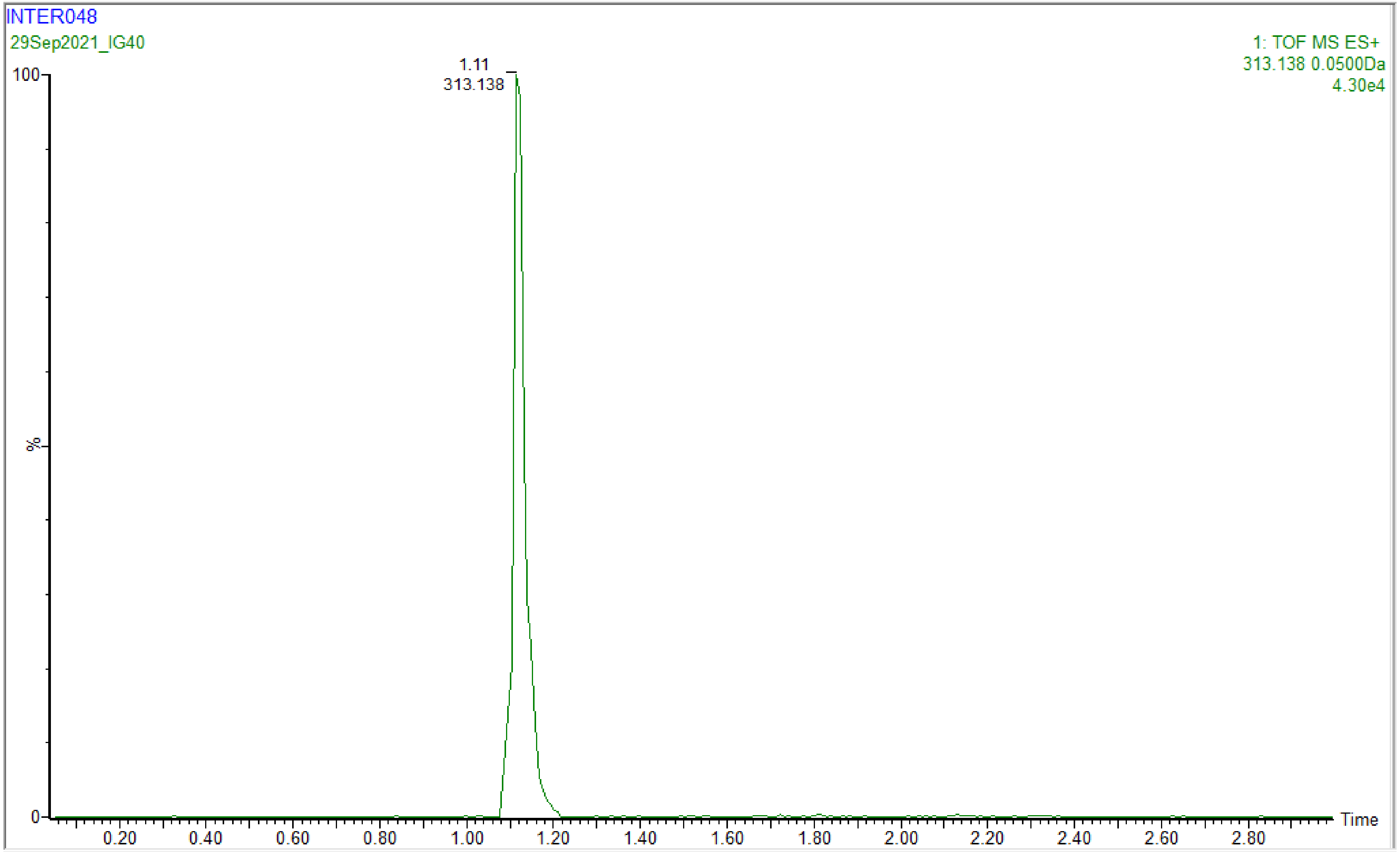
INTER048
29Sep2021_IG40
1: TOF MS ES+
313.138 0.0500Da
4.30e4
1.11
313.138
100
%
0
0.20
0.40
0.60
0.80
1.00
1.20
1.40
1.60
1.80
2.00
2.20
2.40
2.60
2.80
Time

2-(2-Chloro-4-(trifluoromethyl)phenyl)-6-methyl-1,3,6,2-dioxazaborocane-4,8-dione 8h

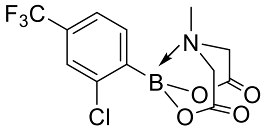

8h

2-(2-chloro-4-(trifluoromethyl)phenyl)-6-methyl-1,3,6,2-dioxazaborocane-4,8-dione
Chemical Formula: $C_{12}H_{10}BClF_3NO_4$
Molecular Weight: 335.4713

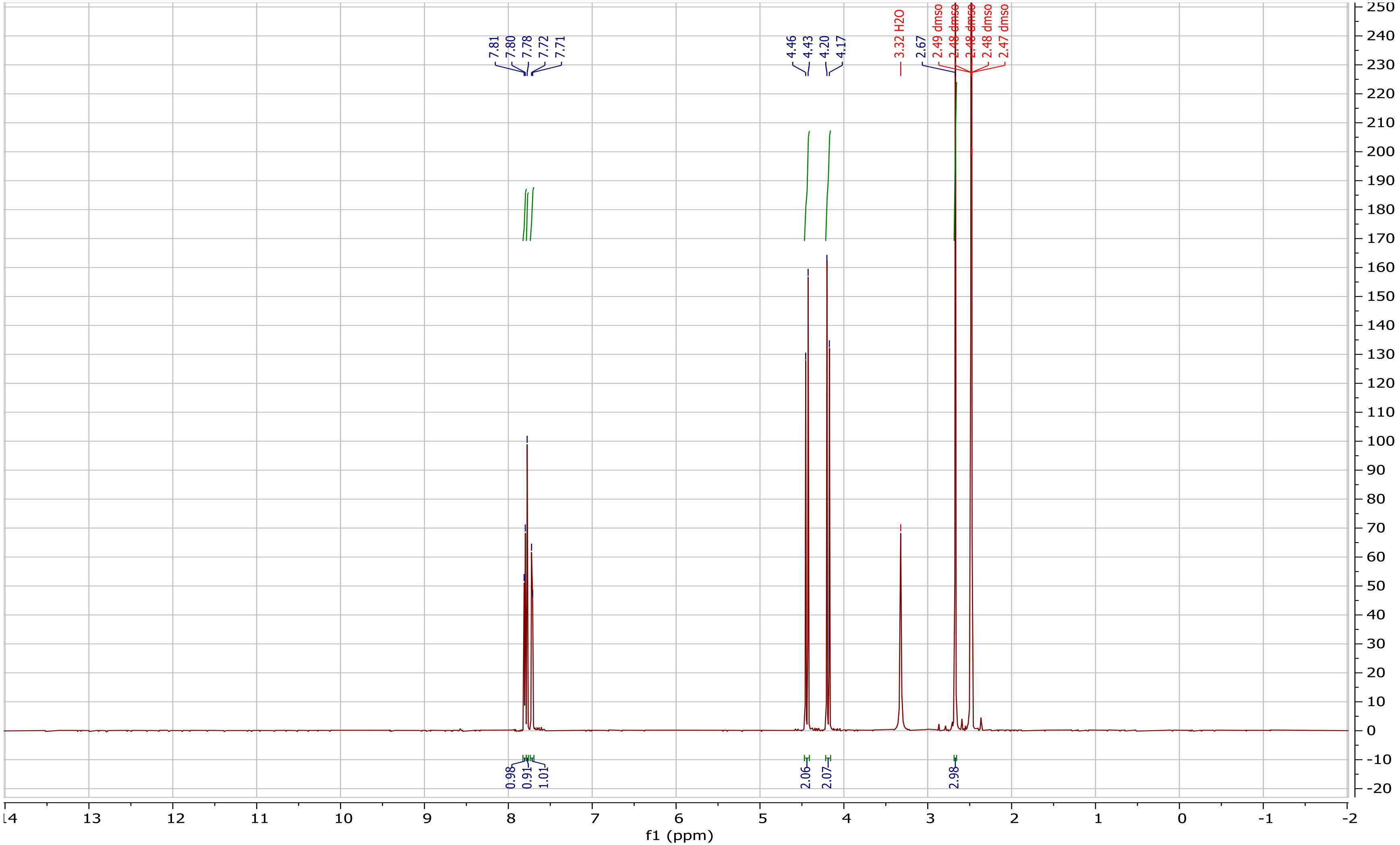

7.81
7.80
7.78
7.72
7.71
4.46
4.43
4.20
4.17
3.32 H2O
2.67
2.49 dmso
2.48 dmso
2.48 dmso
2.48 dmso
2.47 dmso
0.98
0.91
1.01
2.06
2.07
2.98
f1 (ppm)

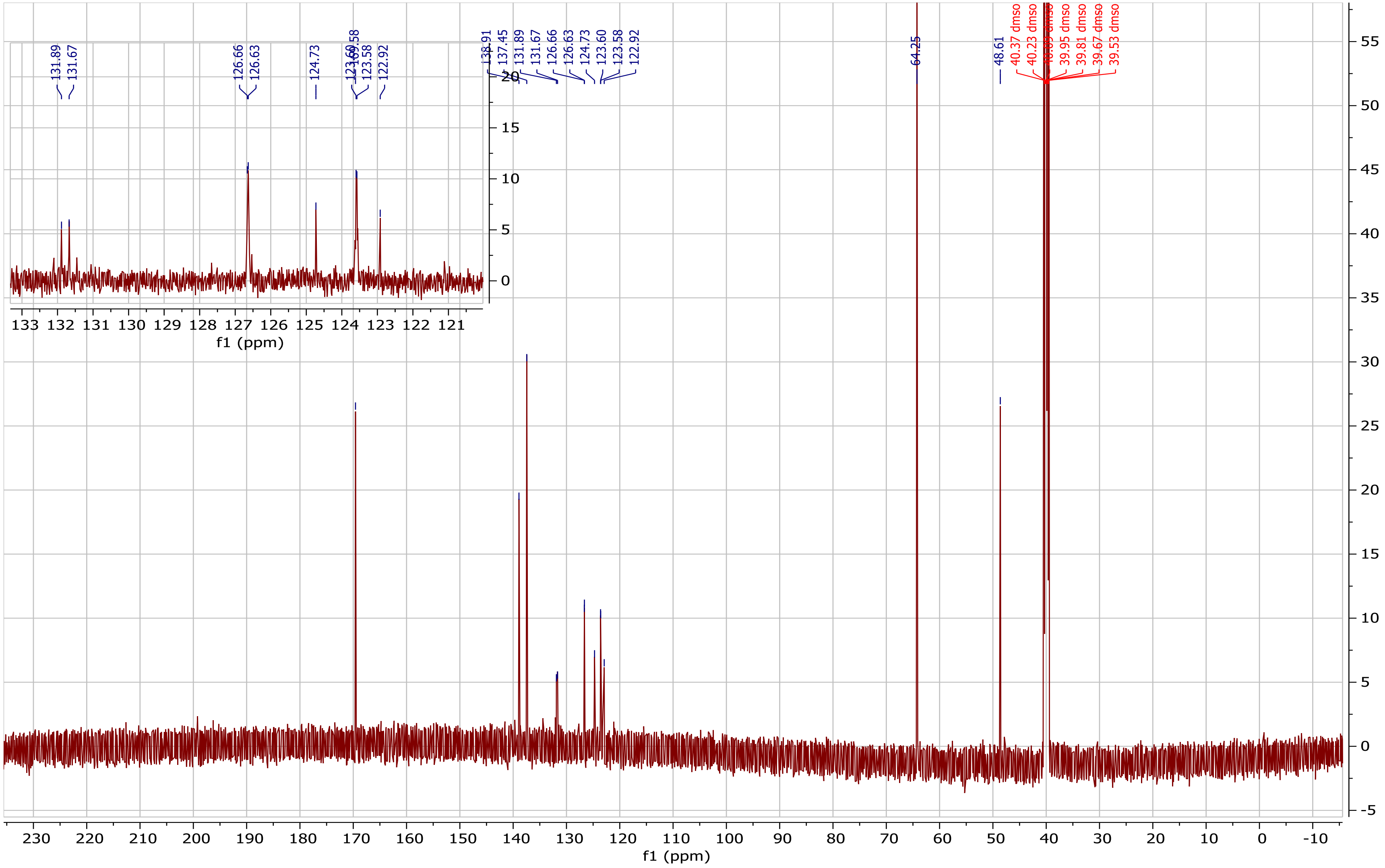

131.89
131.67
126.66
126.63
124.73
123.60
123.58
122.92
f1 (ppm)
138.91
137.45
169.58
131.89
131.67
126.66
126.63
124.73
123.60
123.58
122.92
64.25
48.61
40.37 dmso
40.23 dmso
39.95 dmso
39.81 dmso
39.67 dmso
39.53 dmso
f1 (ppm)

Single Mass Analysis
Tolerance = 5.0 mDa / DBE: min = -1.5, max = 120.0
Element prediction: Off
Number of isotope peaks used for i-FIT = 3
Monoisotopic Mass, Even Electron Ions
236 formula(e) evaluated with 1 results within limits (up to 20 best isotopic matches for each mass)
Elements Used:

Mass	Calc. Mass	mDa	PPM	DBE	Formula	i-FIT	i-FIT Norm	Fit Conf %	C	H	11B	N	O	Cl	F
353.0685	353.0687	-0.2	-0.6	5.5	C12 H14 11B N2 O4 Cl F3	197.3	n/a	n/a	12	14	1	2	4	1	3

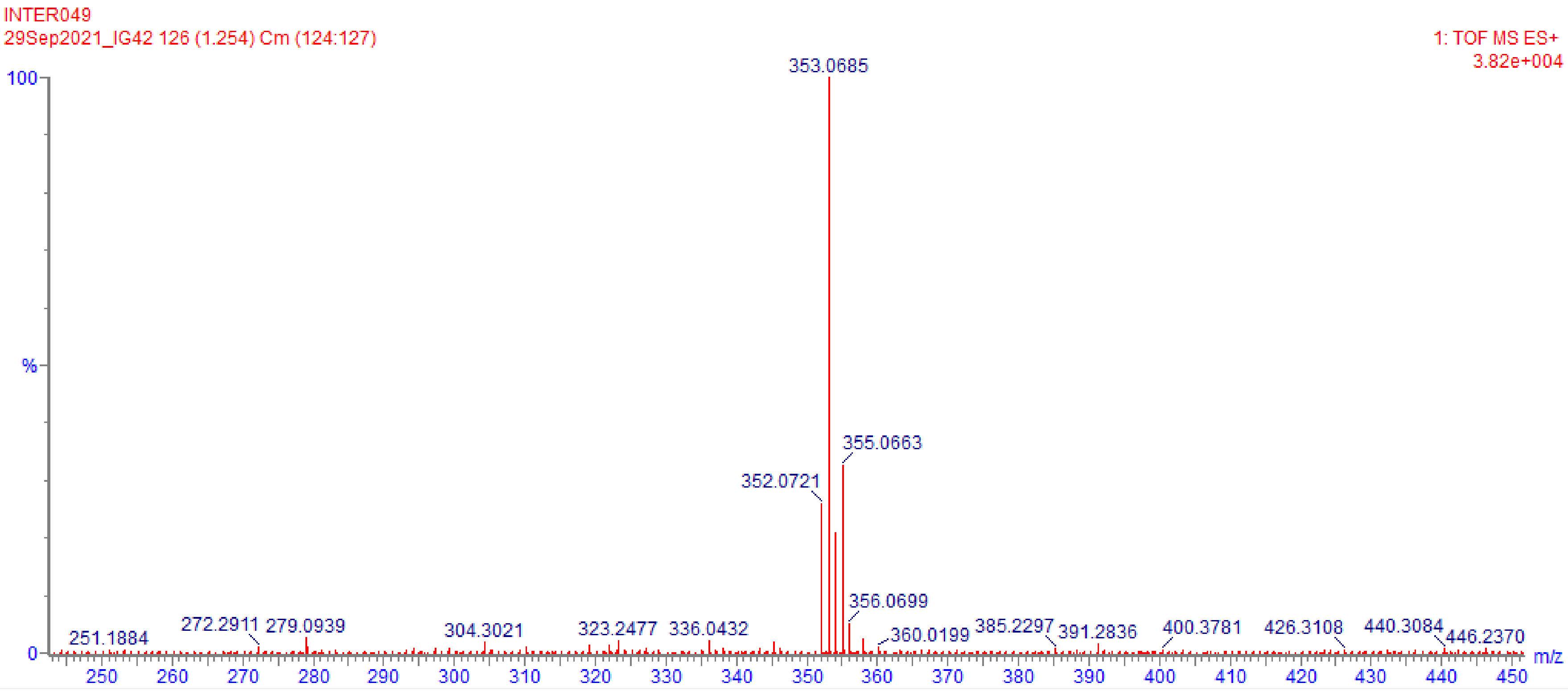

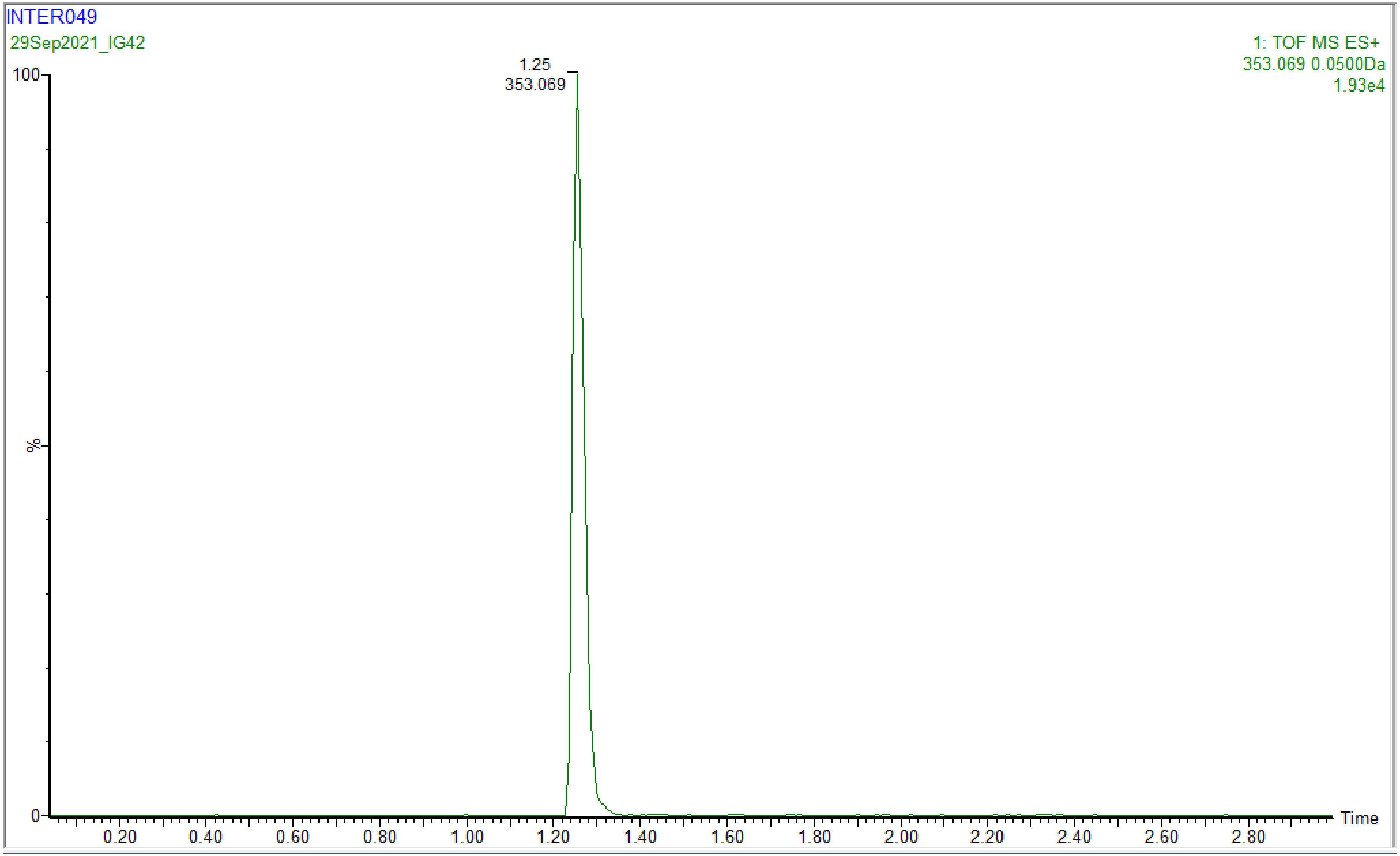

INTER049
29Sep2021_IG42
1: TOF MS ES+
353.069 0.0500Da
1.93e4
1.25
353.069
100
%
0
0.20
0.40
0.60
0.80
1.00
1.20
1.40
1.60
1.80
2.00
2.20
2.40
2.60
2.80
Time

2-(2,4-difluorophenyl)-6-methyl-1,3,6,2-dioxazaborocane-4,8-dione 8i

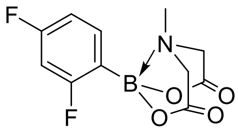

8i

2-(2,4-difluorophenyl)-6-methyl-1,3,6,2-dioxazaborocane-4,8-dione
Chemical Formula: $C_{11}H_{10}BF_2NO_4$
Molecular Weight: 269.0092

Yield = 209.7 mg, (78%).

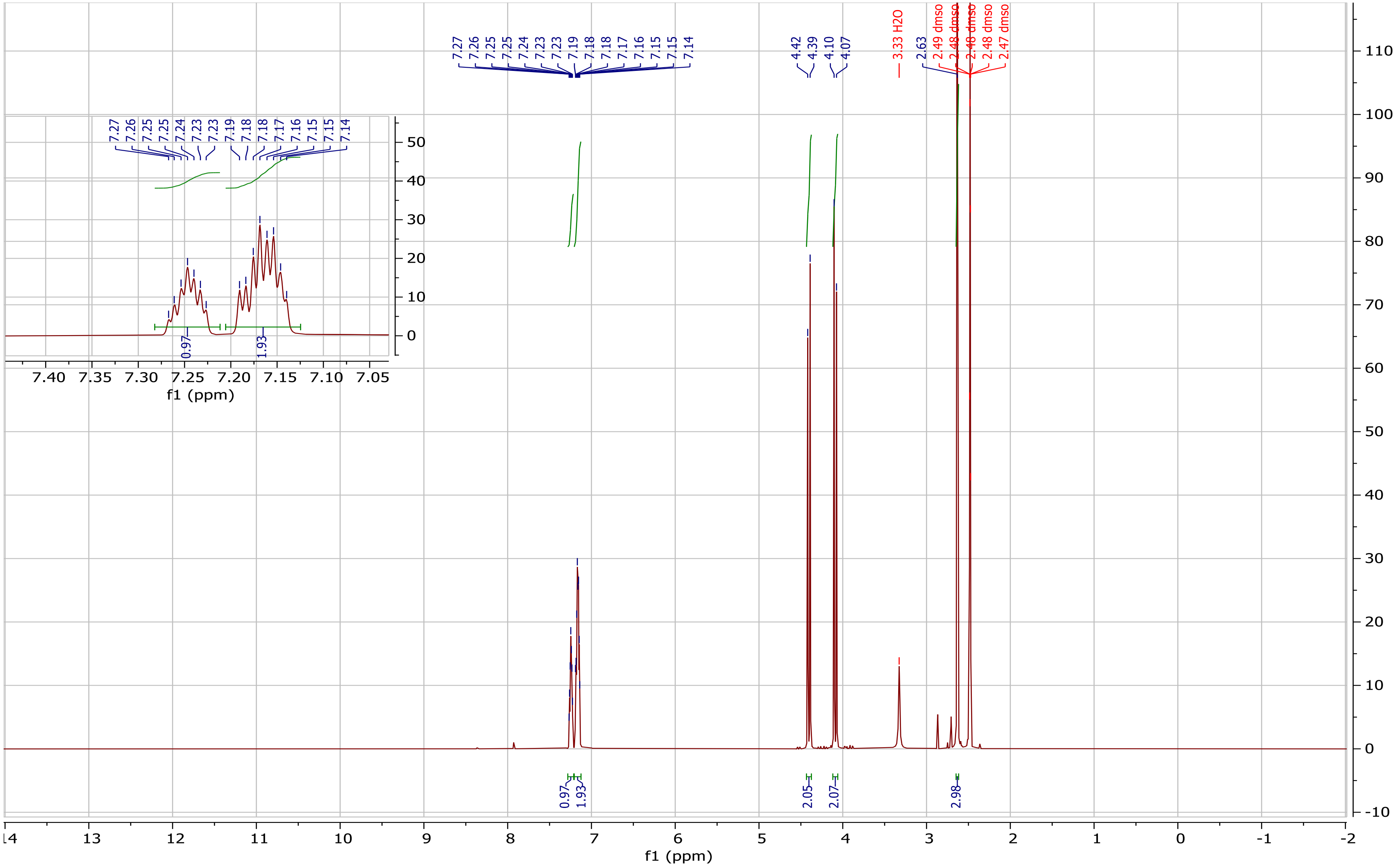

7.27 7.26 7.25 7.25 7.24 7.23 7.23 7.19 7.18 7.18 7.17 7.16 7.15 7.15 7.14
4.42 4.39 4.10 4.07
3.33 H2O
2.63
2.49 dmso 2.48 dmso 2.48 dmso 2.48 dmso 2.47 dmso
0.97 1.93 2.05 2.07 2.98
f1 (ppm)

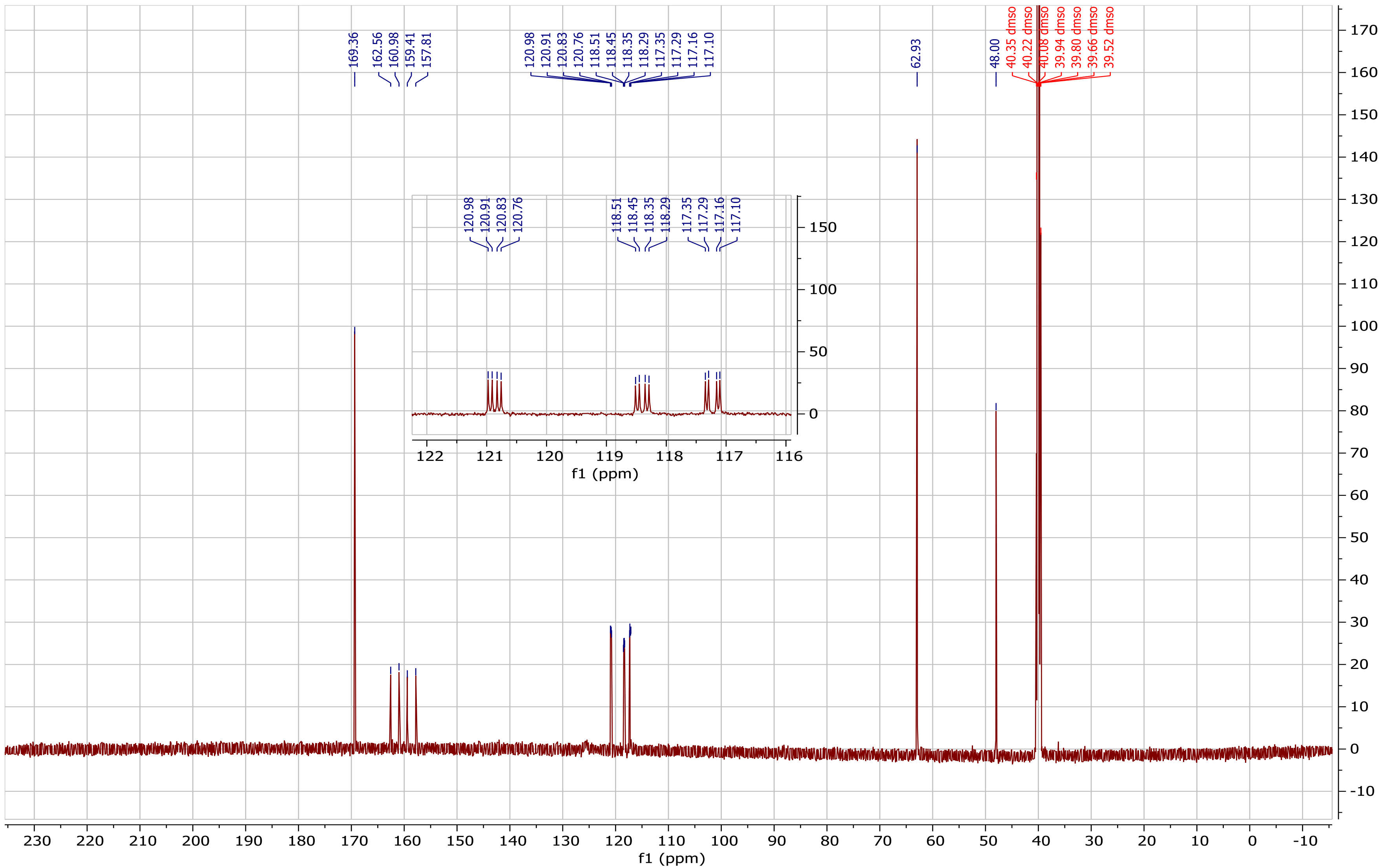

169.36
162.56
160.98
159.41
157.81
120.98
120.91
120.83
120.76
118.51
118.45
118.35
118.29
117.35
117.29
117.16
117.10
62.93
48.00
40.35 dmso
40.22 dmso
40.08 dmso
39.94 dmso
39.80 dmso
39.66 dmso
39.52 dmso
f1 (ppm)

Single Mass Analysis

Tolerance = 5.0 mDa / DBE: min = -1.5, max = 120.0

Element prediction: Off

Number of isotope peaks used for i-FIT = 3

Monoisotopic Mass, Even Electron Ions

56 formula(e) evaluated with 1 results within limits (up to 20 best isotopic matches for each mass)

Elements Used:

Mass	Calc. Mass	mDa	PPM	DBE	Formula	i-FIT	i-FIT Norm	Fit Conf %	C	H	11B	N	O	F
270.0743	270.0749	-0.6	-2.2	6.5	C11 H11 11B N O4 F2	141.6	n/a	n/a	11	11	1	1	4	2

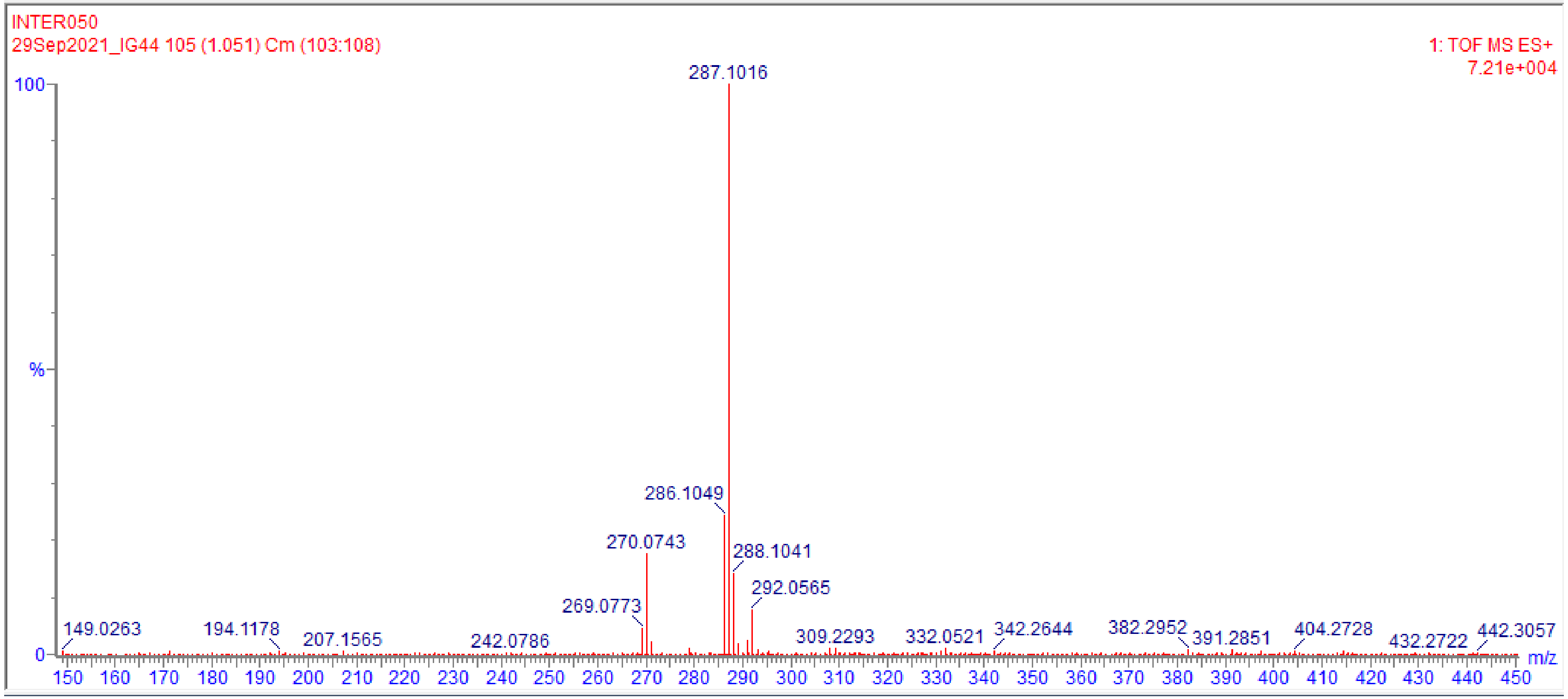

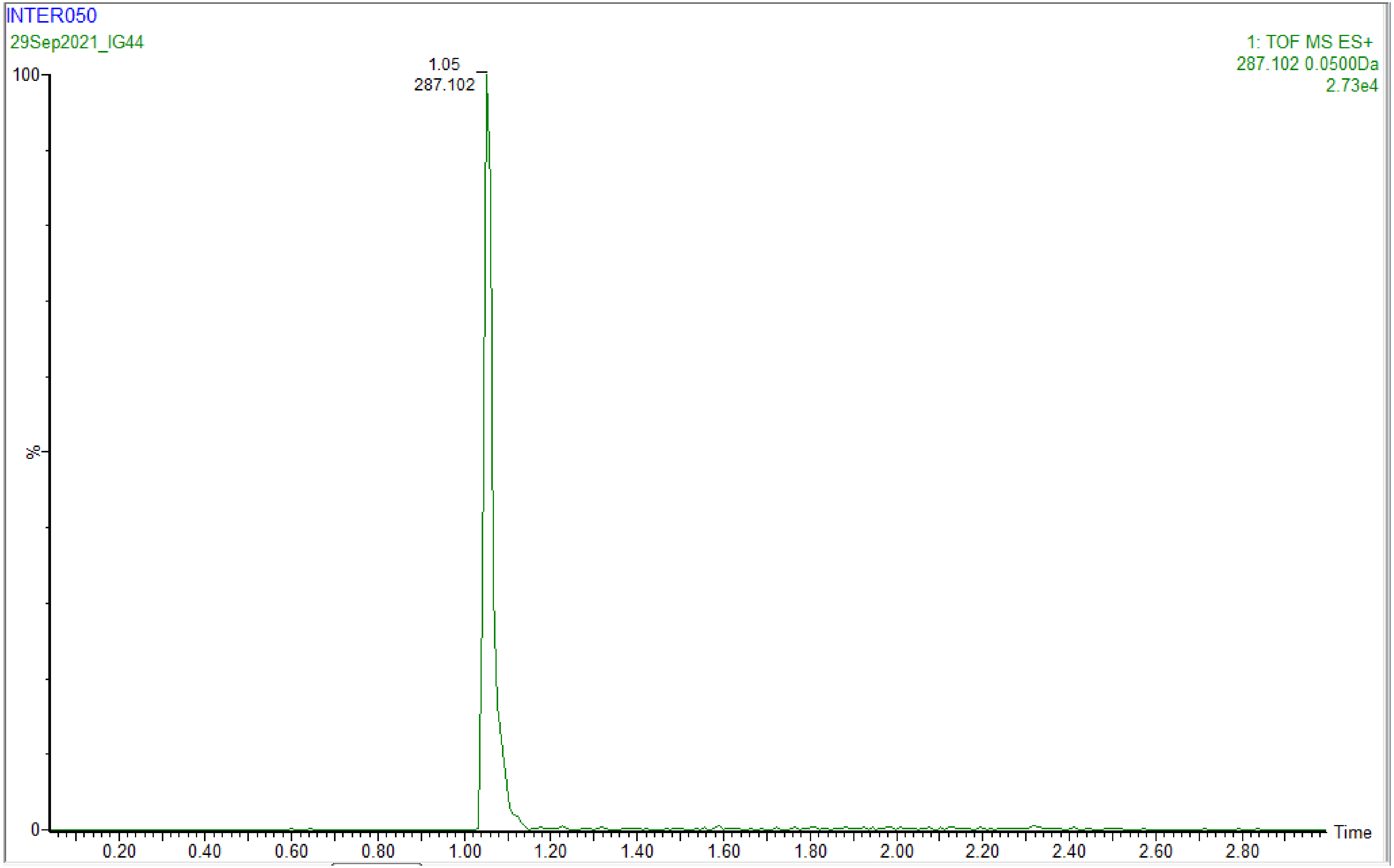
INTER050
29Sep2021_IG44
1: TOF MS ES+
287.102 0.0500Da
2.73e4
1.05
287.102
100
%
0
0.20
0.40
0.60
0.80
1.00
1.20
1.40
1.60
1.80
2.00
2.20
2.40
2.60
2.80
Time

N-(*tert*-butyl)-3-(6-methyl-4,8-dioxo-1,3,6,2-dioxazaborocan-2-yl)benzamide 8j

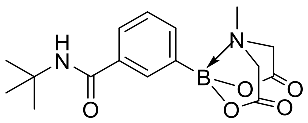

8j

N-(*tert*-butyl)-3-(6-methyl-4,8-dioxo-1,3,6,2-dioxazaborocan-2-yl)benzamide
Chemical Formula: $C_{16}H_{21}BN_2O_5$
Molecular Weight: 332.1593

Yield = 129.0 mg (39%).

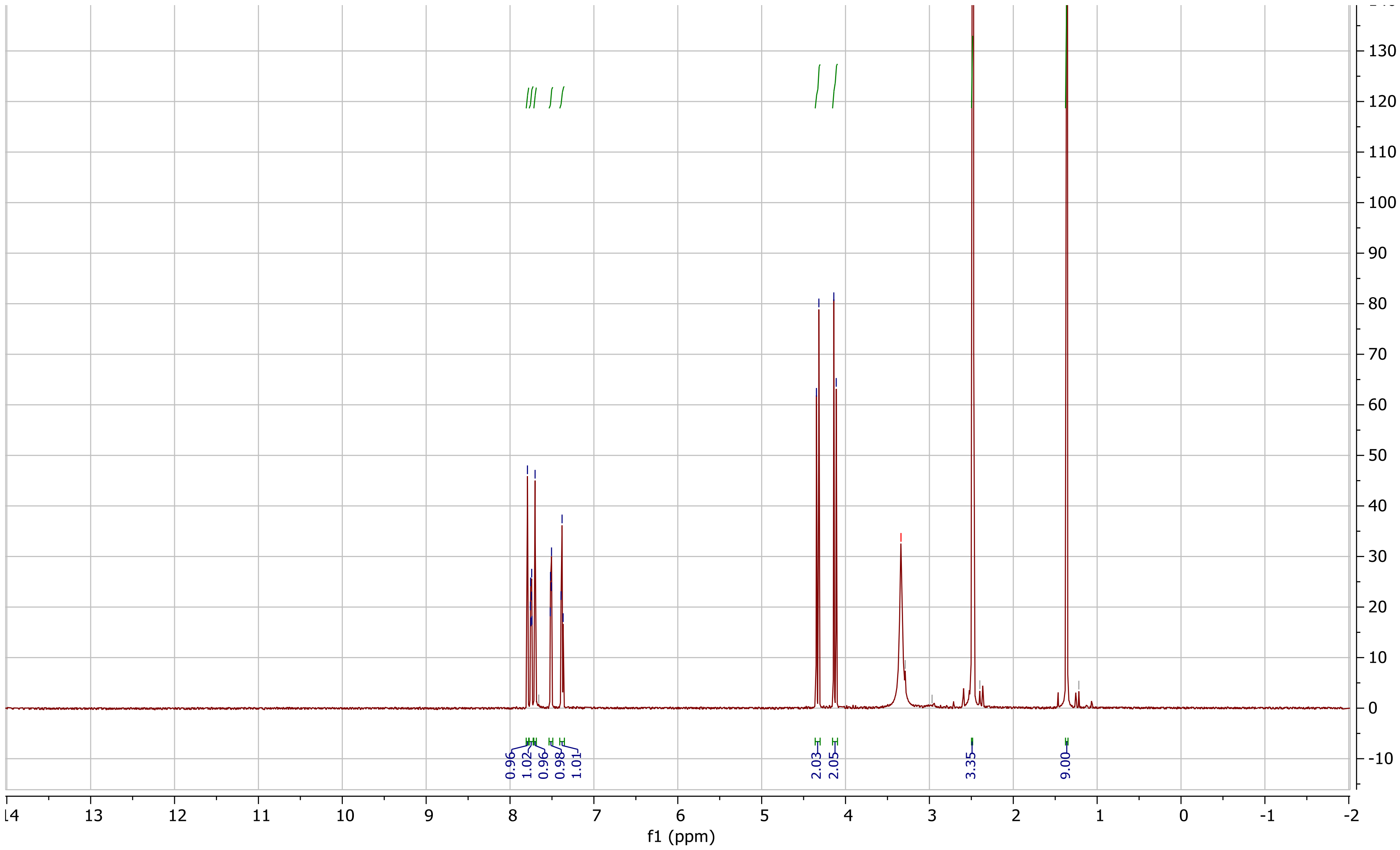

0.96
1.02
0.96
0.98
1.01
2.03
2.05
3.35
9.00
f1 (ppm)

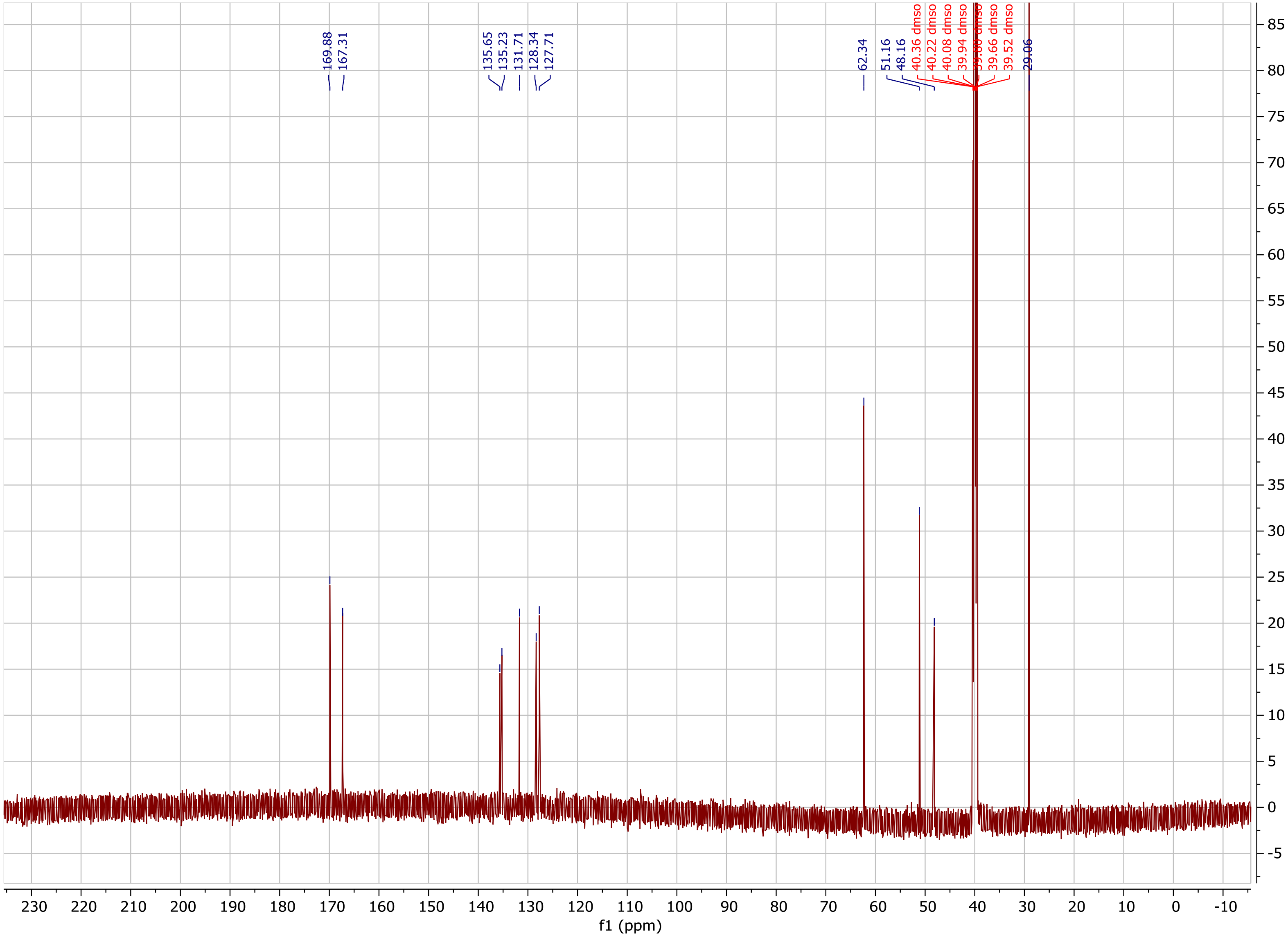
169.88
167.31
135.65
135.23
131.71
128.34
127.71
62.34
51.16
48.16
40.36 dmso
40.22 dmso
40.08 dmso
39.94 dmso
39.66 dmso
39.52 dmso
29.06
f1 (ppm)

Single Mass Analysis

Tolerance = 5.0 mDa / DBE: min = -1.5, max = 120.0

Element prediction: Off

Number of isotope peaks used for i-FIT = 3

Monoisotopic Mass, Even Electron Ions

68 formula(e) evaluated with 1 results within limits (up to 20 best isotopic matches for each mass)

Elements Used:

Mass	Calc. Mass	mDa	PPM	DBE	Formula	i-FIT	i-FIT Norm	Fit Conf %	C	H	11B	N	O	23Na
355.1446	355.1441	0.5	1.4	7.5	C16 H21 11B N2 O5 23Na	101.5	n/a	n/a	16	21	1	2	5	1

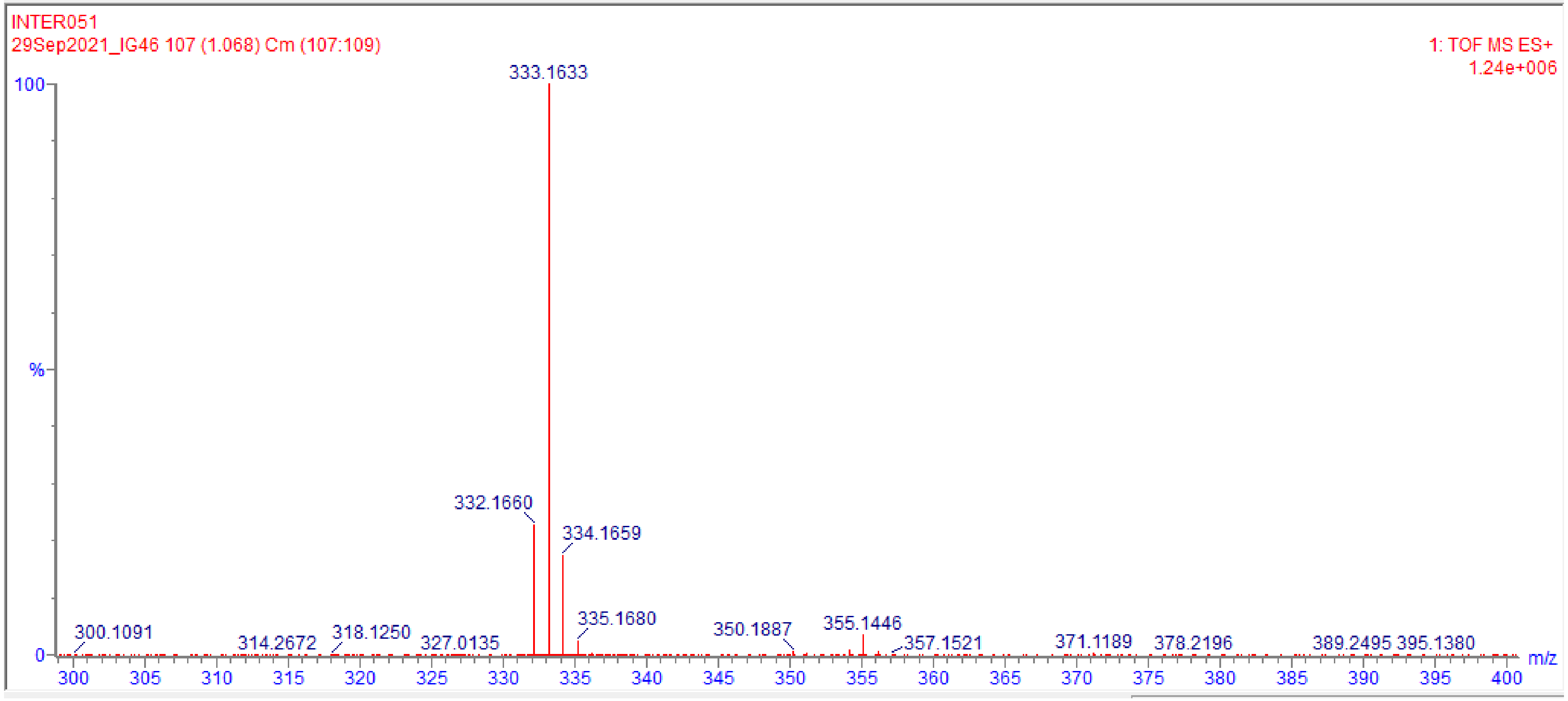

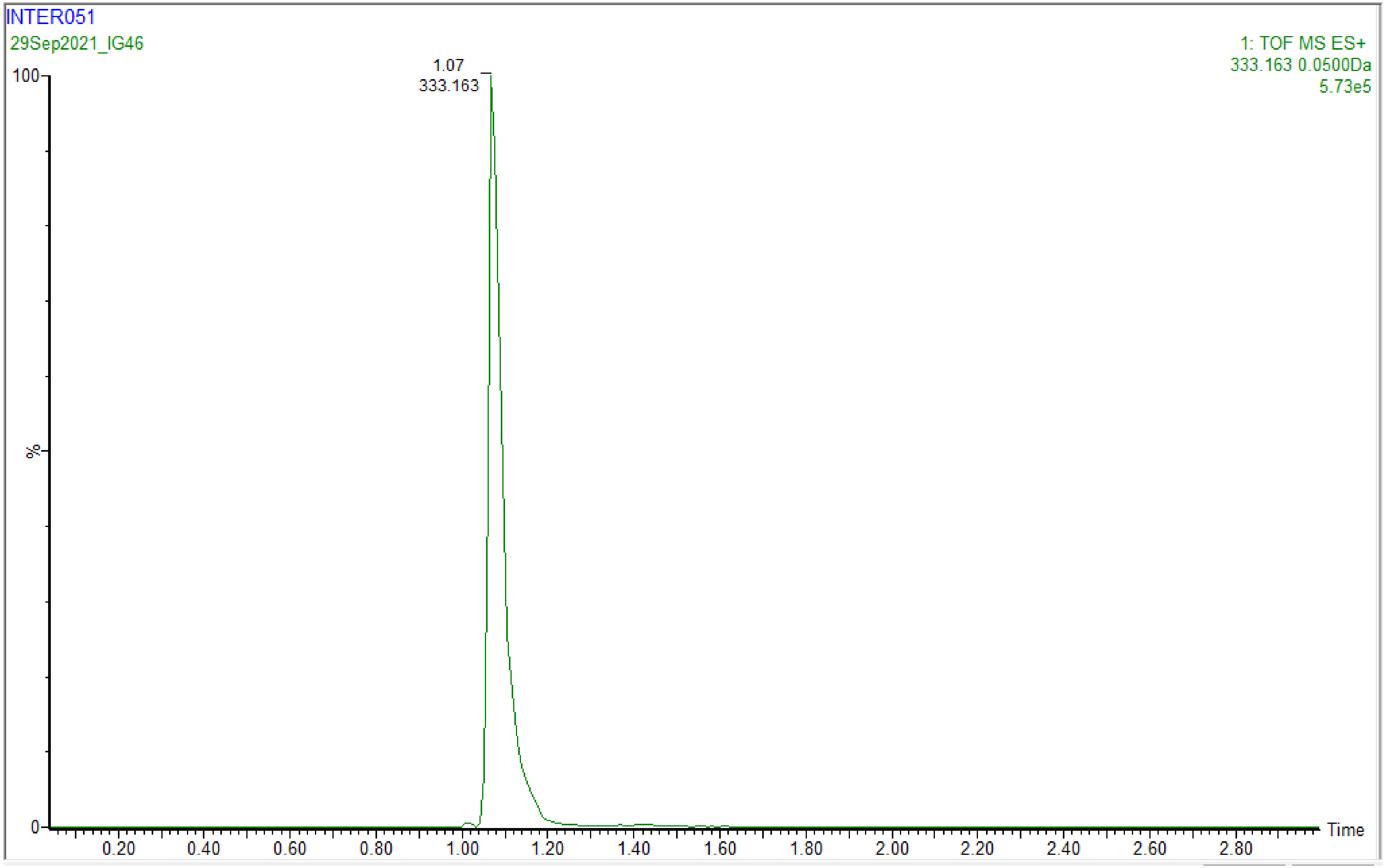
INTER051
29Sep2021_IG46
1: TOF MS ES+
333.163 0.0500Da
5.73e5
1.07
333.163
100
%
0
0.20
0.40
0.60
0.80
1.00
1.20
1.40
1.60
1.80
2.00
2.20
2.40
2.60
2.80
Time

6-Methyl-2-(4-(pyrrolidine-1-carbonyl)phenyl)-1,3,6,2-dioxazaborocane-4,8-dione 8k

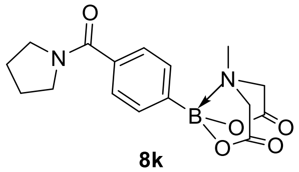

8k

6-methyl-2-(4-(pyrrolidine-1-carbonyl)phenyl)-1,3,6,2-dioxazaborocane-4,8-dione
Chemical Formula: $C_{16}H_{19}BN_2O_5$
Molecular Weight: 330.1435

Yield = 83.2 mg (25%).

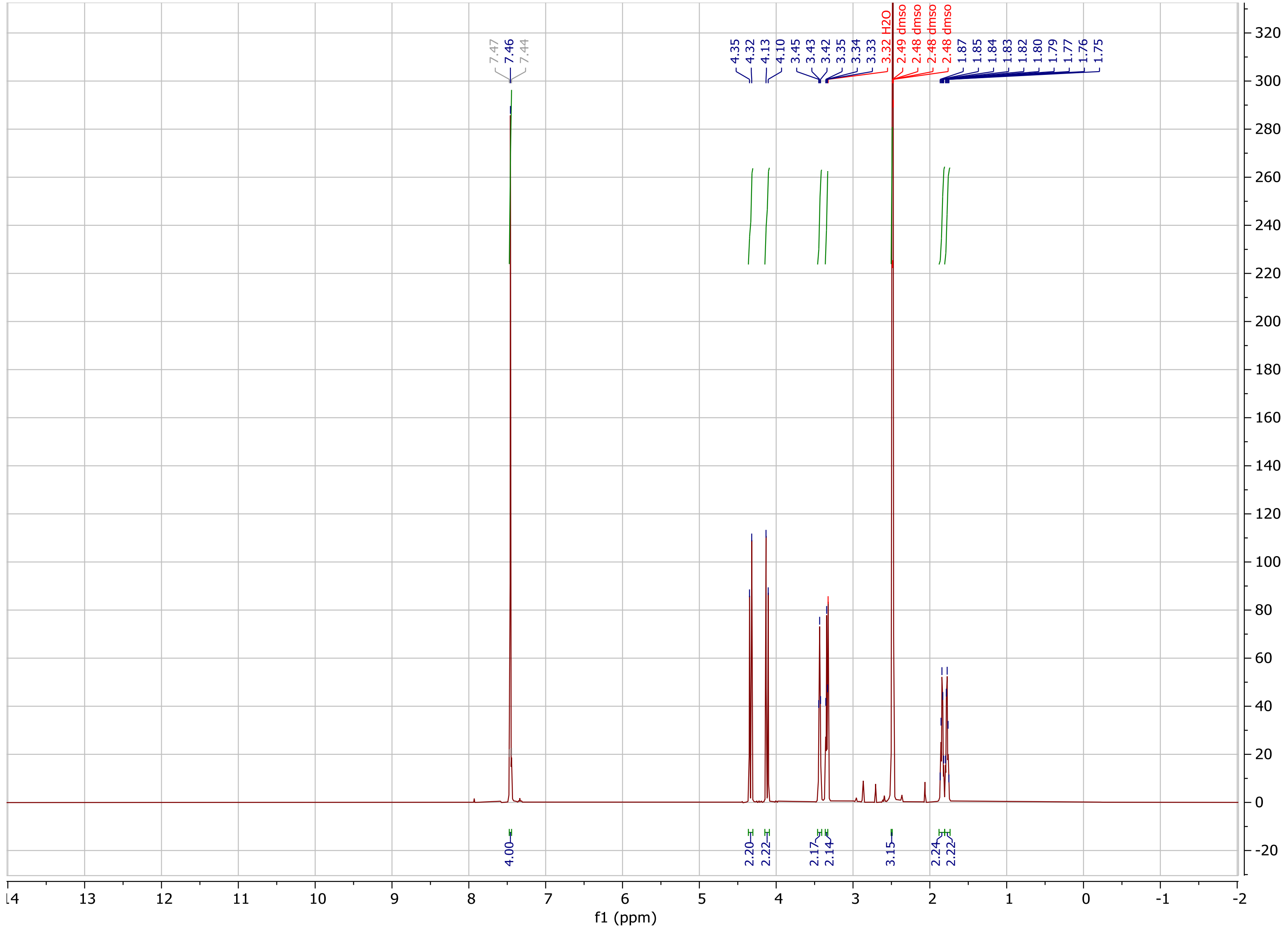
7.47
7.46
7.44
4.35
4.32
4.13
4.10
3.45
3.43
3.42
3.35
3.34
3.33
3.32 H2O
2.49 dmso
2.48 dmso
2.48 dmso
2.48 dmso
1.87
1.85
1.84
1.83
1.82
1.80
1.79
1.77
1.76
1.75
4.00
2.20
2.22
2.17
2.14
3.15
2.24
2.22
f1 (ppm)

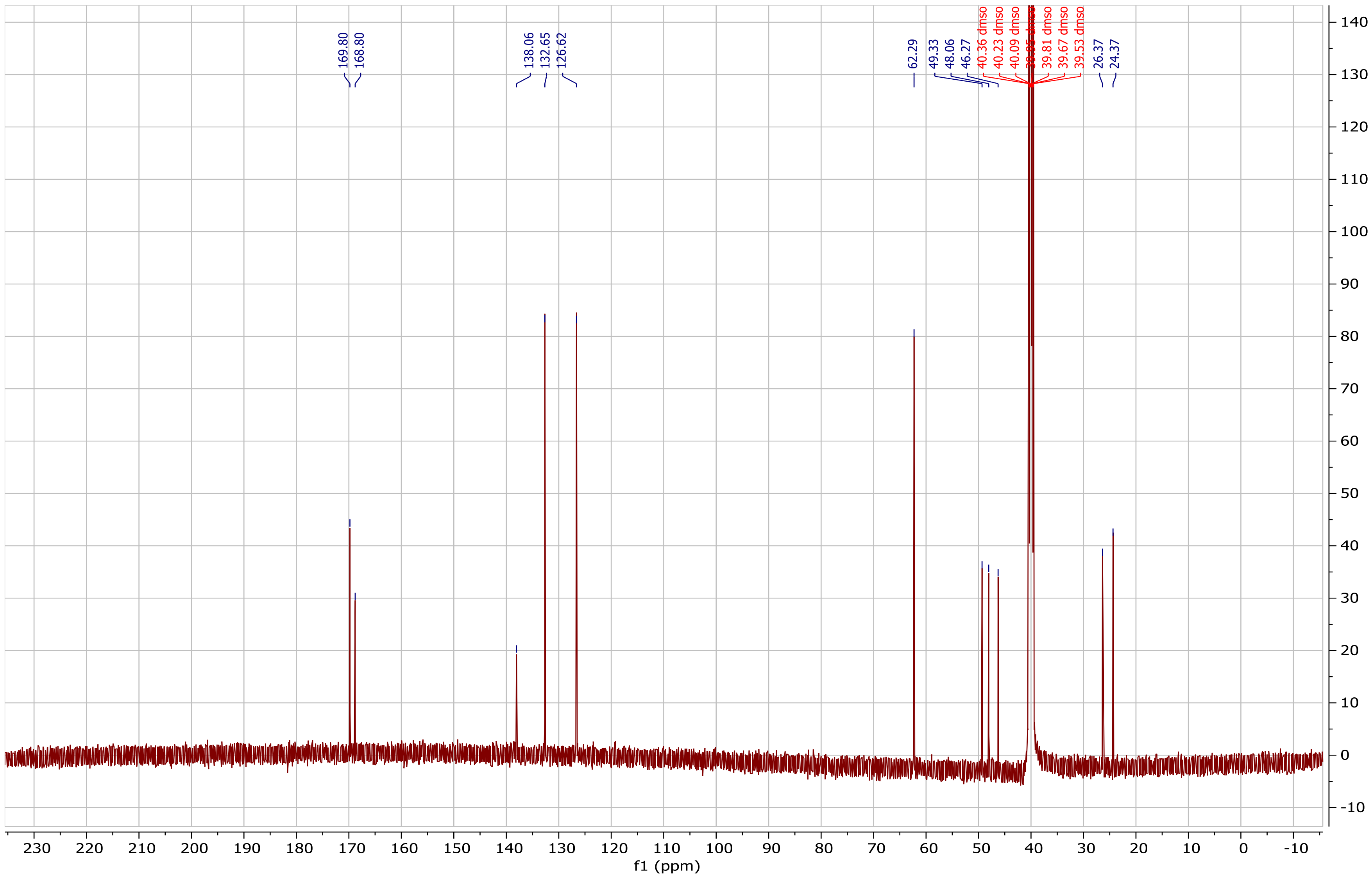
169.80
168.80
138.06
132.65
126.62
62.29
49.33
48.06
46.27
40.36 dmso
40.23 dmso
40.09 dmso
39.81 dmso
39.67 dmso
39.53 dmso
26.37
24.37
f1 (ppm)

Single Mass Analysis
Tolerance = 5.0 mDa / DBE: min = -1.5, max = 120.0
Element prediction: Off
Number of isotope peaks used for i-FIT = 3
Monoisotopic Mass, Even Electron Ions
87 formula(e) evaluated with 1 results within limits (up to 20 best isotopic matches for each mass)
Elements Used:

Mass	Calc. Mass	mDa	PPM	DBE	Formula	i-FIT	i-FIT Norm	Fit Conf %	C	H	11B	N	O	23Na
353.1286	353.1285	0.1	0.3	8.5	C16 H19 11B N2 O5 23Na	172.6	n/a	n/a	16	19	1	2	5	1

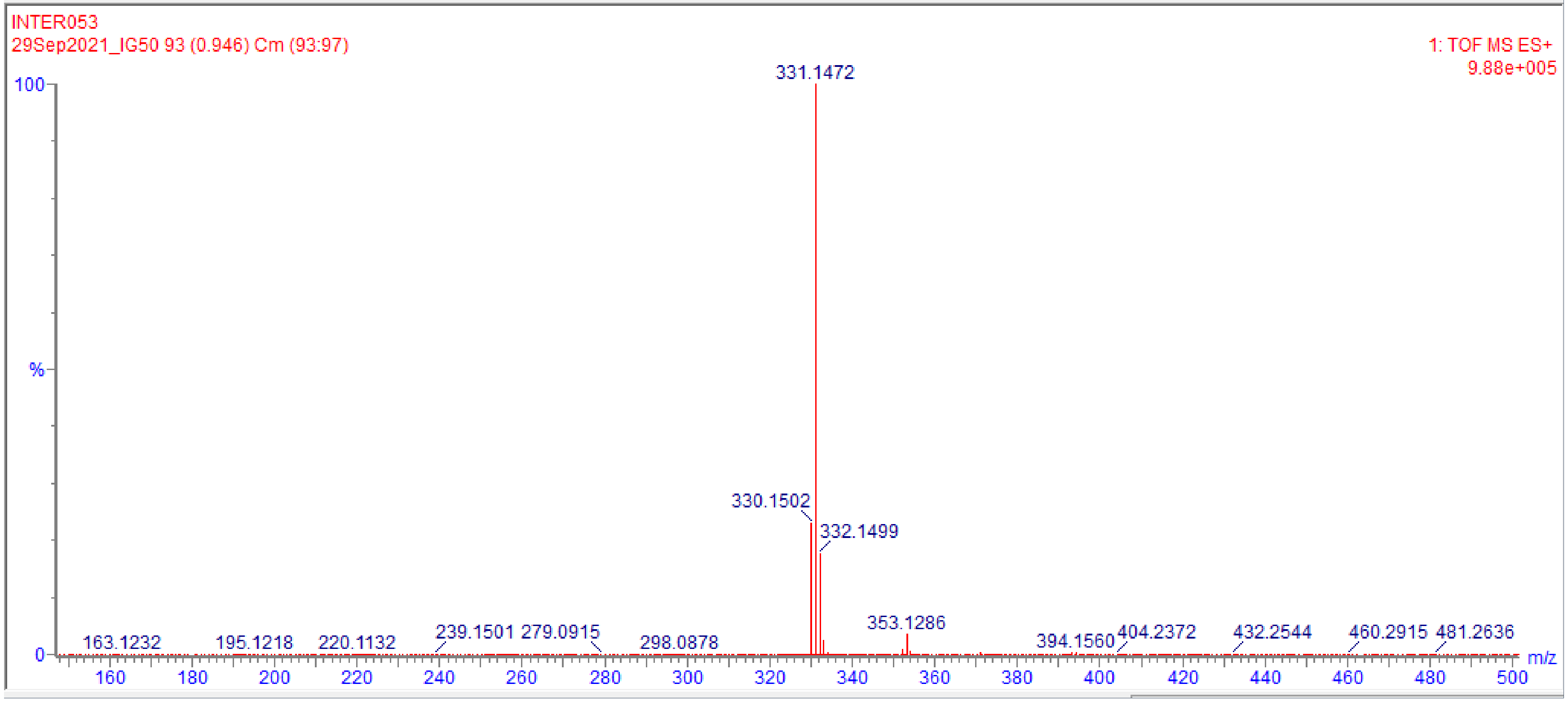

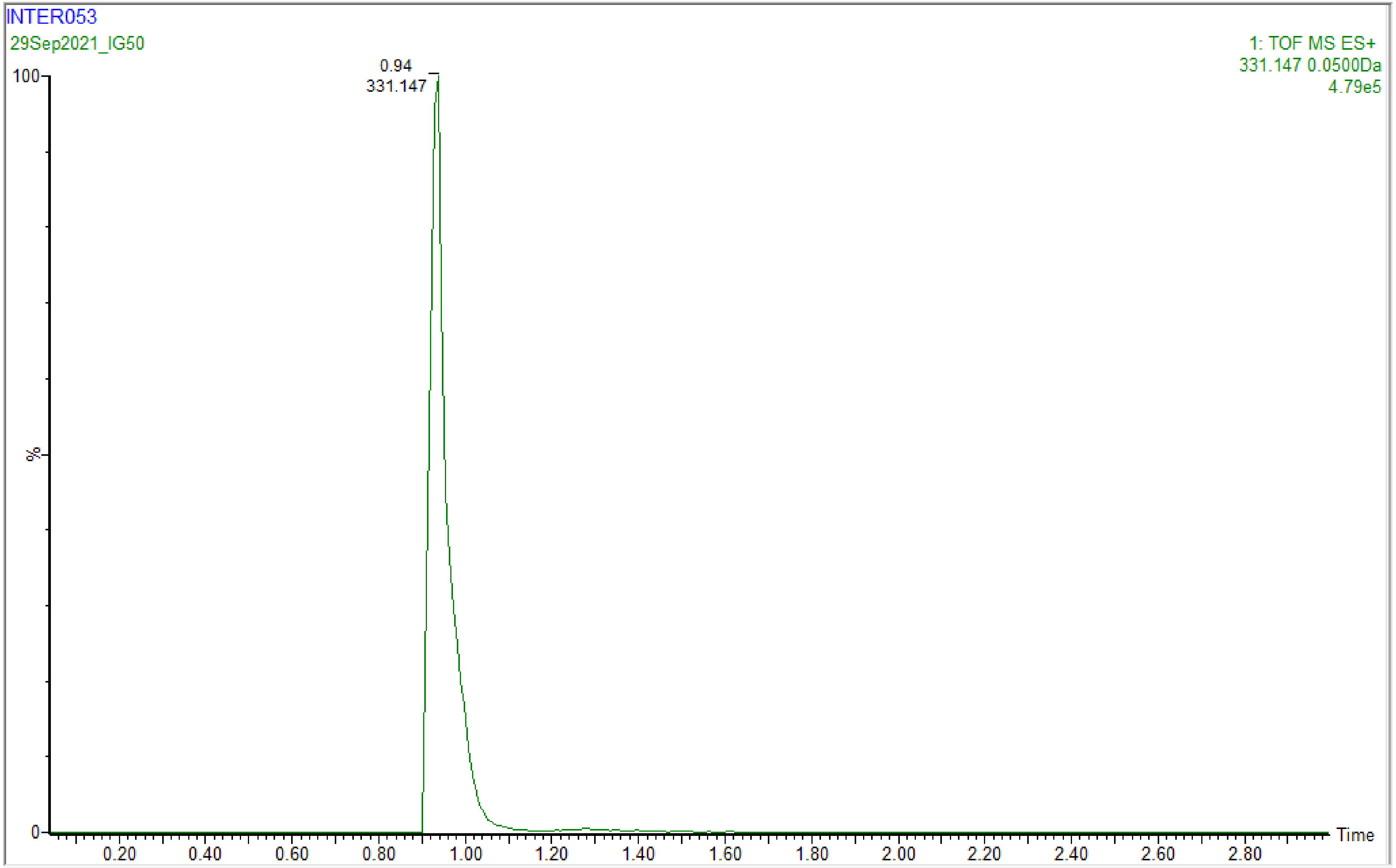
INTER053
29Sep2021_IG50
1: TOF MS ES+
331.147 0.0500Da
4.79e5
0.94
331.147
100
%
0
0.20
0.40
0.60
0.80
1.00
1.20
1.40
1.60
1.80
2.00
2.20
2.40
2.60
2.80
Time

2-([1,1'-Biphenyl]-3-yl)-6-methyl-1,3,6,2-dioxazaborocane-4,8-dione 8l

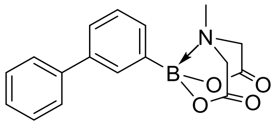

8l

2-([1,1'-biphenyl]-3-yl)-6-methyl-1,3,6,2-dioxazaborocane-4,8-dione
Chemical Formula: $C_{17}H_{16}BNO_4$
Molecular Weight: 309.1242

Yeild = 281.6 mg (86%).

PROTON_01
AEPD-0050-1

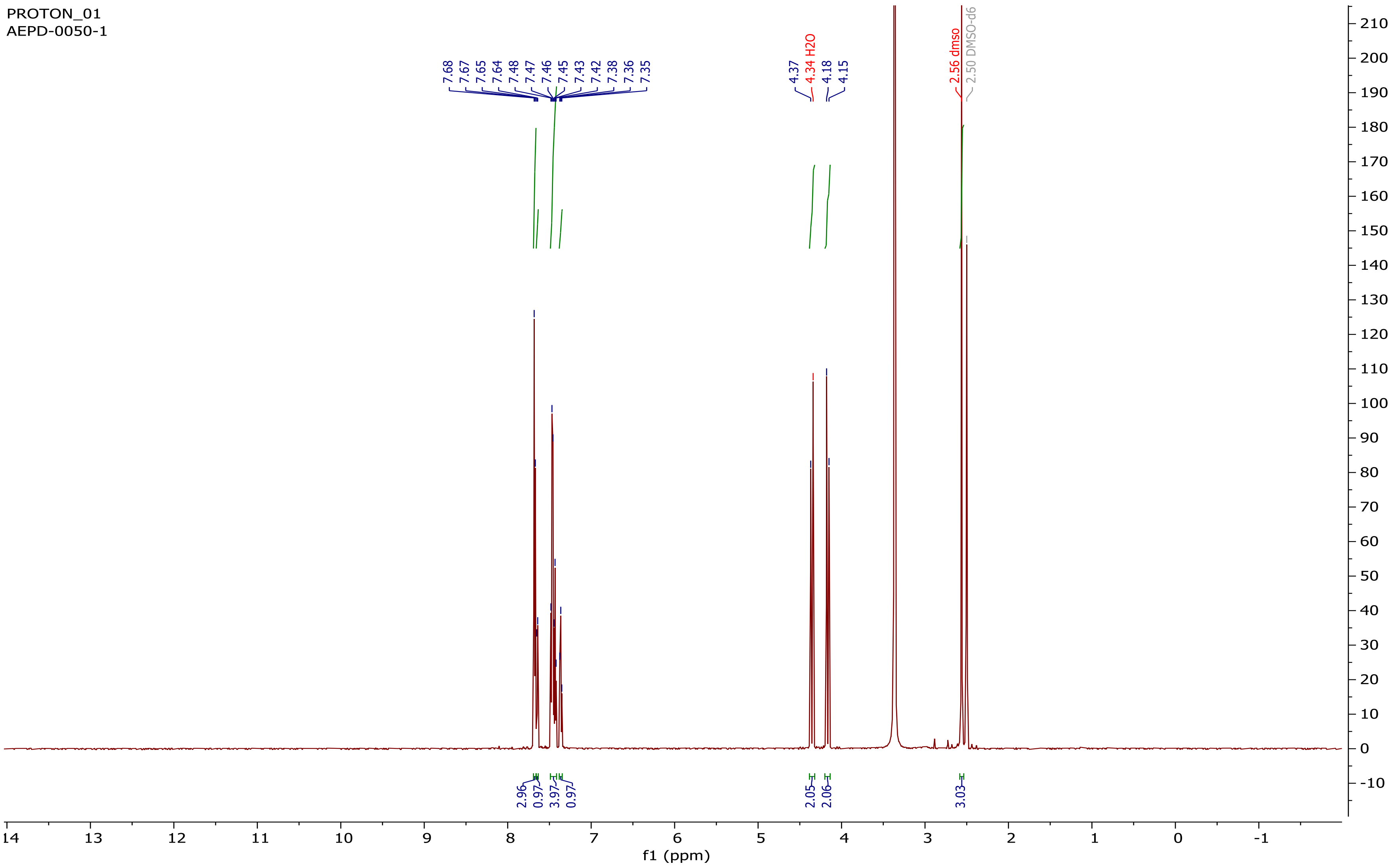

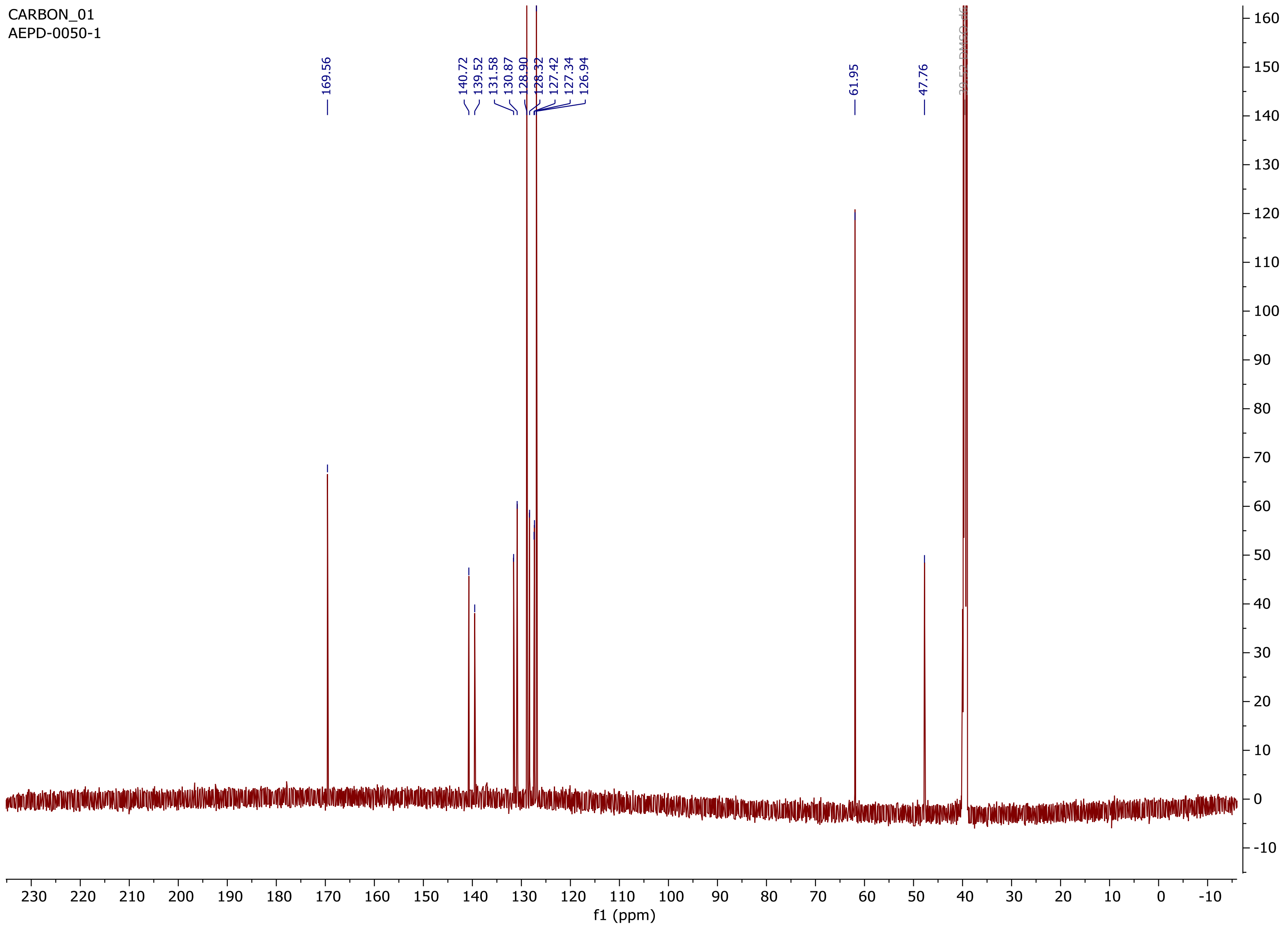

CARBON_01
AEPD-0050-1
169.56
140.72
139.52
131.58
130.87
128.90
128.32
127.42
127.34
126.94
61.95
47.76
f1 (ppm)

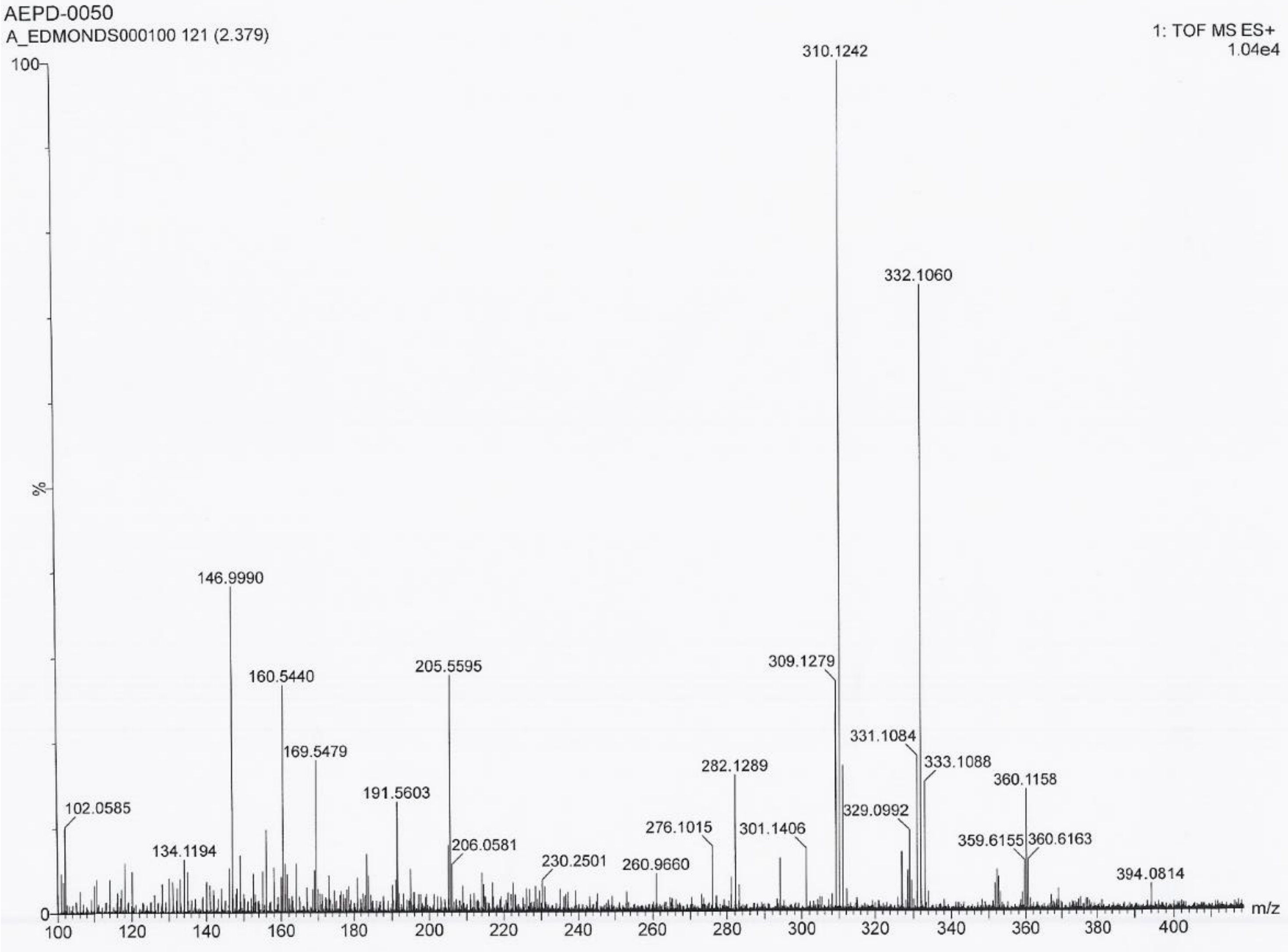
AEPD-0050
A_EDMONDS000100 121 (2.379)
1: TOF MS ES+
1.04e4
100
%
0
310.1242
332.1060
146.9990
205.5595
309.1279
160.5440
331.1084
169.5479
333.1088
282.1289
360.1158
191.5603
102.0585
329.0992
276.1015
301.1406
359.6155
360.6163
134.1194
206.0581
230.2501
260.9660
394.0814
100
120
140
160
180
200
220
240
260
280
300
320
340
360
380
400
m/z

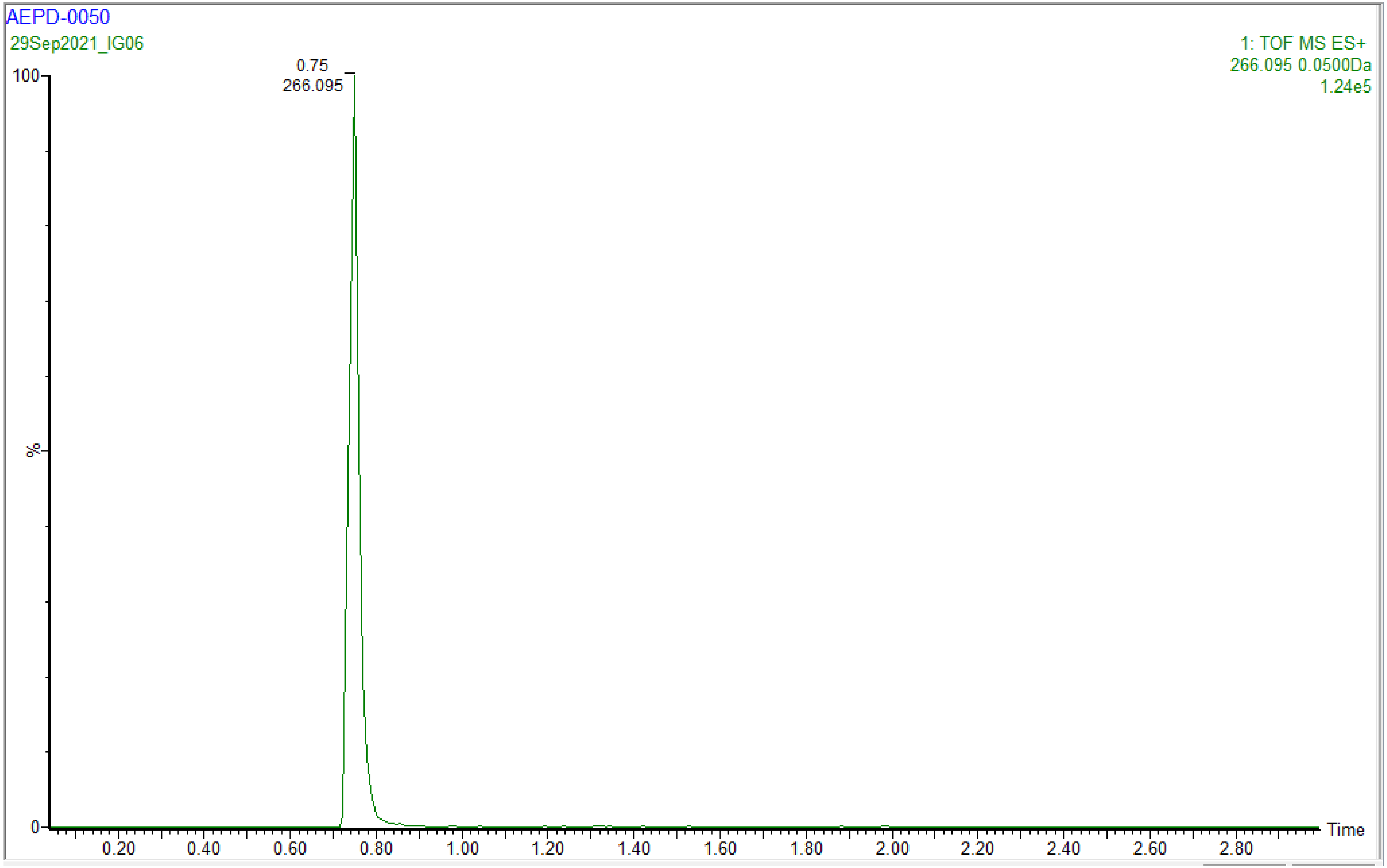
AEPD-0050
29Sep2021_IG06
1: TOF MS ES+
266.095 0.0500Da
1.24e5
0.75
266.095
100
%
0
Time
0.20
0.40
0.60
0.80
1.00
1.20
1.40
1.60
1.80
2.00
2.20
2.40
2.60
2.80

2-(3,5-bis(Trifluoromethyl)phenyl)-6-methyl-1,3,6,2-dioxazaborocane-4,8-dione 8m

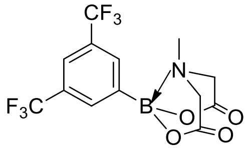

8m

2-(3,5-bis(trifluoromethyl)phenyl)-6-methyl-1,3,6,2-dioxazaborocane-4,8-dione
Chemical Formula: $C_{13}H_{10}BF_6NO_4$
Molecular Weight: 369.0242

Yield = 311.6 mg (80%).

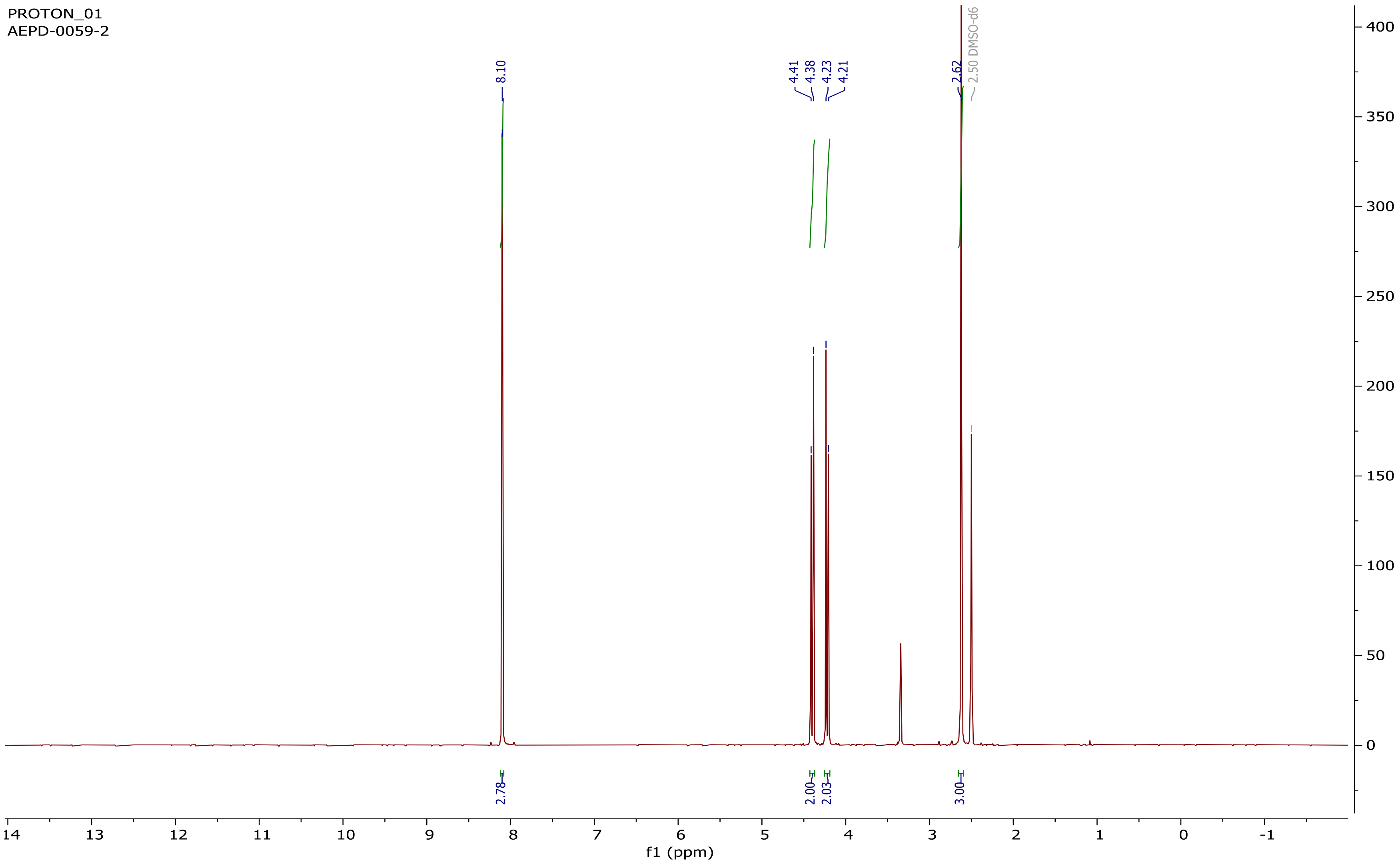

PROTON_01
AEPD-0059-2
8.10
4.41
4.38
4.23
4.21
2.62
2.50 DMSO-d6
2.78
2.00
2.03
3.00
f1 (ppm)

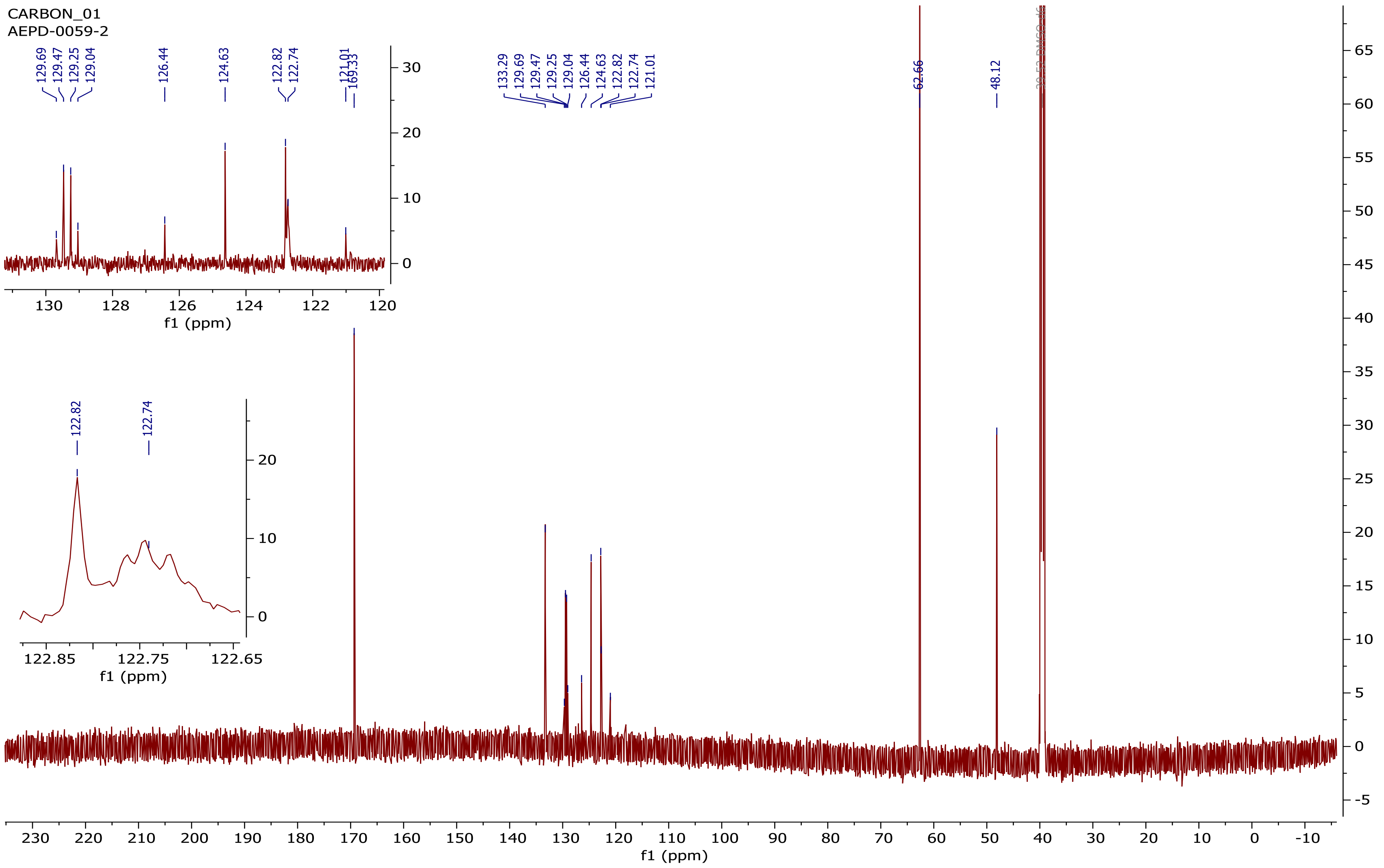

CARBON_01
AEPD-0059-2
129.69
129.47
129.25
129.04
126.44
124.63
122.82
122.74
121.01
169.33
f1 (ppm)
122.82
122.74
f1 (ppm)
133.29
129.69
129.47
129.25
129.04
126.44
124.63
122.82
122.74
121.01
62.66
48.12
f1 (ppm)

Single Mass Analysis
Tolerance = 5.0 mDa / DBE: min = -1.5, max = 120.0
Element prediction: Off
Number of isotope peaks used for i-FIT = 3
Monoisotopic Mass, Even Electron Ions
200 formula(e) evaluated with 1 results within limits (up to 20 best isotopic matches for each mass)
Elements Used:

Mass	Calc. Mass	mDa	PPM	DBE	Formula	i-FIT	i-FIT Norm	Fit Conf %	C	H	11B	N	O	F
370.0676	370.0685	-0.9	-2.4	6.5	C13 H11 11B N O4 F6	19.6	n/a	n/a	13	11	1	1	4	6

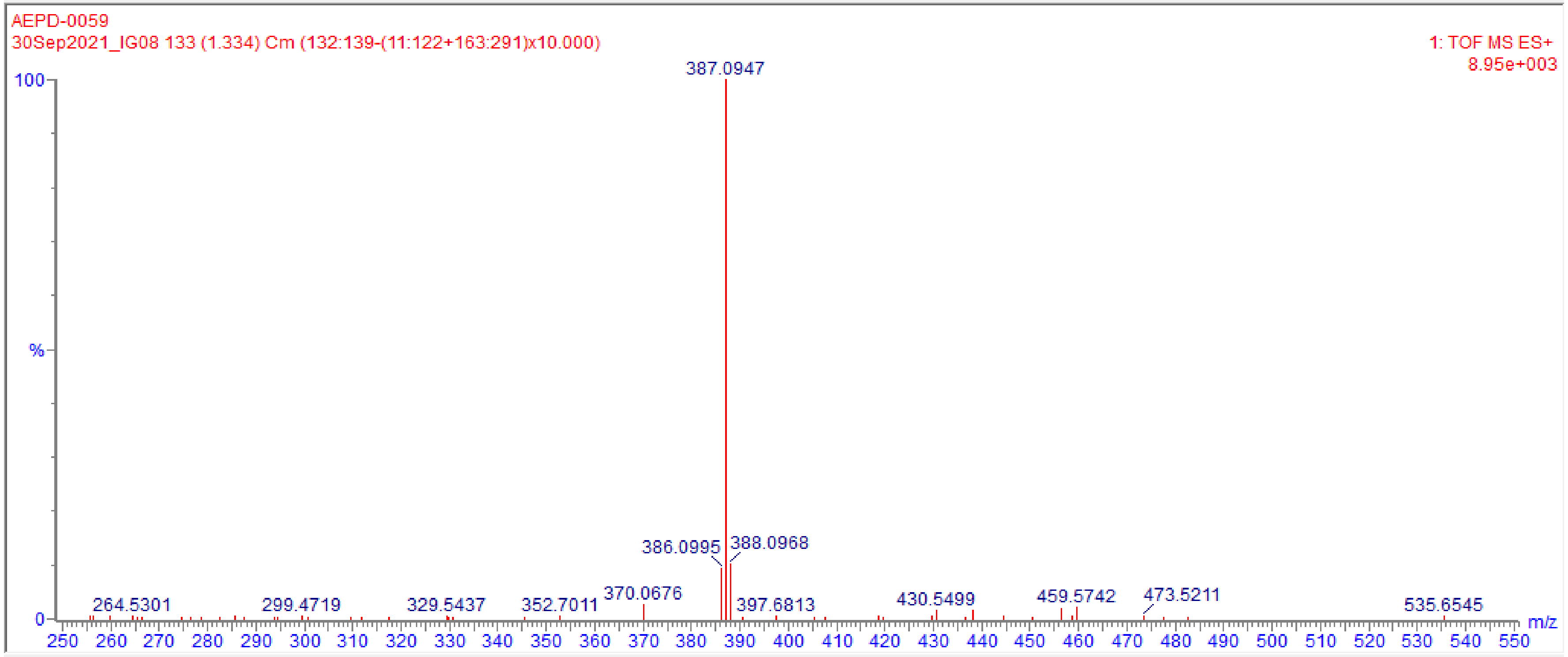

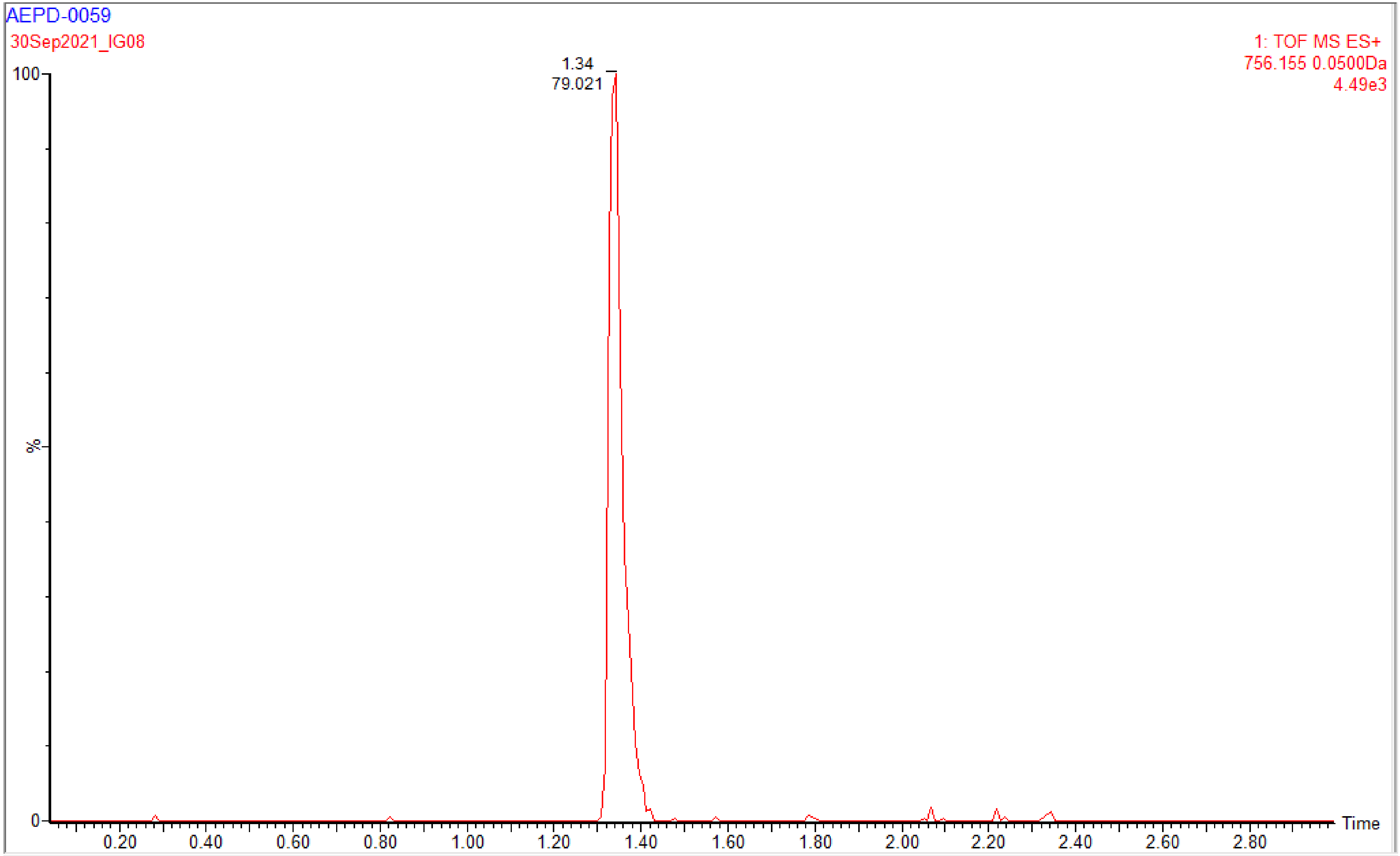
AEPD-0059
30Sep2021_IG08
1: TOF MS ES+
756.155 0.0500Da
4.49e3
1.34
79.021
100
%
0
0.20
0.40
0.60
0.80
1.00
1.20
1.40
1.60
1.80
2.00
2.20
2.40
2.60
2.80
Time

6-Methyl-2-(4-(pyrrolidin-1-ylsulfonyl)phenyl)-1,3,6,2-dioxazaborocane-4,8-dione 8n

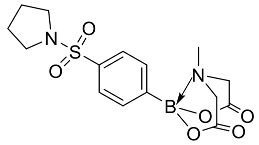

8n

6-methyl-2-(4-(pyrrolidin-1-ylsulfonyl)phenyl)-1,3,6,2-dioxazaborocane-4,8-dione
Chemical Formula: $C_{15}H_{19}BN_2O_6S$
Molecular Weight: 366.1972

Yield = 326.3 mg (84%).

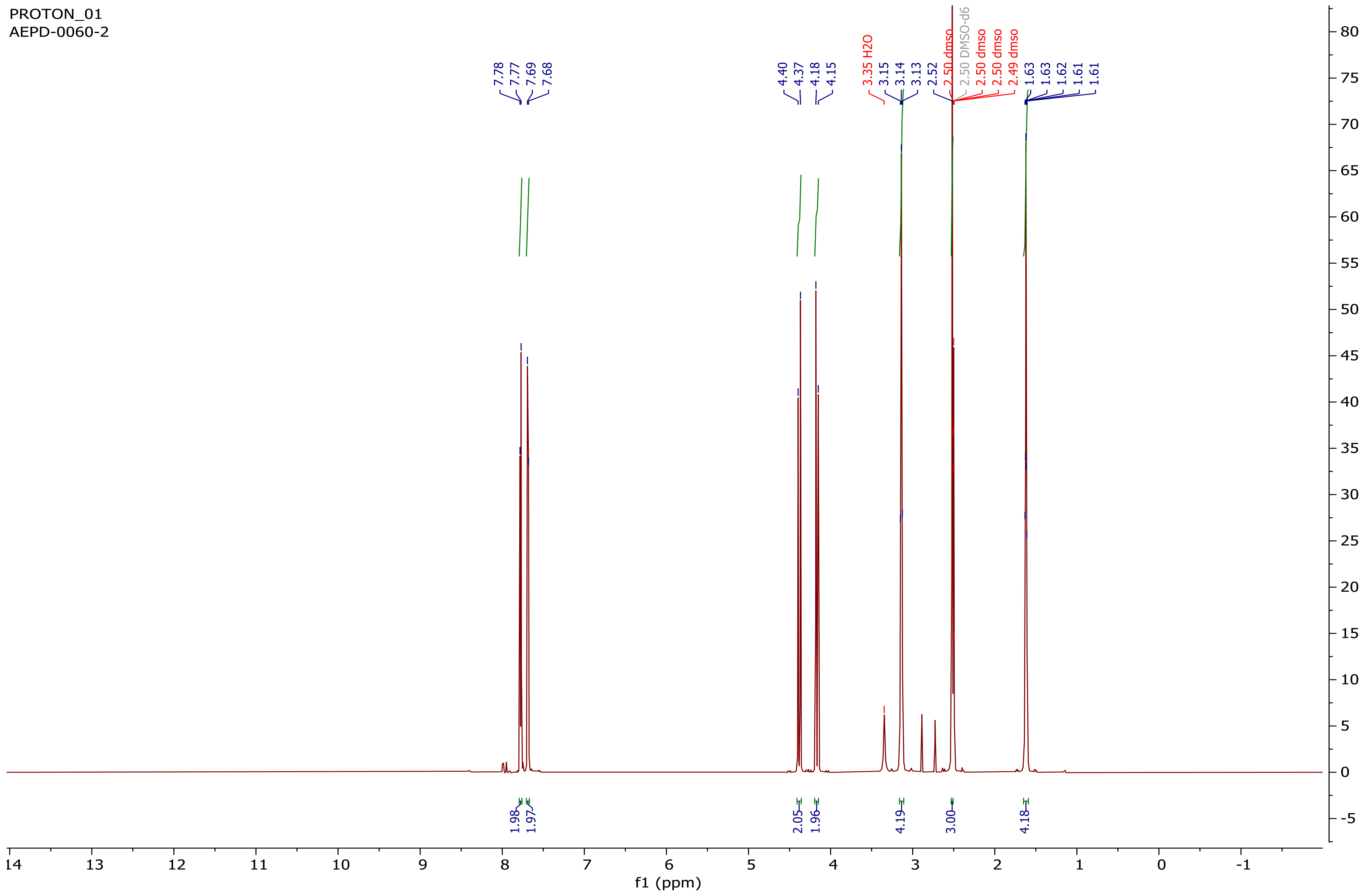

PROTON_01
AEPD-0060-2
7.78
7.77
7.69
7.68
4.40
4.37
4.18
4.15
3.35 H2O
3.15
3.14
3.13
2.52
2.50 dmso
2.50 DMSO-d6
2.50 dmso
2.50 dmso
2.49 dmso
1.63
1.63
1.62
1.61
1.61
1.98
1.97
2.05
1.96
4.19
3.00
4.18
f1 (ppm)

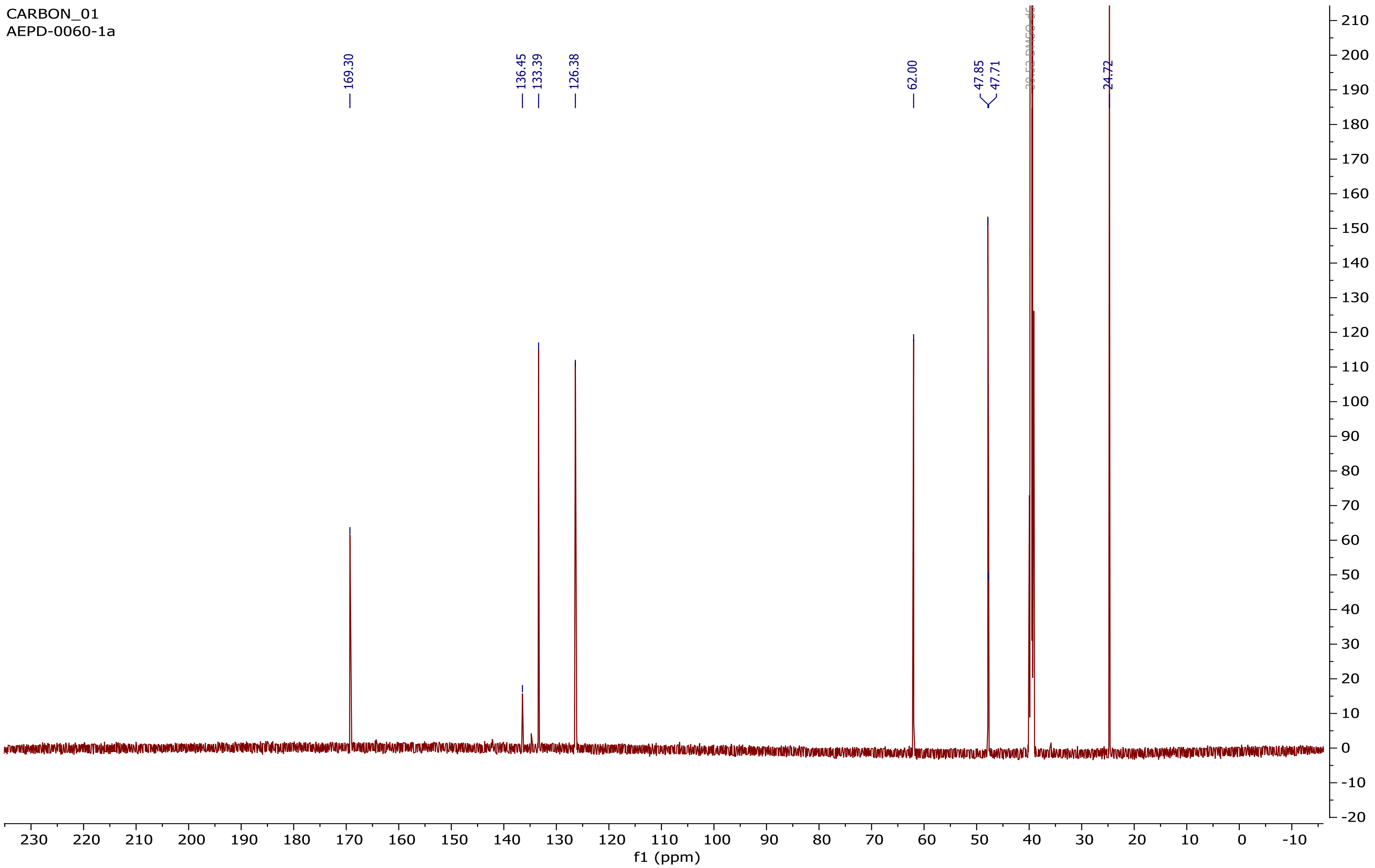
CARBON_01
AEPD-0060-1a
169.30
136.45
133.39
126.38
62.00
47.85
47.71
24.72
f1 (ppm)

Single Mass Analysis
Tolerance = 5.0 mDa / DBE: min = -1.5, max = 120.0
Element prediction: Off
Number of isotope peaks used for i-FIT = 3
Monoisotopic Mass, Even Electron Ions
81 formula(e) evaluated with 1 results within limits (up to 20 best isotopic matches for each mass)
Elements Used:

Mass	Calc. Mass	mDa	PPM	DBE	Formula	i-FIT	i-FIT Norm	Fit Conf %	C	H	11B	N	O	S
367.1129	367.1135	-0.6	-1.6	7.5	C15 H20 11B N2 O6 S	239.2	n/a	n/a	15	20	1	2	6	1

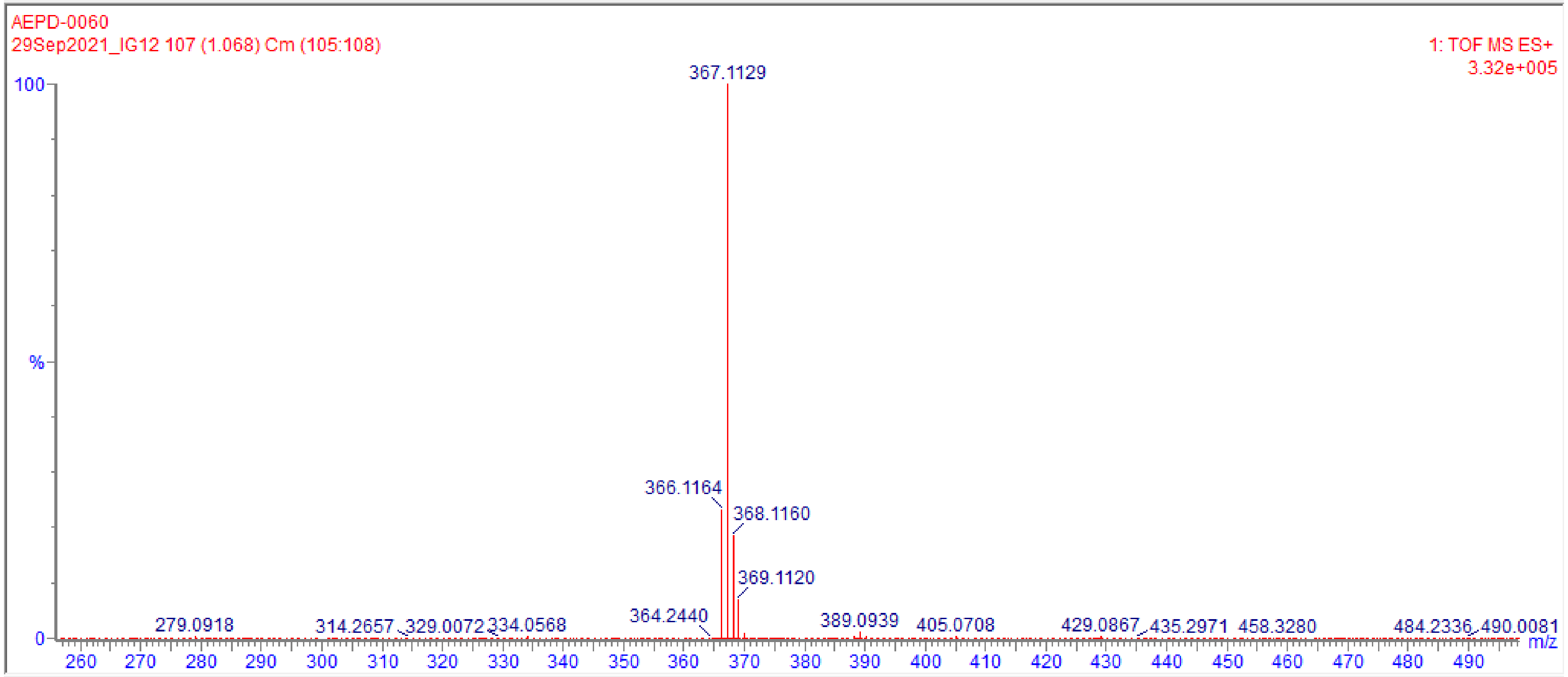

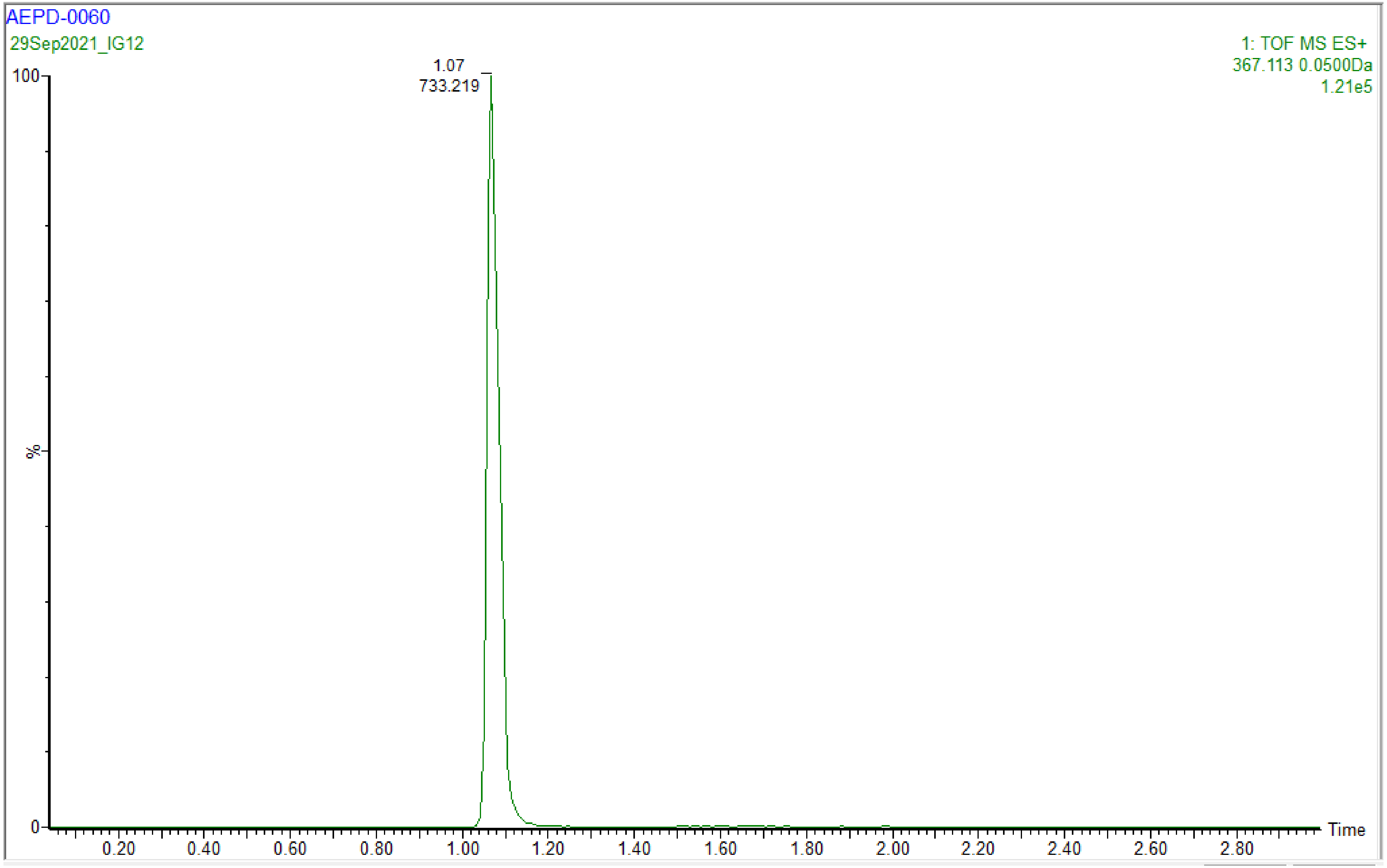
AEPD-0060
29Sep2021_IG12
1: TOF MS ES+
367.113 0.0500Da
1.21e5
1.07
733.219
100
%
0
Time
0.20
0.40
0.60
0.80
1.00
1.20
1.40
1.60
1.80
2.00
2.20
2.40
2.60
2.80

2-(3-(tert-Butyl)phenyl)-6-methyl-1,3,6,2-dioxazaborocane-4,8-dione 8o

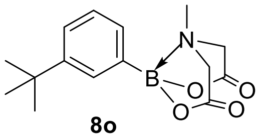

8o

2-(3-(*tert*-butyl)phenyl)-6-methyl-1,3,6,2-dioxazaborocane-4,8-dione
Chemical Formula: $C_{15}H_{20}BNO_4$
Molecular Weight: 289.1346

Yield = 230.0 mg (75%).

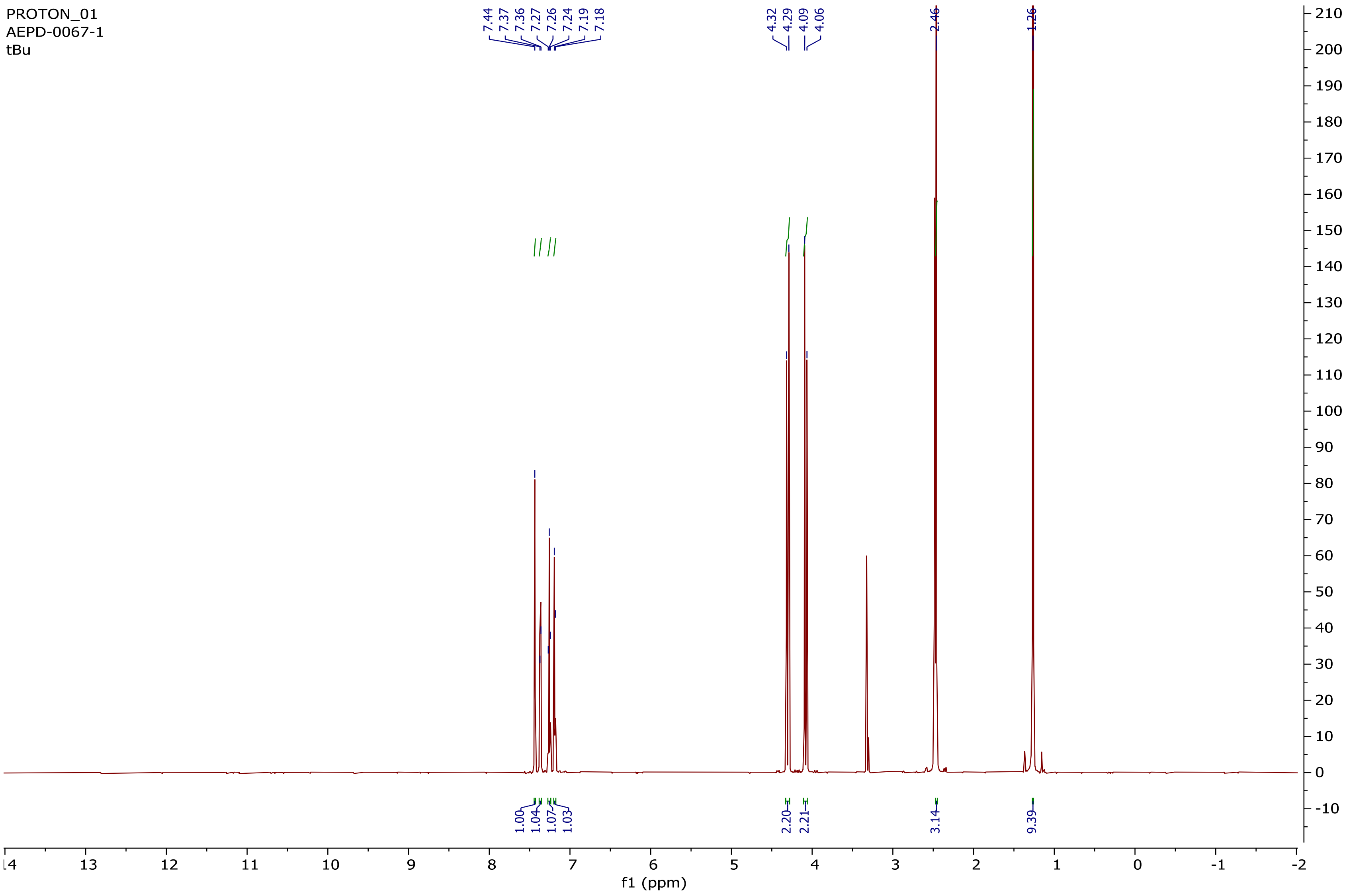
PROTON_01
AEPD-0067-1
tBu
7.44
7.37
7.36
7.27
7.26
7.24
7.19
7.18
4.32
4.29
4.09
4.06
2.46
1.26
1.00
1.04
1.07
1.03
2.20
2.21
3.14
9.39
f1 (ppm)

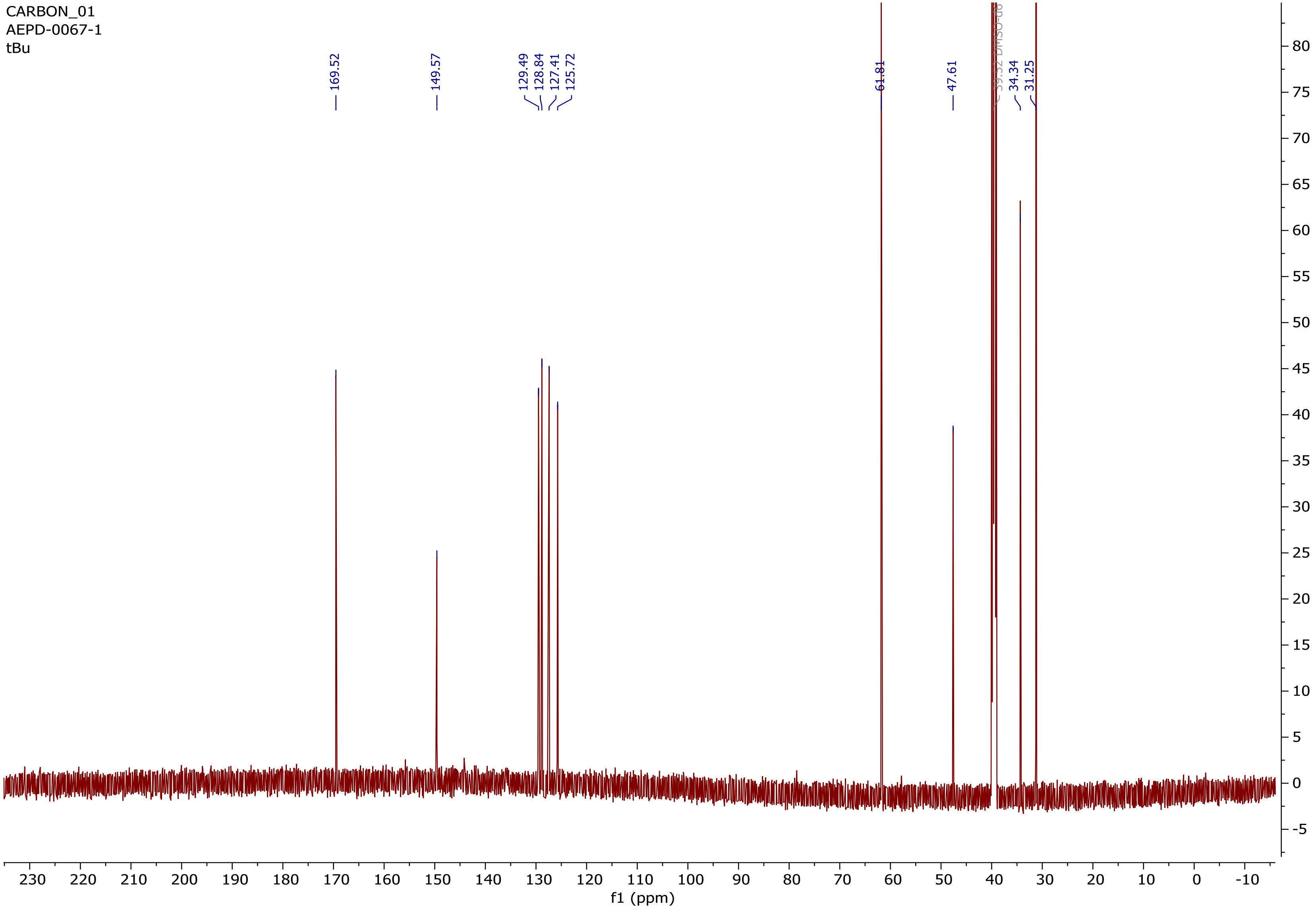

CARBON_01
AEPD-0067-1
tBu
169.52
149.57
129.49
128.84
127.41
125.72
61.81
47.61
39.52 DMSO-d6
34.34
31.25
f1 (ppm)

Single Mass Analysis

Tolerance = 5.0 mDa / DBE: min = -1.5, max = 120.0

Element prediction: Off

Number of isotope peaks used for i-FIT = 3

Monoisotopic Mass, Odd and Even Electron Ions

47 formula(e) evaluated with 1 results within limits (up to 20 best isotopic matches for each mass)

Elements Used:

Mass	Calc. Mass	mDa	PPM	DBE	Formula	i-FIT	i-FIT Norm	Fit Conf %	C	H	11B	N	O
307.1837	307.1829	0.8	2.6	5.5	C15 H24 11B N2 O4	243.6	n/a	n/a	15	24	1	2	4

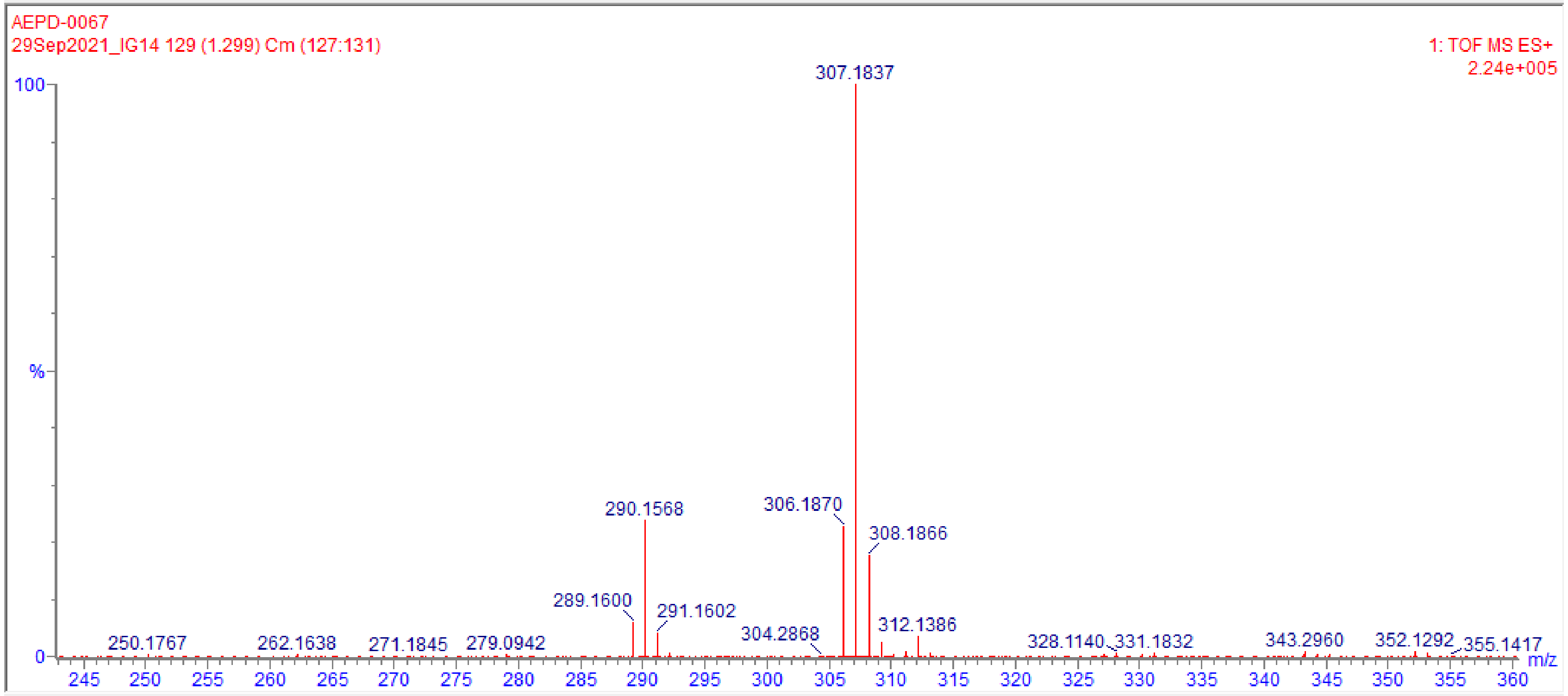

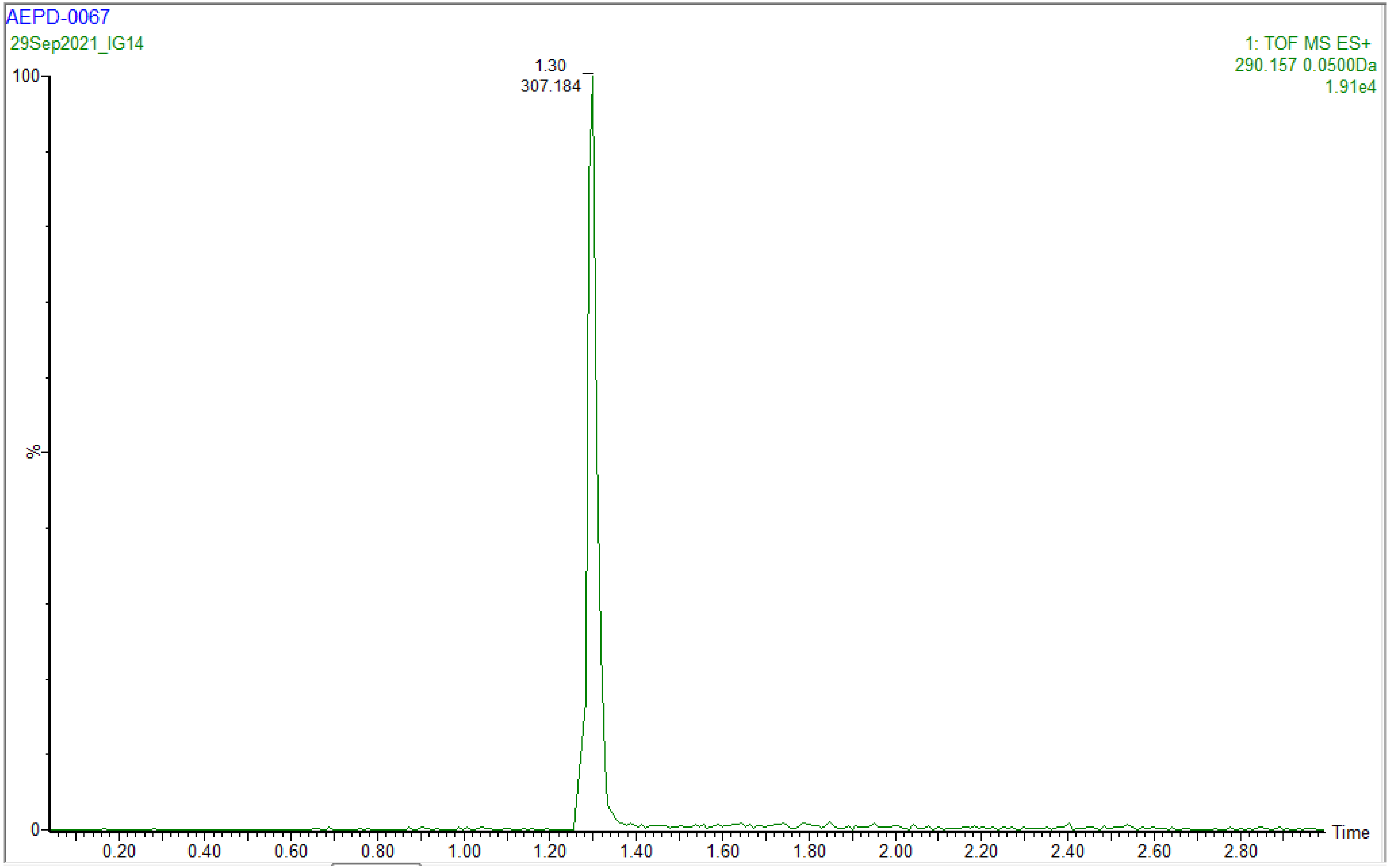
AEPD-0067
29Sep2021_IG14
1: TOF MS ES+
290.157 0.0500Da
1.91e4
1.30
307.184
100
%
0
0.20
0.40
0.60
0.80
1.00
1.20
1.40
1.60
1.80
2.00
2.20
2.40
2.60
2.80
Time

2-(3-(2-Methoxyethoxy)phenyl)-6-methyl-1,3,6,2-dioxazaborocane-4,8-dione 8p

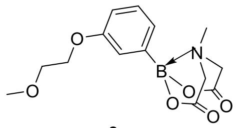

8p

2-(3-(2-methoxyethoxy)phenyl)-6-methyl-1,3,6,2-dioxazaborocane-4,8-dione
Chemical Formula: $C_{14}H_{18}BNO_6$
Molecular Weight: 307.1068

Yield = 199.2 mg (61%).

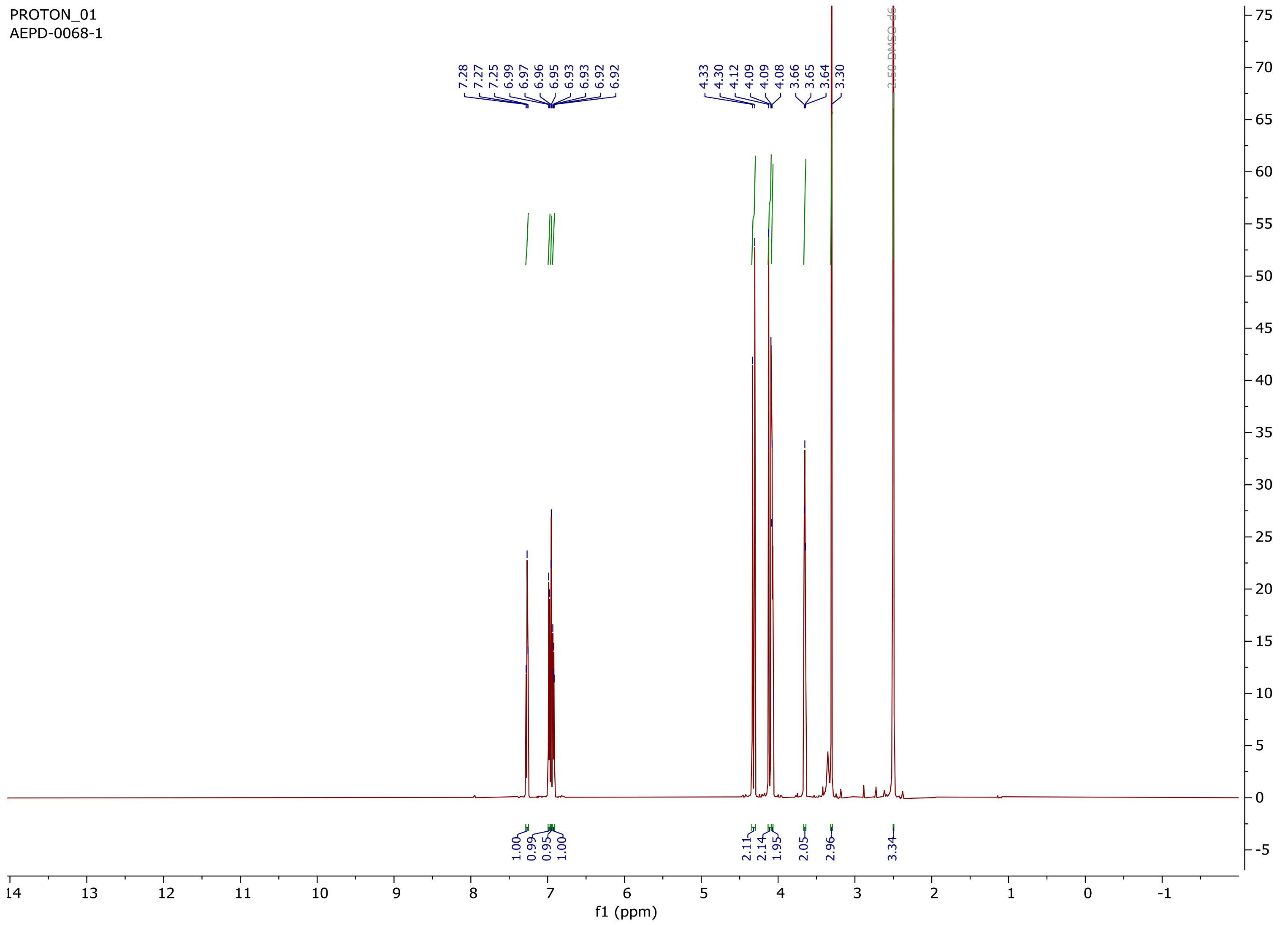
PROTON_01
AEPD-0068-1
7.28
7.27
7.25
6.99
6.97
6.96
6.95
6.93
6.93
6.92
6.92
4.33
4.30
4.12
4.09
4.09
4.08
3.66
3.65
3.64
3.30
2.50 DMSO-d6
1.00
0.99
0.95
1.00
2.11
2.14
1.95
2.05
2.96
3.34
f1 (ppm)

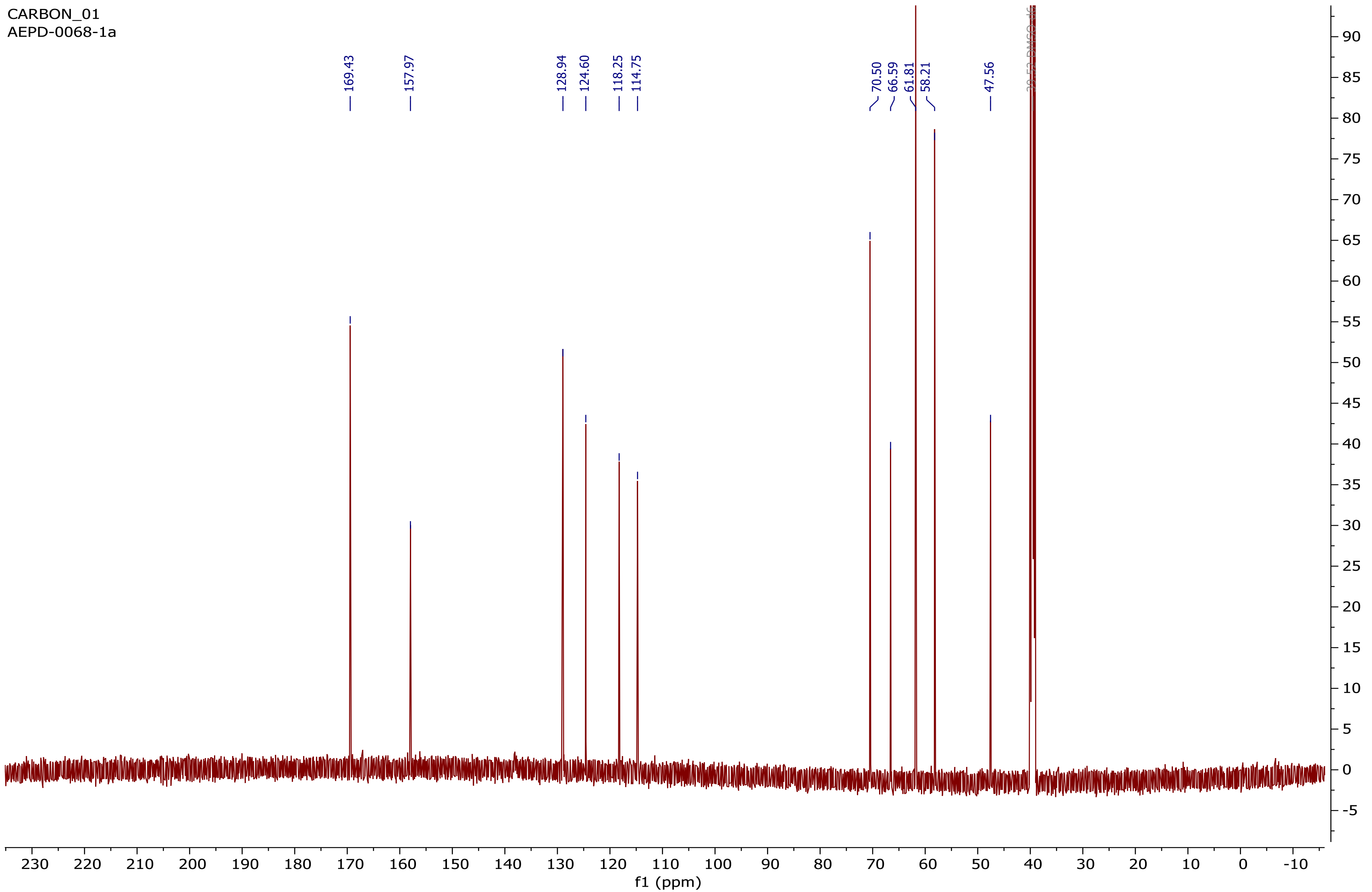

CARBON_01
AEPD-0068-1a
169.43
157.97
128.94
124.60
118.25
114.75
70.50
66.59
61.81
58.21
47.56
f1 (ppm)

Single Mass Analysis
Tolerance = 5.0 mDa / DBE: min = -1.5, max = 120.0
Element prediction: Off
Number of isotope peaks used for i-FIT = 3
Monoisotopic Mass, Even Electron Ions
63 formula(e) evaluated with 1 results within limits (up to 20 best isotopic matches for each mass)
Elements Used:

Mass	Calc. Mass	mDa	PPM	DBE	Formula	i-FIT	i-FIT Norm	Fit Conf %	C	H	11B	N	O
308.1305	308.1305	0.0	0.0	6.5	C14 H19 11B N O6	302.1	n/a	n/a	14	19	1	1	6

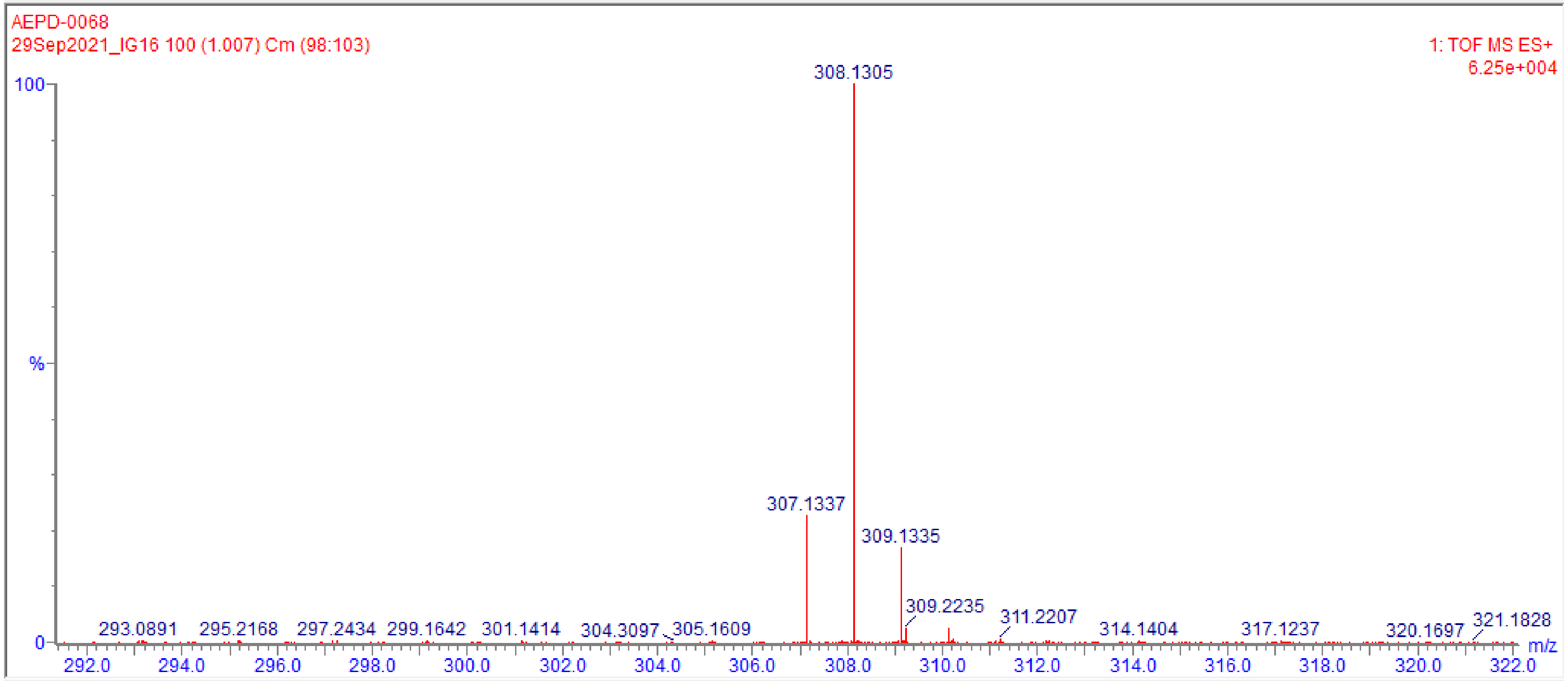

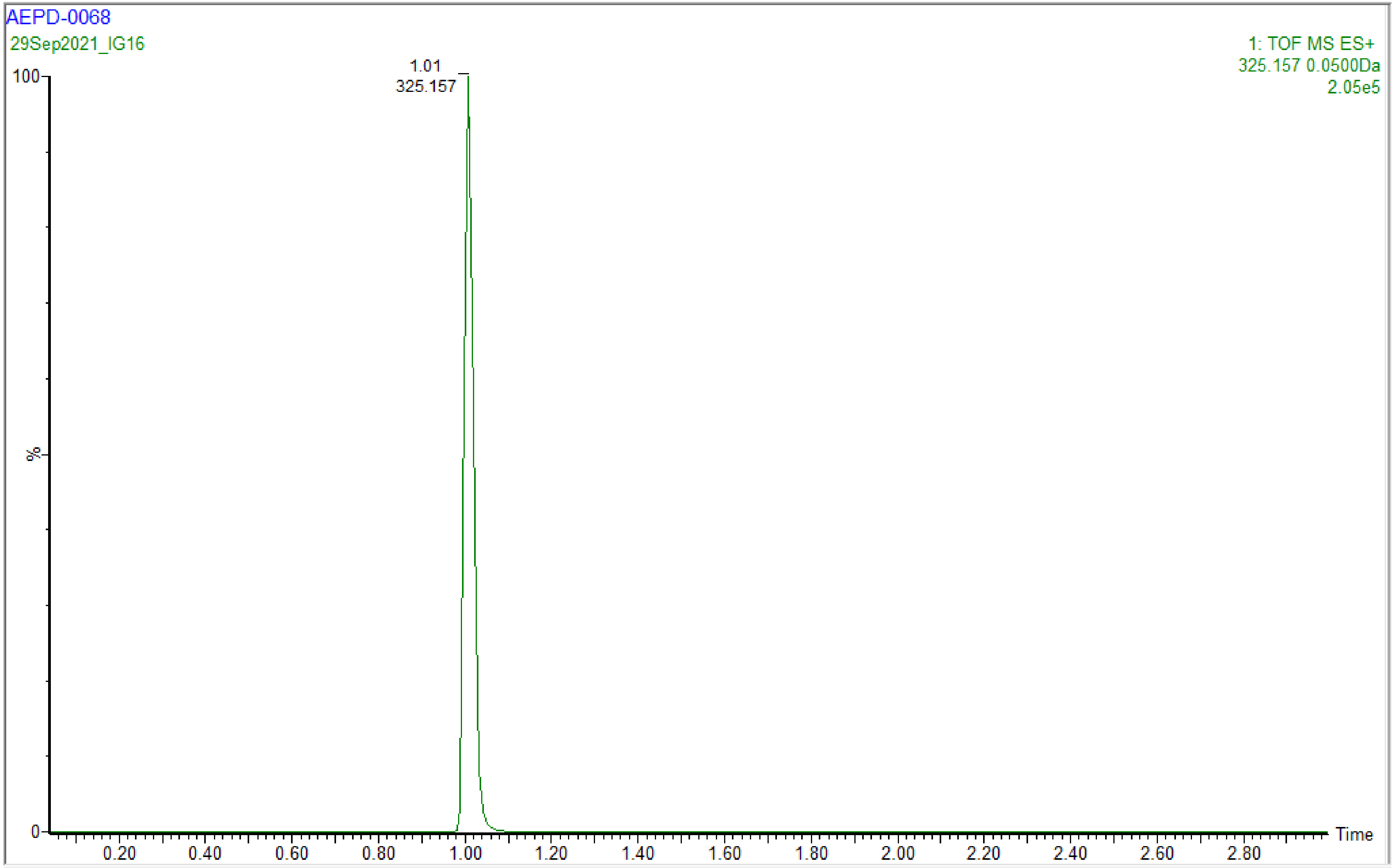
AEPD-0068
29Sep2021_IG16
1: TOF MS ES+
325.157 0.0500Da
2.05e5
1.01
325.157
100
%
0
0.20
0.40
0.60
0.80
1.00
1.20
1.40
1.60
1.80
2.00
2.20
2.40
2.60
2.80
Time

2-(3,5-Dimethylisoxazol-4-yl)-6-methyl-1,3,6,2-dioxazaborocane-4,8-dione 9

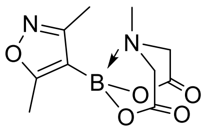

9

2-(3,5-dimethylisoxazol-4-yl)-6-methyl-1,3,6,2-dioxazaborocane-4,8-dione
Chemical Formula: $C_{10}H_{13}BN_2O_5$
Molecular Weight: 252.03

Yield = 154.0 mg (57%).

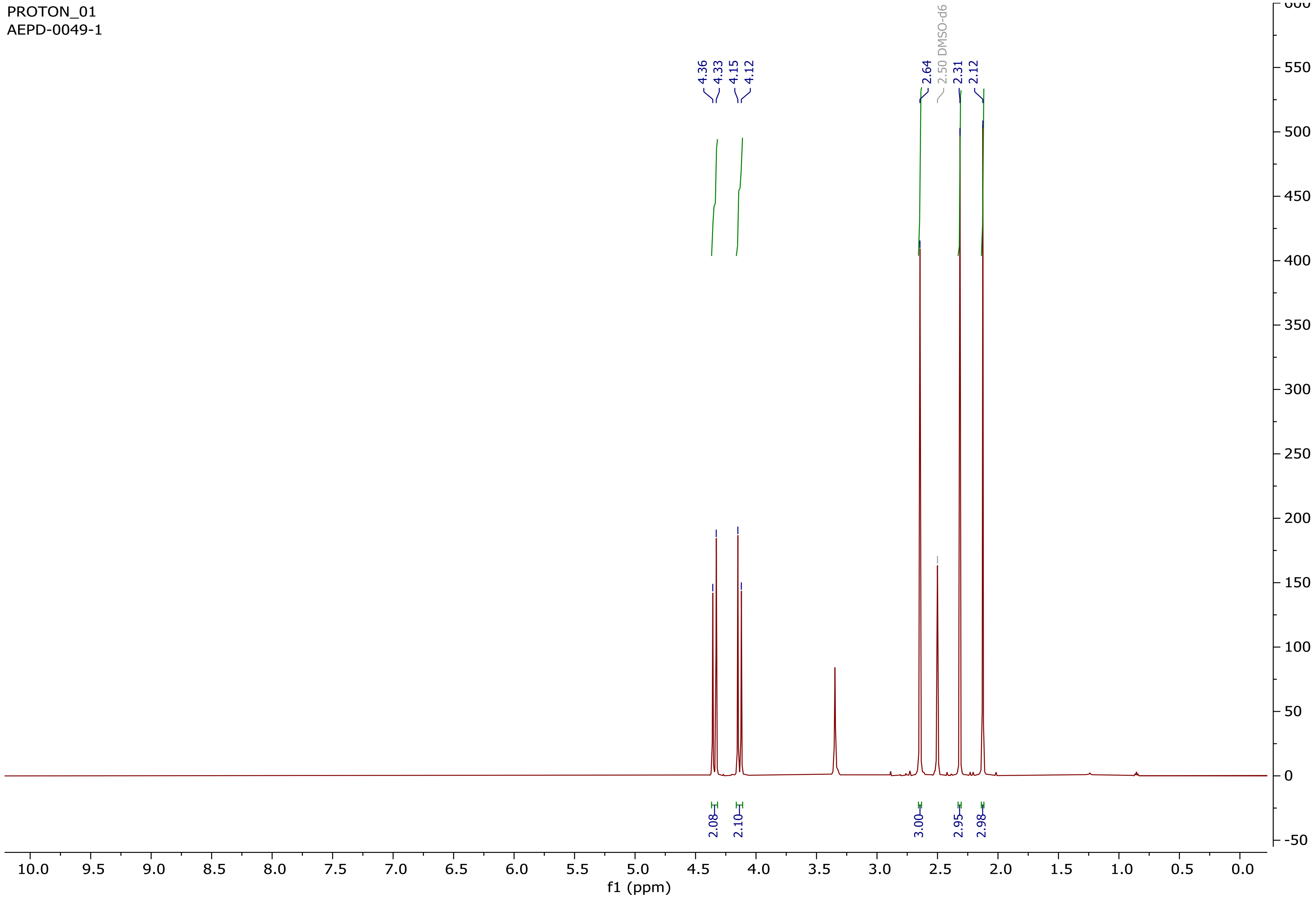
PROTON_01
AEPD-0049-1
4.36
4.33
4.15
4.12
2.64
2.50 DMSO-d6
2.31
2.12
2.08
2.10
3.00
2.95
2.98
f1 (ppm)

CARBON_01
AEPD-0049-1

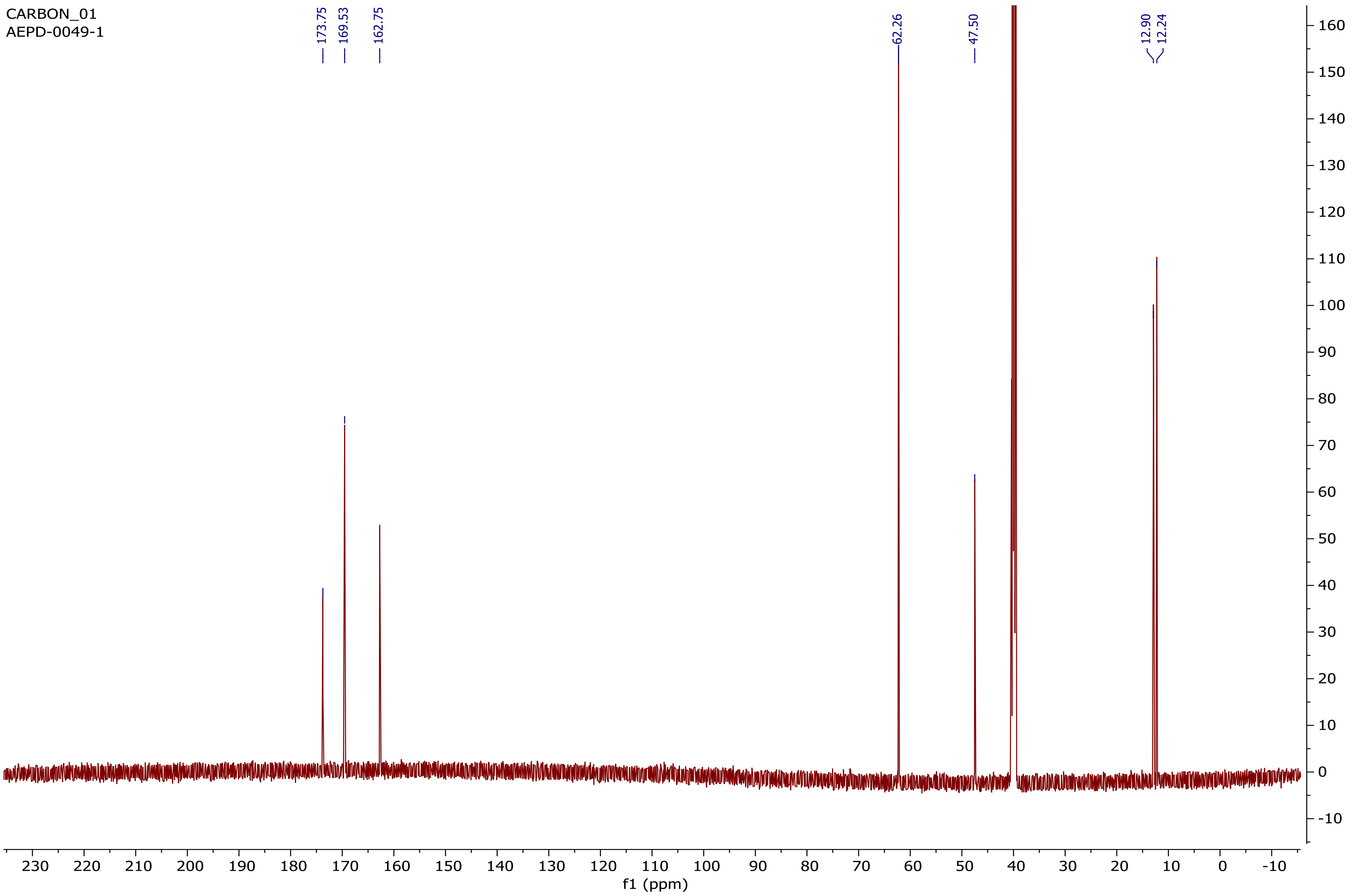

Single Mass Analysis

Tolerance = 5.0 mDa / DBE: min = -1.5, max = 120.0
Element prediction: Off
Number of isotope peaks used for i-FIT = 3
Monoisotopic Mass, Even Electron Ions
22 formula(e) evaluated with 1 results within limits (up to 20 best isotopic matches for each mass)
Elements Used:

Mass	Calc. Mass	mDa	PPM	DBE	Formula	i-FIT	i-FIT Norm	Fit Conf %	C	H	11B	N	O
253.1007	253.0996	1.1	4.3	5.5	C10 H14 11B N2 O5	67.3	n/a	n/a	10	14	1	2	5

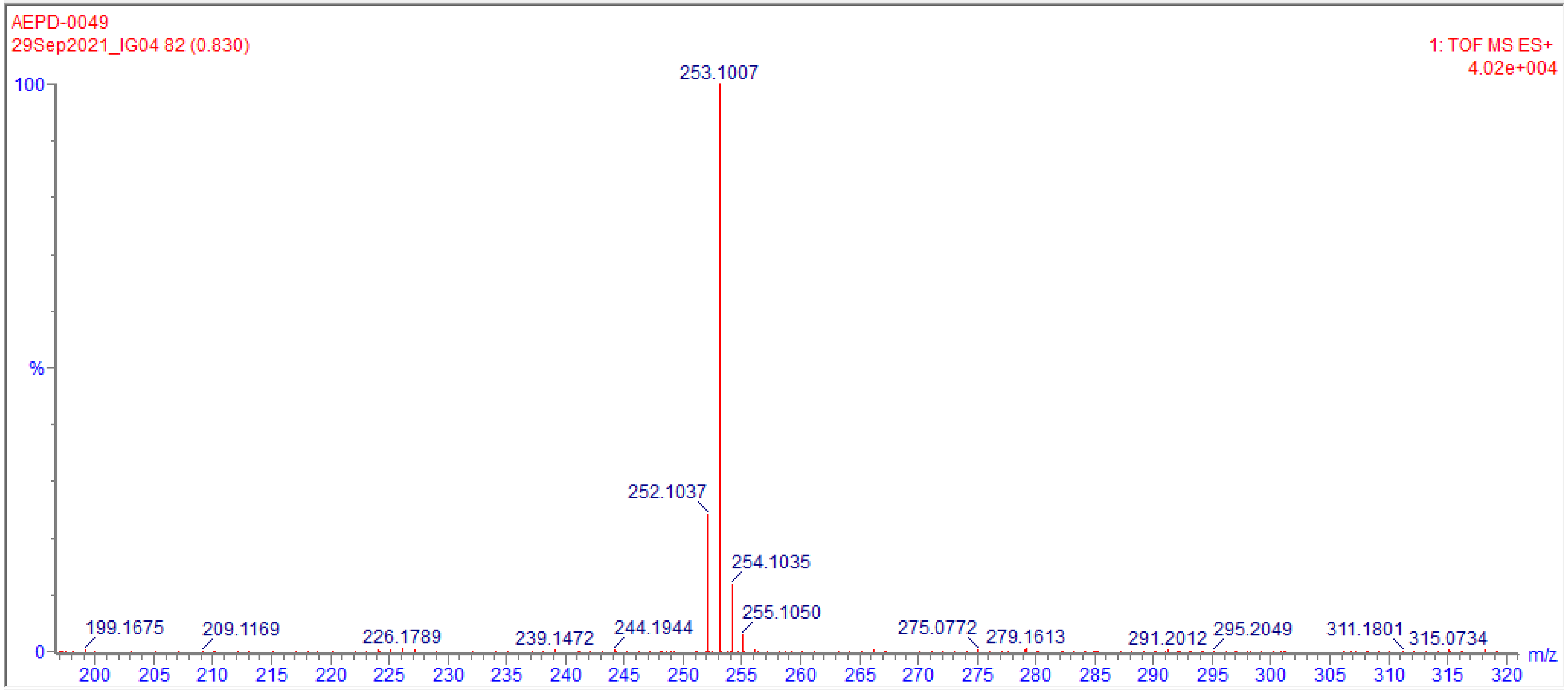

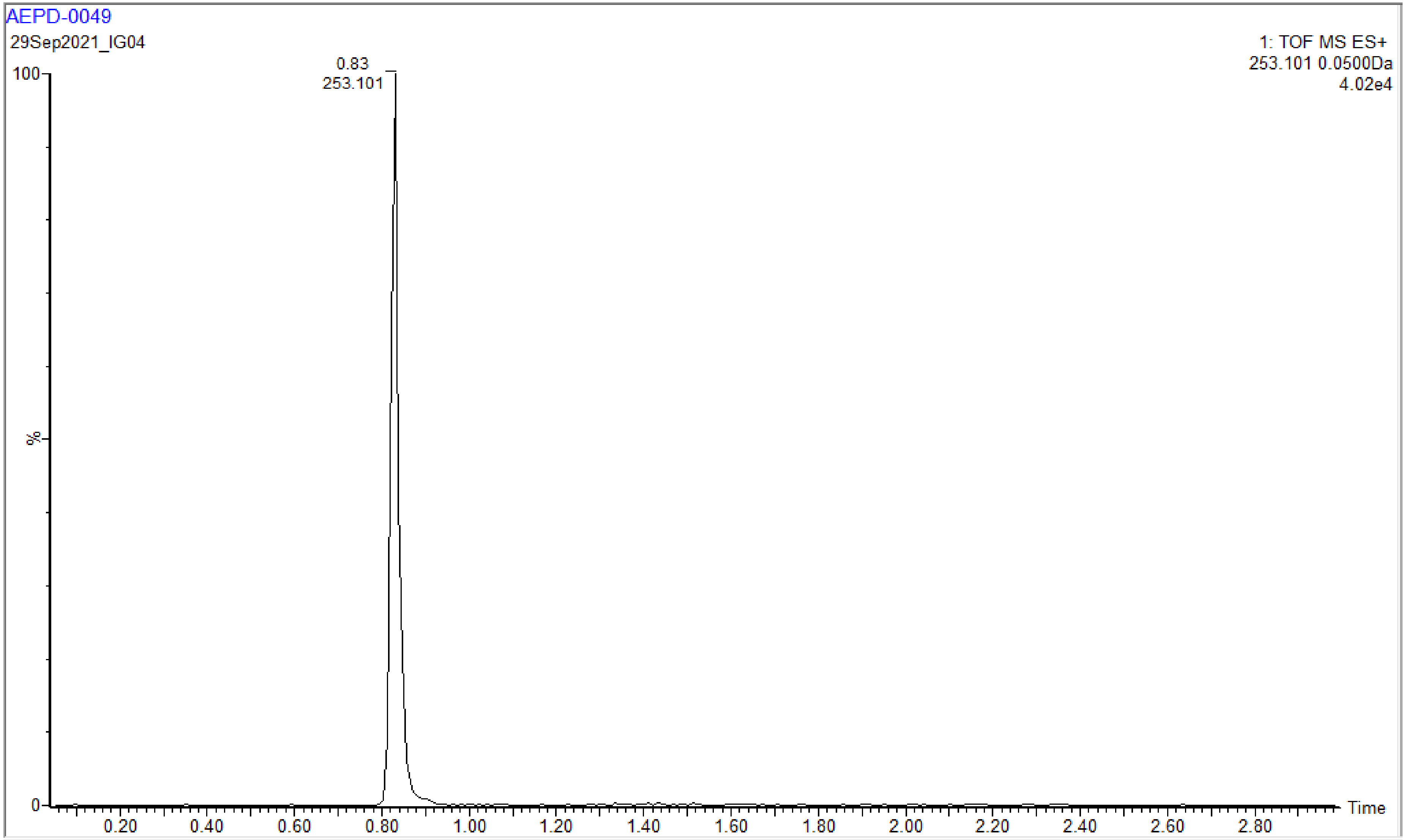
AEPD-0049
29Sep2021_IG04
1: TOF MS ES+
253.101 0.0500Da
4.02e4
0.83
253.101
100
%
0
Time
0.20
0.40
0.60
0.80
1.00
1.20
1.40
1.60
1.80
2.00
2.20
2.40
2.60
2.80

6-Methyl-2-propyl-1,3,6,2-dioxazaborocane-4,8-dione 10

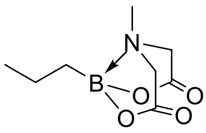

6-methyl-2-propyl-1,3,6,2-dioxazaborocane-4,8-dione
Chemical Formula: $C_8H_{14}BNO_4$
Molecular Weight: 199.0121

Yield = 34.4 mg (17%).

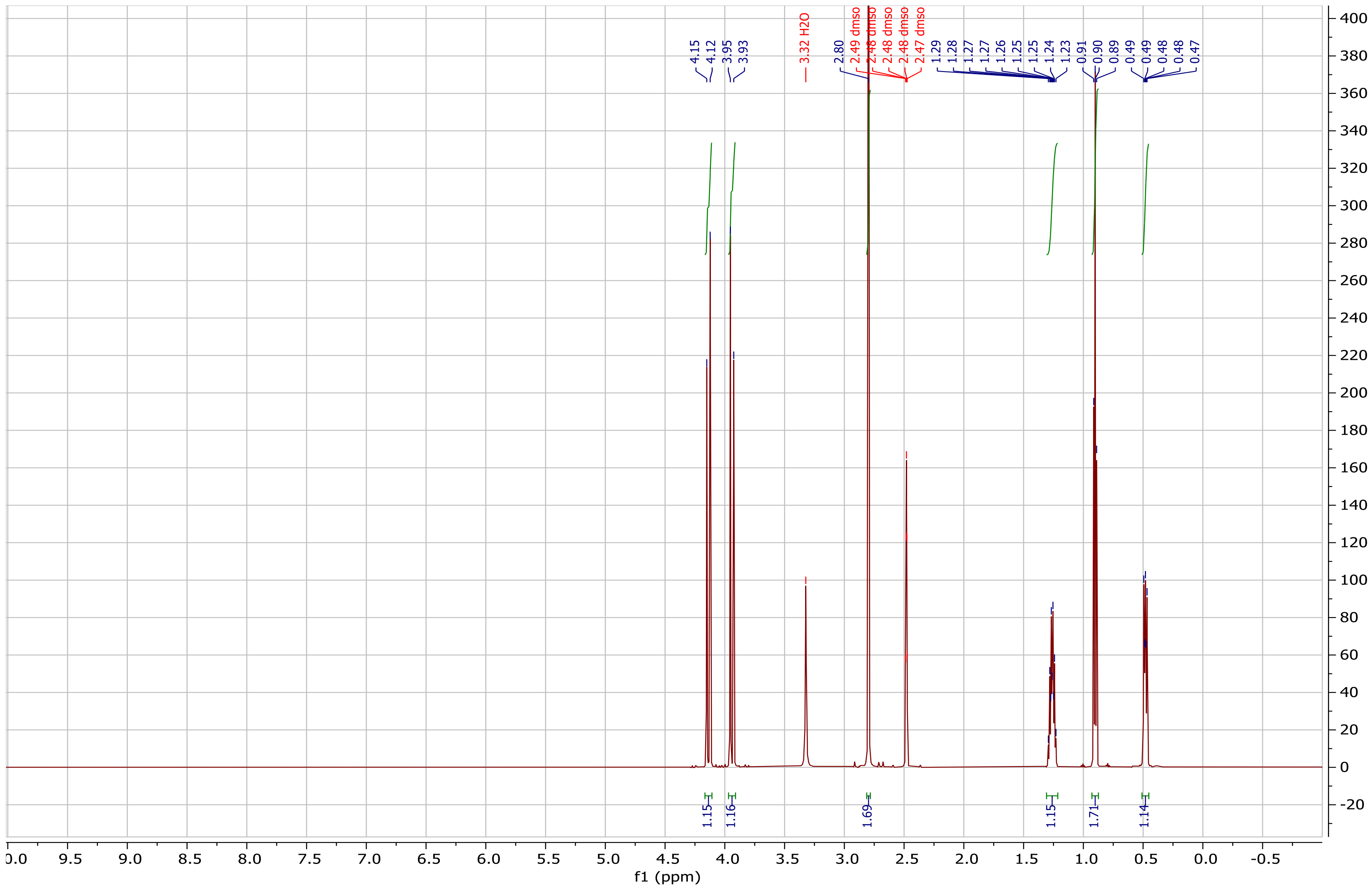
4.15
4.12
3.95
3.93
3.32 H2O
2.80
2.49 dmso
2.48 dmso
2.48 dmso
2.48 dmso
2.47 dmso
1.29
1.28
1.27
1.27
1.26
1.25
1.25
1.24
1.23
0.91
0.90
0.89
0.49
0.49
0.48
0.48
0.47
1.15
1.16
1.69
1.15
1.71
1.14
f1 (ppm)

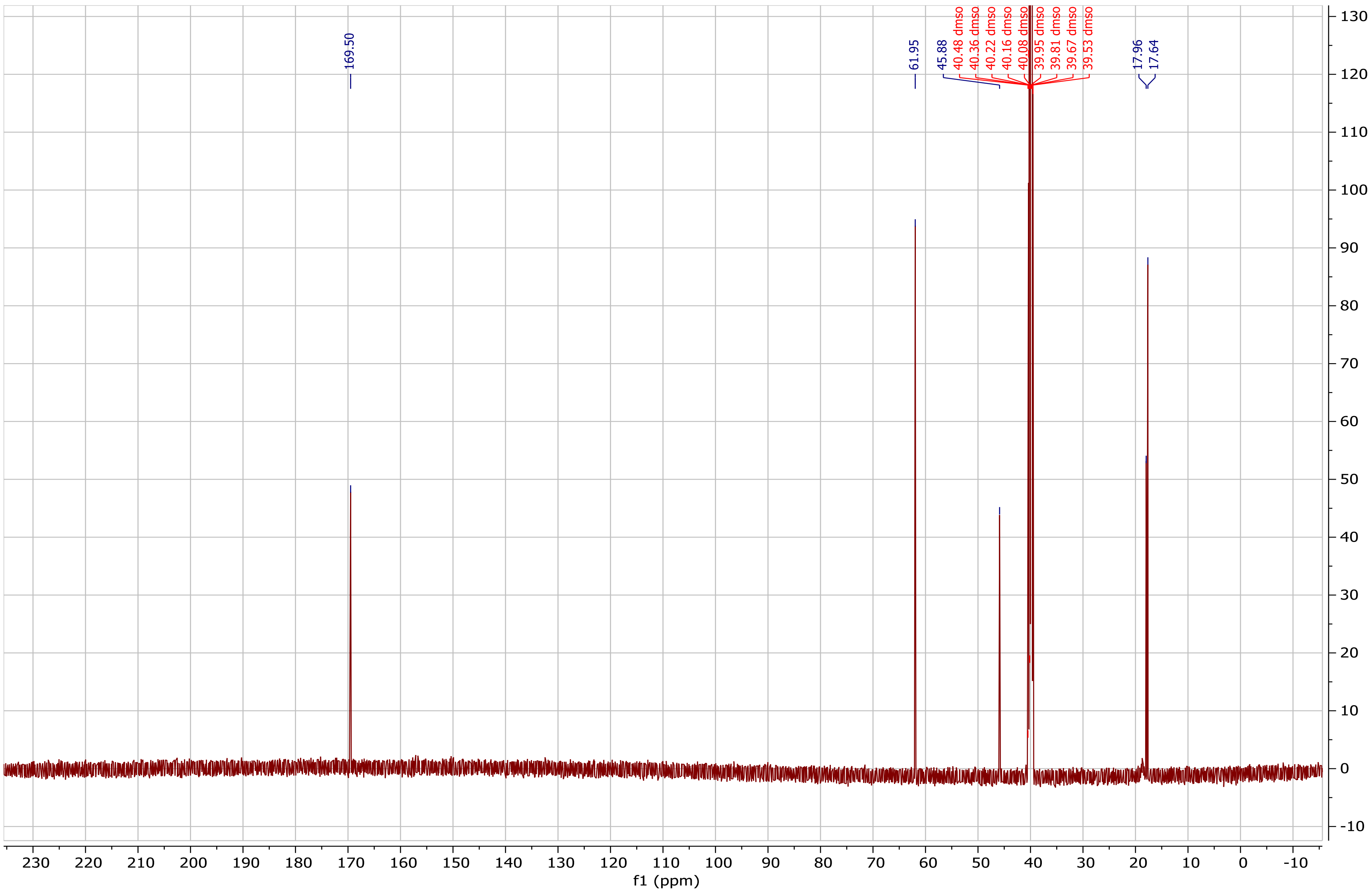

169.50
61.95
45.88
40.48 dmso
40.36 dmso
40.22 dmso
40.16 dmso
40.08 dmso
39.95 dmso
39.81 dmso
39.67 dmso
39.53 dmso
17.96
17.64
f1 (ppm)

Single Mass Analysis
Tolerance = 5.0 mDa / DBE: min = -1.5, max = 120.0
Element prediction: Off
Number of isotope peaks used for i-FIT = 3
Monoisotopic Mass, Even Electron Ions
28 formula(e) evaluated with 1 results within limits (up to 20 best isotopic matches for each mass)
Elements Used:

Mass	Calc. Mass	mDa	PPM	DBE	Formula	i-FIT	i-FIT Norm	Fit Conf %	C	H	11B	N	O
200.1094	200.1094	0.0	0.0	2.5	C8 H15 11B N O4	92.3	n/a	n/a	8	15	1	1	4

INTER043
29Sep2021_IG32 92 (0.937) Cm (91:95)

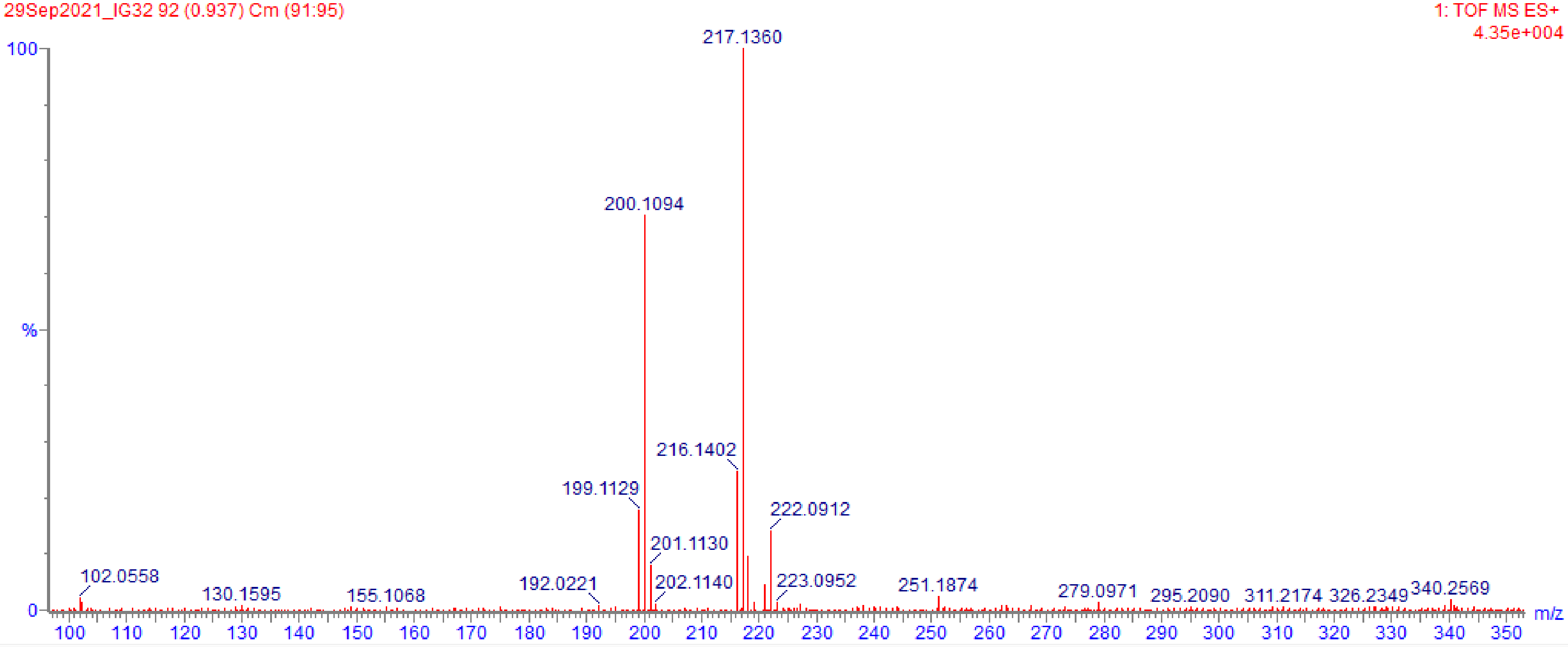

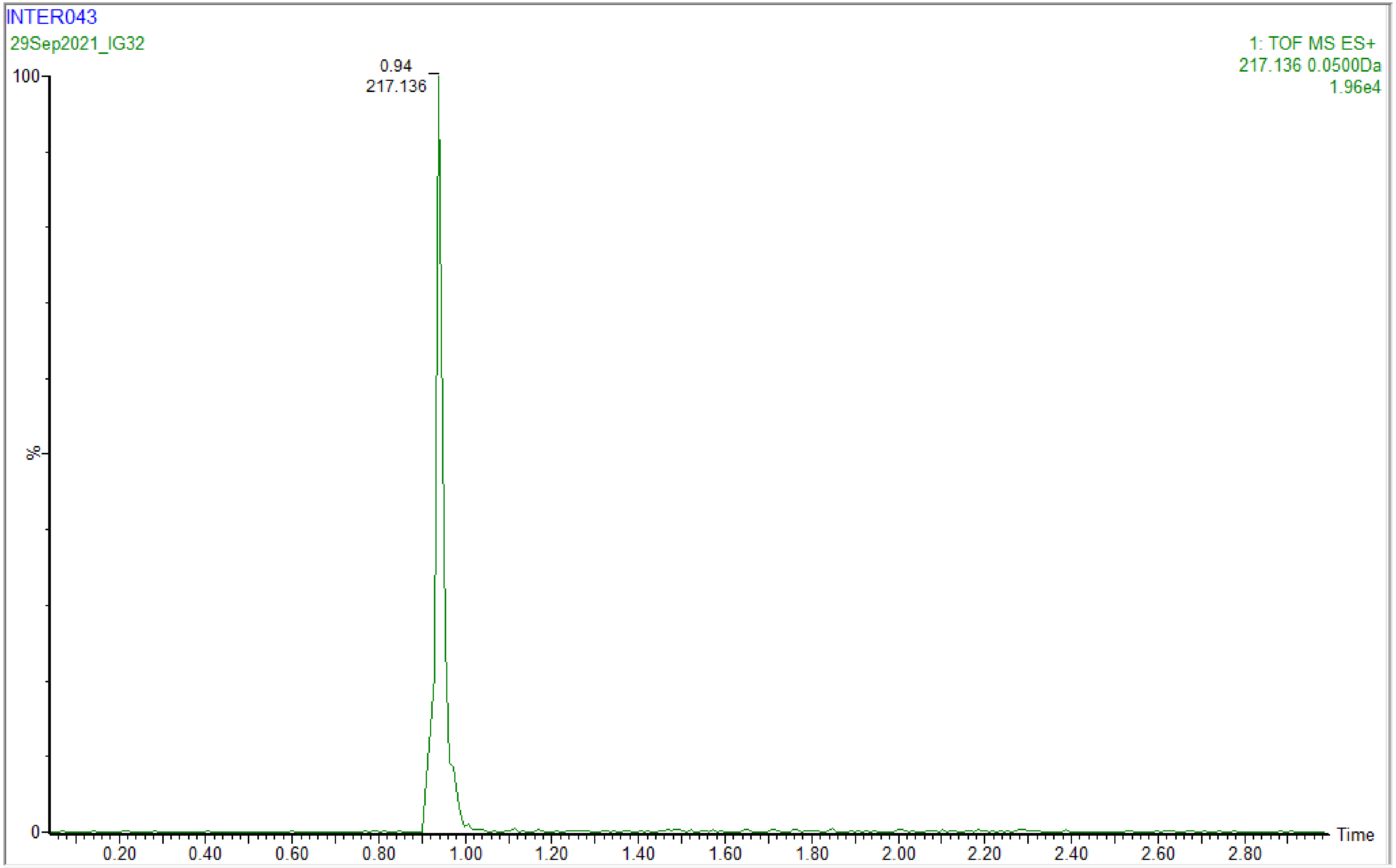

INTER043
29Sep2021_IG32
1: TOF MS ES+
217.136 0.0500Da
1.96e4
0.94
217.136
100
%
0
0.20
0.40
0.60
0.80
1.00
1.20
1.40
1.60
1.80
2.00
2.20
2.40
2.60
2.80
Time

6-Methyl-2-(1-methyl-1H-benzo[d]imidazol-5-yl)-1,3,6,2-dioxazaborocane-4,8-dione 11

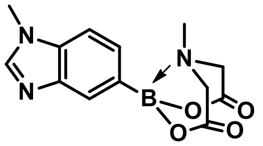

6-methyl-2-(1-methyl-1*H*-benzo[*d*]imidazol-5-yl)-1,3,6,2-dioxazaborocane-4,8-dione
Chemical Formula: $C_{13}H_{14}BN_3O_4$
Molecular Weight: 287.0790

Yield = 161.0 mg (56%).

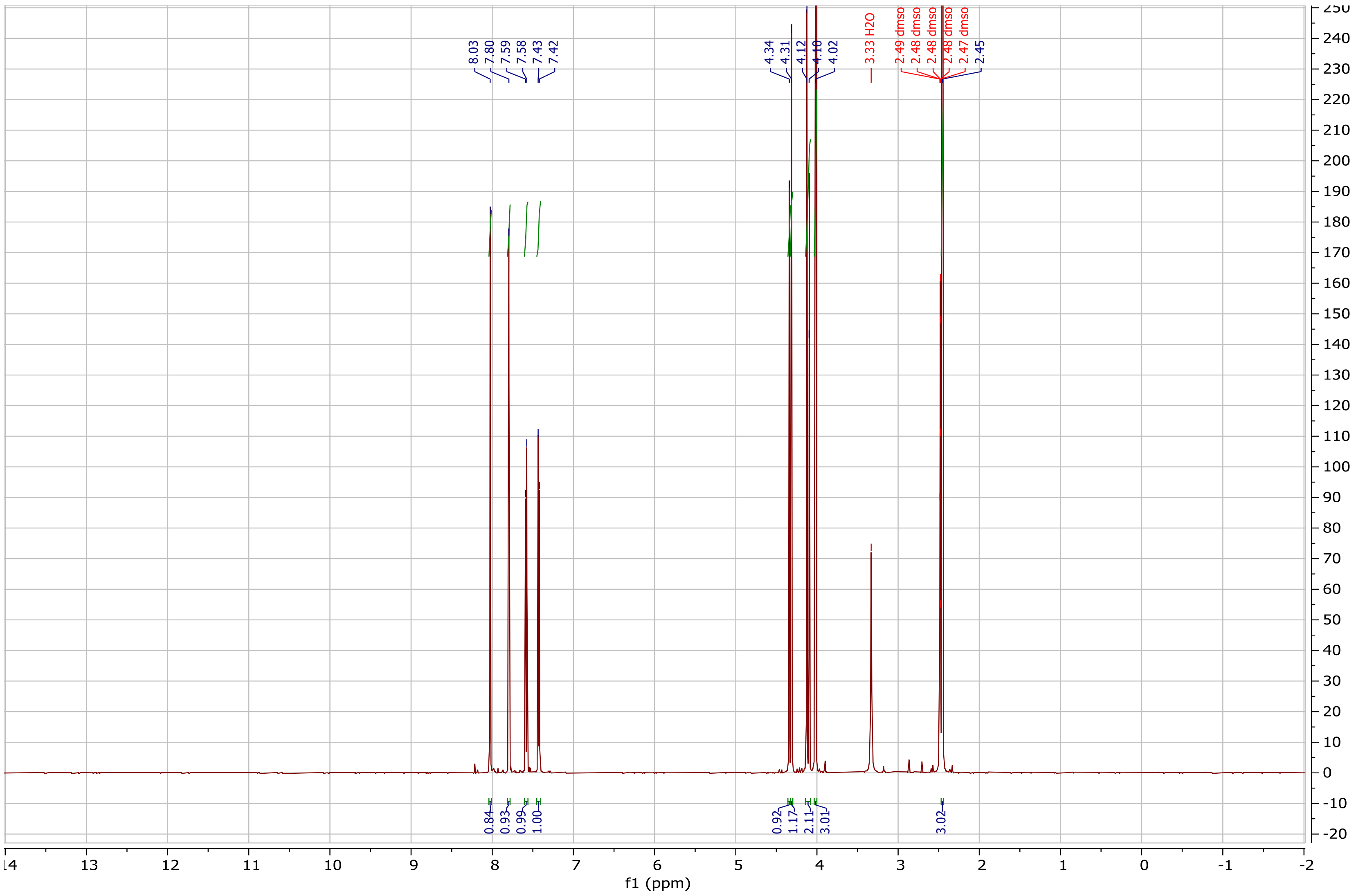

8.03
7.80
7.59
7.58
7.43
7.42
4.34
4.31
4.12
4.10
4.02
3.33 H2O
2.49 dmso
2.48 dmso
2.48 dmso
2.48 dmso
2.47 dmso
2.45
0.84
0.93
0.99
1.00
0.92
1.17
2.11
3.01
3.02
f1 (ppm)

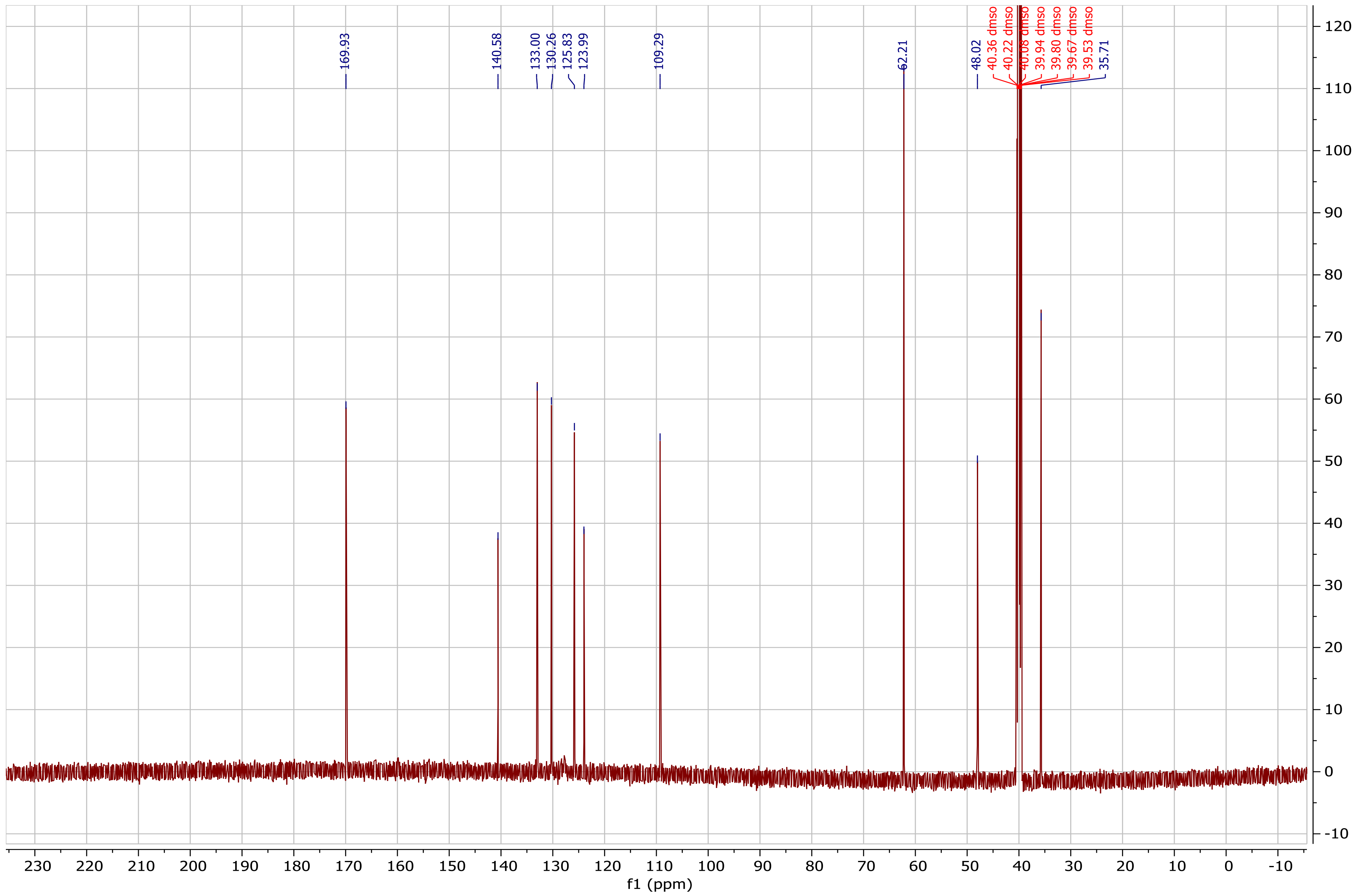
169.93
140.58
133.00
130.26
125.83
123.99
109.29
62.21
48.02
40.36 dmso
40.22 dmso
40.08 dmso
39.94 dmso
39.80 dmso
39.67 dmso
39.53 dmso
35.71
f1 (ppm)

Single Mass Analysis
Tolerance = 5.0 mDa / DBE: min = -1.5, max = 120.0
Element prediction: Off
Number of isotope peaks used for i-FIT = 3
Monoisotopic Mass, Even Electron Ions
36 formula(e) evaluated with 1 results within limits (up to 20 best isotopic matches for each mass)
Elements Used:

Mass	Calc. Mass	mDa	PPM	DBE	Formula	i-FIT	i-FIT Norm	Fit Conf %	C	H	11B	N	O
288.1161	288.1156	0.5	1.7	8.5	C13 H15 11B N3 O4	408.5	n/a	n/a	13	15	1	3	4

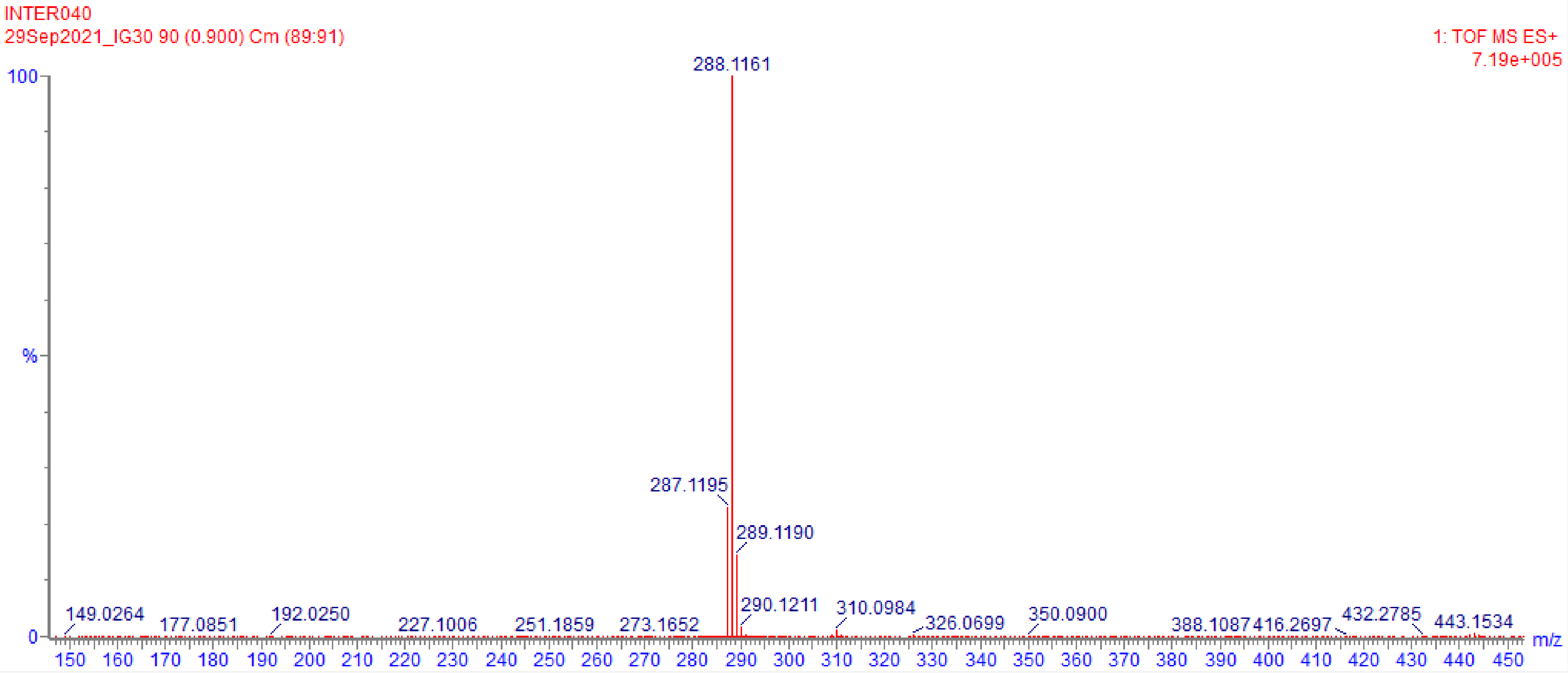

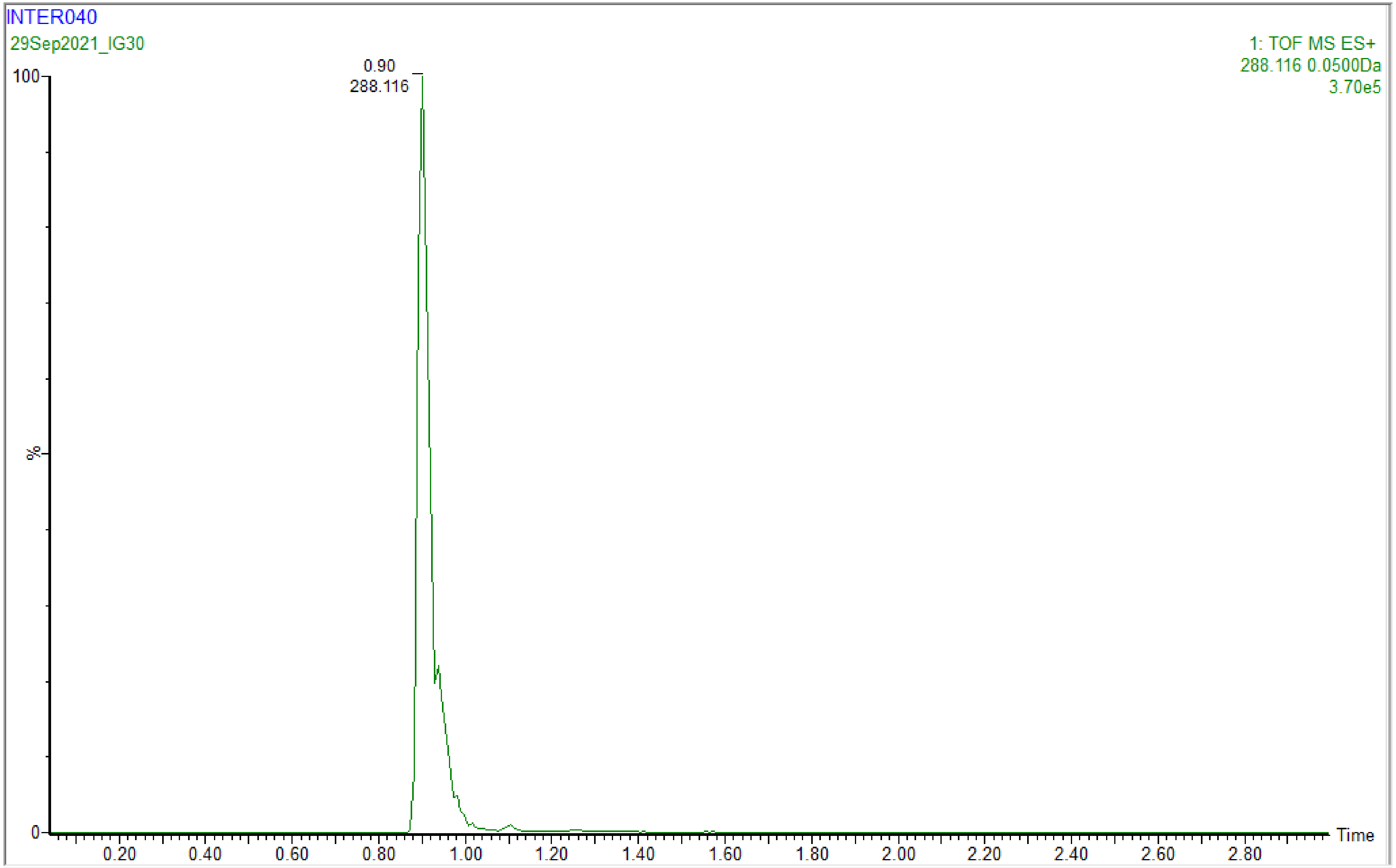

INTER040
29Sep2021_IG30
1: TOF MS ES+
288.116 0.0500Da
3.70e5
0.90
288.116
100
%
0
0.20
0.40
0.60
0.80
1.00
1.20
1.40
1.60
1.80
2.00
2.20
2.40
2.60
2.80
Time

2-(*1H*-Indol-5-yl)-6-methyl-1,3,6,2-dioxazaborocane-4,8-dione 12

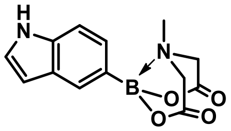

2-(1*H*-indol-5-yl)-6-methyl-1,3,6,2-dioxazaborocane-4,8-dione
Chemical Formula: $C_{13}H_{13}BN_2O_4$
Molecular Weight: 272.0643

Yield = 142.2 mg (52%).

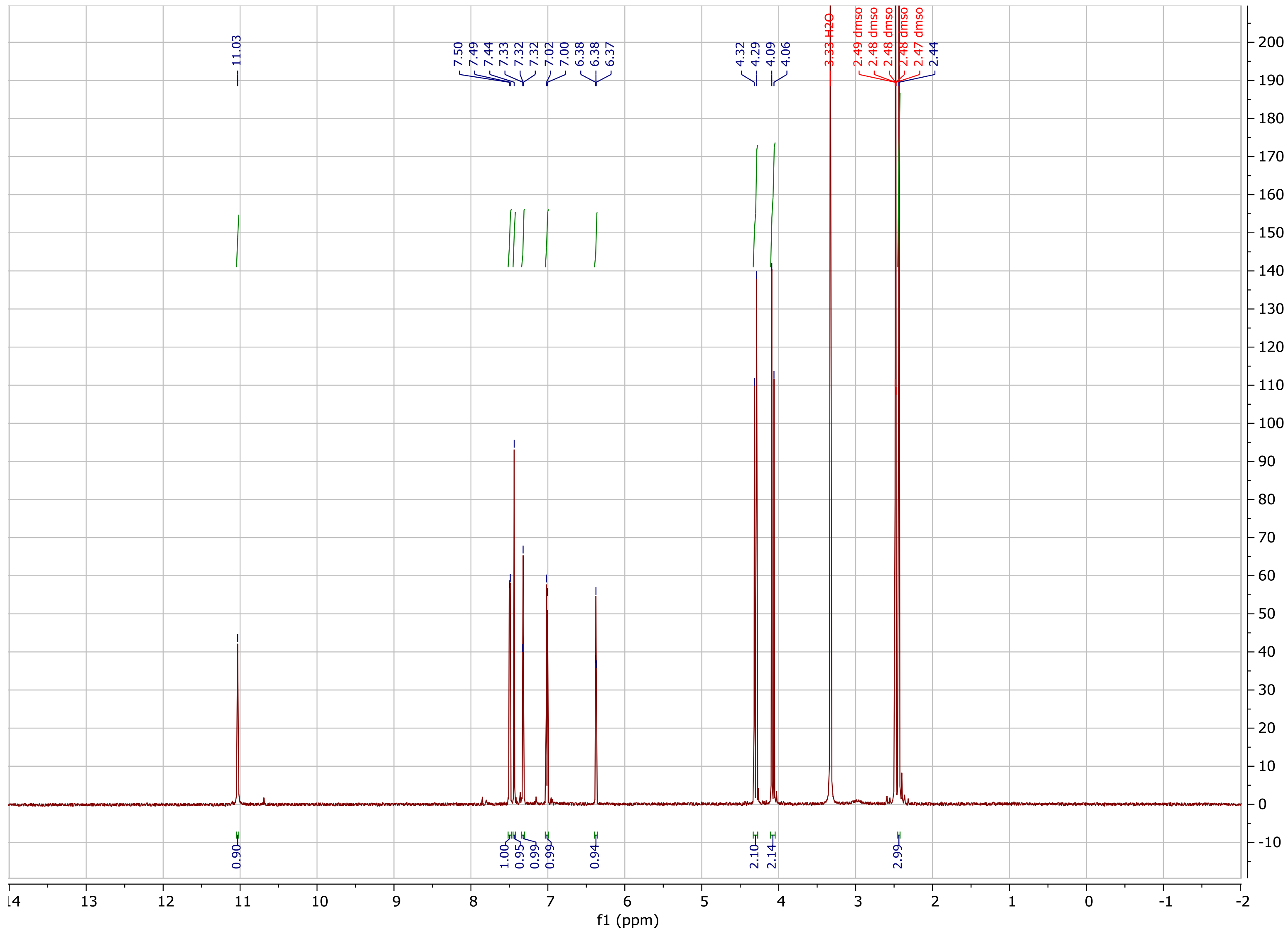
11.03
7.50
7.49
7.44
7.33
7.32
7.32
7.02
7.00
6.38
6.38
6.37
4.32
4.29
4.09
4.06
3.33 H2O
2.49 dmso
2.48 dmso
2.48 dmso
2.48 dmso
2.47 dmso
2.44
0.90
1.00
0.95
0.99
0.99
0.94
2.10
2.14
2.99
f1 (ppm)

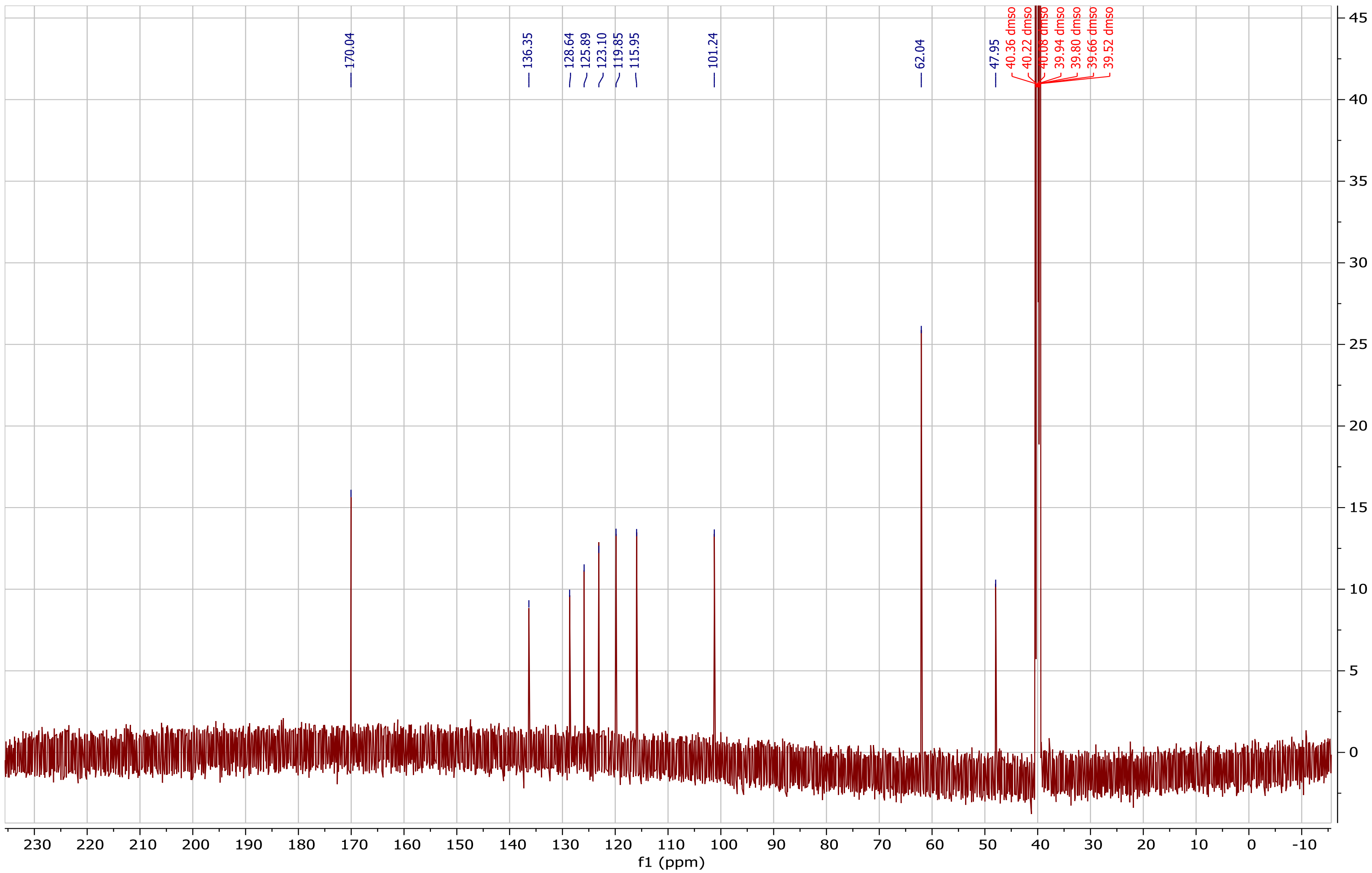
170.04
136.35
128.64
125.89
123.10
119.85
115.95
101.24
62.04
47.95
40.36 dmso
40.22 dmso
40.08 dmso
39.94 dmso
39.80 dmso
39.66 dmso
39.52 dmso
f1 (ppm)

Single Mass Analysis
Tolerance = 5.0 mDa / DBE: min = -1.5, max = 120.0
Element prediction: Off
Number of isotope peaks used for i-FIT = 3
Monoisotopic Mass, Even Electron Ions
22 formula(e) evaluated with 1 results within limits (up to 20 best isotopic matches for each mass)
Elements Used:

Mass	Calc. Mass	mDa	PPM	DBE	Formula	i-FIT	i-FIT Norm	Fit Conf %	C	H	11B	N	O
273.1051	273.1047	0.4	1.5	8.5	C13 H14 11B N2 O4	370.5	n/a	n/a	13	14	1	2	4

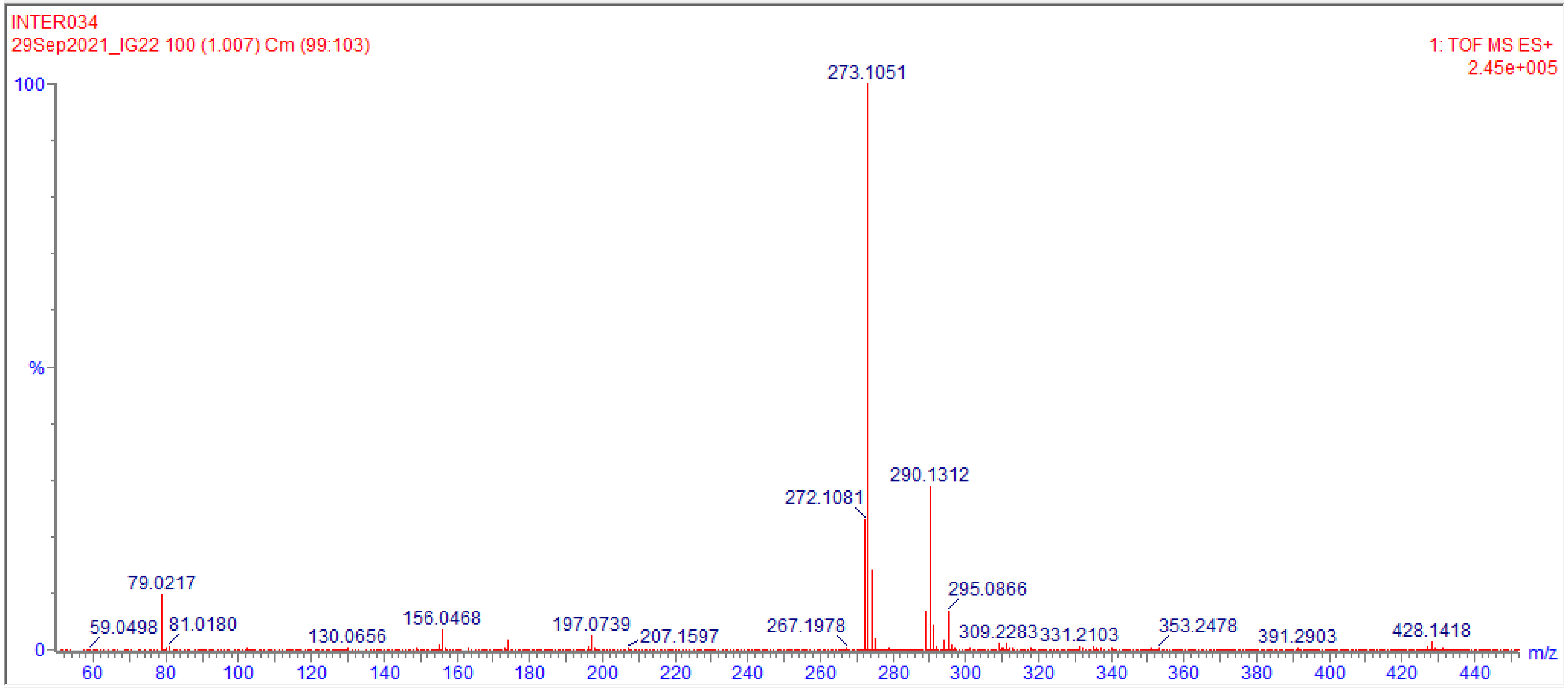

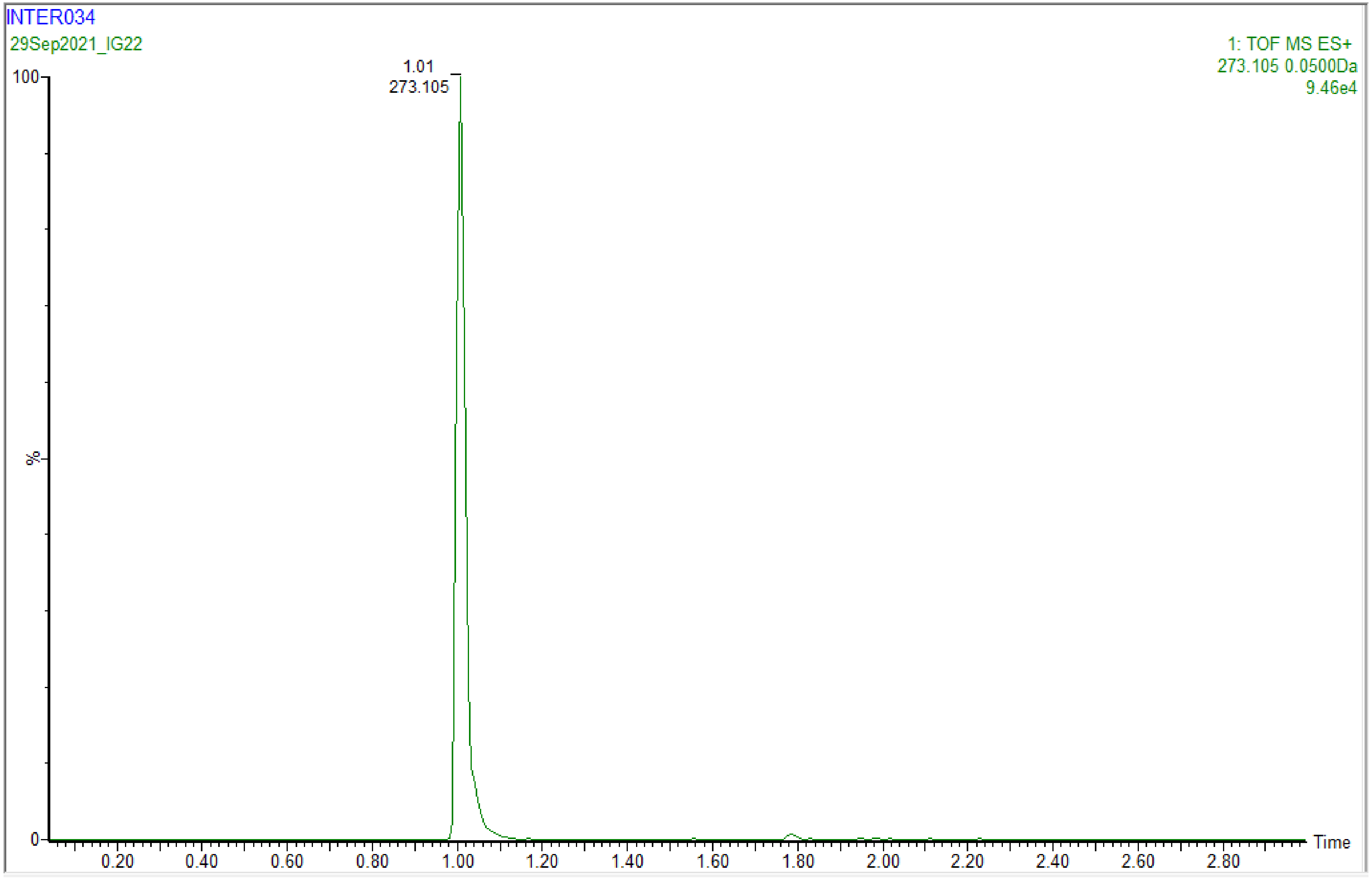
INTER034
29Sep2021_IG22
1: TOF MS ES+
273.105 0.0500Da
9.46e4
1.01
273.105
100
%
0
0.20
0.40
0.60
0.80
1.00
1.20
1.40
1.60
1.80
2.00
2.20
2.40
2.60
2.80
Time

6-Methyl-2-(1-methyl-3-(trifluoromethyl)-1H-pyrazol-5-yl)-1,3,6,2-dioxazaborocane-4,8-dione 13

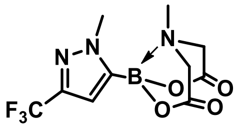

6-methyl-2-(1-methyl-3-(trifluoromethyl)-1*H*-pyrazol-5-yl)-1,3,6,2-dioxazaborocane-4,8-dione
Chemical Formula: $C_{10}H_{11}BF_3N_3O_4$
Molecular Weight: 305.0182

Yield = 21.0 mg (7%).

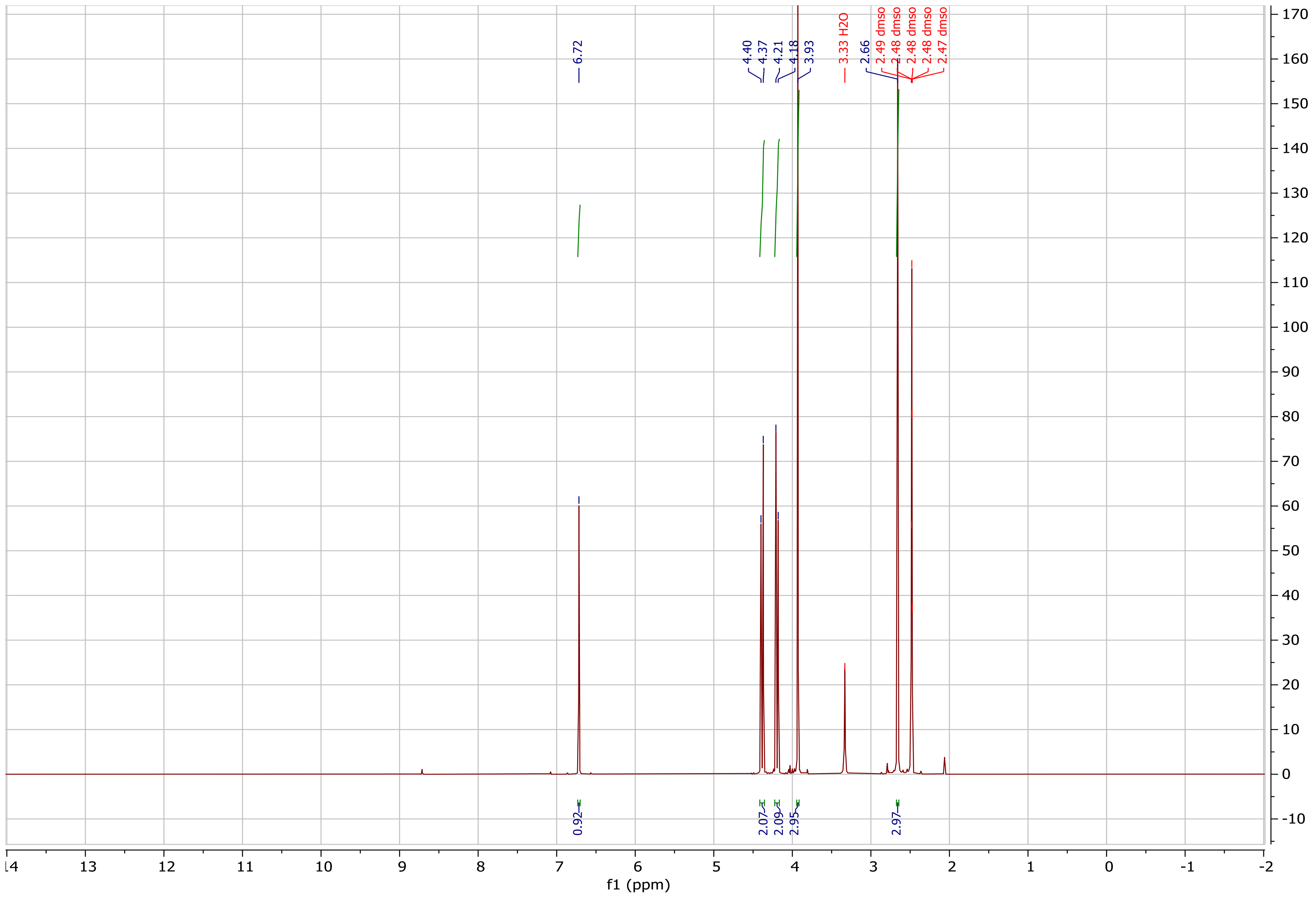
6.72
4.40
4.37
4.21
4.18
3.93
3.33 H2O
2.66
2.49 dmso
2.48 dmso
2.48 dmso
2.48 dmso
2.47 dmso
0.92
2.07
2.09
2.95
2.97
f1 (ppm)

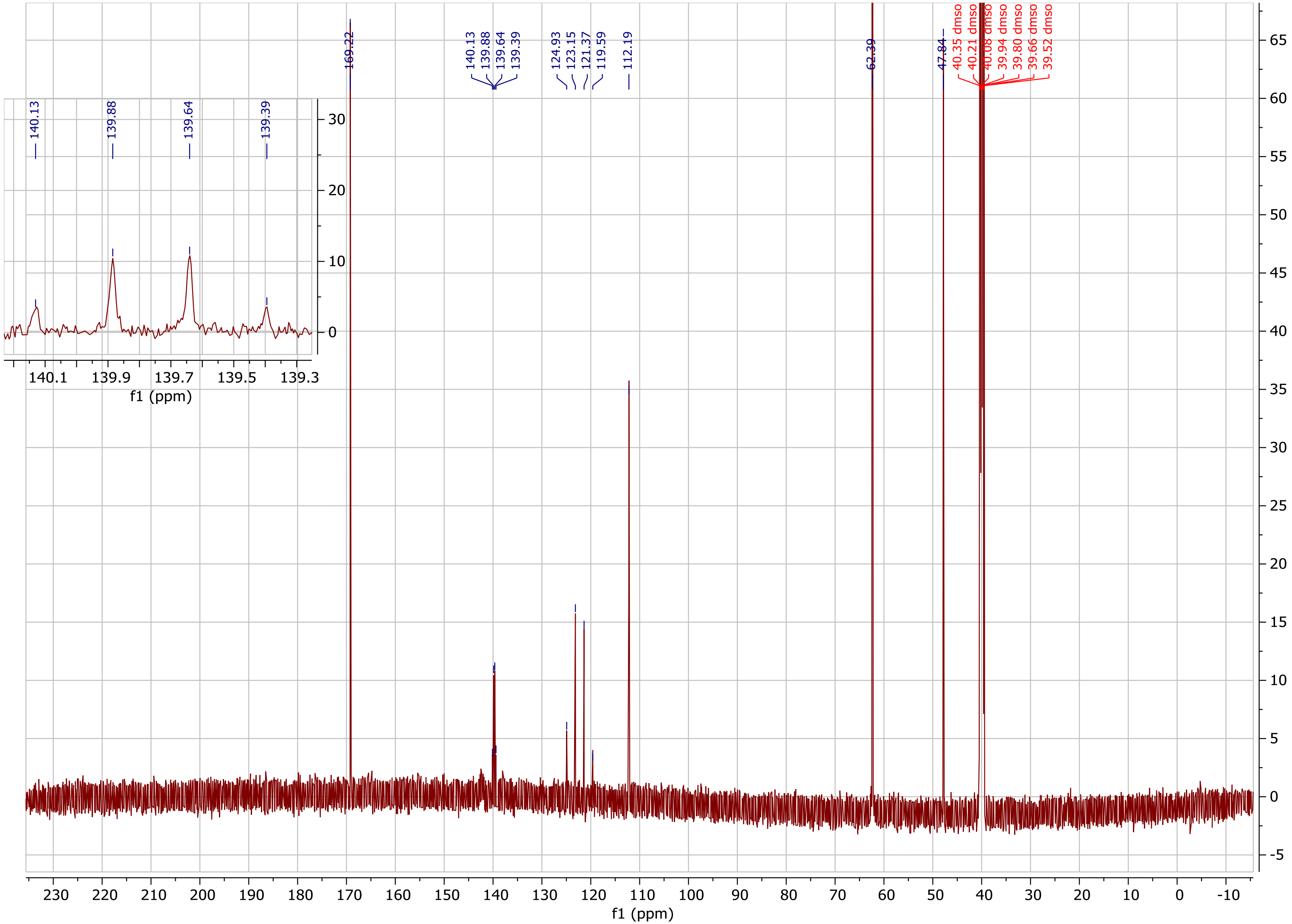

169.22
140.13
139.88
139.64
139.39
124.93
123.15
121.37
119.59
112.19
62.39
47.84
40.35 dmso
40.21 dmso
40.08 dmso
39.94 dmso
39.80 dmso
39.66 dmso
39.52 dmso
f1 (ppm)

Single Mass Analysis
Tolerance = 5.0 mDa / DBE: min = -1.5, max = 120.0
Element prediction: Off
Number of isotope peaks used for i-FIT = 3
Monoisotopic Mass, Even Electron Ions
306 formula(e) evaluated with 1 results within limits (up to 20 best isotopic matches for each mass)
Elements Used:

Mass	Calc. Mass	mDa	PPM	DBE	Formula	i-FIT	i-FIT Norm	Fit Conf %	C	H	11B	N	O	23Na	F
306.0875	306.0873	0.2	0.7	5.5	C10 H12 11B N3 O4 F3	169.5	n/a	n/a	10	12	1	3	4		3

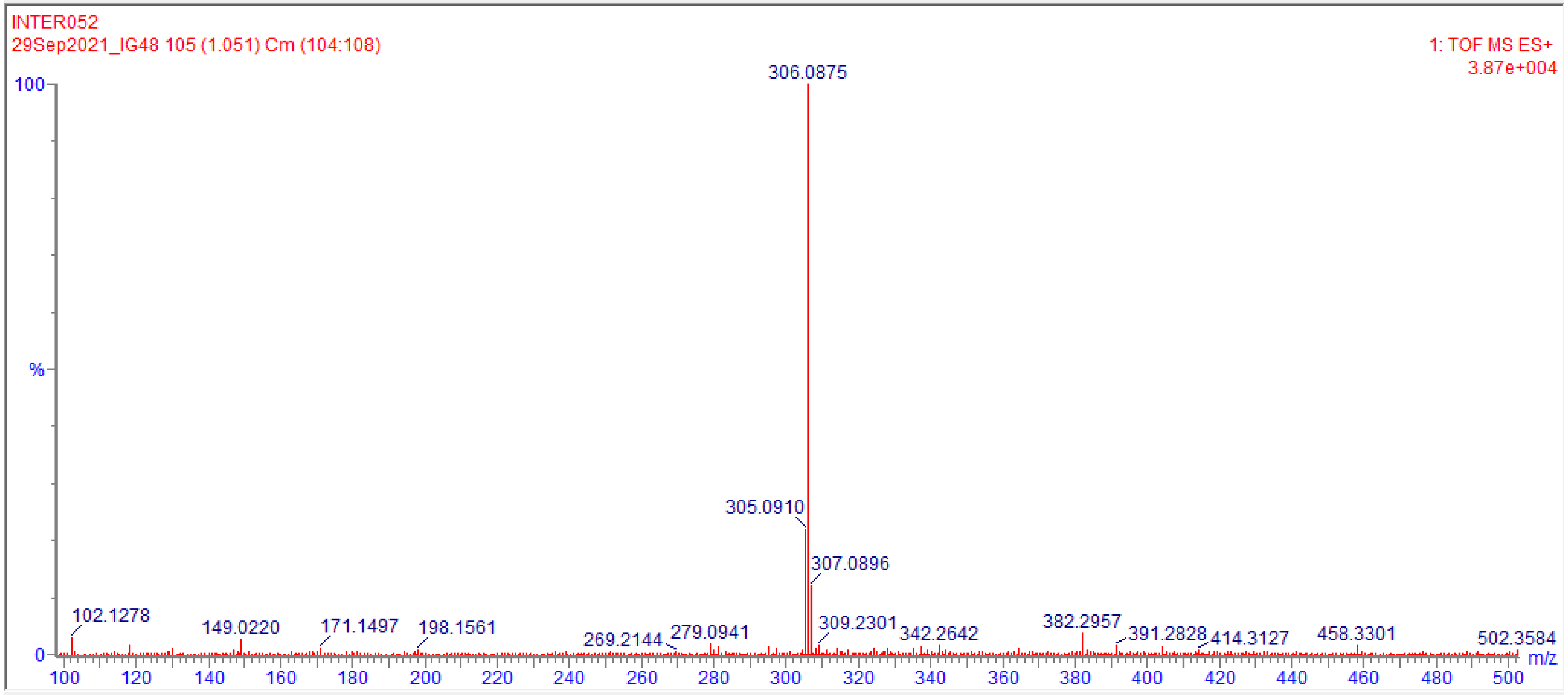

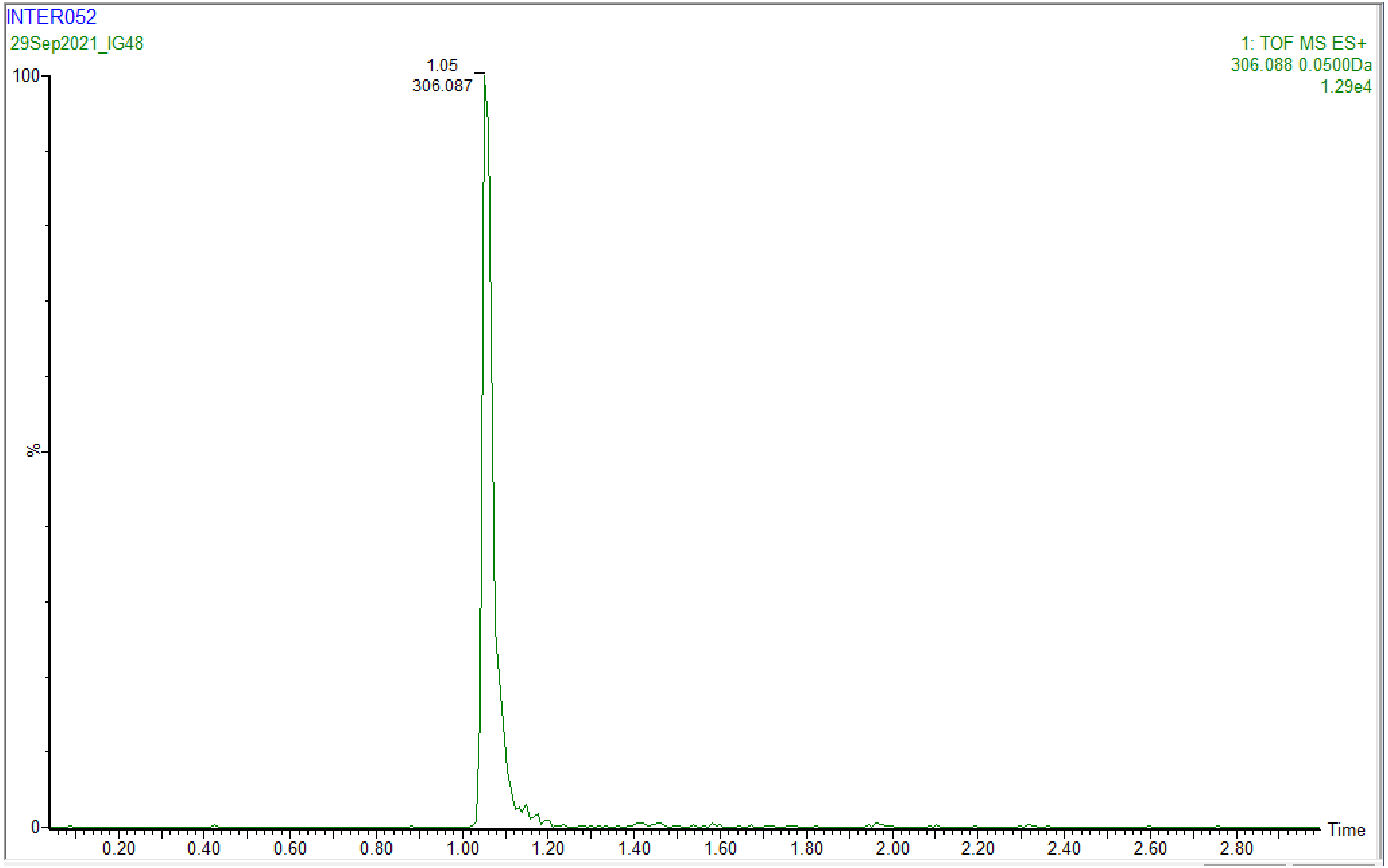
INTER052
29Sep2021_IG48
1: TOF MS ES+
306.088 0.0500Da
1.29e4
1.05
306.087
100
%
0
0.20
0.40
0.60
0.80
1.00
1.20
1.40
1.60
1.80
2.00
2.20
2.40
2.60
2.80
Time

2-(Benzo[d][1,3]dioxol-5-yl)-6-methyl-1,3,6,2-dioxazaborocane-4,8-dione 14

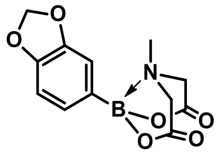

2-(benzo[*d*][1,3]dioxol-5-yl)-6-methyl-1,3,6,2-dioxazaborocane-4,8-dione
Chemical Formula: $C_{12}H_{12}BNO_6$
Molecular Weight: 277.0378

Yield = 199.1 mg (71%).

PROTON_01
AEPD-0072-2a

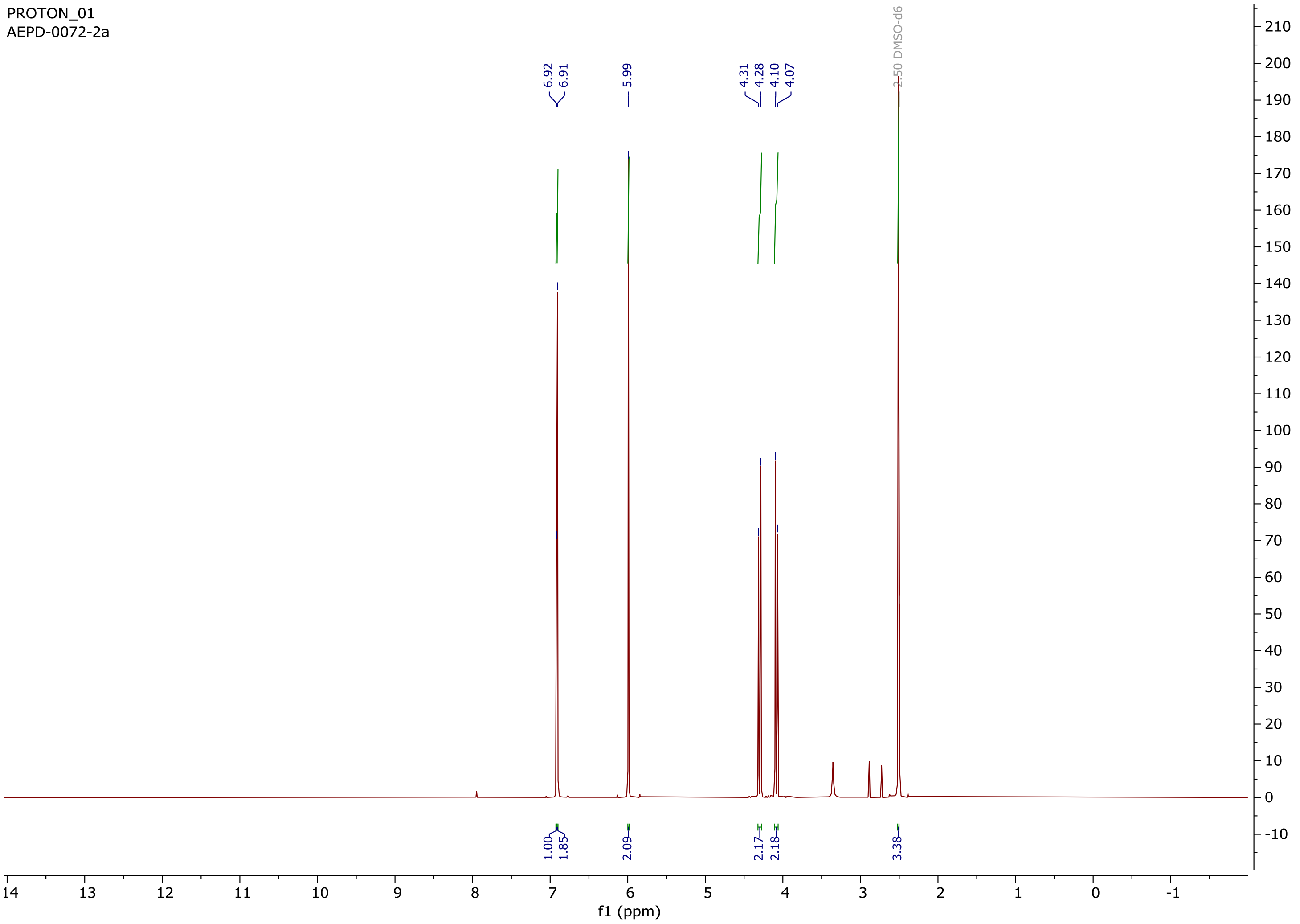

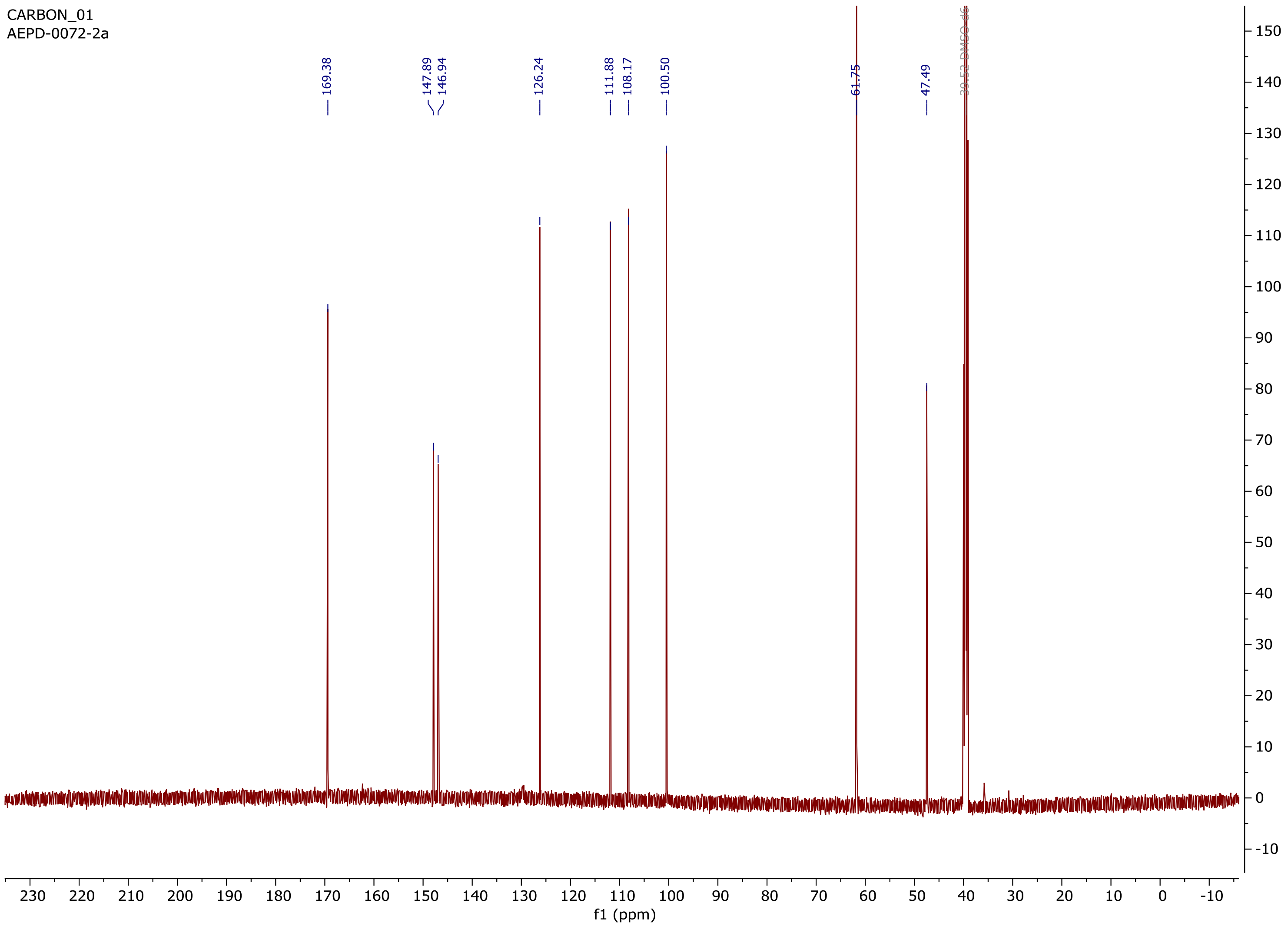

CARBON_01
AEPD-0072-2a
169.38
147.89
146.94
126.24
111.88
108.17
100.50
61.75
47.49
f1 (ppm)

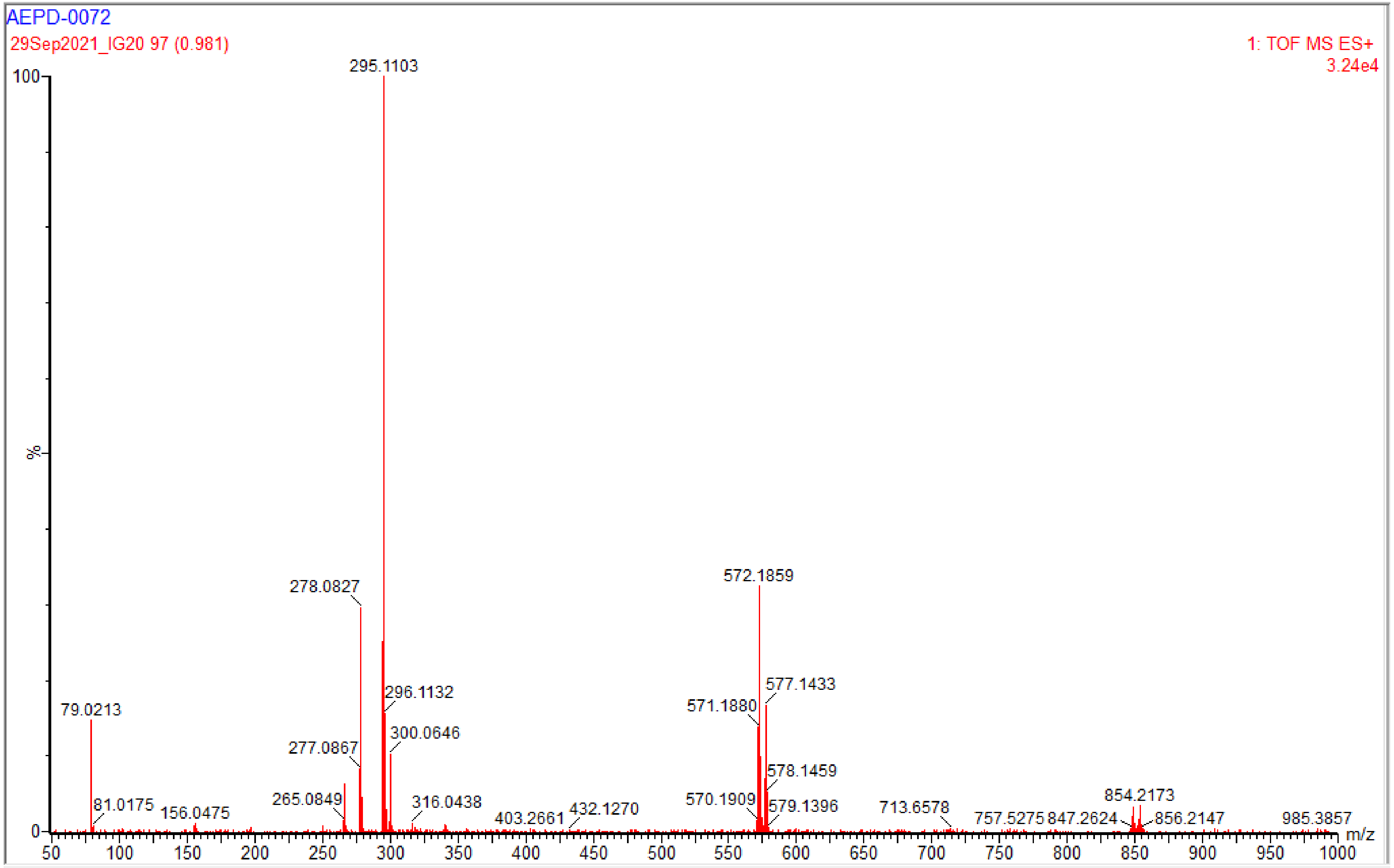
AEPD-0072
29Sep2021_IG20 97 (0.981)
1: TOF MS ES+
3.24e4
295.1103
278.0827
572.1859
577.1433
571.1880
296.1132
79.0213
300.0646
277.0867
578.1459
81.0175
156.0475
265.0849
316.0438
403.2661
432.1270
570.1909
579.1396
713.6578
757.5275
847.2624
854.2173
856.2147
985.3857
100
%
0
m/z
50
100
150
200
250
300
350
400
450
500
550
600
650
700
750
800
850
900
950
1000

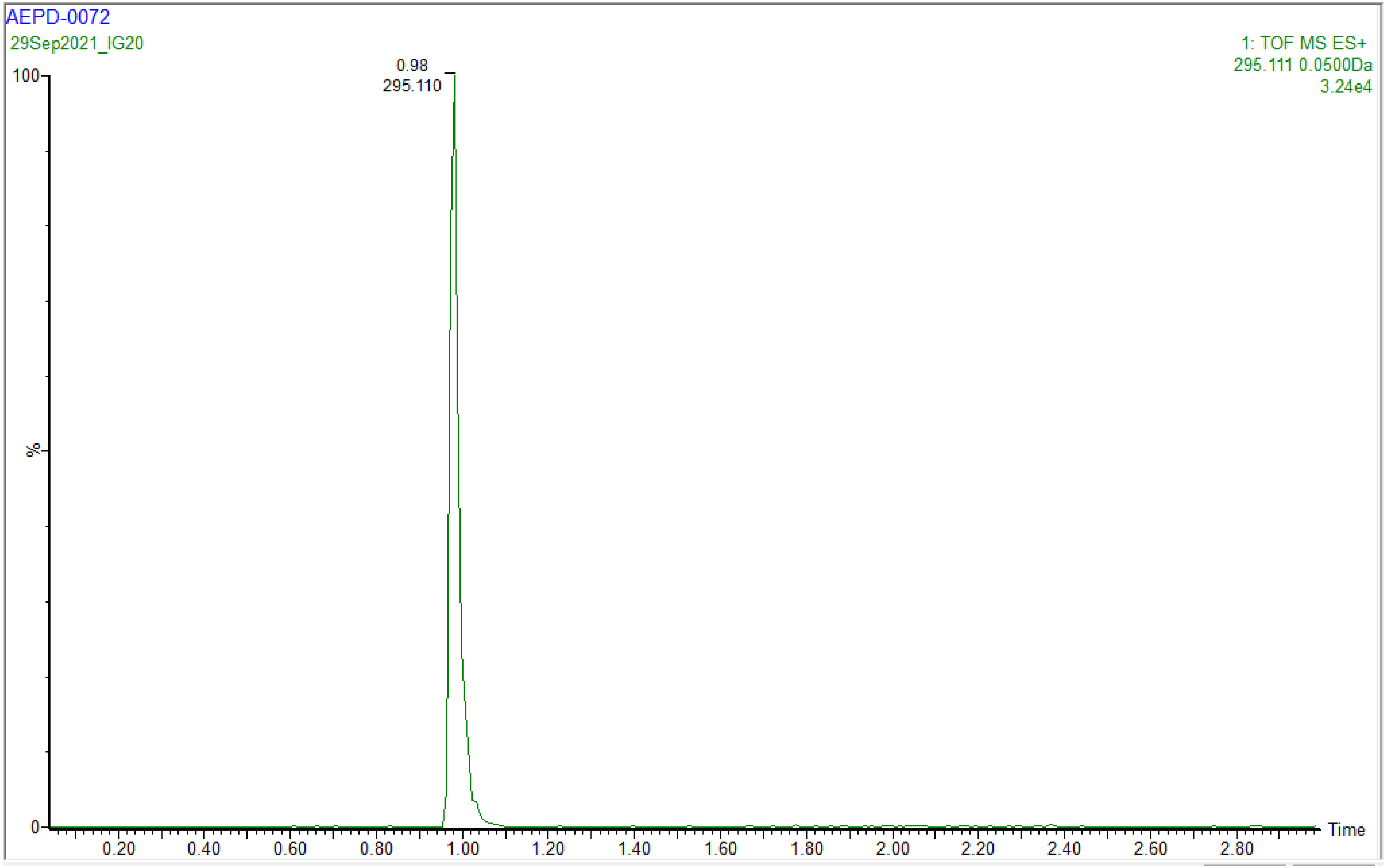
AEPD-0072
29Sep2021_IG20
1: TOF MS ES+
295.111 0.0500Da
3.24e4
0.98
295.110
100
%
0
0.20
0.40
0.60
0.80
1.00
1.20
1.40
1.60
1.80
2.00
2.20
2.40
2.60
2.80
Time

2-(3-ethoxyphenyl)-6-methyl-1,3,6,2-dioxazaborocane-4,8-dione 8c

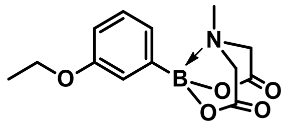

2-(3-ethoxyphenyl)-6-methyl-1,3,6,2-dioxazaborocane-4,8-dione
Chemical Formula: $C_{13}H_{16}BNO_5$
Molecular Weight: 277.0808

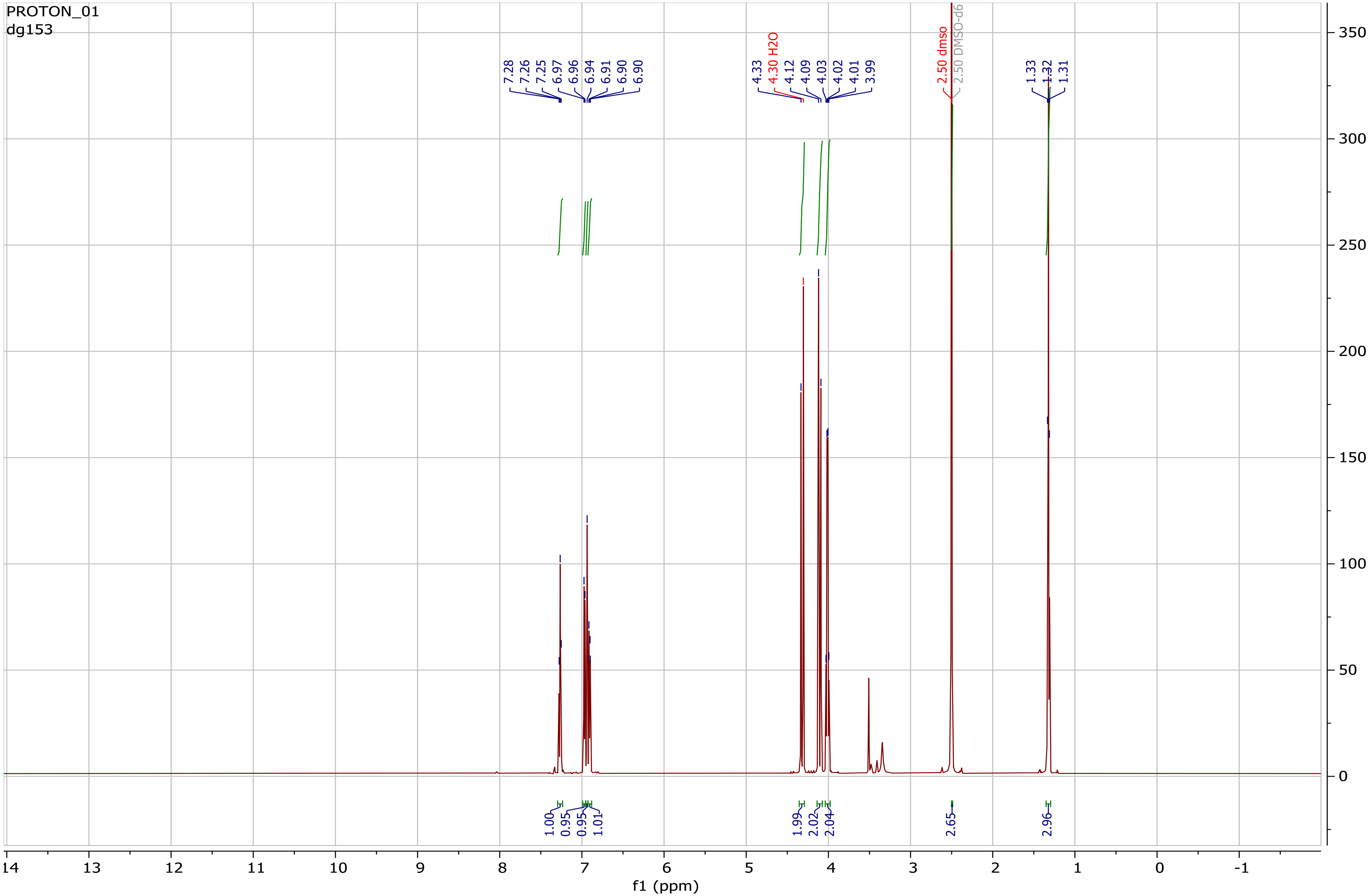

PROTON_01
dg153
7.28
7.26
7.25
6.97
6.96
6.94
6.91
6.90
6.90
4.33
4.30 H2O
4.12
4.09
4.03
4.02
4.01
3.99
2.50 dmso
2.50 DMSO-d6
1.33
1.32
1.31
1.00
0.95
0.95
1.01
1.99
2.02
2.04
2.65
2.96
f1 (ppm)

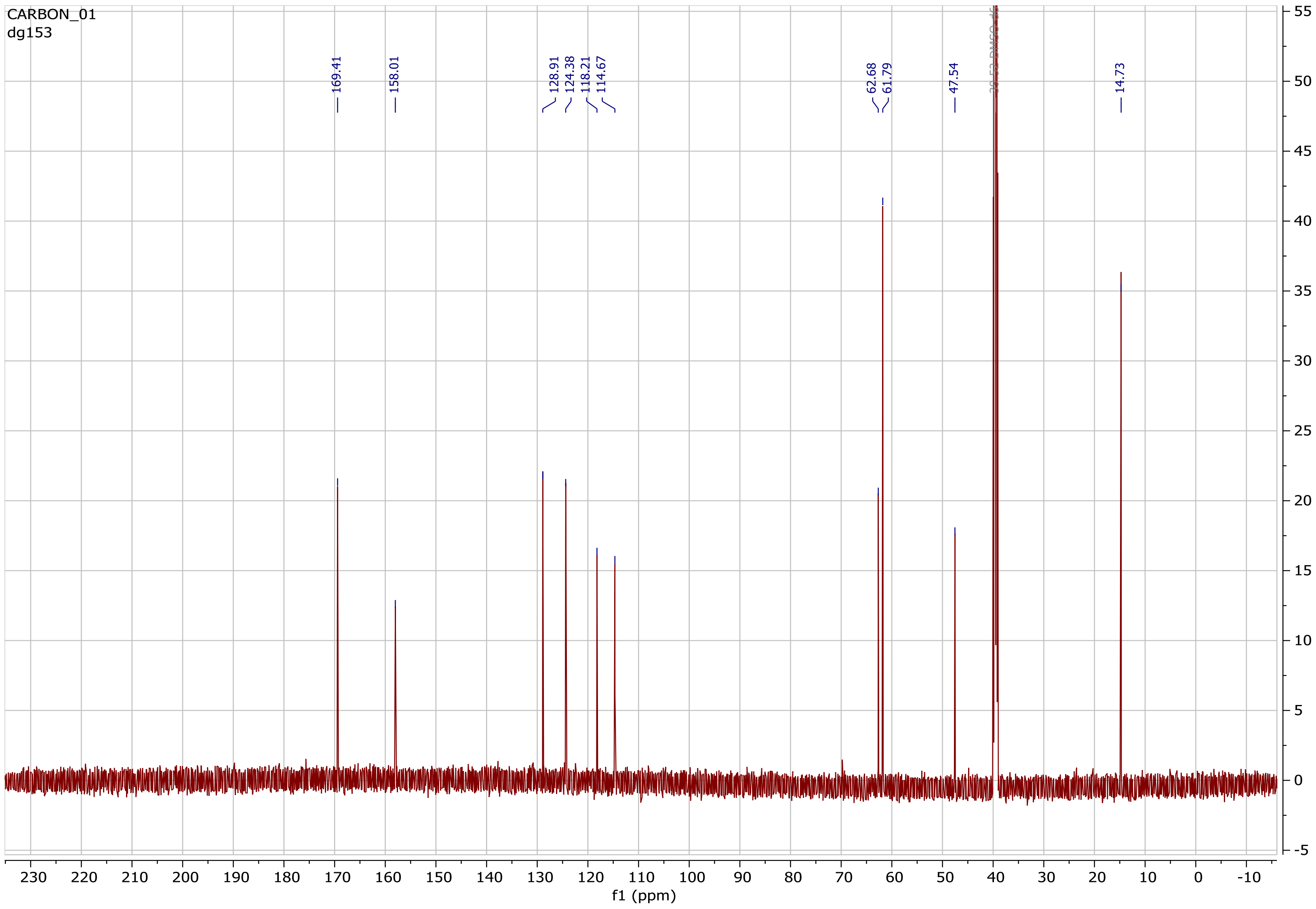

CARBON_01
dg153
169.41
158.01
128.91
124.38
118.21
114.67
62.68
61.79
47.54
14.73
f1 (ppm)

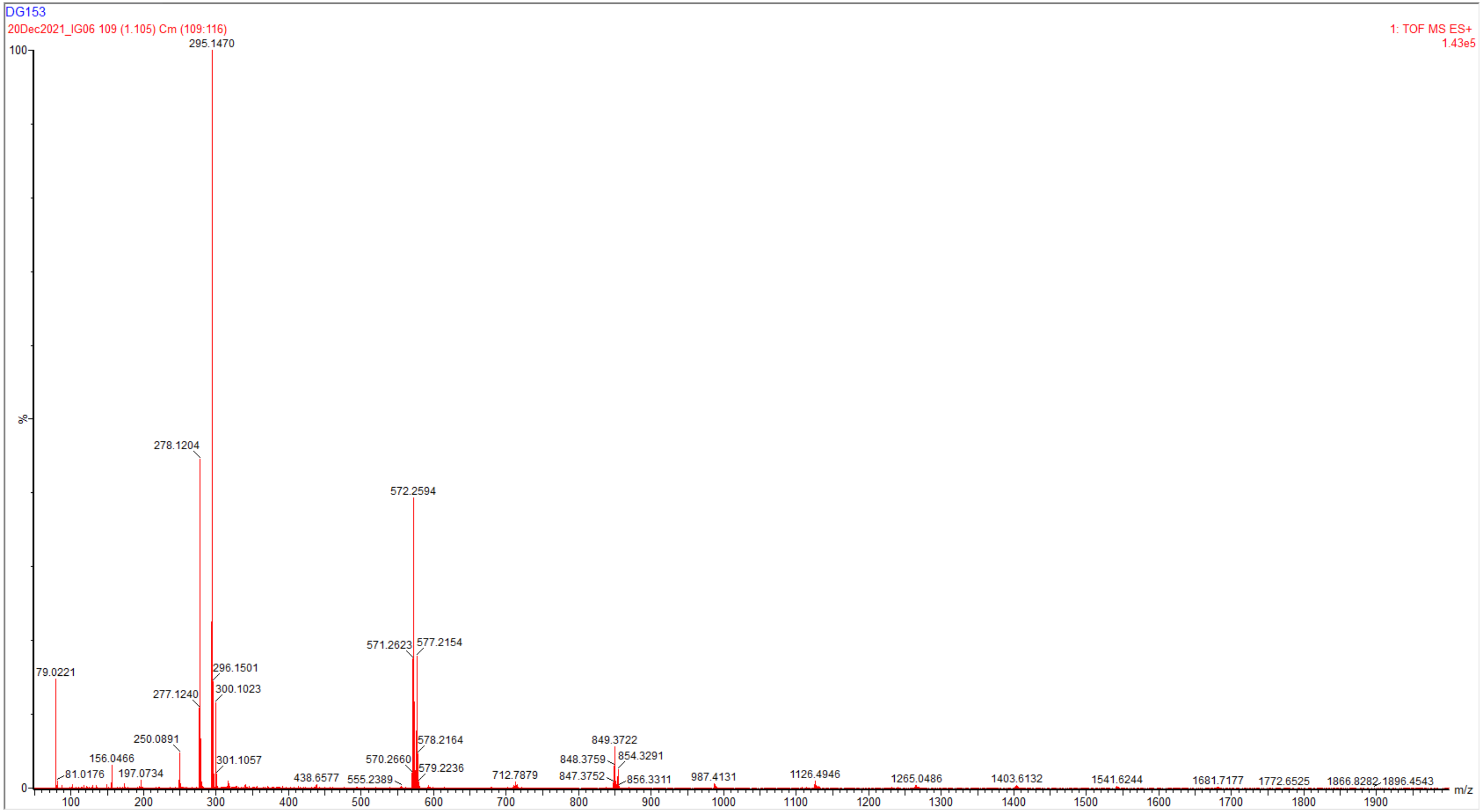
DG153
20Dec2021_IG06 109 (1.105) Cm (109:116)
1: TOF MS ES+
1.43e5
295.1470
278.1204
572.2594
571.2623
577.2154
296.1501
79.0221
277.1240
300.1023
250.0891
578.2164
849.3722
156.0466
570.2660
301.1057
848.3759
854.3291
81.0176
197.0734
579.2236
712.7879
847.3752
1126.4946
438.6577
555.2389
856.3311
987.4131
1265.0486
1403.6132
1541.6244
1681.7177
1772.6525
1866.8282
1896.4543
%
m/z

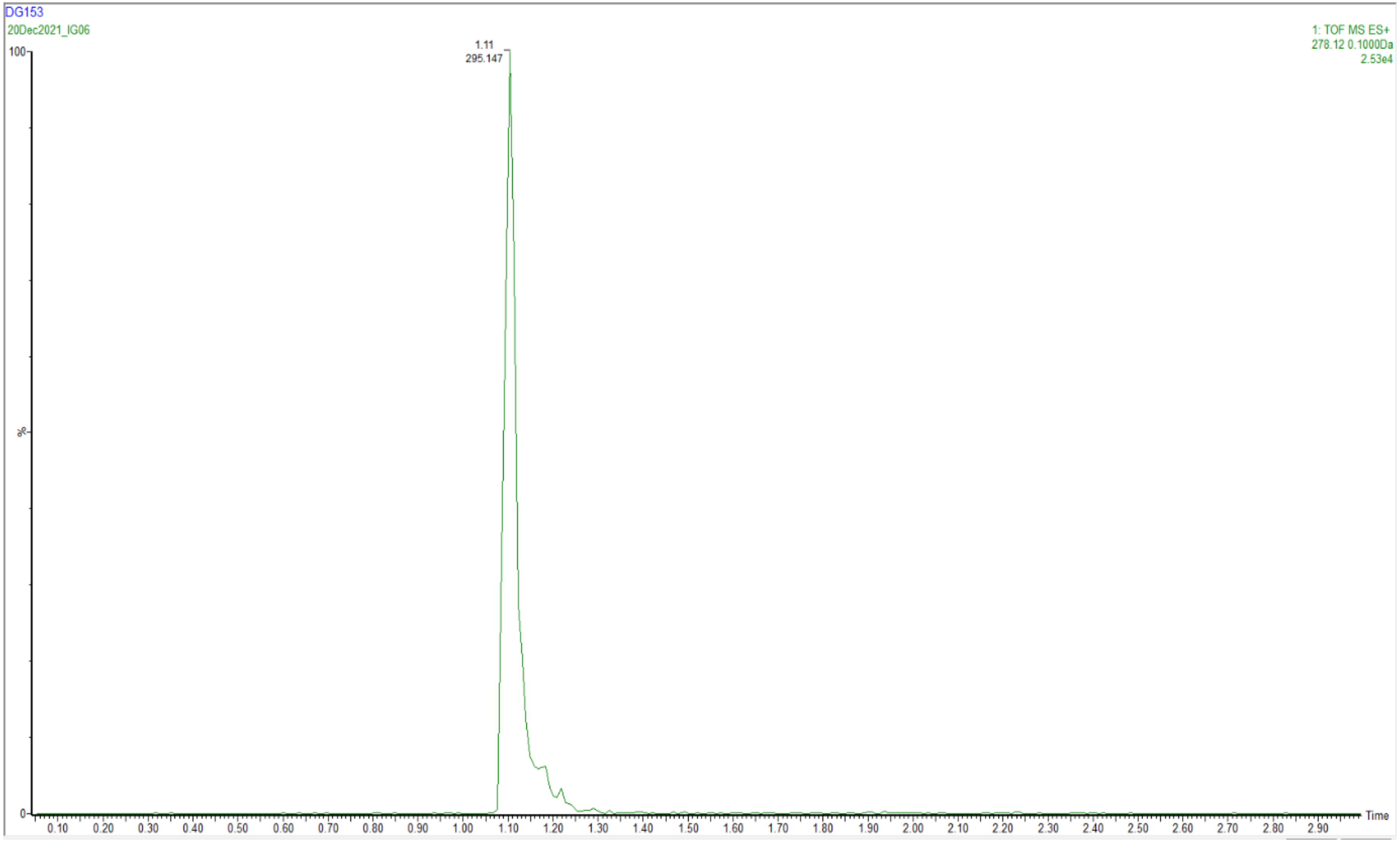

DG153
20Dec2021_IG06
1: TOF MS ES+
278.12 0.1000Da
2.53e4
1.11
295.147
100
%
0
0.10
0.20
0.30
0.40
0.50
0.60
0.70
0.80
0.90
1.00
1.10
1.20
1.30
1.40
1.50
1.60
1.70
1.80
1.90
2.00
2.10
2.20
2.30
2.40
2.50
2.60
2.70
2.80
2.90
Time

Methyl 4-(6-methyl-4,8-dioxo-1,3,6,2-dioxazaborocan-2-yl)benzoate 8d

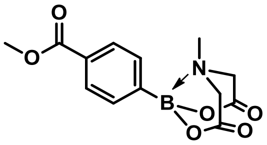

methyl 4-(6-methyl-4,8-dioxo-1,3,6,2-dioxazaborocan-2-yl)benzoate
Chemical Formula: $C_{13}H_{14}BNO_6$
Molecular Weight: 291.0644

Yield = 193.0 mg (67%).

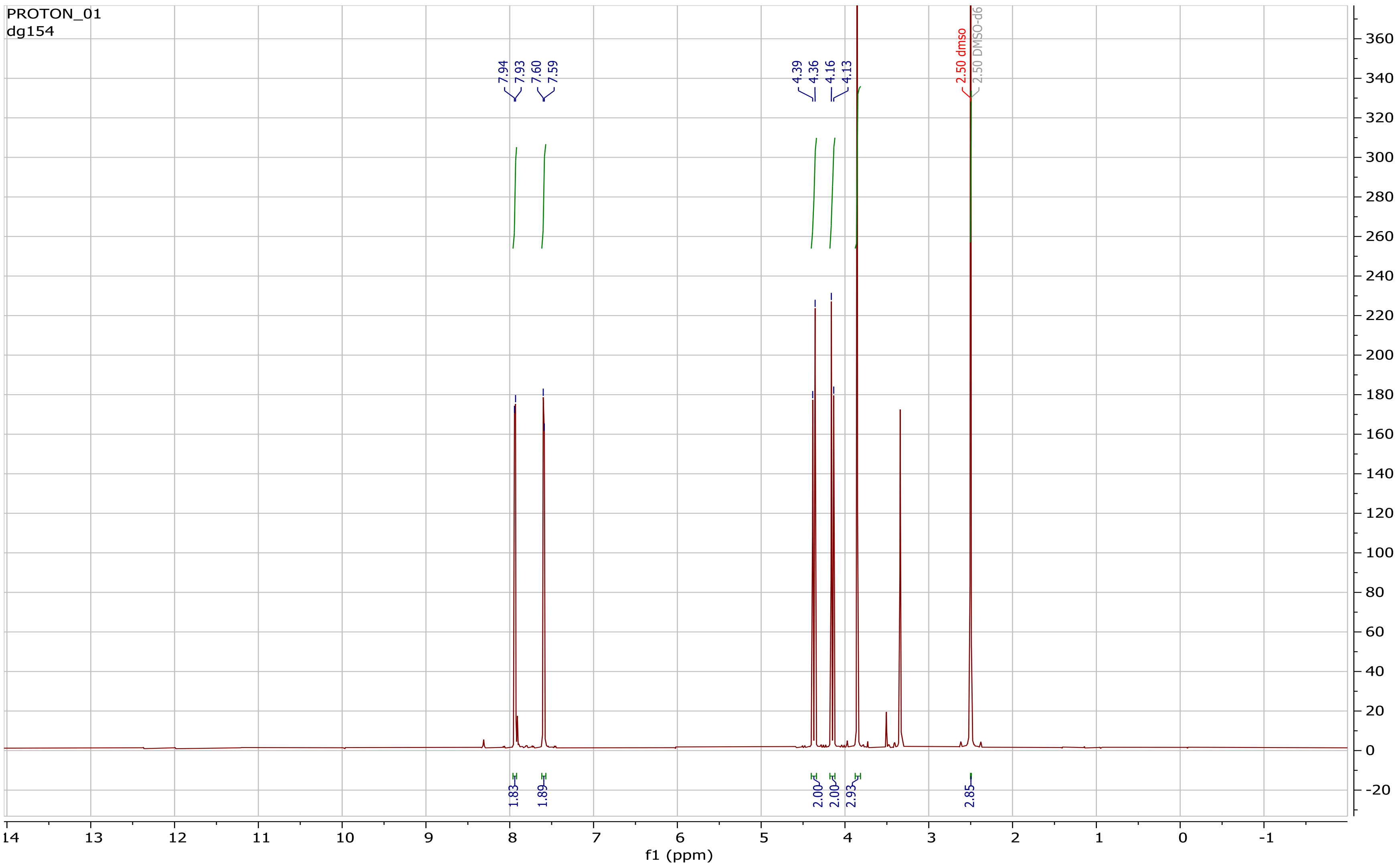
PROTON_01
dg154
7.94
7.93
7.60
7.59
4.39
4.36
4.16
4.13
2.50 dmso
2.50 DMSO-d6
1.83
1.89
2.00
2.00
2.93
2.85
f1 (ppm)

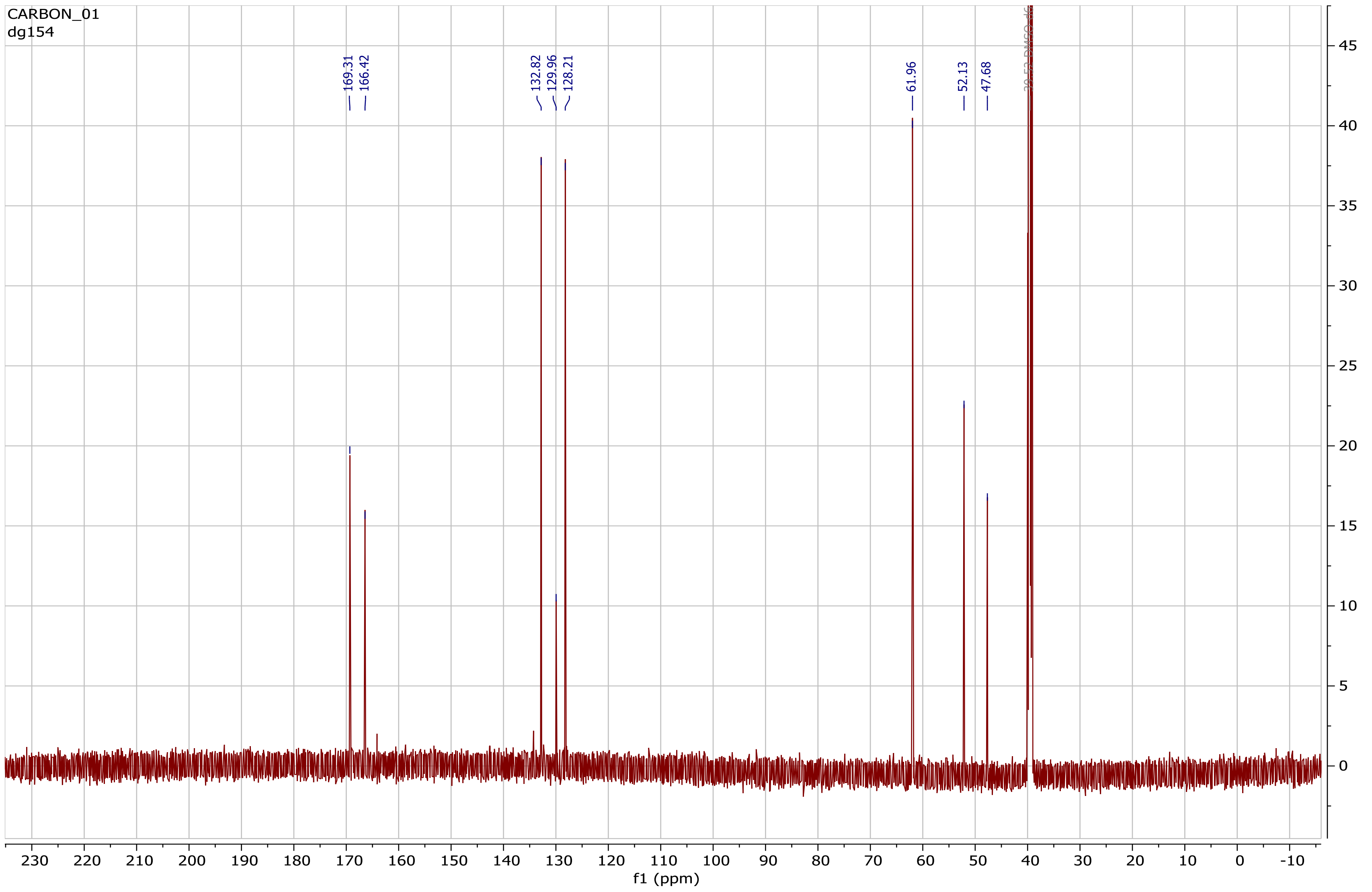
CARBON_01
dg154
169.31
166.42
132.82
129.96
128.21
61.96
52.13
47.68
39.52 DMSO-d6
f1 (ppm)

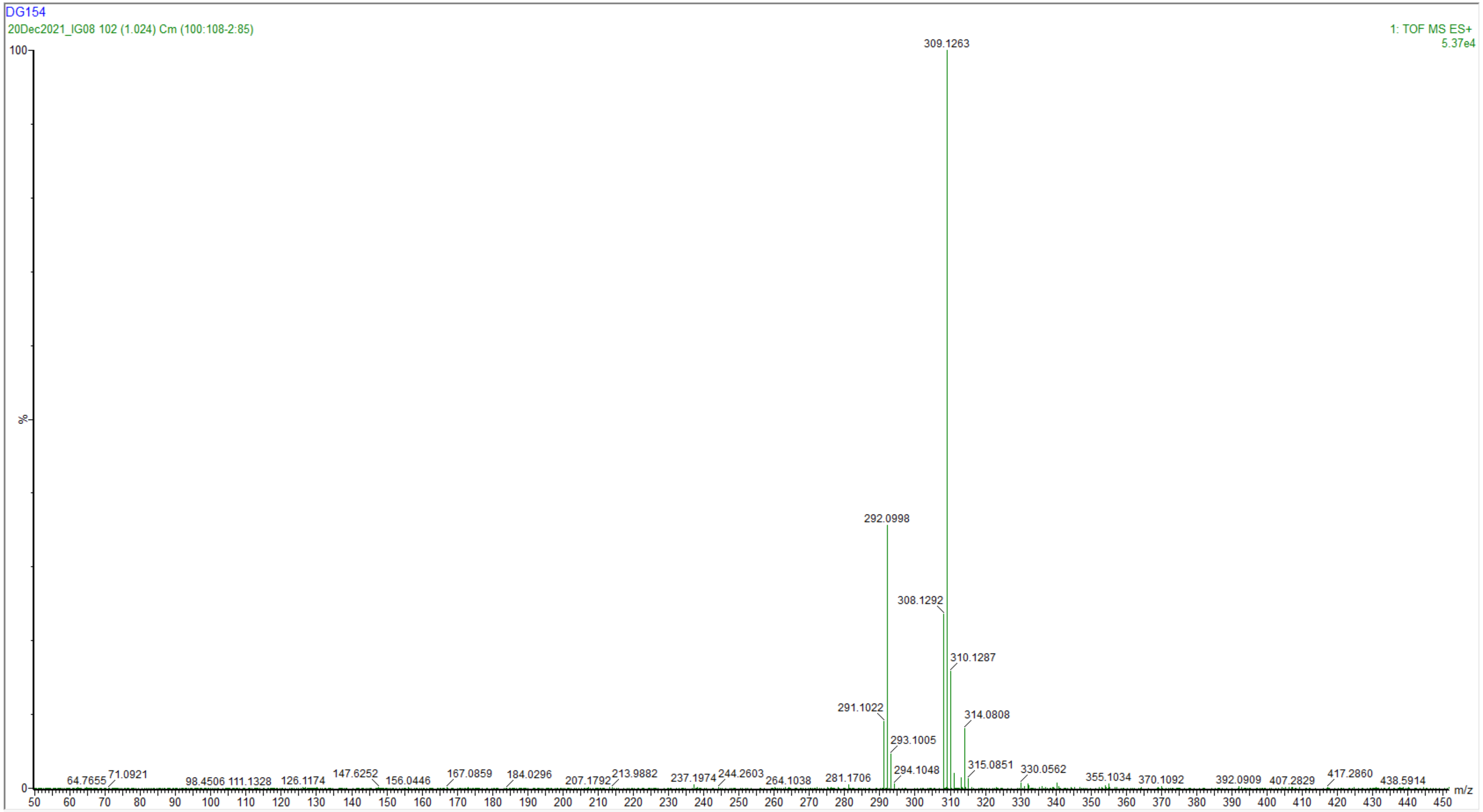
DG154
20Dec2021_IG08 102 (1.024) Cm (100:108-2:85)
1: TOF MS ES+
5.37e4
309.1263
292.0998
308.1292
310.1287
291.1022
314.0808
293.1005
294.1048
315.0851
330.0562
64.7655
71.0921
98.4506
111.1328
126.1174
147.6252
156.0446
167.0859
184.0296
207.1792
213.9882
237.1974
244.2603
264.1038
281.1706
355.1034
370.1092
392.0909
407.2829
417.2860
438.5914
100
%
0
m/z
50
60
70
80
90
100
110
120
130
140
150
160
170
180
190
200
210
220
230
240
250
260
270
280
290
300
310
320
330
340
350
360
370
380
390
400
410
420
430
440
450

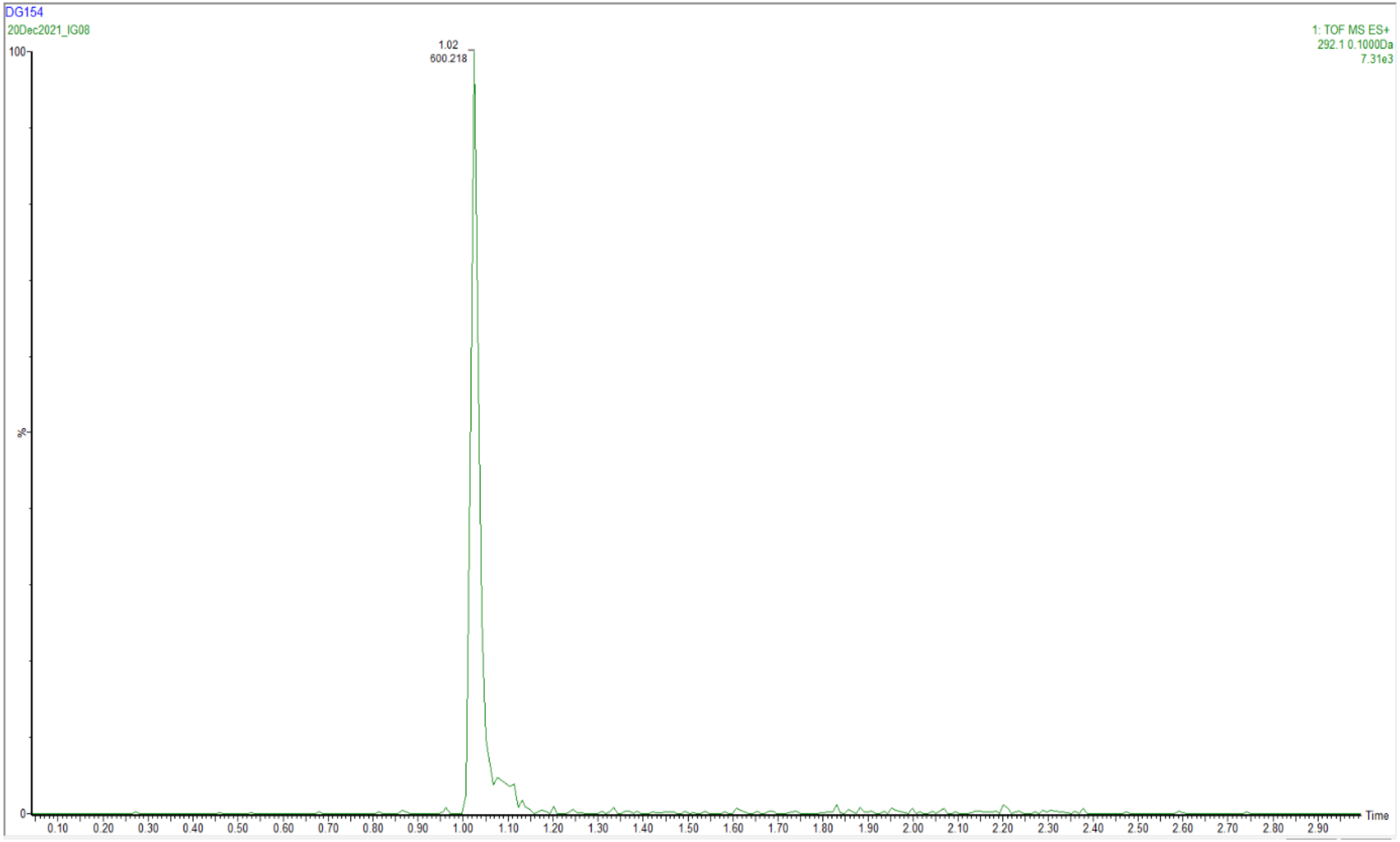

DG154
20Dec2021_IG08
1: TOF MS ES+
292.1 0.1000Da
7.31e3
1.02
600.218
100
%
0
0.10
0.20
0.30
0.40
0.50
0.60
0.70
0.80
0.90
1.00
1.10
1.20
1.30
1.40
1.50
1.60
1.70
1.80
1.90
2.00
2.10
2.20
2.30
2.40
2.50
2.60
2.70
2.80
2.90
Time

2-([1,1'-biphenyl]-4-yl)-6-methyl-1,3,6,2-dioxazaborocane-4,8-dione 8l

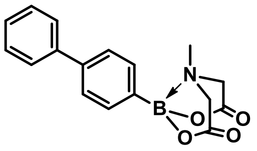

2-([1,1'-biphenyl]-4-yl)-6-methyl-1,3,6,2-dioxazaborocane-4,8-dione
Chemical Formula: $C_{17}H_{16}BNO_4$
Molecular Weight: 309.1242

Yield = 309.0 mg (99%).

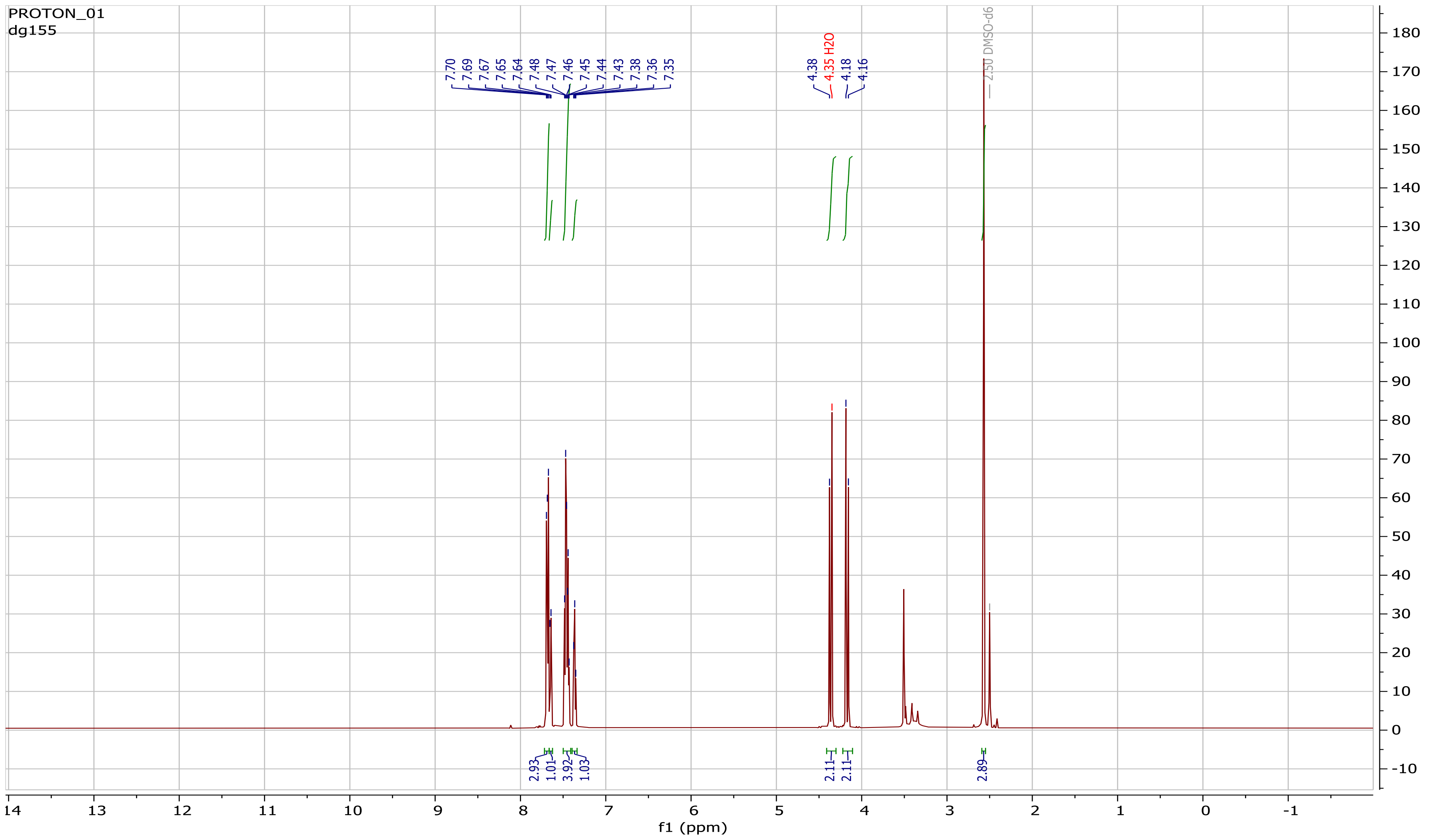
PROTON_01
dg155
7.70
7.69
7.67
7.65
7.64
7.48
7.47
7.46
7.45
7.44
7.43
7.38
7.36
7.35
4.38
4.35 H2O
4.18
4.16
2.50 DMSO-d6
2.93
1.01
3.92
1.03
2.11
2.11
2.89
f1 (ppm)

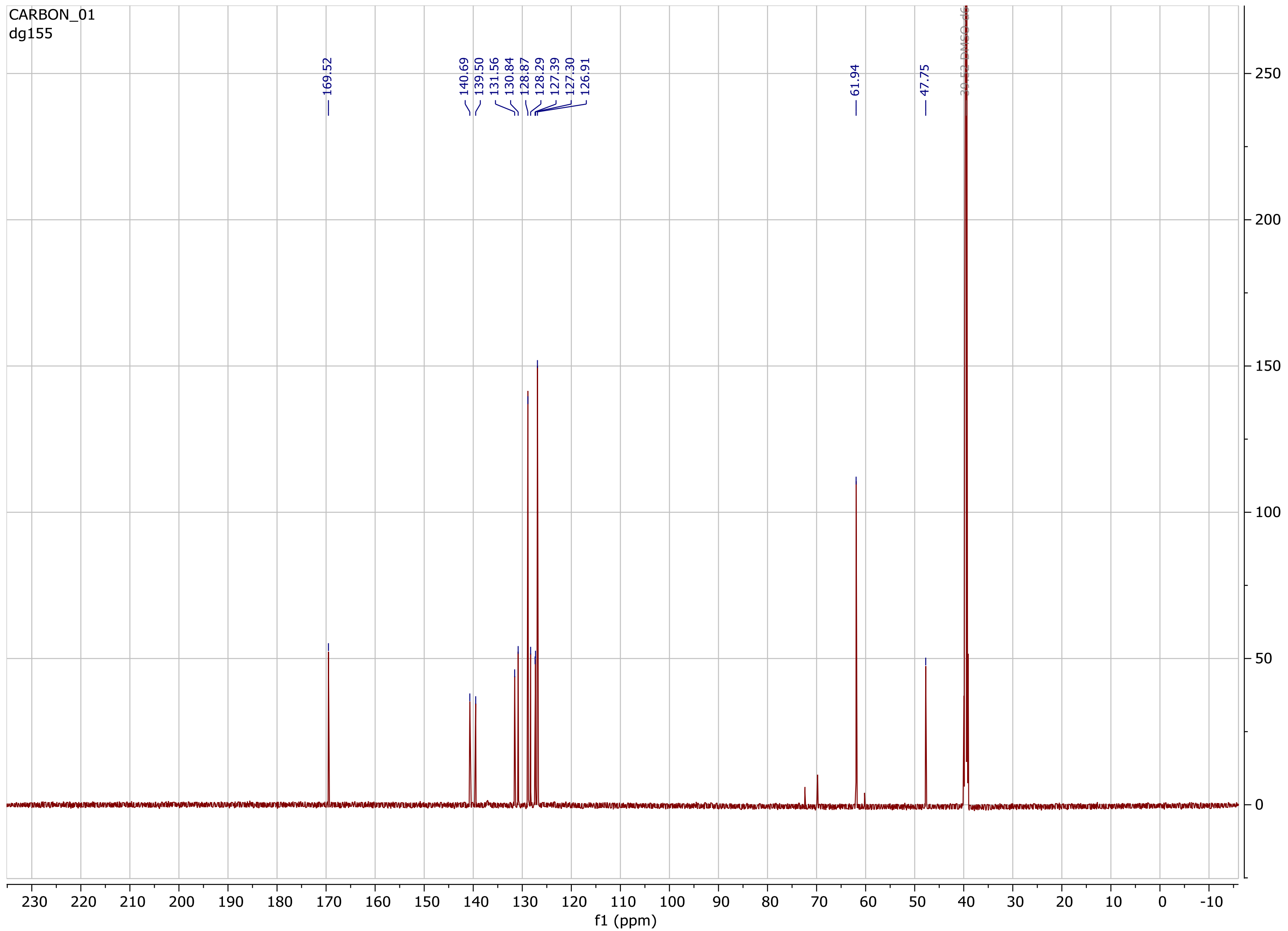

CARBON_01
dg155
169.52
140.69
139.50
131.56
130.84
128.87
128.29
127.39
127.30
126.91
61.94
47.75
39.52 DMSO-d6
f1 (ppm)

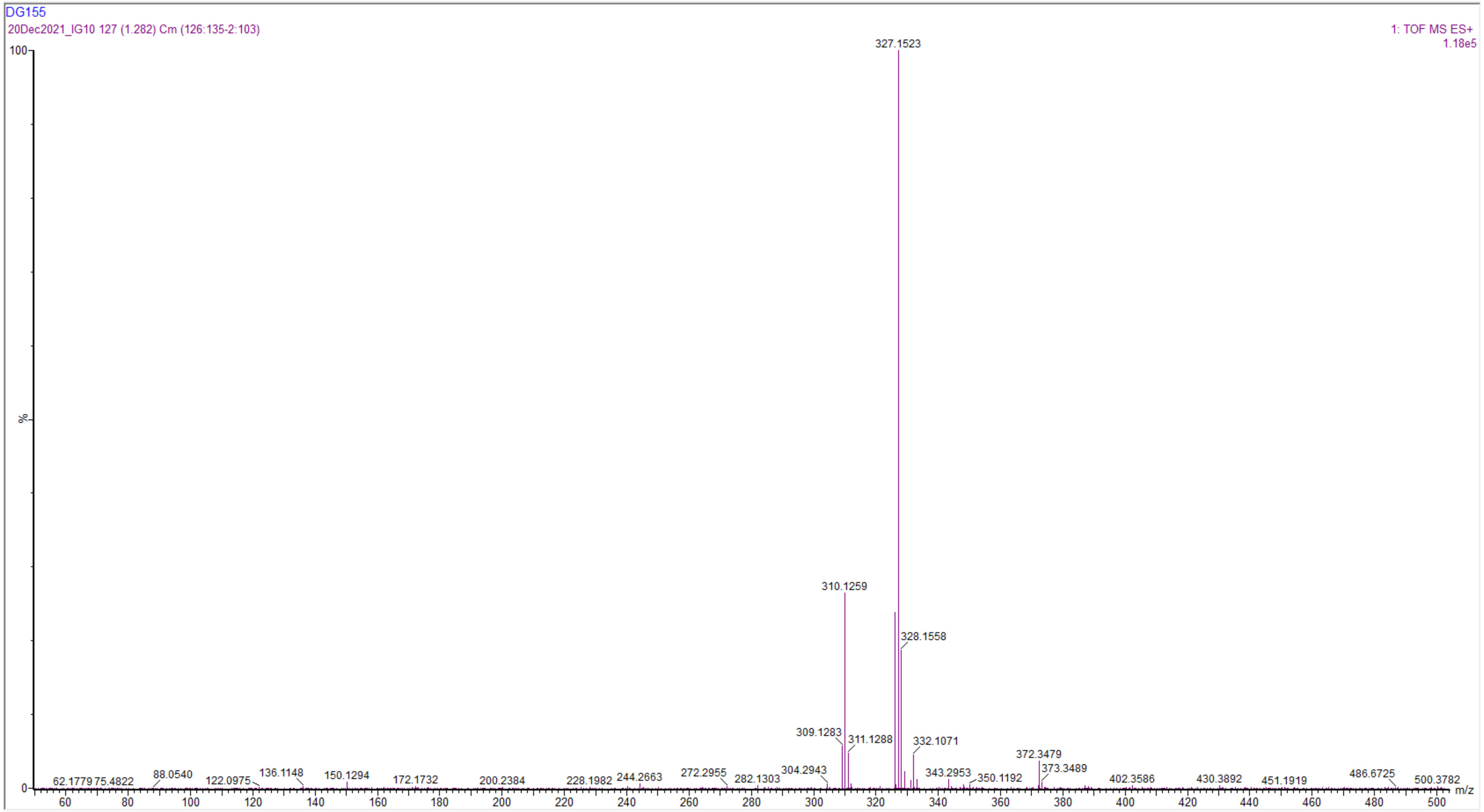

HPLC Purity > 95%

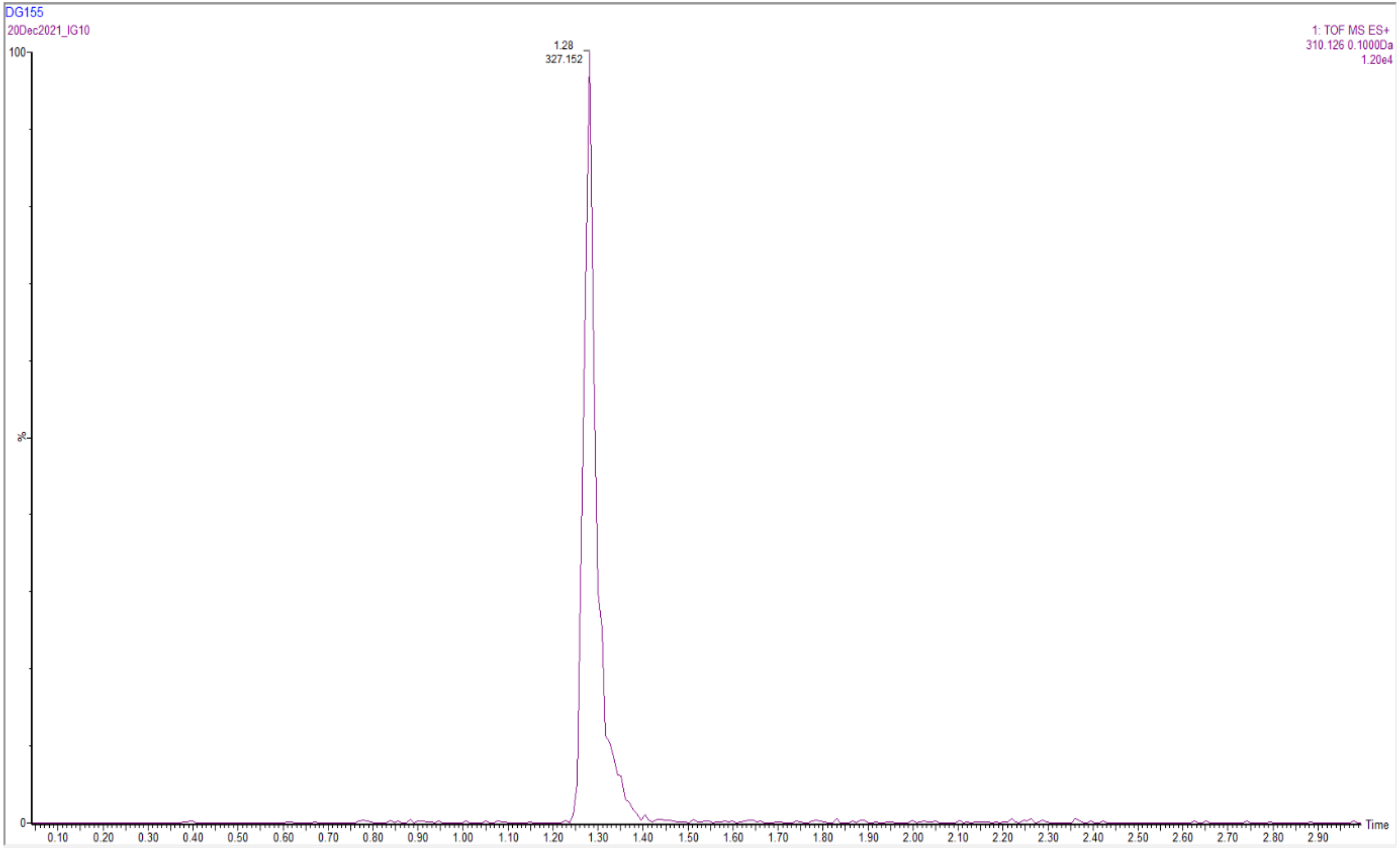

DG155
20Dec2021_IG10
1: TOF MS ES+
310.126 0.1000Da
1.20e4
1.28
327.152
100
%
0
Time
0.10
0.20
0.30
0.40
0.50
0.60
0.70
0.80
0.90
1.00
1.10
1.20
1.30
1.40
1.50
1.60
1.70
1.80
1.90
2.00
2.10
2.20
2.30
2.40
2.50
2.60
2.70
2.80
2.90

2-(4-(tert-Butyl)phenyl)-6-methyl-1,3,6,2-dioxazaborocane-4,8-dione 8q

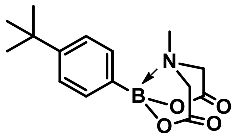

2-(4-(*tert*-butyl)phenyl)-6-methyl-1,3,6,2-dioxazaborocane-4,8-dione
Chemical Formula: $C_{15}H_{20}BNO_4$
Molecular Weight: 289.1346

Yield = 299.0 mg (97 %).

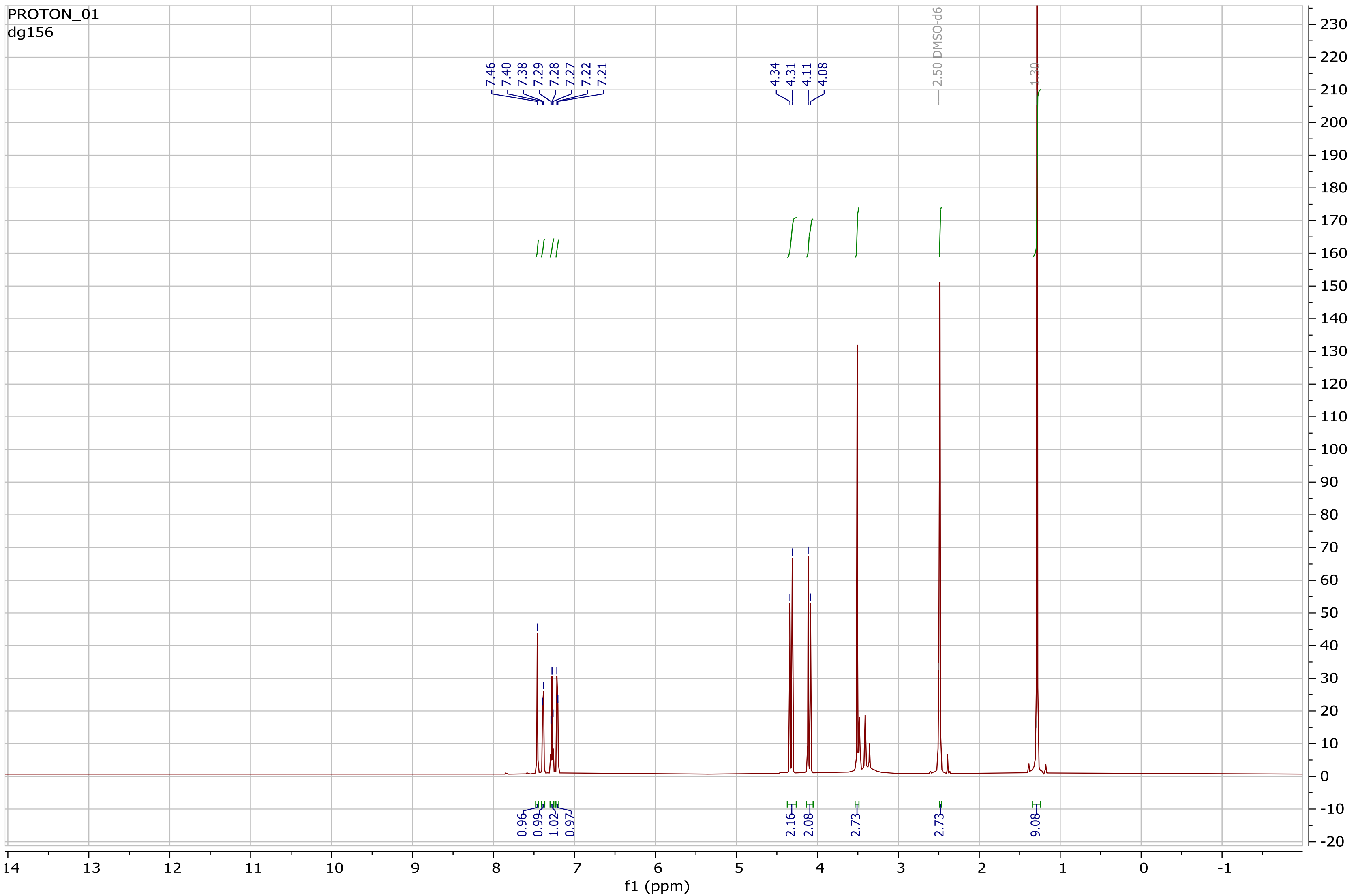
PROTON_01
dg156
7.46
7.40
7.38
7.29
7.28
7.27
7.22
7.21
4.34
4.31
4.11
4.08
2.50 DMSO-d6
1.30
0.96
0.99
1.02
0.97
2.16
2.08
2.73
2.73
9.08
f1 (ppm)

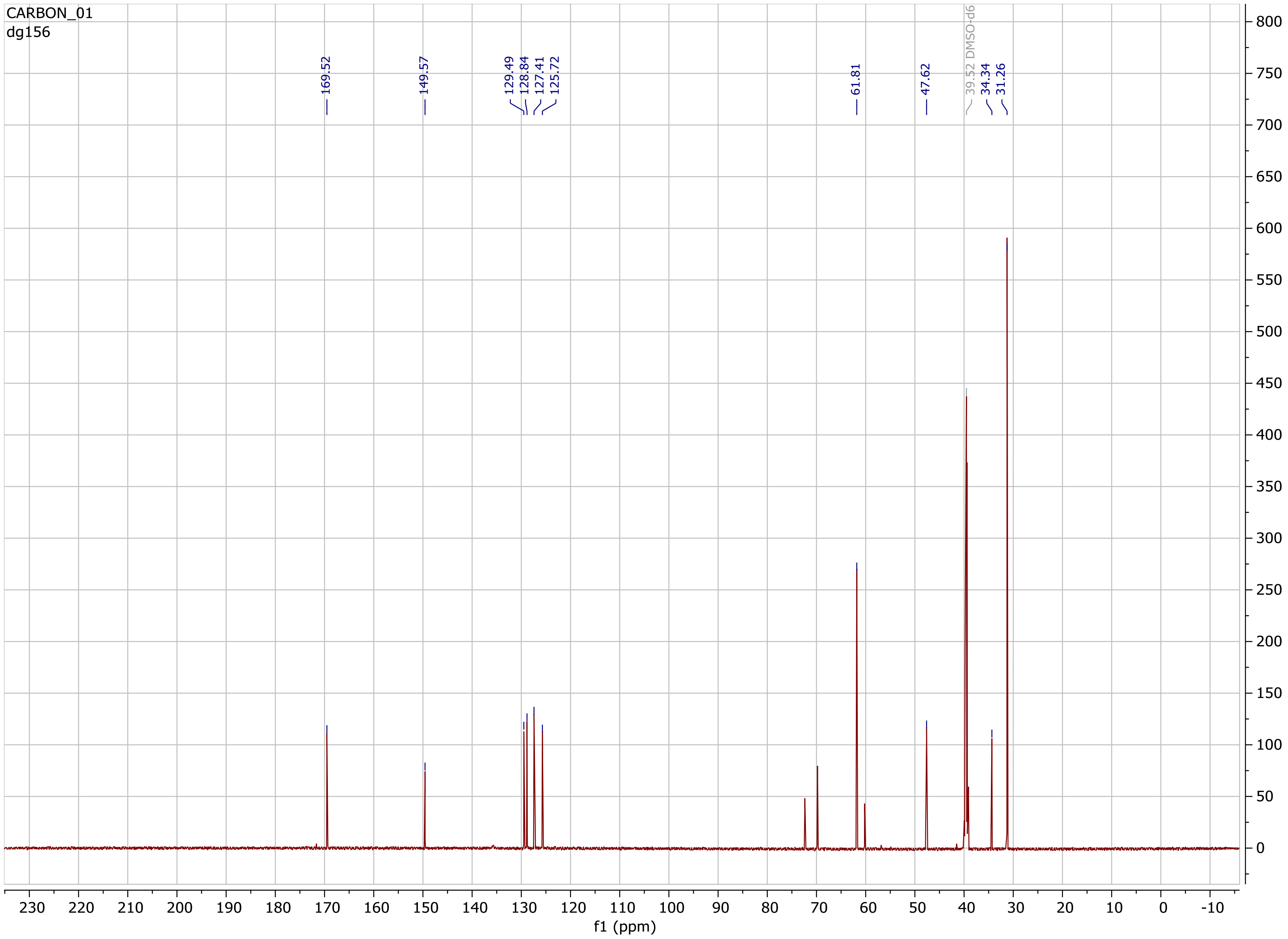
CARBON_01
dg156
169.52
149.57
129.49
128.84
127.41
125.72
61.81
47.62
39.52 DMSO-d6
34.34
31.26
800
750
700
650
600
550
500
450
400
350
300
250
200
150
100
50
0
230 220 210 200 190 180 170 160 150 140 130 120 110 100 90 80 70 60 50 40 30 20 10 0 -10
f1 (ppm)

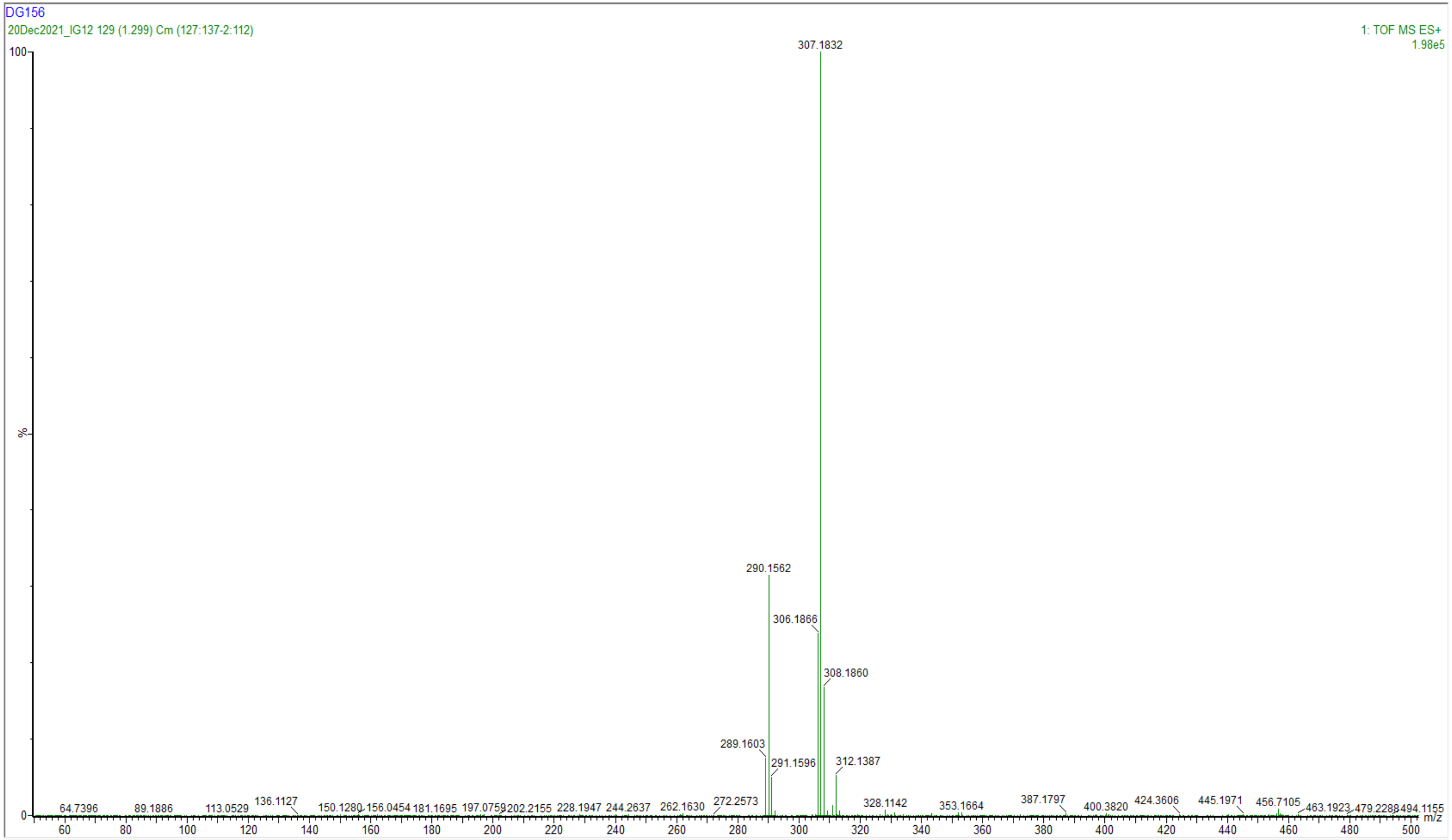
DG156
20Dec2021_IG12 129 (1.299) Cm (127:137-2:112)
1: TOF MS ES+
1.98e5
307.1832
290.1562
306.1866
308.1860
289.1603
291.1596
312.1387
64.7396
89.1886
113.0529
136.1127
150.1280
156.0454
181.1695
197.0759
202.2155
228.1947
244.2637
262.1630
272.2573
328.1142
353.1664
387.1797
400.3820
424.3606
445.1971
456.7105
463.1923
479.2288
494.1155
m/z
%
100
0

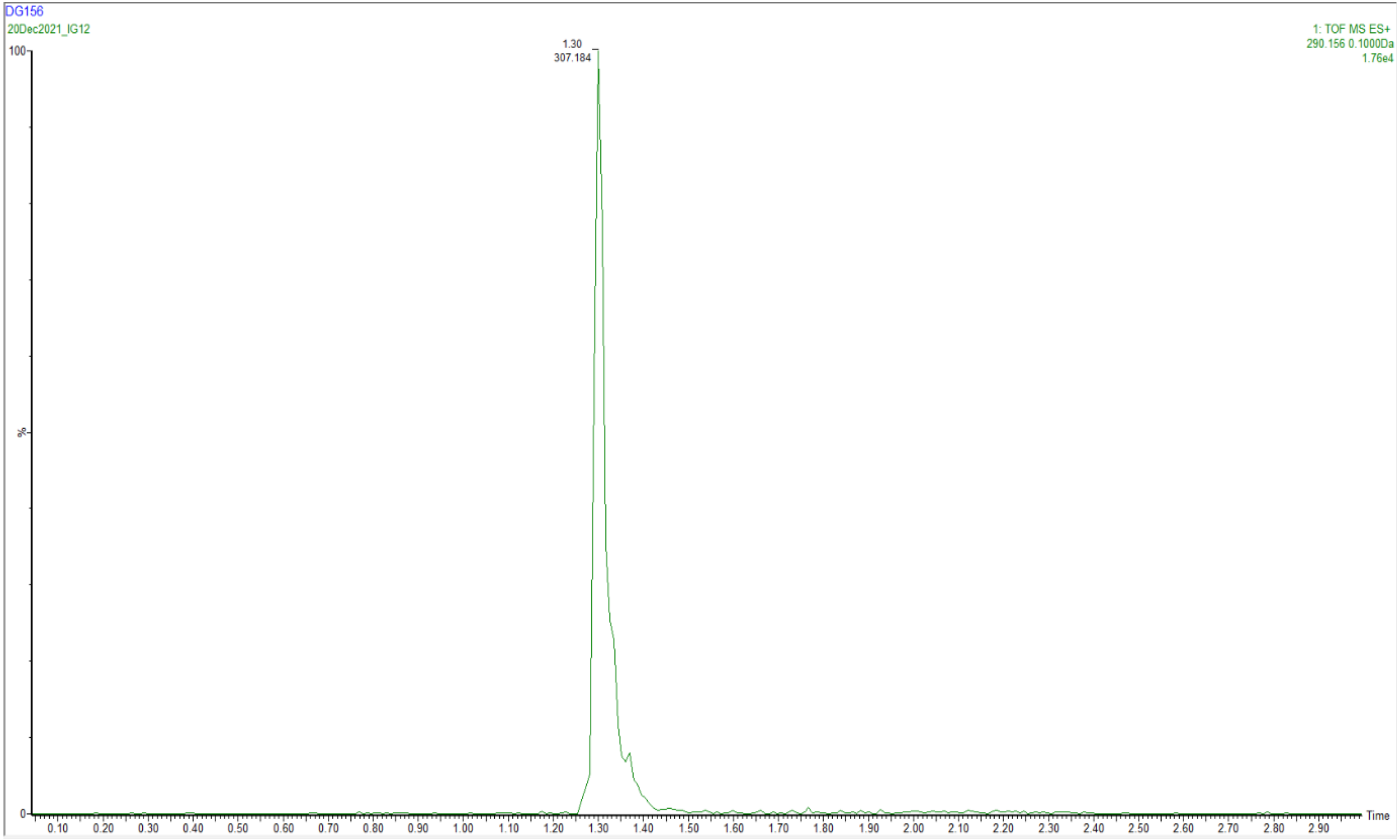

DG156
20Dec2021_IG12
1: TOF MS ES+
290.156 0.1000Da
1.76e4
1.30
307.184
100
%
0
Time
0.10 0.20 0.30 0.40 0.50 0.60 0.70 0.80 0.90 1.00 1.10 1.20 1.30 1.40 1.50 1.60 1.70 1.80 1.90 2.00 2.10 2.20 2.30 2.40 2.50 2.60 2.70 2.80 2.90

2-(benzo[d][1,3]dioxol-5-yl)-6-methyl-1,3,6,2-dioxazaborocane-4,8-dione 14

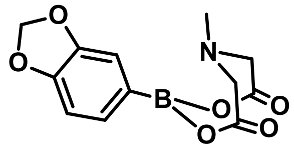

2-(benzo[*d*][1,3]dioxol-5-yl)-6-methyl-1,3,6,2-dioxazaborocane-4,8-dione
Chemical Formula: $C_{12}H_{12}BNO_6$
Molecular Weight: 277.0378

Yield = 187.0 mg (68%)

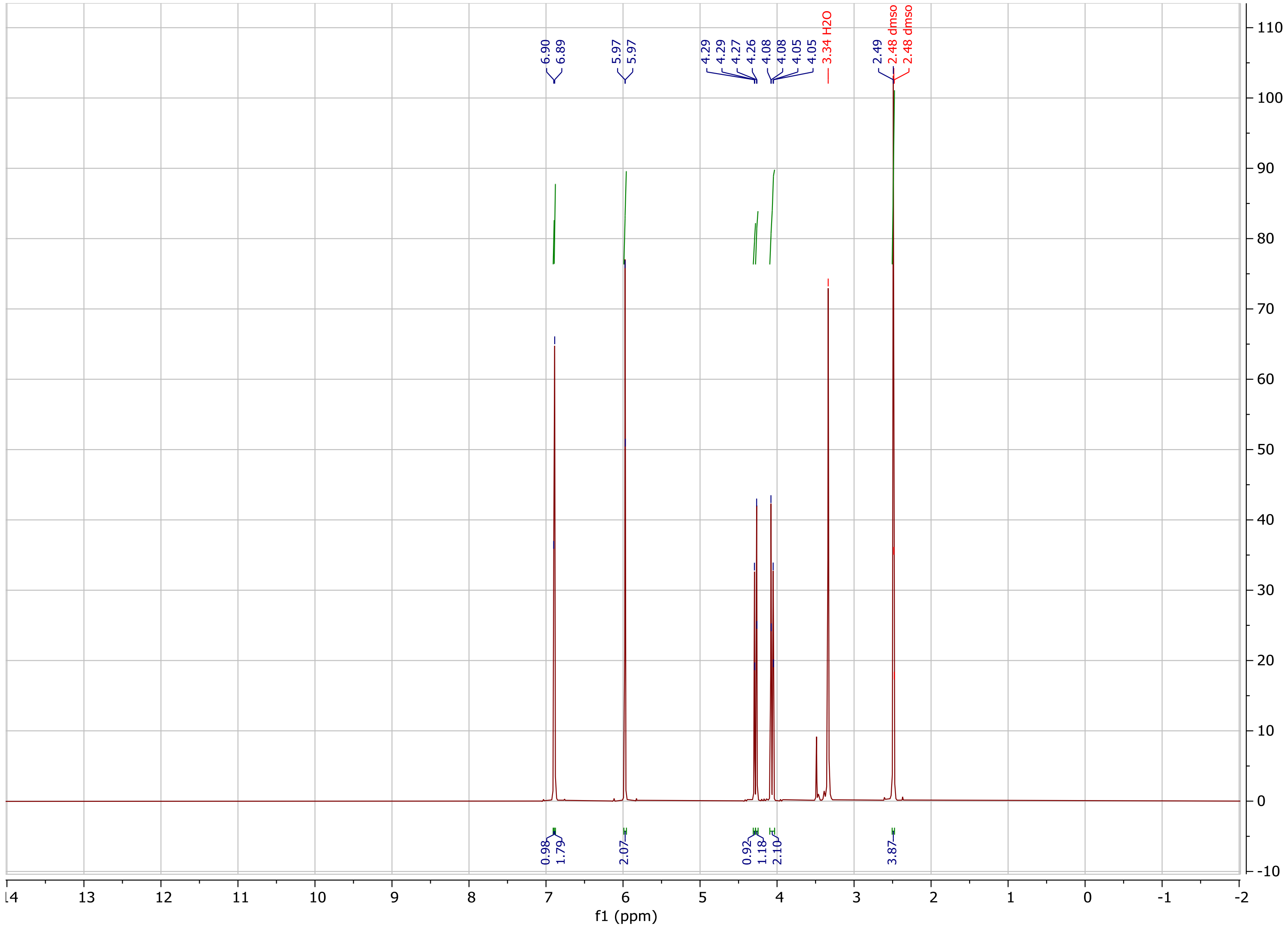
6.90
6.89
5.97
5.97
4.29
4.29
4.27
4.26
4.08
4.08
4.05
4.05
3.34 H2O
2.49
2.48 dmso
2.48 dmso
0.98
1.79
2.07
0.92
1.18
2.10
3.87
f1 (ppm)

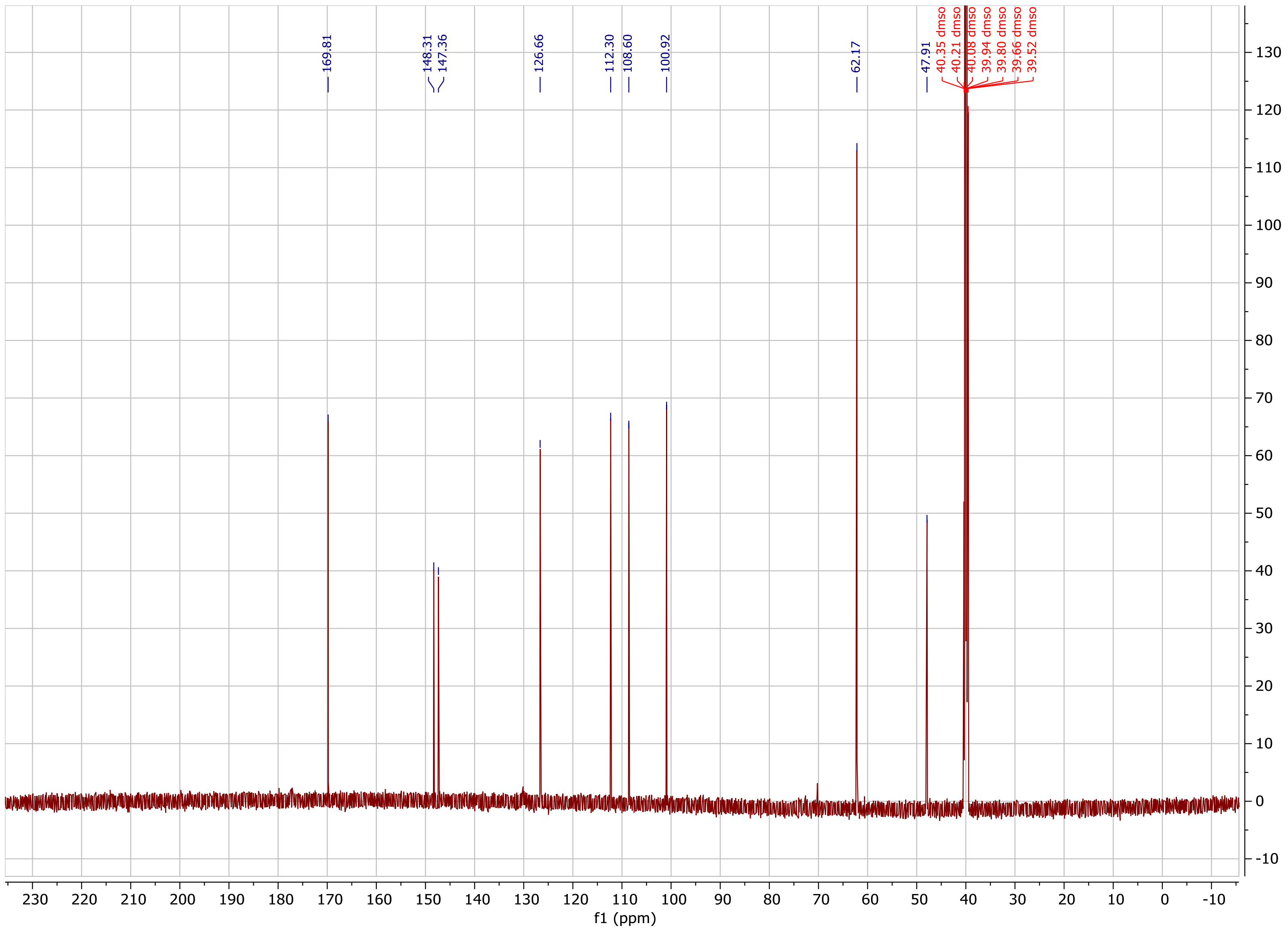
169.81
148.31
147.36
126.66
112.30
108.60
100.92
62.17
47.91
40.35 dmso
40.21 dmso
40.08 dmso
39.94 dmso
39.80 dmso
39.66 dmso
39.52 dmso
f1 (ppm)

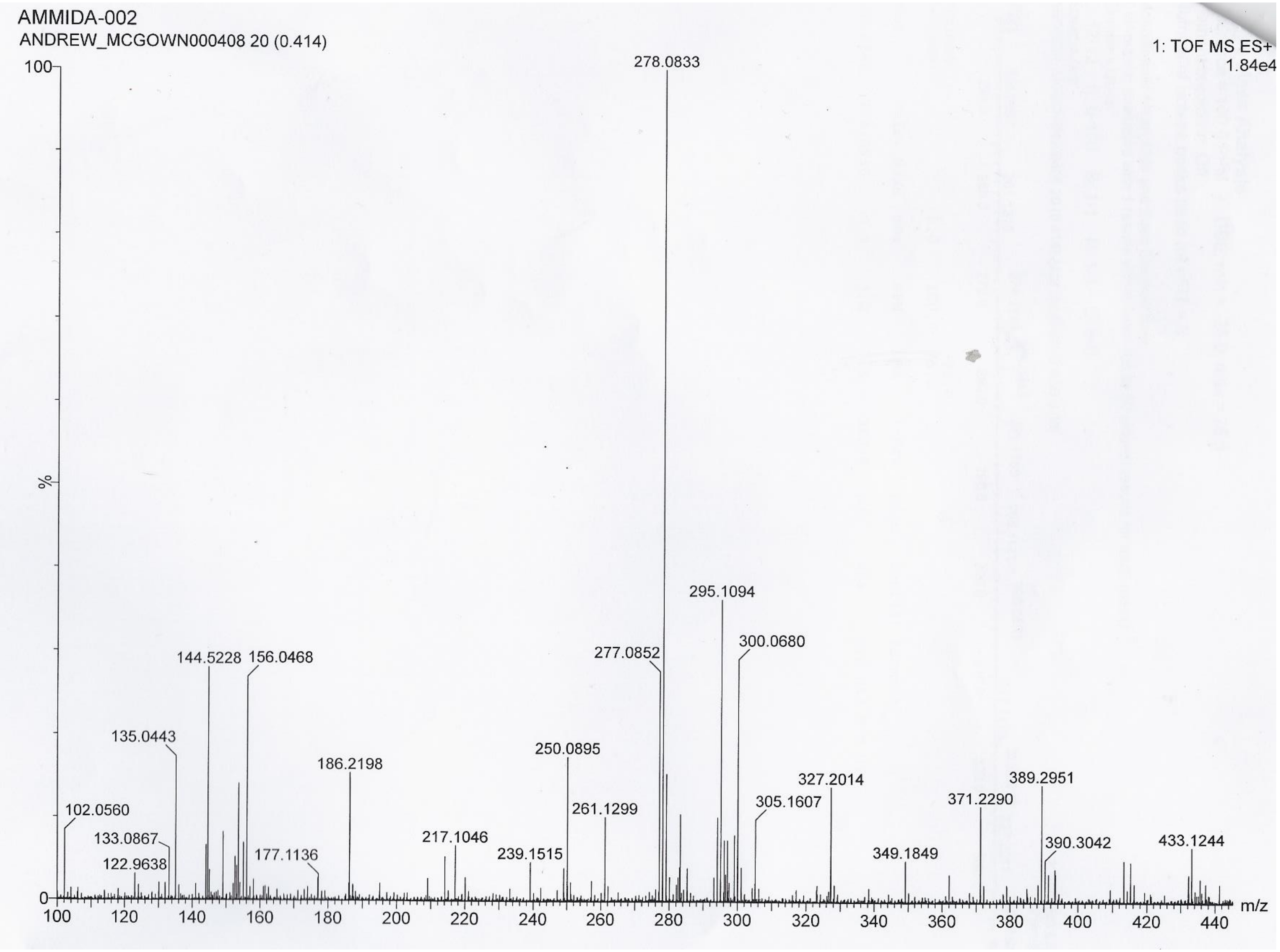

AMMIDA-002
ANDREW_MCGOWN000408 20 (0.414)
1: TOF MS ES+
1.84e4
100
%
0
278.0833
295.1094
300.0680
277.0852
144.5228
156.0468
135.0443
186.2198
250.0895
327.2014
389.2951
305.1607
371.2290
261.1299
102.0560
133.0867
217.1046
390.3042
433.1244
239.1515
349.1849
177.1136
122.9638
100
120
140
160
180
200
220
240
260
280
300
320
340
360
380
400
420
440
m/z

2-(3,5-Dimethylisoxazol-4-yl)-6-methyl-1,3,6,2-dioxazaborocane-4,8-dione 9 PEG

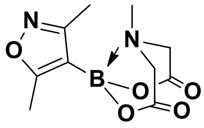

9

2-(3,5-dimethylisoxazol-4-yl)-6-methyl-1,3,6,2-dioxazaborocane-4,8-dione
Chemical Formula: $C_{10}H_{13}BN_2O_5$
Molecular Weight: 252.03

Yield = 80.2 mg (32%)

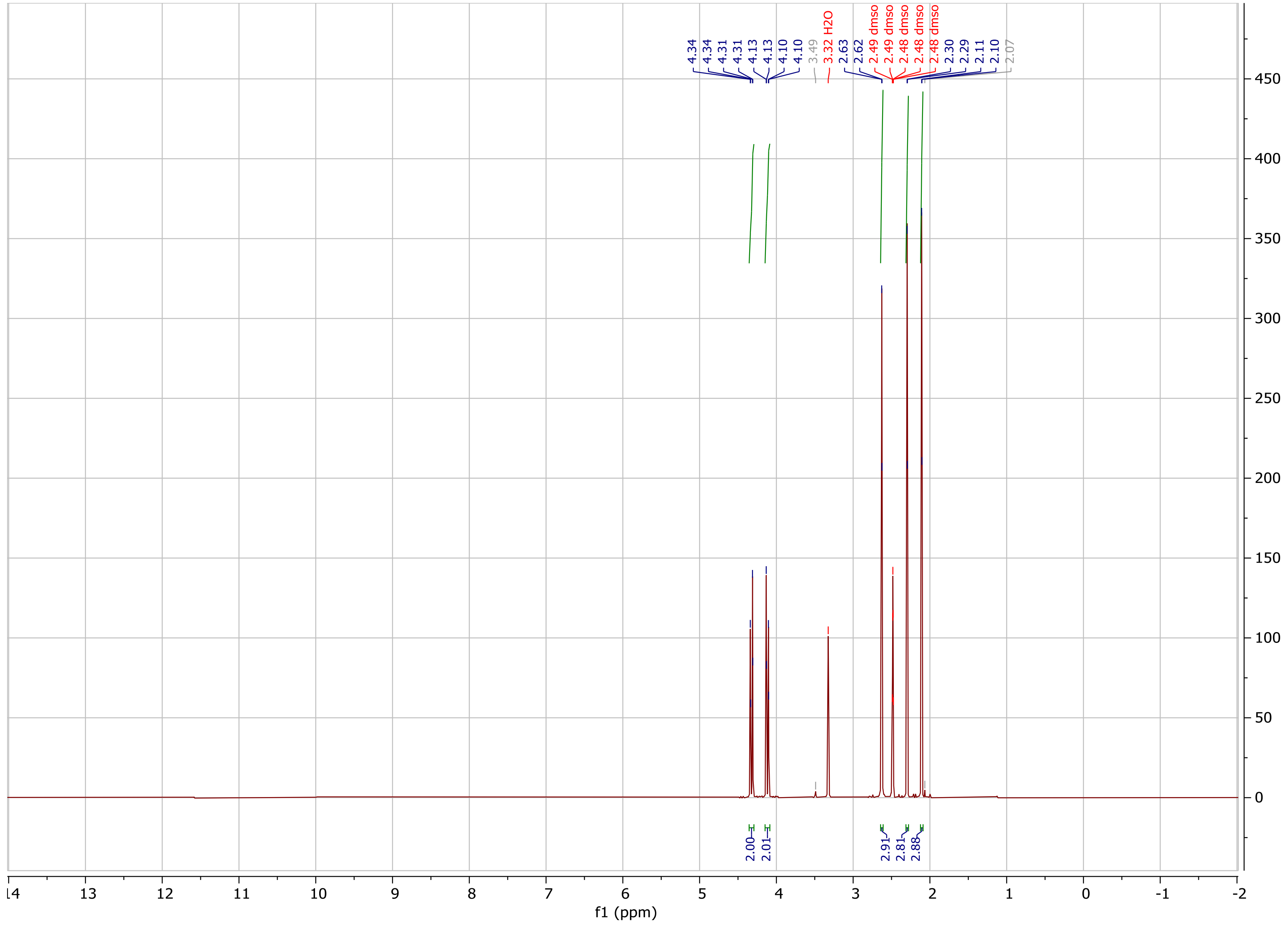
4.34
4.34
4.31
4.31
4.13
4.13
4.10
4.10
3.49
3.32 H2O
2.63
2.62
2.49 dmso
2.49 dmso
2.48 dmso
2.48 dmso
2.48 dmso
2.30
2.29
2.11
2.10
2.07
2.00
2.01
2.91
2.81
2.88
f1 (ppm)

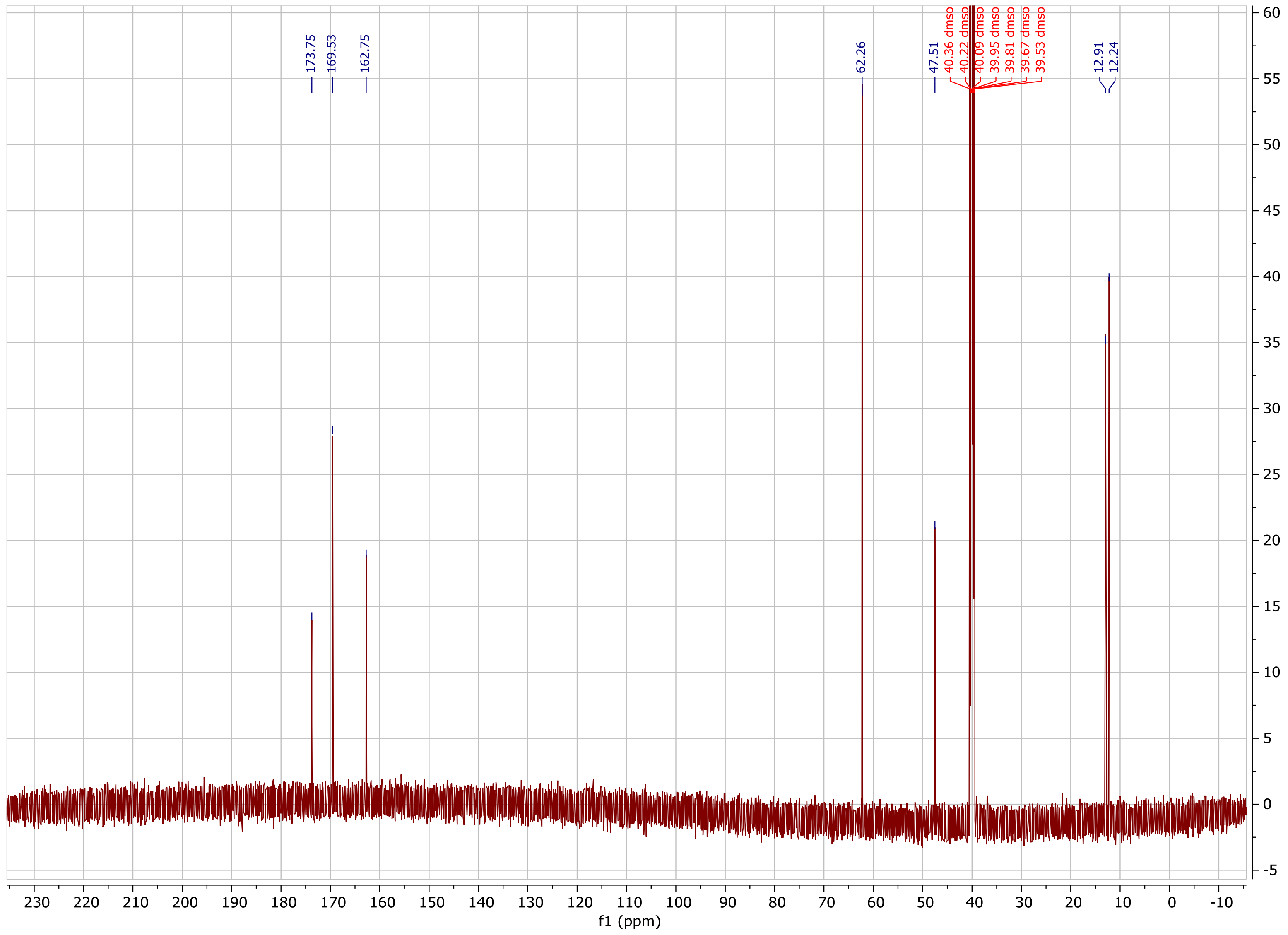
173.75
169.53
162.75
62.26
47.51
40.36 dmso
40.22 dmso
40.09 dmso
39.95 dmso
39.81 dmso
39.67 dmso
39.53 dmso
12.91
12.24
f1 (ppm)

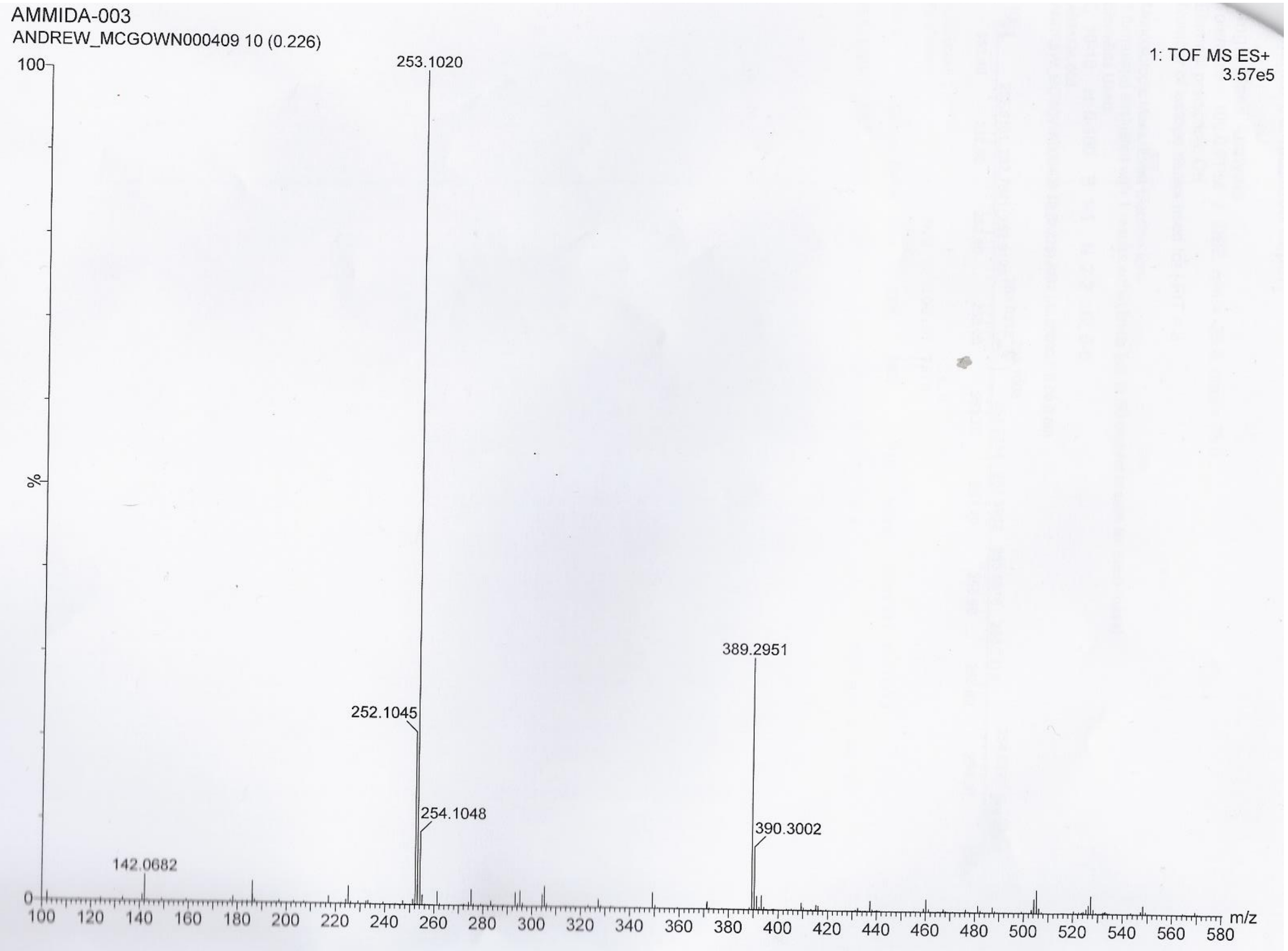
AMMIDA-003
ANDREW_MCGOWN000409 10 (0.226)
1: TOF MS ES+
3.57e5
100
%
0
253.1020
252.1045
254.1048
389.2951
390.3002
142.0682
m/z
100 120 140 160 180 200 220 240 260 280 300 320 340 360 380 400 420 440 460 480 500 520 540 560 580

2-(2-Hydroxyphenyl)-6-methyl-1,3,6,2-dioxazaborocane-4,8-dione 15

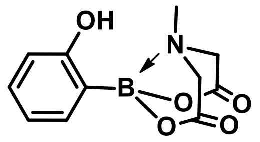

2-(2-hydroxyphenyl)-6-methyl-1,3,6,2-dioxazaborocane-4,8-dione
Chemical Formula: $C_{11}H_{12}BNO_5$
Molecular Weight: 249.0277

Yield = 26.2 mg (11%)

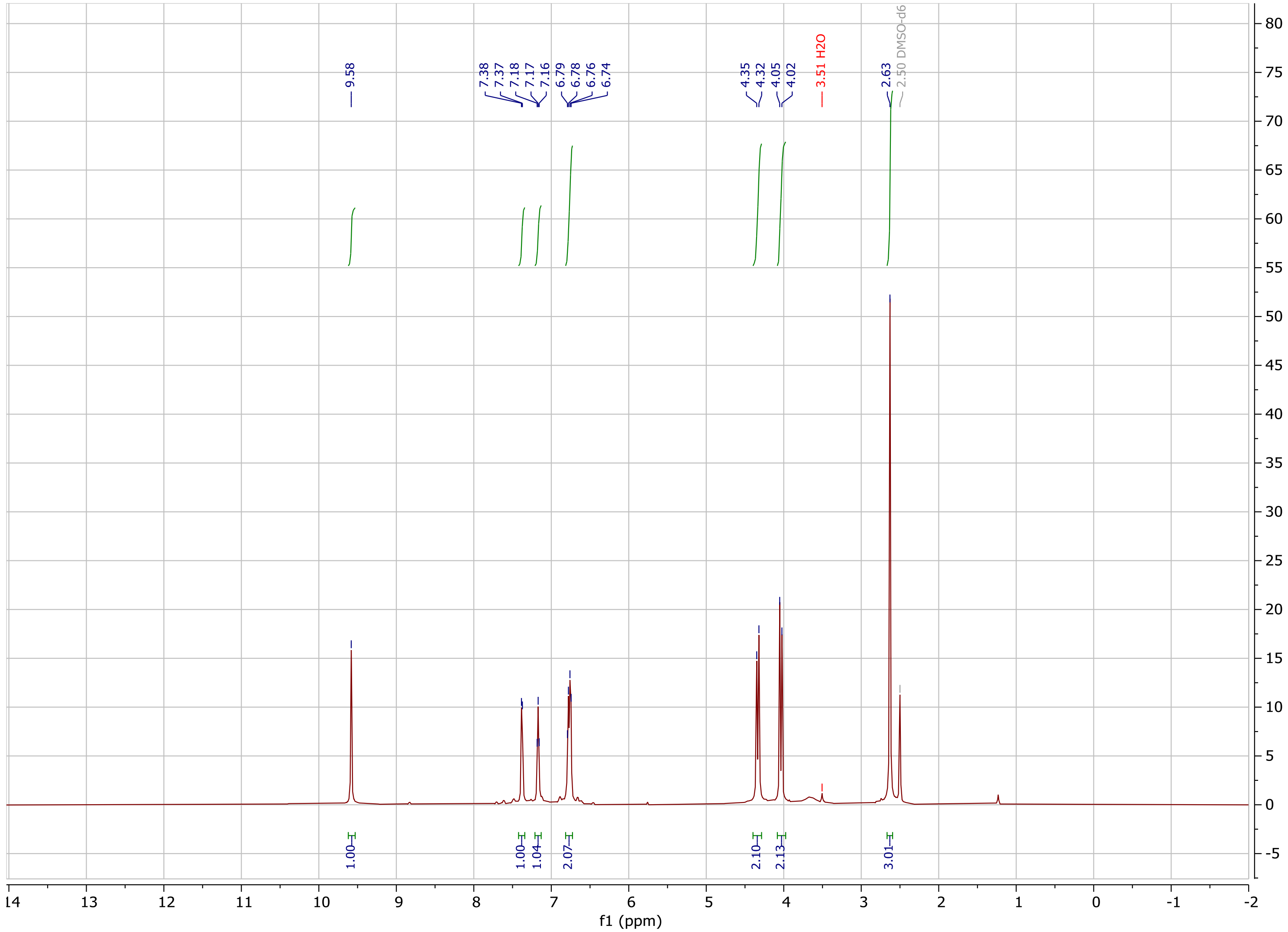

9.58
7.38
7.37
7.18
7.17
7.16
6.79
6.78
6.76
6.74
4.35
4.32
4.05
4.02
3.51 H2O
2.63
2.50 DMSO-d6
1.00
1.00
1.04
2.07
2.10
2.13
3.01
f1 (ppm)

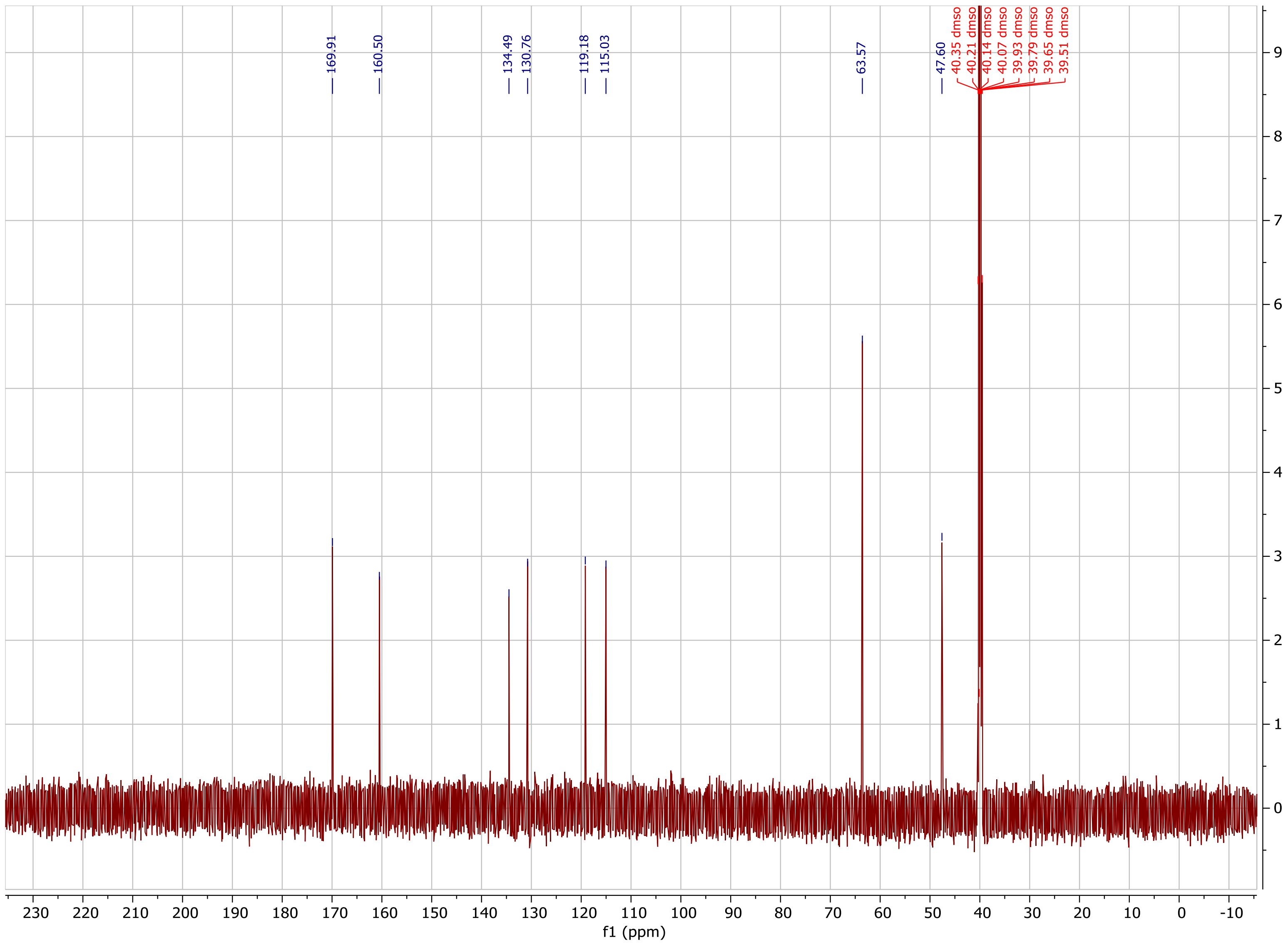
169.91
160.50
134.49
130.76
119.18
115.03
63.57
47.60
40.35 dmso
40.21 dmso
40.14 dmso
40.07 dmso
39.93 dmso
39.79 dmso
39.65 dmso
39.51 dmso
f1 (ppm)

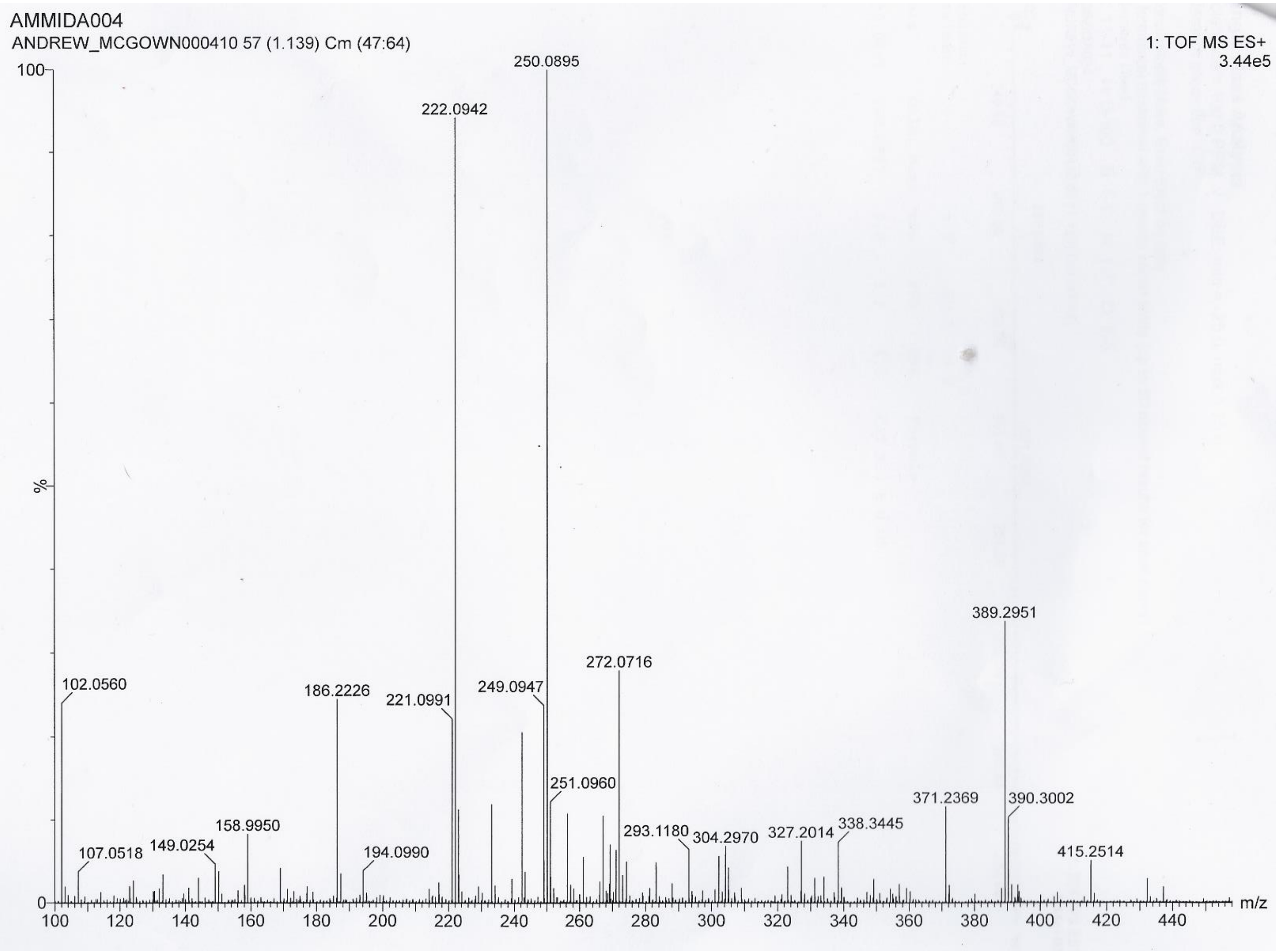
AMMIDA004
ANDREW_MCGOWN000410 57 (1.139) Cm (47:64)
1: TOF MS ES+
3.44e5
100
%
0
250.0895
222.0942
389.2951
272.0716
102.0560
186.2226
221.0991
249.0947
251.0960
371.2369
390.3002
158.9950
293.1180
304.2970
327.2014
338.3445
149.0254
107.0518
194.0990
415.2514
m/z
100
120
140
160
180
200
220
240
260
280
300
320
340
360
380
400
420
440

6-methyl-2-(perfluorophenyl)-1,3,6,2-dioxazaborocane-4,8-dione 8r

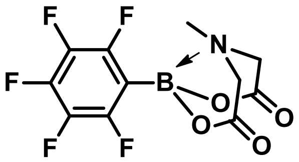

6-methyl-2-(perfluorophenyl)-1,3,6,2-dioxazaborocane-4,8-dione
Chemical Formula: $C_{11}H_7BF_5NO_4$
Molecular Weight: 322.9806

Yield = 68.2 mg (21%)

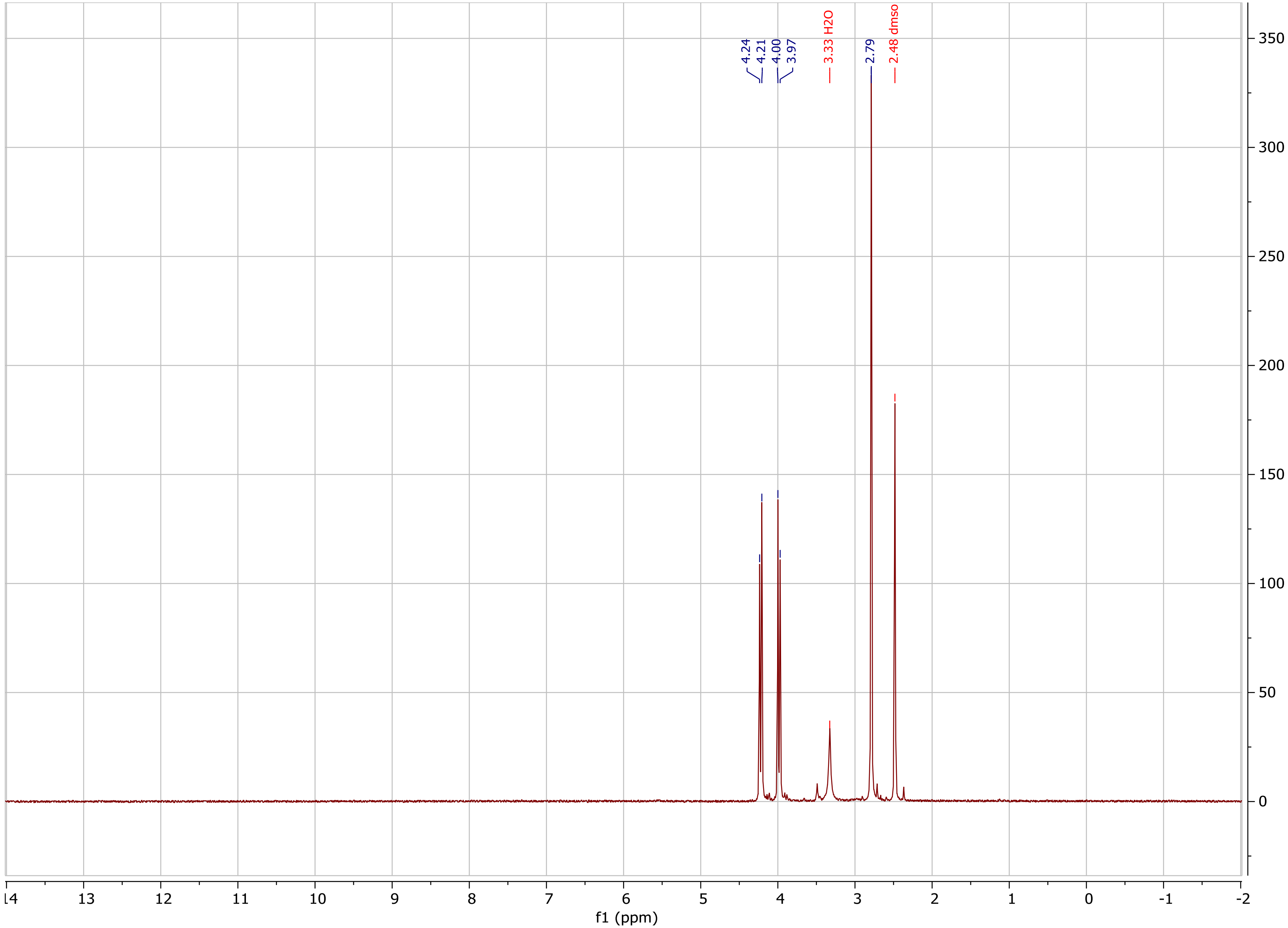
4.24
4.21
4.00
3.97
3.33 H2O
2.79
2.48 dmso
350
300
250
200
150
100
50
0
14
13
12
11
10
9
8
7
6
5
4
3
2
1
0
-1
-2
f1 (ppm)

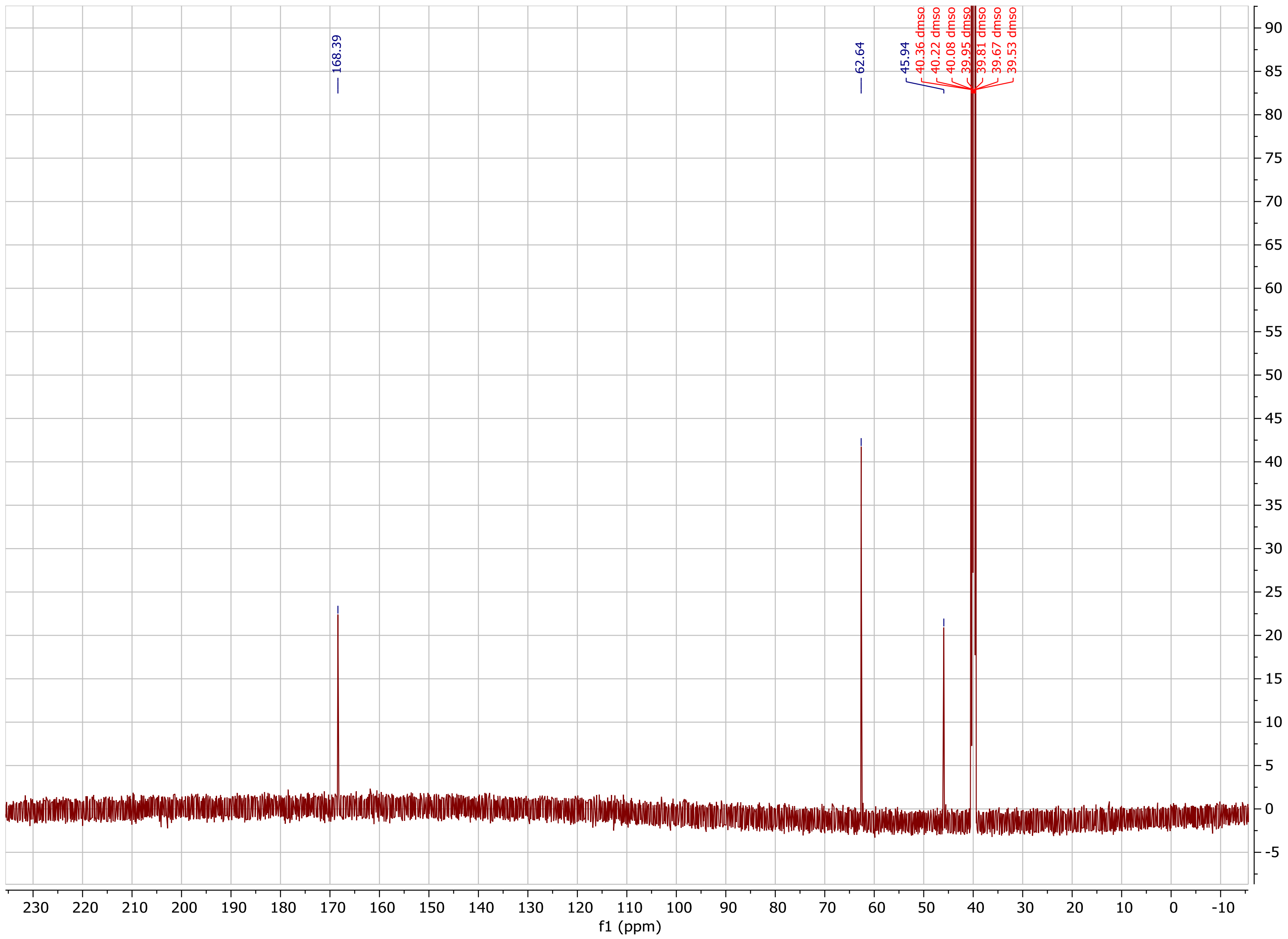

168.39
62.64
45.94
40.36 dmso
40.22 dmso
40.08 dmso
39.95 dmso
39.81 dmso
39.67 dmso
39.53 dmso
f1 (ppm)
